# Associations between sleep health and grey matter volume in the UK Biobank cohort (*n* = 33 356)

**DOI:** 10.1093/braincomms/fcad200

**Published:** 2023-07-12

**Authors:** Julian E Schiel, Sandra Tamm, Florian Holub, Roxana Petri, Hassan S Dashti, Katharina Domschke, Bernd Feige, Matthew O Goodman, Samuel E Jones, Jacqueline M Lane, Pietro-Luca Ratti, David W Ray, Susan Redline, Dieter Riemann, Martin K Rutter, Richa Saxena, Claire E Sexton, Masoud Tahmasian, Heming Wang, Michael N Weedon, Antoine Weihs, Simon D Kyle, Kai Spiegelhalder

**Affiliations:** Department of Psychiatry and Psychotherapy, Faculty of Medicine, Medical Center—University of Freiburg, Hauptstraße 5, 79104 Freiburg, Germany; Department of Clinical Neuroscience, Karolinska Institutet, Retzius väg 8, 17165 Stockholm, Sweden; Department of Psychiatry, University of Oxford, Warneford Lane, OX3 7JX Oxford, UK; Department of Psychiatry and Psychotherapy, Faculty of Medicine, Medical Center—University of Freiburg, Hauptstraße 5, 79104 Freiburg, Germany; Department of Psychiatry and Psychotherapy, Faculty of Medicine, Medical Center—University of Freiburg, Hauptstraße 5, 79104 Freiburg, Germany; Program in Medical and Population Genetics, Broad Institute of MIT and Harvard, Main St. 415, Cambridge, MA 02142, USA; Center for Genomic Medicine, Massachusetts General Hospital, Cambridge St. 185, Boston, MA 02114, USA; Department of Anesthesia, Critical Care and Pain Medicine, Massachusetts General Hospital and Harvard Medical School, Fruit St. 55, Boston, MA 02114, USA; Department of Psychiatry and Psychotherapy, Faculty of Medicine, Medical Center—University of Freiburg, Hauptstraße 5, 79104 Freiburg, Germany; Department of Psychiatry and Psychotherapy, Faculty of Medicine, Medical Center—University of Freiburg, Hauptstraße 5, 79104 Freiburg, Germany; Division of Sleep and Circadian Disorders, Brigham and Women’s Hospital and Harvard Medical School, Francis St. 75, Boston, MA 02115, USA; Institute for Molecular Medicine (FIMM), University of Helsinki, Tukholmankatu 8, 00290 Helsinki, Finland; Program in Medical and Population Genetics, Broad Institute of MIT and Harvard, Main St. 415, Cambridge, MA 02142, USA; Center for Genomic Medicine, Massachusetts General Hospital, Cambridge St. 185, Boston, MA 02114, USA; Department of Anesthesia, Critical Care and Pain Medicine, Massachusetts General Hospital and Harvard Medical School, Fruit St. 55, Boston, MA 02114, USA; Neurocenter of Southern Switzerland, Regional Hospital of Lugano, Viale Officina 3, 6500 Bellinzona, Switzerland; Division of Endocrinology, Diabetes & Gastroenterology, School of Medical Sciences, Faculty of Biology, Medicine and Health, University of Manchester, Grafton St. 46, M13 9NT Manchester, UK; Division of Sleep and Circadian Disorders, Brigham and Women’s Hospital and Harvard Medical School, Francis St. 75, Boston, MA 02115, USA; Department of Psychiatry and Psychotherapy, Faculty of Medicine, Medical Center—University of Freiburg, Hauptstraße 5, 79104 Freiburg, Germany; Faculty of Biology, Medicine and Health, Centre for Biological Timing, University of Manchester, Grafton St. 46, M13 9NT Manchester, UK; Diabetes, Endocrinology and Metabolism Centre, Manchester University NHS Foundation Trust, Manchester Academic Health Science Centre, Grafton St. 46, M13 9NT Manchester, UK; Program in Medical and Population Genetics, Broad Institute of MIT and Harvard, Main St. 415, Cambridge, MA 02142, USA; Center for Genomic Medicine, Massachusetts General Hospital, Cambridge St. 185, Boston, MA 02114, USA; Department of Anesthesia, Critical Care and Pain Medicine, Massachusetts General Hospital and Harvard Medical School, Fruit St. 55, Boston, MA 02114, USA; Department of Psychiatry, University of Oxford, Warneford Lane, OX3 7JX Oxford, UK; Department of Neurology, Global Brain Health Institute, Memory and Aging Center, University of California, Nelson Rising Lane 675, San Francisco, CA 94158, USA; Institute of Neuroscience and Medicine, Brain and Behavior (INM-7), Research Center Jülich, Wilhelm-Johnen-Straße 14.6y, 52428 Jülich, Germany; Medical Faculty, Institute for Systems Neuroscience, Heinrich-Heine University Düsseldorf, Moorenstraße 5, 40225 Düsseldorf, Germany; Program in Medical and Population Genetics, Broad Institute of MIT and Harvard, Main St. 415, Cambridge, MA 02142, USA; Division of Sleep and Circadian Disorders, Brigham and Women’s Hospital and Harvard Medical School, Francis St. 75, Boston, MA 02115, USA; Department of Population and Quantitative Health Sciences, Case Western Reserve University, Euclid Ave. 10900, Cleveland, OH 44106-7288, USA; Genetics of Complex Traits, University of Exeter Medical School, Royal Devon & Exeter Hospital, Barrack Road, EX2 5DW Exeter, UK; Department of Psychiatry and Psychotherapy, University Medicine Greifswald, Ellernholzstraße 1-2, 17475 Greifswald, Germany; Nuffield Department of Clinical Neurosciences, Sleep and Circadian Neuroscience Institute (SCNi), University of Oxford, South Parks Road, OX1 3QU Oxford, UK; Department of Psychiatry and Psychotherapy, Faculty of Medicine, Medical Center—University of Freiburg, Hauptstraße 5, 79104 Freiburg, Germany

**Keywords:** sleep health, grey matter volume, UK Biobank, sleep duration, basal ganglia

## Abstract

As suggested by previous research, sleep health is assumed to be a key determinant of future morbidity and mortality. In line with this, recent studies have found that poor sleep is associated with impaired cognitive function. However, to date, little is known about brain structural abnormalities underlying this association. Although recent findings link sleep health deficits to specific alterations in grey matter volume, evidence remains inconsistent and reliant on small sample sizes. Addressing this problem, the current preregistered study investigated associations between sleep health and grey matter volume (139 imaging-derived phenotypes) in the UK Biobank cohort (33 356 participants). Drawing on a large sample size and consistent data acquisition, sleep duration, insomnia symptoms, daytime sleepiness, chronotype, sleep medication and sleep apnoea were examined. Our main analyses revealed that long sleep duration was systematically associated with larger grey matter volume of basal ganglia substructures. Insomnia symptoms, sleep medication and sleep apnoea were not associated with any of the 139 imaging-derived phenotypes. Short sleep duration, daytime sleepiness as well as late and early chronotype were associated with solitary imaging-derived phenotypes (no recognizable pattern, small effect sizes). To our knowledge, this is the largest study to test associations between sleep health and grey matter volume. Clinical implications of the association between long sleep duration and larger grey matter volume of basal ganglia are discussed. Insomnia symptoms as operationalized in the UK Biobank do not translate into grey matter volume findings.

## Teaser

Investigating associations between sleep health and grey matter volume in over 33 000 individuals, Schiel *et al*. report that insomnia symptoms do not translate directly into brain morphometry. Long sleep duration was associated with larger grey matter volume of basal ganglia substructures, possibly indicating early stage sleep apnoea in old age.

## Graphical Abstract

**Graphical Abstract:**
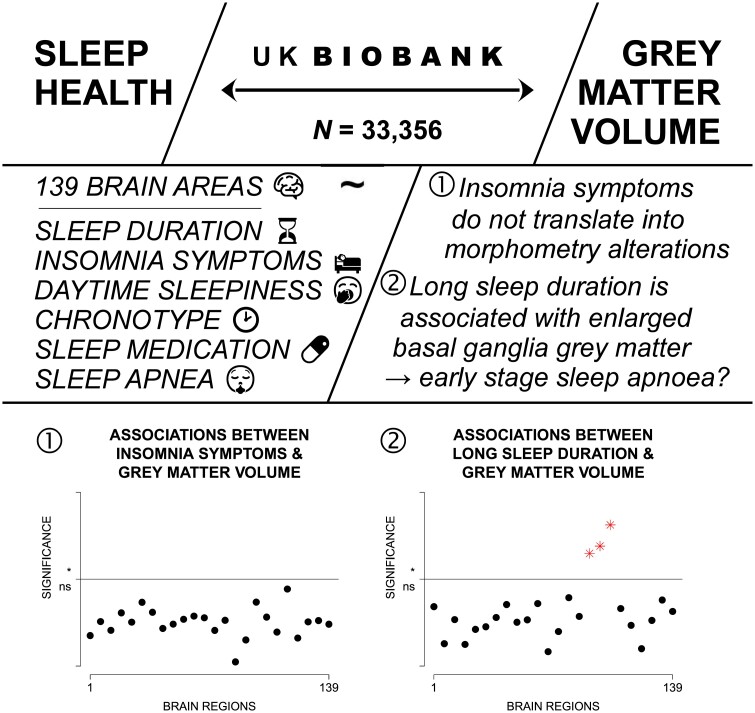

## Introduction

Sleep is a complex physiological process regulated by various brain circuits and neurotransmitter systems.^1^ Decades of empirical research have clearly demonstrated that different aspects of sleep health (SH) are related to future morbidity and mortality, e.g. short and long sleep duration,^2,3^ difficulties initiating or maintaining sleep,^4‐6^ sleep medication use,^7^ excessive daytime sleepiness^8^ and the sleep apnoea syndrome.^9^ The same SH dimensions, as well as chronotype, have been linked to impaired cognitive function, in particular in the domains of attention, memory and executive functions.^10‐13^ However, it is unclear whether neurostructural abnormalities underlie the relationships between SH dimensions and cognitive function. Several small-scale cross-sectional case–control studies have supported this hypothesis, however, the results so far are somewhat inconclusive. Self-reported short sleep duration has been linked to cortical thinning within fronto-temporal regions,^14^ hippocampal volume loss^15^ and increased rates of ventricular expansion.^16^ Insomnia has been found to be associated with smaller hippocampal volume,^17^ smaller frontal grey matter volume (GMV)^18‐20^ and larger anterior cingulate cortex volume.^21^ Daytime sleepiness has been reported to be related to a reduction of GMV in the medial orbitofrontal cortex.^22^ Sleep apnoea syndrome has been found to be associated with smaller GMV in several cortical and subcortical areas including the anterior cingulate cortex, frontal and temporal lobes, cortical motor areas and the cerebellum.^23,24^ Markers of sleep apnoea severity as well as sleep-disordered breathing have been found to be associated with ‘larger’ GMV and greater amyloid burden in multiple cortical and subcortical brain regions.^25,26^ In addition, associations have been reported between brain structure and sleep quality^27,28^ and objectively-determined sleep fragmentation.^29^

However, other studies have found conflicting results or have reported null findings.^28,30,31^ After reviewing the methodological quality of brain imaging studies in this field, it has been suggested that the observed inconsistencies are likely to be related to small sample sizes and other sources of heterogeneity such as sample characteristics, data acquisition and image processing.^32,33^ Thus, in light of recent doubts about replicability and low power in neuroimaging studies,^34,35^ the current study sought to investigate the independent associations between several SH variables (sleep duration, insomnia symptoms, daytime sleepiness and chronotype) and brain morphometry in a large sample of individuals of the UK Biobank.

## Materials and methods

### Preregistration

Full details of the current analysis plan were officially preregistered at Open Science Framework (https://osf.io/vyjfw) on 14 May 2020. As a consequence, all (non-explorative) analysis steps had been determined before the current dataset was downloaded from the UK Biobank (UKBB) server on 2 May 2022.

### Participants

The UKBB project is a prospective epidemiological study. Between 2006 and 2010, over 500 000 adults aged 40 to 69 years were enrolled at various locations in the UK (initial visit = Instance 0).^36^ In 2014, multimodal magnetic resonance imaging (MRI) was introduced with a planned subgroup size of *n* = 100 000 (first imaging visit = Instance 2). By the time the current analysis was conducted, imaging data were available for 42 801 participants. However, to eliminate systematic biases, participants were excluded in advance if they reported a neurological condition (*n* = 1026; see Supplementary Table 1 for a list of conditions). Furthermore, participants were excluded from the remaining sample if they lacked data or responded ‘do not know’ or ‘prefer not to answer’ at Instance 2 for insomnia symptoms (*n* = 311), sleep duration (*n* = 387), excessive daytime sleepiness (*n* = 330), chronotype (*n* = 3972), socioeconomic status (*n* = 40), depressive symptoms (*n* = 1321), body mass index (BMI, *n* = 1418) or qualifications (*n* = 2994) leaving a study sample of 33 356 participants. All research procedures within the UKBB project are approved by the NHS National Research Ethics Service (Ref. 11/NW/0382), and all participants had to give written informed consent before inclusion. Ethical standards are continuously controlled by a concerned Ethics Advisory Committee (EAC, http://www.ukbiobank.ac.uk/ethics), based on a project-specific Ethics and Governance Framework (given in full at http://www.ukbiobank.ac.uk/wp-content/uploads/2011/05/EGF20082.pdf). The current analyses were conducted under UK Biobank application number 6818.

### Sleep-related variables

The current operationalization of SH is based on previous considerations (representing central aspects of SH, maintaining consistency with previous SH studies and providing a multifaceted picture of SH).^37^ Accordingly, the selected variables were sleep duration, insomnia symptoms, daytime sleepiness and chronotype.

Sleep duration was assessed by asking participants ‘About how many hours sleep do you get in every 24 hours? (please include naps)’. In light of previously established U-shape relationships with health and cognition,^38^ sleep duration was categorized into short (<7 hours), normal (7–9 hours) and long (>9 hours) referring to recent guidelines.^39^ Insomnia symptoms were assessed by asking participants ‘Do you have trouble falling asleep at night or do you wake up in the middle of the night?’ with responses ‘never/rarely’, ‘sometimes’ and ‘usually’. Participants were categorized as having frequent insomnia symptoms if they answered ‘usually’ to this question, while the remaining participants made up the group without frequent insomnia symptoms. Daytime sleepiness was assessed by asking participants ‘How likely are you to doze off or fall asleep during the daytime when you don’t mean to? (e.g. when working, reading or driving)’ with responses ‘never/rarely’, ‘sometimes’ and ‘often’. Participants were categorized as having excessive daytime sleepiness if they answered ‘often’, the remaining participants made up the group without excessive daytime sleepiness. No participant answered ‘all of the time’. Dichotomization of the SH dimensions insomnia symptoms and daytime sleepiness served for comparison between participants with and without clinically relevant symptoms.^11,24^ Chronotype was assessed by asking ‘Do you consider yourself to be definitely a “morning” person/more a “morning” than an “evening” person/more an “evening” than a “morning” person/definitely an “evening” person?’. Participants were categorized as early chronotype if they answered ‘definitely a “morning” person’ and as late chronotype if they answered ‘definitely an “evening” person’, the remaining participants made up the reference group (intermediate chronotype). Complementing sleep-related variables were sleep medication use and sleep apnoea. The former (hypnotics and sedatives as specified in Dashti *et al.*^40^; see Supplementary Table 2 for a complete list) was assessed by reports to a research nurse. The latter was assessed by means of self-reported non-cancer illness codes.

### Magnetic resonance imaging

MRI acquisition protocols, processing pipelines and derived measures of brain structure [imaging-derived phenotypes (IDPs)] for the brain imaging project of the UK Biobank have been described previously in full detail,^41^ with documentation available online (http://biobank.ctsu.ox.ac.uk/crystal/refer.cgi?id=2367 and http://biobank.ctsu.ox.ac.uk/crystal/refer.cgi?id=1977). Of particular importance for the current analysis, FMRIB’s Automated Segmentation Tool (FAST^42^) was used for brain segmentation to generate 139 IDPs within regions-of-interest. These regions-of-interest were defined in MNI152 space, combining parcellations from the HarvardOxford cortical and subcortical atlases (https://fsl.fmrib.ox.ac.uk/fsl/fslwiki/Atlases) and the Diedrichsen cerebellar atlas (http://www.diedrichsenlab.org/imaging/propatlas.htm). A full list of these IDPs is provided in Supplementary Table 3.

### Covariates

Socioeconomic status was quantified by the Townsend index of material deprivation. Due to skewed distribution, the measure was log-transformed with an ln(*x* + 7) equation (minimum of non-transformed index: −6.26). Educational qualifications were assessed by asking ‘Which of the following qualifications do you have? (You can select more than one)’ with responses ‘College or University degree’, ‘A levels/AS levels or equivalent’, ‘O levels/GCSEs or equivalent’, ‘CSEs or equivalent’, ‘NVQ or HND or HNC or equivalent’ and/or ‘Other professional qualifications eg: nursing, teaching’. Participants were categorized as academics if they answered ‘College or University degree’, while the remaining participants made up the group without academic training. Depressive symptoms were assessed by asking ‘Over the past two weeks, how often have you felt down, depressed or hopeless?’ with responses ‘not at all’, ‘several days’, ‘more than half the days’ or ‘nearly every day’. Participants were categorized as having depressive symptoms if they answered ‘several days’, ‘more than half the days’ or ‘nearly every day’, the remaining participants made up the group without depressive symptoms. Intracranial brain volume (ICV; volume of grey and white matter plus volume of ventricular cerebrospinal fluid), BMI, sex and age as well as psychotropic medication use (antidepressants, antipsychotics and mood stabilizers; see Supplementary Table 4) were also incorporated as covariates in the analyses. All data were taken, if available, from Instance 2.

### Statistical analysis

Participants who responded ‘do not know’ or ‘prefer not to answer’ at Instance 2 (if data available) were excluded from the current analysis (insomnia symptoms: *n* = 3, sleep duration: *n* = 135, excessive daytime sleepiness: *n* = 69, chronotype: *n* = 4434, depressive symptoms: *n* = 1285 and educational qualifications: *n* = 3476). Descriptive data are presented as mean values and standard deviations. Associations between SH variables and brain morphometry were analysed using multivariate linear regression models (LMs) for each IDP, with sleep duration (three factor levels, reference category: normal sleep duration), insomnia symptoms (two factor levels), excessive daytime sleepiness (two factor levels), chronotype (three factor levels, reference category: intermediate type), sleep medication use (two factor levels) and sleep apnoea (two factor levels) as predictor variables and the respective IDP as dependent variable.

Socioeconomic status, educational qualifications, depressive symptoms, ICV, BMI, sex, age and psychotropic medication use were incorporated as covariates. This way, all variables were used as specified in our preregistration, referring to our previous studies on SH.^11,37^ Given the analysis of 139 IDPs, the alpha level was set at *P* < 3.6 × 10^−4^ (0.05/139; two-tailed) for all analyses.

Following the principle of parsimony for statistical models, variables were introduced gradually starting with a baseline model (SH variables only: insomnia symptoms, sleep duration, excessive daytime sleepiness, chronotype; LM1), continuing with an adjusted model (adding basic demographic covariates: socioeconomic status, level of education, ICV, BMI, sex, age; LM2) and ending with a fully developed model comprising all described variables (adding remaining sleep-related variables and clinical covariates: sleep medication use, sleep apnoea, depressive symptoms, psychotropic medication use; LM3, see Supplementary Table 5). Hereby, the prioritization of self-reported insomnia symptoms, sleep duration, excessive daytime sleepiness, and chronotype over sleep medication use and sleep apnoea is supposed to reflect the study’s primary focus on psychological aspects of SH rather than on pharmaceutical aspects and sleep-related breathing disorders. All models were compared by the means of partial *F*-tests.

As a sensitivity analysis, we examined (i) if an alternative operationalization of sleep apnoea might help to detect the presence or absence of sleep apnoea more accurately (see Supplementary Fig. 10). At this, our rationale was to reduce the amount of undetected positives in the control group (by excluding participants who reported snoring ‘and’ excessive daytime sleepiness) and to increase the amount of detected positives in the sleep apnoea group (by including combinations of indirectly indicative variables). Results relying on the non-cancer illness code variable only might have been prone to underestimating the actual prevalence of sleep apnoea in the current sample. Additionally, (ii) we addressed possible collinearity effects underlying the original analysis by implementing separate linear models for each SH variable. With respect to previous findings of age- and sex-dependent associations between sleep and brain imaging variables in the UK Biobank,^43^ we also implemented exploratory linear models comprising interaction effects between age or sex and sleep-related variables (iii).

## Results

The sample consisted of 17 936 female (53.8%) and 15 420 male (46.2%) participants (=33 356) with a mean age of 63.5 ± 7.6 years. Further sample characteristics are described in Table 1.

**Table 1:** Sample characteristics

	Short, *n* (%)	Normal, *n* (%)	Long, *n* (%)
Sleep duration	7871 (23.6)	25 102 (75.3)	383 (1.1)
	**Early, *n* (%)**	**Intermediate, *n* (%)**	**Late, *n* (%)**
Chronotype	9235 (27.7)	20 911 (62.7)	3210 (9.6)
	**Yes, *n* (%)**	**No, *n* (%)**	
Insomnia symptoms	10 438 (31.3)	22 918 (68.7)	
Excessive daytime sleepiness	7564 (22.7)	25 792 (77.3)	
Sleep medication use	90 (0.3)	33 266 (99.7)	
Sleep apnoea	133 (0.4)	33 223 (99.6)	
Psych. medication use	2016 (6)	31 340 (94.0)	
Depressive symptoms	5914 (17.7)	27 442 (82.3)	

**Figure 1:**
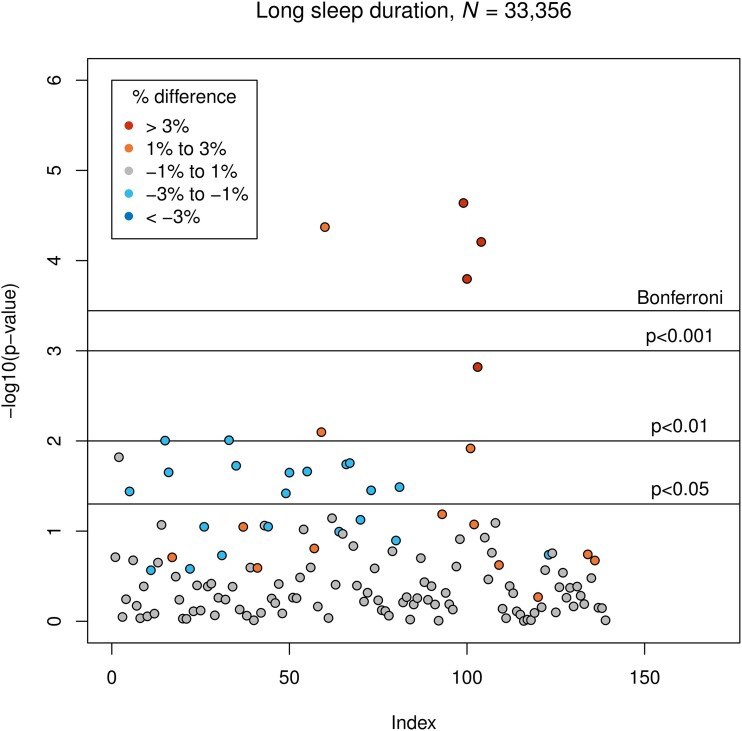
**Results of LM3 (one-sixth; adjusted linear model): associations between long sleep duration and GMV of all 139 IDPs as indexed in** Supplementary Table 3. The vertical axis indicates the *P*-value, and colours indicate the direction of difference (plus percentage difference). Significant associations after Bonferroni correction: IDPs 60, 99, 100 and 104 (see Supplementary Table 3).

Long sleep duration was associated with larger GMV of the right cingulate gyrus, posterior division (*β* = 118.2, corresponding to 2.2% difference to the normal sleep duration group; *P* = 4.3 × 10^−5^), the left caudate (*β* = 138.4, corresponding to 4.5%; *P* = 2.3 × 10^−5^), the right caudate (*β* = 128.4, corresponding to 3.9%; *P* = 1.6 × 10^−4^) and the right pallidum (*β* = 8.9, corresponding to 14.9%; *P* = 6.2 × 10^−5^). Associations between long sleep duration and GMVs are presented in Fig. 1.

**Figure 2:**
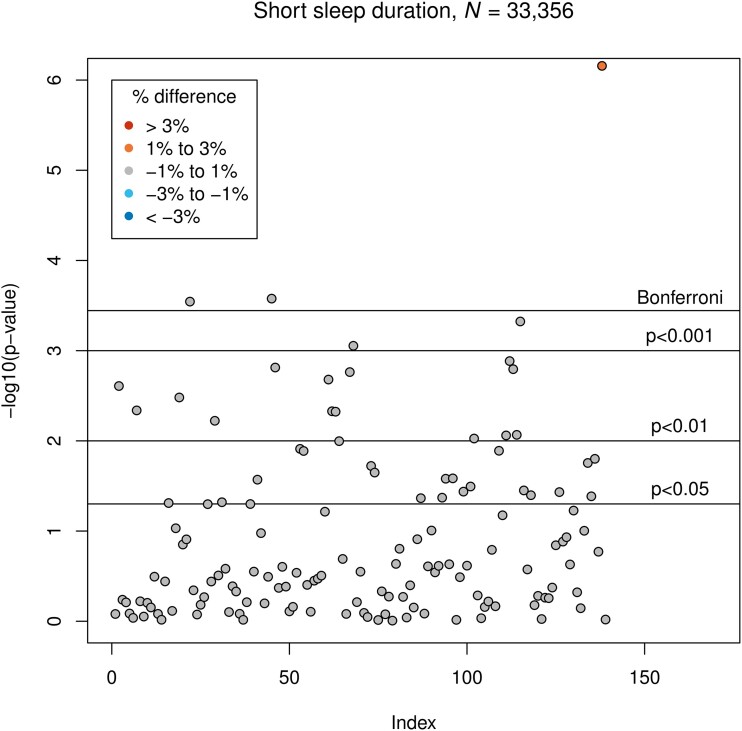
**Results of LM3 (two-sixths; adjusted linear model): associations between short sleep duration and GMV.** The vertical axis indicates the *P*-value, and colours indicate the direction of difference (plus percentage difference). Significant associations after Bonferroni correction: IDPs 22, 45 and 138 (see Supplementary Table 3).

Short sleep duration was associated with smaller GMV of the right middle temporal gyrus, anterior division (*β* = −13.7; corresponding to −0.9% difference to the normal sleep duration group; *P* = 2.9 × 10^−4^) and the left lateral occipital cortex, inferior division (*β* = −51.0, corresponding to −0.7%; *P* = 2.6 × 10^−4^), as well as with larger GMV of the cerebellar vermis lobule X (*β* = 2.7, corresponding to 1.2%; *P* = 7.0 × 10^−7^). Associations between short sleep duration and GMVs are presented in Fig. 2.

**Figure 3:**
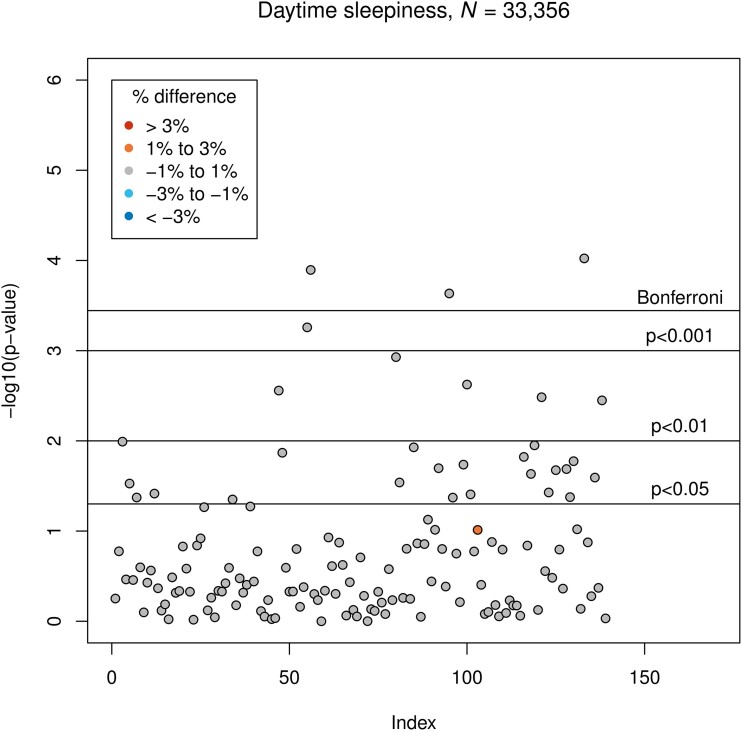
**Results of LM3 (three-sixths; adjusted linear model): associations between excessive daytime sleepiness and GMV.** The vertical axis indicates the *P*-value, and colours indicate the direction of difference (plus percentage difference). Significant associations after Bonferroni correction: IDPs 56, 95 and 133 (see Supplementary Table 3).

Excessive daytime sleepiness was associated with smaller GMV of the right paracingulate gyrus (*β* = −35.1, corresponding to −0.6% difference to the group without excessive daytime sleepiness; *P* = 1.3 × 10^−4^), the left occipital pole (*β* = −57.4, corresponding to −0.7%; *P* = 2.3 × 10^−4^) and the right cerebellar lobule VIIIb (*β* = −25.6, corresponding to −0.9%; *P* = 9.5 × 10^−5^). Associations between excessive daytime sleepiness and GMVs are presented in Fig. 3.

**Figure 4:**
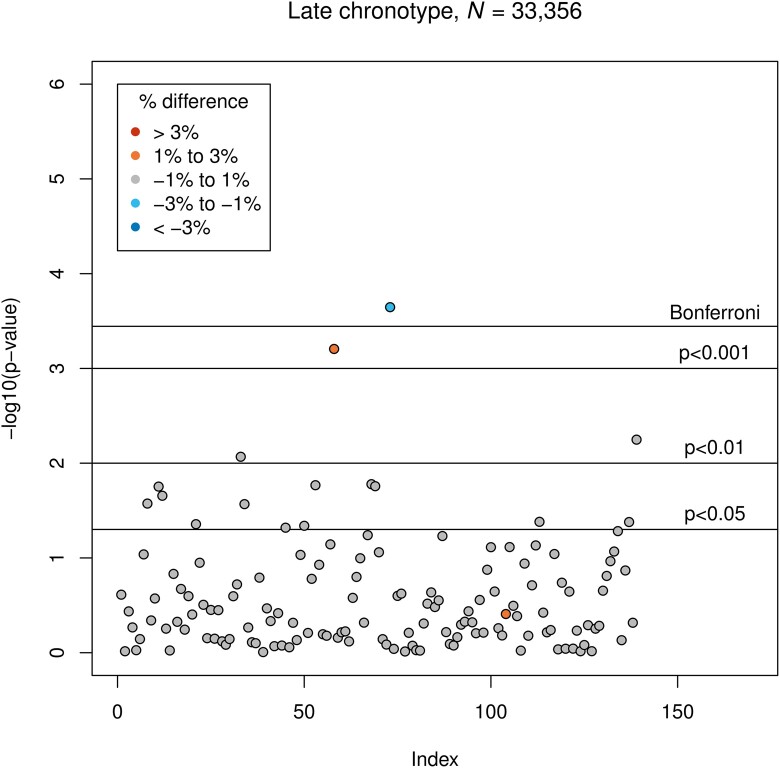
**Results of LM3 (four-sixths; adjusted linear model): associations between late chronotype and GMV.** The vertical axis indicates the *P*-value, and colours indicate the direction of difference (plus percentage difference). Significant associations after Bonferroni correction: IDP 73 (see Supplementary Table 3).

**Figure 5:**
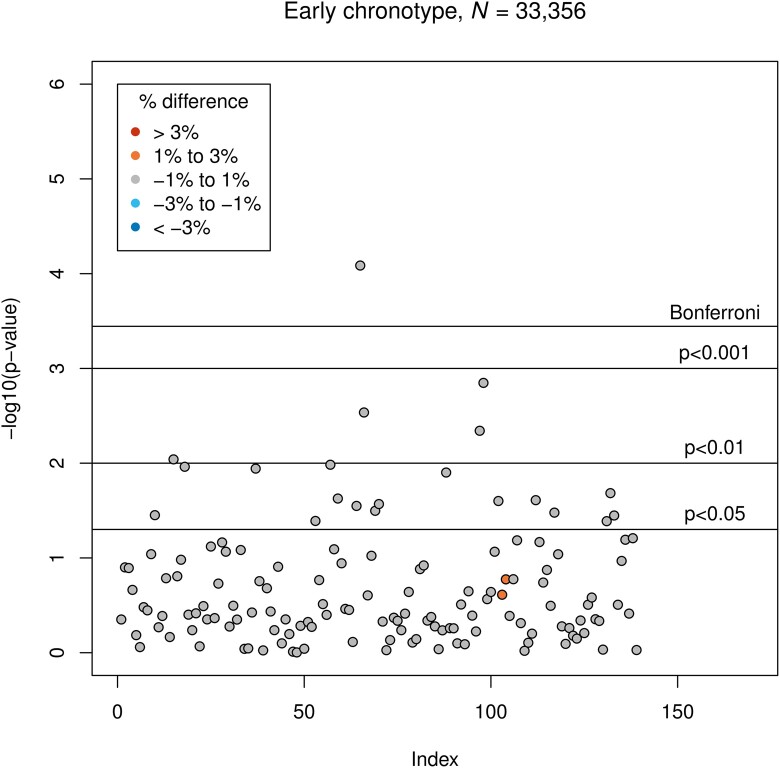
**Results of LM3 (five-sixths; adjusted linear model): associations between early chronotype and GMV.** The vertical axis indicates the *P*-value, and colours indicate the direction of difference (plus percentage difference). Significant associations after Bonferroni correction: IDP 65 (see Supplementary Table 3).

Late chronotype was associated with smaller GMV of the left temporal fusiform cortex, anterior division (*β* = −15.4, corresponding to −1.0% difference to the intermediate chronotype group; *P* = 2.3 × 10^−4^). Early chronotype was associated with smaller GMV of the left frontal orbital cortex (*β* = −31, corresponding to −0.5% difference to the intermediate chronotype group; *P* = 8.2 × 10^−5^). Associations between both chronotypes and GMVs are presented in Figs 4 and 5.

Insomnia symptoms were not associated with GMV (Fig. 6). Associations between the remaining variables and GMV are presented in Supplementary Figs 1–9. All results refer to LM3 (for the results of all applied partial *F*-tests, see Supplementary Table 6; full LM3 summaries available in the supplementary material).

**Figure 6:**
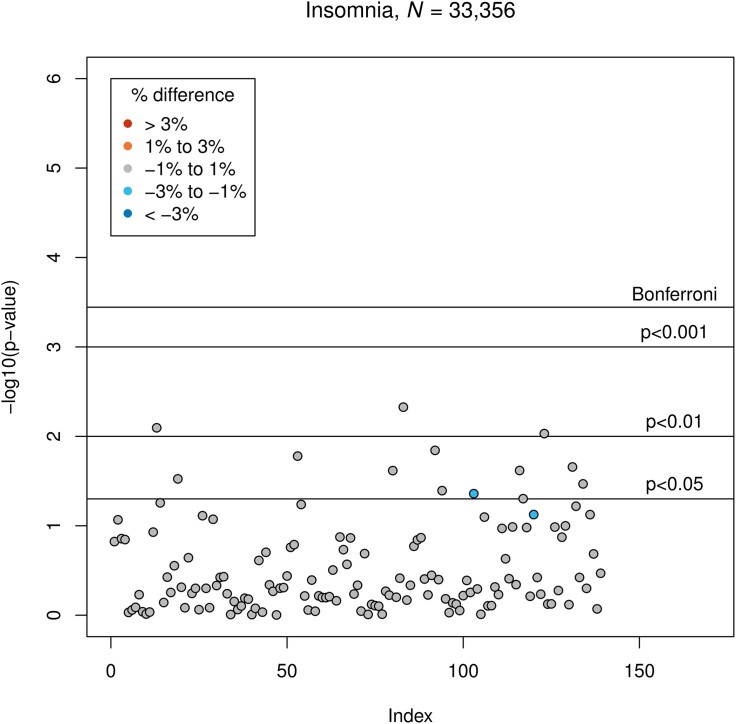
**Results of LM3 (six-sixths; adjusted linear model): associations between insomnia symptoms and GMV.** The vertical axis indicates the *P*-value, and colours indicate the direction of difference (plus percentage difference). Significant associations after Bonferroni correction: None.

Sensitivity analysis 1: Aiming for a more differentiated operationalization of sleep apnoea, we implemented an alternative system of detecting the presence or absence of sleep apnoea, which is oriented towards previous research^44^ and described in Supplementary Fig. 10 (new sample size: *n* = 28 115). Replacing the previous with the new definition of sleep apnoea in LM3, long sleep duration was associated with larger GMV of the right and left cingulate gyrus, posterior division (*β* = 147.5 and 117.8, corresponding to 2.7% and 2.3% difference to the normal sleep duration group; *P* = 7.1 × 10^−6^ and 1.8 × 10^−4^) and the right and left caudate (*β* = 147.6 and 151.2, corresponding to 4.5% and 5.0%; *P* = 1.0 × 10^−4^ and 3.7 × 10^−5^). Associations between GMV of other basal ganglia substructures (putamen and pallidum) and long sleep duration were not significant, although reaching small *P*-values (left putamen: *P* = 1.3 × 10^−2^; right pallidum: *P* = 1.0 × 10^−2^) and/or large effect sizes (right and left pallidum: *β* = 6.4 and 3.8, corresponding to 10.7% and 9.1% difference to the normal sleep duration group). Associations between long sleep duration and GMVs (sensitivity analysis) are presented in Supplementary Fig. 1^1^. The new defined sleep apnoea variable was not associated with any brain morphometry variable either. For an overview, see Supplementary Fig. 1^2^.

Sensitivity analysis 2: Aiming for an exclusion of possible collinearity effects, we examined associations between SH variables and GMVs without adding multiple SH variables to the model ‘simultaneously’. However, after implementing a separate linear model for each SH variable (sLM1-6), results did not deviate considerably from abovementioned core findings (see Supplementary Figs 13–20; full sLM summaries available in the supplementary material).

**Figure 7:**
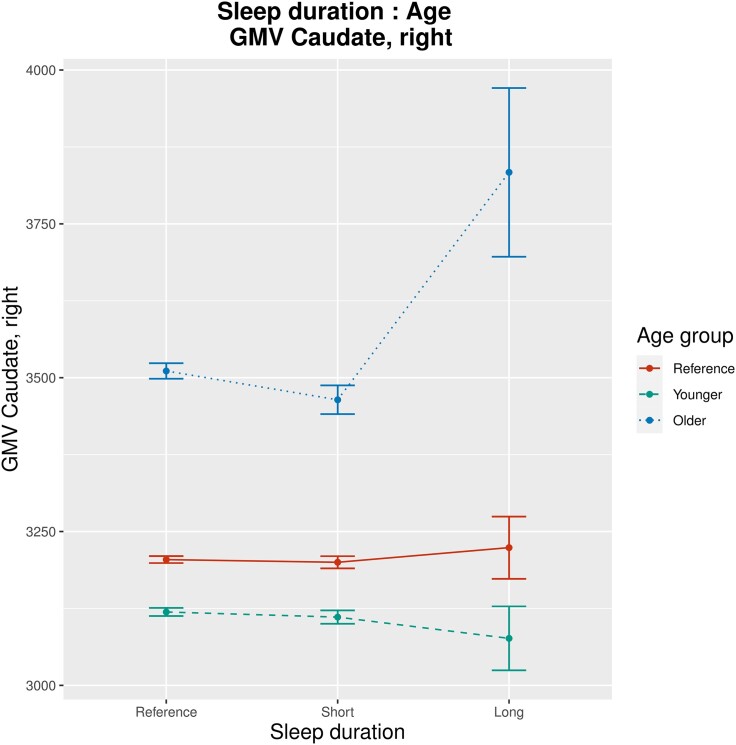
**Results of sLM7 (one-half; interaction effects between sex/age and all sleep-related variables included): group-wise mean GMV, illustrating interaction effects between sleep duration and age regarding their association with GMV of the left caudate (*β* = 18.4, *P* = 3.7 × 10^−5^).** For visualization purposes, the continuous variable age has been factorized into three categories (‘younger’, ‘reference’ and ‘older’), with a uniform division of the range between minimal and maximal age. Bars indicate group-wise standard error. Units of measurement (GMV) are mm^3^.

**Figure 8:**
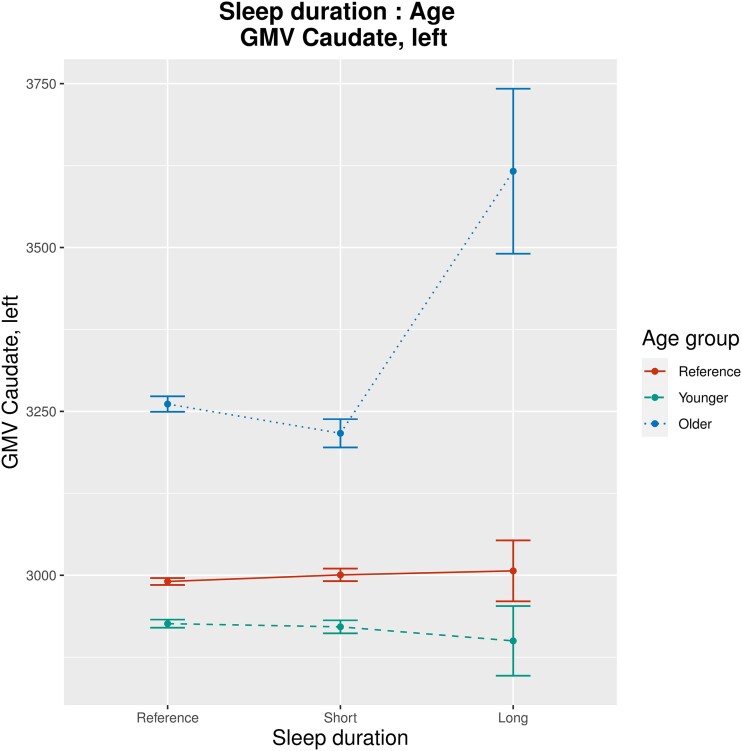
**Results of sLM7 (two-twos; interaction effects between sex/age and all sleep-related variables included): group-wise mean GMV, illustrating interaction effects between sleep duration and age regarding their association with GMV of the right caudate (*β* = 18.3, *P* = 2.0 × 10^−5^).** For visualization purposes, the continuous variable age has been factorized into three categories (‘younger’, ‘reference’ and ‘older’), with a uniform division of the range between minimal and maximal age. Bars indicate group-wise standard error. Units of measurement (GMV) are mm^3^.

Sensitivity analysis 3: Taking into account that age or sex may affect associations between SH and GMV, we implemented an exploratory linear model with interaction effects between age or sex and all sleep-related variables (sleep duration, insomnia symptoms, daytime sleepiness, chronotype, sleep medication use, sleep apnoea; sLM7): age and long sleep duration interacted significantly regarding their associations with GMV of the right and left caudate (*β* = 18.4 and 18.3; *P* = 3.7 × 10^−5^ and 2.0 × 10^−5^; Figs 7 and 8). Age and daytime sleepiness interacted significantly regarding their associations with GMV of the right and left caudate (*β* = 5.7 and 4.5; *P* = 4.8 × 10^−7^ and 4.5 × 10^−5^; see Supplementary Figs 2^1^ and 22) as well as with GMV of the right and left pallidum (*β* = 0.3 and 0.2; *P* = 2.4 × 10^−5^ and 3.4 × 10^−4^; see Supplementary Figs 2^3^ and 24). Further significant findings were interaction effects between age and daytime sleepiness regarding GMV of diverse cerebellar lobuli as well as an interaction effect between age and sleep medication regarding GMV of the left middle frontal gyrus (see full sLM7 summaries in the supplementary material). There were no significant interaction effects between sex and any sleep-related variable regarding their association with GMV.

## Discussion

The current results suggest that long sleep duration is associated with larger GMV of the right cingulate gyrus (posterior division), the left and right caudate and the right pallidum. Short sleep duration was found to be associated with smaller GMV of the right middle temporal gyrus (anterior division) and the left lateral occipital cortex (inferior division) as well as with larger GMV of the cerebellar vermis lobule X. Furthermore, the current analyses suggest that excessive daytime sleepiness is associated with smaller GMV of the right paracingulate gyrus, the left occipital pole and the right cerebellar lobule VIIIb. Late chronotype was found to be associated with smaller GMV of the left temporal fusiform cortex (anterior division), while early chronotype was associated with smaller GMV of the left frontal orbital cortex. The current results do not suggest any association between insomnia symptoms, sleep medication, or sleep apnoea and GMV.

### Insomnia symptoms

It must be considered a particularly striking result that insomnia symptoms were not related to any alteration in brain morphometry. Also, most of the brain areas that have been previously connected to insomnia or poor sleep (e.g. frontal lobe substructures and the hippocampus) did not show alterations for any of the SH variables tested in the current analysis. This might indicate that inconsistent findings of previous studies have come about due to the absence of clear-cut associations in combination with insufficient sample sizes and, hence, statistical power. Notably, the main difference compared to these studies is that the current investigation relies only on one self-report item for assessing nocturnal insomnia symptoms while previous investigations have examined well-characterized groups of patients with insomnia disorder. However, considering the large sample size of the UKBB cohort, statistical power to detect small effect sizes could still be guaranteed in severely contaminated group comparisons (e.g. healthy good sleepers falsely categorized as patients with insomnia).

Thus, it is unlikely that the definition of insomnia in the current study explains the discrepant results compared to previous studies. It might consequently be assumed that insomnia symptoms are reflected in other neurobiological parameters than mere GMV. A more differentiated approach could be, for example, shape analysis methods as used by several recent studies.^45,46^ Also, the current results do not contradict functional imaging findings suggesting associations between insomnia symptoms and aberrant brain activation or connectivity (e.g. default mode network hyperconnecitvity^47,48^).

### Long sleep duration and basal ganglia

Regarding the magnitude of significant GMV differences the findings for sleep duration stand out. In particular, the results indicate an association between long sleep duration and an extensive enlargement of basal ganglia substructures. This association is quite clear-cut as GMV differences for long sleep duration were the highest for the exact 6 out of 139 IDPs representing basal ganglia (left and right caudate, pallidum and putamen). It is however challenging to interpret this outcome since literature about larger GMV of basal ganglia is scarce. One approach could be based upon a study by Kumar *et al.*^49^: their findings suggest an association between sleep apnoea syndrome and larger basal ganglia volume at an early stage of the disorder. Since there is good reason to assume that (early) sleep apnoea syndrome might often have been undetected in the current sample (assessment via self-report and no systematic screening), and since individuals with sleep apnoea syndrome often display a prolonged sleep duration,^50^ this interpretation seems likely. However, it remains unclear why excessive daytime sleepiness, another indicator for sleep apnoea syndrome,^51^ was not associated with GMV of basal ganglia. Additionally, the absence of an association between the new defined sleep apnoea variable and GMV might suggest that a fully developed sleep apnoea syndrome does not (or no longer) go along with larger basal ganglia substructures. Nevertheless, considering that our sensitivity analysis (sLM7) yielded greater associations between long sleep duration as well as excessive daytime sleepiness and larger GMV of basal ganglia in older persons, the initial interpretation might still be valid for a subpopulation of higher age. While previous research did not find associations between sleep apnoea and larger GMV of basal ganglia, markers of sleep apnoea (hypoxaemia, respiratory disturbances and sleep fragmentation) have been shown to be associated with larger GMV of multiple cortical and subcortical brain regions.^25^ These findings are assumed to represent adaptive brain mechanisms compensating for deficits related to early stage sleep apnoea. Considering this, our results might indicate a compensation effect for impairments induced by sleep apnoea-related long sleep duration or excessive daytime sleepiness in older persons. In the context of sleep-disordered breathing, these impairments might be represented by a greater amyloid burden, as suggested in a study on middle-aged and older adults.^26^ Conclusively, the current finding can only be a starting point for future studies on this topic.

## Further findings

Long sleep duration was also found to be related to larger GMV of the right cingulate gyrus (posterior division). The posterior cingulate gyrus has previously been shown to play a key role in the default mode network (DMN),^52^ a resting state connectivity pattern that is assumed to be altered or unbalanced in insomnia as well as in obstructive sleep apnoea.^53,54^ It is problematic to integrate morphometry findings into (directed) connectivity theories since morphometric alterations cannot be translated into aberrant functionality without further consideration. However, speculatively, the current findings might underline the association between sleep apnoea and DMN-related cognitive symptoms like extensive self-referential processing (e.g. rumination).

The findings regarding short sleep duration, excessive daytime sleepiness and chronotype indicate relatively small, non-systematic GMV alterations (mostly under 1.0% difference). Interpretive approaches based on previous research are presented in the supplementary material (see ‘Further findings’ section).

## Conclusions

Although we reported significant associations between neurocognitive function and long sleep duration, sleep medication use and the two extreme chronotypes,^11^ these findings do not translate into GMV findings. This discrepancy may tell us that neurocognitive impairment does not necessarily imply GMV alterations. However, it should also be noted that the sample size of the current study is considerably smaller than the one of our previous investigation. Still, our findings contradict the idea of considering alterations in GMV as sufficient biomarker for SH-related impairment of neurocognitive function.

### Limitations and outlook

One limitation of this study is that the UK Biobank sample is relatively healthy^55^ resulting in a low number of individuals suffering from sleep apnoea syndrome and a low number of cases with sleep or psychiatric medication. However, in comparison with previous reports on the association between SH and brain morphometry, the larger sample size of this study may compensate for this limitation. As described above, this form of compensation also applies for possible weaknesses in operationalization. Nevertheless, it must be mentioned that statistical power differed considerably between comparisons involving characteristics of low prevalence (e.g. sleep apnoea syndrome or long sleep duration) and comparisons involving characteristics of moderate or high prevalence only. Furthermore, it must be assumed that sample characteristics have been influenced by selection processes from recruitment to imaging visit. Persons participating in the UK Biobank project are most likely not unrestrictedly representative for the (older) population of the UK but, among others, tend to be healthier. Potential biases arising from this circumstance must be kept in mind when interpreting the current results.^56^

For future research, it might be of particular interest to experimentally examine if patients with obstructive sleep apnoea display morphometric alterations of basal ganglia substructures. At this, it seems to be crucial to differentiate between early and late stages of the disorder. Finding reliable associations between distinct stages of obstructive sleep apnoea and specific morphometric alterations (=biomarkers) might serve as a helpful tool in diagnostics and prevention. Furthermore, the current results indicate that neurobiological correlates of insomnia cannot be detected by only considering GMVs. Much rather, future research should focus on either functional neuroimaging (e.g. connectivity analyses) or more differentiated morphometric data (e.g. shape analyses).

## Supplementary Material

fcad200_Supplementary_Data

## Data Availability

The data used in this study are available via the UKBB access management system (https://bbams.ndph.ox.ac.uk/ams/). Information regarding registration and application for access is available at https://www.ukbiobank.ac.uk/enable-your-research.

## Supplementary material

Supplementary Tables 1–6, Supplementary Figs 1–24 and secondary discussion points (see ‘Further findings’ section) are available at *Brain Communications* online.

Full LM3 (original and alternative operationalization of sleep apnoea), sLM1-6 and sLM7 summaries are available in the Supplementary material at *Brain Communications* online.
