# Supplementary material for: Associations between sleep health and grey matter volume in the UK Biobank cohort (n = 33 356)
Source: Brain Commun. 2023 Jul 12;5(4):fcad200. doi: 10.1093/braincomms/fcad200 (PMC10365832; doi:10.1093/braincomms/fcad200)
Supplement: fcad200_Supplementary_Data [file fcad200_supplementary_data.zip › Supplementary_tables_and_figures.docx]

**Supplementary tables and figures**

Supplementary Table 1

*List of Neurological Conditions*

| Data Field | Code(s) | Description |
| --- | --- | --- |
| 6150 | 3 | Stroke |
| 20001 | 1031  1032 | Meningeal cancer / Malignant meningioma  Brain cancer / Primary malignant brain tumour |
| 20002 | 1491  1245  1425  1433  1258  1263  1246  1264  1266  1244  1583  1659  1247  1259  1261  1240  1683  1397  1434  1262  1524  1086  1083  1082 | Brain haemorrhage  Brain abscess / Intracranial abscess  Cerebral aneurysm  Cerebral palsy  Chronic / Degenerative neurological problem  Dementia / Alzheimer’s disease / Cognitive impairment  Encephalitis  Epilepsy  Head injury  Infection of nervous system  Ischaemic stroke  Meningioma / Benign meningeal tumour  Meningitis  Motor neurone disease  Multiple Sclerosis  Neurological injury / trauma  Benign Neuroma  Other demyelinating disease (not multiple sclerosis)  Other neurological problem  Parkinson’s disease  Spina bifida  Subarachnoid haemorrhage  Subdural haemorrhage / Haematoma  Transient ischaemic attack (TIA) |

Supplementary Table 2

*List of Sedatives and Hypnotics (Sleep Medication)*

| Data Field | Codes | Description (substance / trade name) |
| --- | --- | --- |
| 20003 | 1140863152  1141157496  1140863244  1140863250  1140855856  1140863202  1140863210  1140863138  1140863144  1140928004  1141171404  1141171410  1140865016  1140864916  1140863182  1140863194  1140855896  1140863196  1140855900  1140855898  1140855902  1140855904  1140863104  1140863106  1140855914  1140855920 | Diazepam  Diazepam product  Valium 2 mg tablet  Valium 2 mg / 5 ml syrup  Valium 10 mg suppository  Temazepam  Normison 10 mg capsule  Euhypnos 10 mg / 5 ml oral solution  Zopiclone  Zimovane ls 3.75 mg tablet  Zaleplon  Sonata 5 mg capsule  Zolpidem  Stilnoct 5 mg tablet  Nitrazepam  Mogadon 5 mg tablet  Nitrados 5 mg tablet  Remnos 5 mg tablet  Somnite 5 mg tablet  Noctesed 5 mg tablet  Surem 5 mg capsule  Unisomnia 5 mg tablet  Flunitrazepam  Rohypnol 1 mg tablet  Triazolam  Halcion 125 micrograms tablet |

Supplementary Table 3

*List of 139 IDPs*

| Data Field | Number | Description |
| --- | --- | --- |
| \| 25782 \|  \| \| --- \| --- \| \|  \| \| 25783 \|  \| \|  \| \| 25784 \|  \| \|  \| \| 25785 \|  \| \|  \| \| 25786 \|  \| \|  \| \| 25787 \|  \| \|  \| \| 25788 \|  \| \|  \| \| 25789 \|  \| \|  \| \| 25790 \|  \| \|  \| \| 25791 \|  \| \|  \| \| 25792 \|  \| \|  \| \| 25793 \|  \| \|  \| \| 25794 \|  \| \|  \| \| 25795 \|  \| \|  \| \| 25796 \|  \| \|  \| \| 25797 \|  \| \|  \| \| 25798 \|  \| \|  \| \| 25799 \|  \| \|  \| \| 25800 \|  \| \|  \| \| 25801 \|  \| \|  \| \| 25802 \|  \| \|  \| \| 25803 \|  \| \|  \| \| 25804 \|  \| \|  \| \| 25805 \|  \| \|  \| \| 25806 \|  \| \|  \| \| 25807 \|  \| \|  \| \| 25808 \|  \| \|  \| \| 25809 \|  \| \|  \| \| 25810 \|  \| \|  \| \| 25811 \|  \| \|  \| \| 25812 \|  \| \|  \| \| 25813 \|  \| \|  \| \| 25814 \|  \| \|  \| \| 25815 \|  \| \|  \| \| 25816 \|  \| \|  \| \| 25817 \|  \| \|  \| \| 25818 \|  \| \|  \| \| 25819 \|  \| \|  \| \| 25820 \|  \| \|  \| \| 25821 \|  \| \|  \| \| 25822 \|  \| \|  \| \| 25823 \|  \| \|  \| \| 25824 \|  \| \|  \| \| 25825 \|  \| \|  \| \| 25826 \|  \| \|  \| \| 25827 \|  \| \|  \| \| 25828 \|  \| \|  \| \| 25829 \|  \| \|  \| \| 25830 \|  \| \|  \| \| 25831 \|  \| \|  \| \| 25832 \|  \| \|  \| \| 25833 \|  \| \|  \| \| 25834 \|  \| \|  \| \| 25835 \|  \| \|  \| \| 25836 \|  \| \|  \| \| 25837 \|  \| \|  \| \| 25838 \|  \| \|  \| \| 25839 \|  \| \|  \| \| 25840 \|  \| \|  \| \| 25841 \|  \| \|  \| \| 25842 \|  \| \|  \| \| 25843 \|  \| \|  \| \| 25844 \|  \| \|  \| \| 25845 \|  \| \|  \| \| 25846 \|  \| \|  \| \| 25847 \|  \| \|  \| \| 25848 \|  \| \|  \| \| 25849 \|  \| \|  \| \| 25850 \|  \| \|  \| \| 25851 \|  \| \|  \| \| 25852 \|  \| \|  \| \| 25853 \|  \| \|  \| \| 25854 \|  \| \|  \| \| 25855 \|  \| \|  \| \| 25856 \|  \| \|  \| \| 25857 \|  \| \|  \| \| 25858 \|  \| \|  \| \| 25859 \|  \| \|  \| \| 25860 \|  \| \|  \| \| 25861 \|  \| \|  \| \| 25862 \|  \| \|  \| \| 25863 \|  \| \|  \| \| 25864 \|  \| \|  \| \| 25865 \|  \| \|  \| \| 25866 \|  \| \|  \| \| 25867 \|  \| \|  \| \| 25868 \|  \| \|  \| \| 25869 \|  \| \|  \| \| 25870 \|  \| \|  \| \| 25871 \|  \| \|  \| \| 25872 \|  \| \|  \| \| 25873 \|  \| \|  \| \| 25874 \|  \| \|  \| \| 25875 \|  \| \|  \| \| 25876 \|  \| \|  \| \| 25877 \|  \| \|  \| \| 25878 \|  \| \|  \| \| 25879 \|  \| \|  \| \| 25880 \|  \| \|  \| \| 25881 \|  \| \|  \| \| 25882 \|  \| \|  \| \| 25883 \|  \| \|  \| \| 25884 \|  \| \|  \| \| 25885 \|  \| \|  \| \| 25886 \|  \| \|  \| \| 25887 \|  \| \|  \| \| 25888 \|  \| \|  \| \| 25889 \|  \| \|  \| \| 25890 \|  \| \|  \| \| 25891 \|  \| \|  \| \| 25892 \|  \| \|  \| \| 25893 \|  \| \|  \| \| 25894 \|  \| \|  \| \| 25895 \|  \| \|  \| \| 25896 \|  \| \|  \| \| 25897 \|  \| \|  \| \| 25898 \|  \| \|  \| \| 25899 \|  \| \|  \| \| 25900 \|  \| \|  \| \| 25901 \|  \| \|  \| \| 25902 \|  \| \|  \| \| 25903 \|  \| \|  \| \| 25904 \|  \| \|  \| \| 25905 \|  \| \|  \| \| 25906 \|  \| \|  \| \| 25907 \|  \| \|  \| \| 25908 \|  \| \|  \| \| 25909 \|  \| \|  \| \| 25910 \|  \| \|  \| \| 25911 \|  \| \|  \| \| 25912 \|  \| \|  \| \| 25913 \|  \| \|  \| \| 25914 \|  \| \|  \| \| 25915 \|  \| \|  \| \| 25916 \|  \| \|  \| \| 25917 \|  \| \|  \| \| 25918 \|  \| \|  \| \| 25919 \|  \| \|  \| \| 25920 \|  \| \|  \| | \| 1 \|  \| \| --- \| --- \| \|  \| \| 2 \|  \| \|  \| \| 3 \|  \| \|  \| \| 4 \|  \| \|  \| \| 5 \|  \| \|  \| \| 6 \|  \| \|  \| \| 7 \|  \| \|  \| \| 8 \|  \| \|  \| \| 9 \|  \| \|  \| \| 10 \|  \| \|  \| \| 11 \|  \| \|  \| \| 12 \|  \| \|  \| \| 13 \|  \| \|  \| \| 14 \|  \| \|  \| \| 15 \|  \| \|  \| \| 16 \|  \| \|  \| \| 17 \|  \| \|  \| \| 18 \|  \| \|  \| \| 19 \|  \| \|  \| \| 20 \|  \| \|  \| \| 21 \|  \| \|  \| \| 22 \|  \| \|  \| \| 23 \|  \| \|  \| \| 24 \|  \| \|  \| \| 25 \|  \| \|  \| \| 26 \|  \| \|  \| \| 27 \|  \| \|  \| \| 28 \|  \| \|  \| \| 29 \|  \| \|  \| \| 30 \|  \| \|  \| \| 31 \|  \| \|  \| \| 32 \|  \| \|  \| \| 33 \|  \| \|  \| \| 34 \|  \| \|  \| \| 35 \|  \| \|  \| \| 36 \|  \| \|  \| \| 37 \|  \| \|  \| \| 38 \|  \| \|  \| \| 39 \|  \| \|  \| \| 40 \|  \| \|  \| \| 41 \|  \| \|  \| \| 42 \|  \| \|  \| \| 43 \|  \| \|  \| \| 44 \|  \| \|  \| \| 45 \|  \| \|  \| \| 46 \|  \| \|  \| \| 47 \|  \| \|  \| \| 48 \|  \| \|  \| \| 49 \|  \| \|  \| \| 50 \|  \| \|  \| \| 51 \|  \| \|  \| \| 52 \|  \| \|  \| \| 53 \|  \| \|  \| \| 54 \|  \| \|  \| \| 55 \|  \| \|  \| \| 56 \|  \| \|  \| \| 57 \|  \| \|  \| \| 58 \|  \| \|  \| \| 59 \|  \| \|  \| \| 60 \|  \| \|  \| \| 61 \|  \| \|  \| \| 62 \|  \| \|  \| \| 63 \|  \| \|  \| \| 64 \|  \| \|  \| \| 65 \|  \| \|  \| \| 66 \|  \| \|  \| \| 67 \|  \| \|  \| \| 68 \|  \| \|  \| \| 69 \|  \| \|  \| \| 70 \|  \| \|  \| \| 71 \|  \| \|  \| \| 72 \|  \| \|  \| \| 73 \|  \| \|  \| \| 74 \|  \| \|  \| \| 75 \|  \| \|  \| \| 76 \|  \| \|  \| \| 77 \|  \| \|  \| \| 78 \|  \| \|  \| \| 79 \|  \| \|  \| \| 80 \|  \| \|  \| \| 81 \|  \| \|  \| \| 82 \|  \| \|  \| \| 83 \|  \| \|  \| \| 84 \|  \| \|  \| \| 85 \|  \| \|  \| \| 86 \|  \| \|  \| \| 87 \|  \| \|  \| \| 88 \|  \| \|  \| \| 89 \|  \| \|  \| \| 90 \|  \| \|  \| \| 91 \|  \| \|  \| \| 92 \|  \| \|  \| \| 93 \|  \| \|  \| \| 94 \|  \| \|  \| \| 95 \|  \| \|  \| \| 96 \|  \| \|  \| \| 97 \|  \| \|  \| \| 98 \|  \| \|  \| \| 99 \|  \| \|  \| \| 100 \|  \| \|  \| \| 101 \|  \| \|  \| \| 102 \|  \| \|  \| \| 103 \|  \| \|  \| \| 104 \|  \| \|  \| \| 105 \|  \| \|  \| \| 106 \|  \| \|  \| \| 107 \|  \| \|  \| \| 108 \|  \| \|  \| \| 109 \|  \| \|  \| \| 110 \|  \| \|  \| \| 111 \|  \| \|  \| \| 112 \|  \| \|  \| \| 113 \|  \| \|  \| \| 114 \|  \| \|  \| \| 115 \|  \| \|  \| \| 116 \|  \| \|  \| \| 117 \|  \| \|  \| \| 118 \|  \| \|  \| \| 119 \|  \| \|  \| \| 120 \|  \| \|  \| \| 121 \|  \| \|  \| \| 122 \|  \| \|  \| \| 123 \|  \| \|  \| \| 124 \|  \| \|  \| \| 125 \|  \| \|  \| \| 126 \|  \| \|  \| \| 127 \|  \| \|  \| \| 128 \|  \| \|  \| \| 129 \|  \| \|  \| \| 130 \|  \| \|  \| \| 131 \|  \| \|  \| \| 132 \|  \| \|  \| \| 133 \|  \| \|  \| \| 134 \|  \| \|  \| \| 135 \|  \| \|  \| \| 136 \|  \| \|  \| \| 137 \|  \| \|  \| \| 138 \|  \| \|  \| \| 139 \|  \| \|  \| | \| Volume of grey matter in Frontal Pole (left) \|  \| \| --- \| --- \| \|  \| \| Volume of grey matter in Frontal Pole (right) \|  \| \|  \| \| Volume of grey matter in Insular Cortex (left) \|  \| \|  \| \| Volume of grey matter in Insular Cortex (right) \|  \| \|  \| \| Volume of grey matter in Superior Frontal Gyrus (left) \|  \| \|  \| \| Volume of grey matter in Superior Frontal Gyrus (right) \|  \| \|  \| \| Volume of grey matter in Middle Frontal Gyrus (left) \|  \| \|  \| \| Volume of grey matter in Middle Frontal Gyrus (right) \|  \| \|  \| \| Volume of grey matter in Inferior Frontal Gyrus, pars triangularis (left) \|  \| \|  \| \| Volume of grey matter in Inferior Frontal Gyrus, pars triangularis (right) \|  \| \|  \| \| Volume of grey matter in Inferior Frontal Gyrus, pars opercularis (left) \|  \| \|  \| \| Volume of grey matter in Inferior Frontal Gyrus, pars opercularis (right) \|  \| \|  \| \| Volume of grey matter in Precentral Gyrus (left) \|  \| \|  \| \| Volume of grey matter in Precentral Gyrus (right) \|  \| \|  \| \| Volume of grey matter in Temporal Pole (left) \|  \| \|  \| \| Volume of grey matter in Temporal Pole (right) \|  \| \|  \| \| Volume of grey matter in Superior Temporal Gyrus, anterior division (left) \|  \| \|  \| \| Volume of grey matter in Superior Temporal Gyrus, anterior division (right) \|  \| \|  \| \| Volume of grey matter in Superior Temporal Gyrus, posterior division (left) \|  \| \|  \| \| Volume of grey matter in Superior Temporal Gyrus, posterior division (right) \|  \| \|  \| \| Volume of grey matter in Middle Temporal Gyrus, anterior division (left) \|  \| \|  \| \| Volume of grey matter in Middle Temporal Gyrus, anterior division (right) \|  \| \|  \| \| Volume of grey matter in Middle Temporal Gyrus, posterior division (left) \|  \| \|  \| \| Volume of grey matter in Middle Temporal Gyrus, posterior division (right) \|  \| \|  \| \| Volume of grey matter in Middle Temporal Gyrus, temporooccipital part (left) \|  \| \|  \| \| Volume of grey matter in Middle Temporal Gyrus, temporooccipital part (right) \|  \| \|  \| \| Volume of grey matter in Inferior Temporal Gyrus, anterior division (left) \|  \| \|  \| \| Volume of grey matter in Inferior Temporal Gyrus, anterior division (right) \|  \| \|  \| \| Volume of grey matter in Inferior Temporal Gyrus, posterior division (left) \|  \| \|  \| \| Volume of grey matter in Inferior Temporal Gyrus, posterior division (right) \|  \| \|  \| \| Volume of grey matter in Inferior Temporal Gyrus, temporooccipital part (left) \|  \| \|  \| \| Volume of grey matter in Inferior Temporal Gyrus, temporooccipital part (right) \|  \| \|  \| \| Volume of grey matter in Postcentral Gyrus (left) \|  \| \|  \| \| Volume of grey matter in Postcentral Gyrus (right) \|  \| \|  \| \| Volume of grey matter in Superior Parietal Lobule (left) \|  \| \|  \| \| Volume of grey matter in Superior Parietal Lobule (right) \|  \| \|  \| \| Volume of grey matter in Supramarginal Gyrus, anterior division (left) \|  \| \|  \| \| Volume of grey matter in Supramarginal Gyrus, anterior division (right) \|  \| \|  \| \| Volume of grey matter in Supramarginal Gyrus, posterior division (left) \|  \| \|  \| \| Volume of grey matter in Supramarginal Gyrus, posterior division (right) \|  \| \|  \| \| Volume of grey matter in Angular Gyrus (left) \|  \| \|  \| \| Volume of grey matter in Angular Gyrus (right) \|  \| \|  \| \| Volume of grey matter in Lateral Occipital Cortex, superior division (left) \|  \| \|  \| \| Volume of grey matter in Lateral Occipital Cortex, superior division (right) \|  \| \|  \| \| Volume of grey matter in Lateral Occipital Cortex, inferior division (left) \|  \| \|  \| \| Volume of grey matter in Lateral Occipital Cortex, inferior division (right) \|  \| \|  \| \| Volume of grey matter in Intracalcarine Cortex (left) \|  \| \|  \| \| Volume of grey matter in Intracalcarine Cortex (right) \|  \| \|  \| \| Volume of grey matter in Frontal Medial Cortex (left) \|  \| \|  \| \| Volume of grey matter in Frontal Medial Cortex (right) \|  \| \|  \| \| Volume of grey matter in Juxtapositional Lobule Cortex (formerly Supplementary Motor Cortex) (left) \|  \| \|  \| \| Volume of grey matter in Juxtapositional Lobule Cortex (formerly Supplementary Motor Cortex) (right) \|  \| \|  \| \| Volume of grey matter in Subcallosal Cortex (left) \|  \| \|  \| \| Volume of grey matter in Subcallosal Cortex (right) \|  \| \|  \| \| Volume of grey matter in Paracingulate Gyrus (left) \|  \| \|  \| \| Volume of grey matter in Paracingulate Gyrus (right) \|  \| \|  \| \| Volume of grey matter in Cingulate Gyrus, anterior division (left) \|  \| \|  \| \| Volume of grey matter in Cingulate Gyrus, anterior division (right) \|  \| \|  \| \| Volume of grey matter in Cingulate Gyrus, posterior division (left) \|  \| \|  \| \| Volume of grey matter in Cingulate Gyrus, posterior division (right) \|  \| \|  \| \| Volume of grey matter in Precuneous Cortex (left) \|  \| \|  \| \| Volume of grey matter in Precuneous Cortex (right) \|  \| \|  \| \| Volume of grey matter in Cuneal Cortex (left) \|  \| \|  \| \| Volume of grey matter in Cuneal Cortex (right) \|  \| \|  \| \| Volume of grey matter in Frontal Orbital Cortex (left) \|  \| \|  \| \| Volume of grey matter in Frontal Orbital Cortex (right) \|  \| \|  \| \| Volume of grey matter in Parahippocampal Gyrus, anterior division (left) \|  \| \|  \| \| Volume of grey matter in Parahippocampal Gyrus, anterior division (right) \|  \| \|  \| \| Volume of grey matter in Parahippocampal Gyrus, posterior division (left) \|  \| \|  \| \| Volume of grey matter in Parahippocampal Gyrus, posterior division (right) \|  \| \|  \| \| Volume of grey matter in Lingual Gyrus (left) \|  \| \|  \| \| Volume of grey matter in Lingual Gyrus (right) \|  \| \|  \| \| Volume of grey matter in Temporal Fusiform Cortex, anterior division (left) \|  \| \|  \| \| Volume of grey matter in Temporal Fusiform Cortex, anterior division (right) \|  \| \|  \| \| Volume of grey matter in Temporal Fusiform Cortex, posterior division (left) \|  \| \|  \| \| Volume of grey matter in Temporal Fusiform Cortex, posterior division (right) \|  \| \|  \| \| Volume of grey matter in Temporal Occipital Fusiform Cortex (left) \|  \| \|  \| \| Volume of grey matter in Temporal Occipital Fusiform Cortex (right) \|  \| \|  \| \| Volume of grey matter in Occipital Fusiform Gyrus (left) \|  \| \|  \| \| Volume of grey matter in Occipital Fusiform Gyrus (right) \|  \| \|  \| \| Volume of grey matter in Frontal Operculum Cortex (left) \|  \| \|  \| \| Volume of grey matter in Frontal Operculum Cortex (right) \|  \| \|  \| \| Volume of grey matter in Central Opercular Cortex (left) \|  \| \|  \| \| Volume of grey matter in Central Opercular Cortex (right) \|  \| \|  \| \| Volume of grey matter in Parietal Operculum Cortex (left) \|  \| \|  \| \| Volume of grey matter in Parietal Operculum Cortex (right) \|  \| \|  \| \| Volume of grey matter in Planum Polare (left) \|  \| \|  \| \| Volume of grey matter in Planum Polare (right) \|  \| \|  \| \| Volume of grey matter in Heschl's Gyrus (includes H1 and H2) (left) \|  \| \|  \| \| Volume of grey matter in Heschl's Gyrus (includes H1 and H2) (right) \|  \| \|  \| \| Volume of grey matter in Planum Temporale (left) \|  \| \|  \| \| Volume of grey matter in Planum Temporale (right) \|  \| \|  \| \| Volume of grey matter in Supracalcarine Cortex (left) \|  \| \|  \| \| Volume of grey matter in Supracalcarine Cortex (right) \|  \| \|  \| \| Volume of grey matter in Occipital Pole (left) \|  \| \|  \| \| Volume of grey matter in Occipital Pole (right) \|  \| \|  \| \| Volume of grey matter in Thalamus (left) \|  \| \|  \| \| Volume of grey matter in Thalamus (right) \|  \| \|  \| \| Volume of grey matter in Caudate (left) \|  \| \|  \| \| Volume of grey matter in Caudate (right) \|  \| \|  \| \| Volume of grey matter in Putamen (left) \|  \| \|  \| \| Volume of grey matter in Putamen (right) \|  \| \|  \| \| Volume of grey matter in Pallidum (left) \|  \| \|  \| \| Volume of grey matter in Pallidum (right) \|  \| \|  \| \| Volume of grey matter in Hippocampus (left) \|  \| \|  \| \| Volume of grey matter in Hippocampus (right) \|  \| \|  \| \| Volume of grey matter in Amygdala (left) \|  \| \|  \| \| Volume of grey matter in Amygdala (right) \|  \| \|  \| \| Volume of grey matter in Ventral Striatum (left) \|  \| \|  \| \| Volume of grey matter in Ventral Striatum (right) \|  \| \|  \| \| Volume of grey matter in Brain-Stem \|  \| \|  \| \| Volume of grey matter in I-IV Cerebellum (left) \|  \| \|  \| \| Volume of grey matter in I-IV Cerebellum (right) \|  \| \|  \| \| Volume of grey matter in V Cerebellum (left) \|  \| \|  \| \| Volume of grey matter in V Cerebellum (right) \|  \| \|  \| \| Volume of grey matter in VI Cerebellum (left) \|  \| \|  \| \| Volume of grey matter in VI Cerebellum (vermis) \|  \| \|  \| \| Volume of grey matter in VI Cerebellum (right) \|  \| \|  \| \| Volume of grey matter in Crus I Cerebellum (left) \|  \| \|  \| \| Volume of grey matter in Crus I Cerebellum (vermis) \|  \| \|  \| \| Volume of grey matter in Crus I Cerebellum (right) \|  \| \|  \| \| Volume of grey matter in Crus II Cerebellum (left) \|  \| \|  \| \| Volume of grey matter in Crus II Cerebellum (vermis) \|  \| \|  \| \| Volume of grey matter in Crus II Cerebellum (right) \|  \| \|  \| \| Volume of grey matter in VIIb Cerebellum (left) \|  \| \|  \| \| Volume of grey matter in VIIb Cerebellum (vermis) \|  \| \|  \| \| Volume of grey matter in VIIb Cerebellum (right) \|  \| \|  \| \| Volume of grey matter in VIIIa Cerebellum (left) \|  \| \|  \| \| Volume of grey matter in VIIIa Cerebellum (vermis) \|  \| \|  \| \| Volume of grey matter in VIIIa Cerebellum (right) \|  \| \|  \| \| Volume of grey matter in VIIIb Cerebellum (left) \|  \| \|  \| \| Volume of grey matter in VIIIb Cerebellum (vermis) \|  \| \|  \| \| Volume of grey matter in VIIIb Cerebellum (right) \|  \| \|  \| \| Volume of grey matter in IX Cerebellum (left) \|  \| \|  \| \| Volume of grey matter in IX Cerebellum (vermis) \|  \| \|  \| \| Volume of grey matter in IX Cerebellum (right) \|  \| \|  \| \| Volume of grey matter in X Cerebellum (left) \|  \| \|  \| \| Volume of grey matter in X Cerebellum (vermis) \|  \| \|  \| \| Volume of grey matter in X Cerebellum (right) \|  \| \|  \| |

Supplementary Table 4 (1/2)

*List of Mood Stabilisers, Antidepressants, and Antipsychotics (Psychotropic Medication)*

| Data Field | Codes | Description (substance / trade name) |
| --- | --- | --- |
| 20003 | 1140867490  1140867504  1140867494  1140872198  1140872200  1141172838  1140872214  1140872064  2038459704  1140872072  1141167860  1141185460  1141162898  1140864452 | Lithium product  Priadel 200 mg m/r tablet  Camcolit 250 tablet  Sodium valproate  Epilim 100 mg crushable tablet  Depakote 250 mg e/c tablet  Valproic acid  Carbamazepine product  Carbamazepine  Tegretol 100 mg tablet  Teril cr 200 mg m/r tablet  Teril retard 200 mg m/r tablet  Timonil retard 200 mg m/r tablet  Epimaz 100 mg tablet |
|  | 1140867888  1140882236  1140879540  1140867876  1140921600  1141151946  1141180212  1141190158  1140867878  1140867884  1140879544  1141152732  1141152736  1141200564  1141201834  1141200570  1140916282  1140916288  1140879616  1140867658  1140867668  1140867662  1140867948  1140867934  1140867938  1140856186  1140867928  1140867850  1140910704  1140867852  1140867920  1140867922  1140879630  1140867712  1140867756  1140867758  1140879628  1140909806  1140867624  1141171824  1140879620 | Paroxetine  Seroxat 20 mg tablet  Fluoxetine  Prozac 20 mg capsule  Citalopram  Cipramil 10 mg tablet  Escitalopram  Cipralex 5 mg tablet  Sertraline  Lustral 50 mg tablet  Fluvoxamine  Mirtazapine  Zispin 30 mg tablet  Duloxetine  Cymbalta 30 mg gastro-resistant capsule  Yentreve 20 mg gastro-resistant capsule  Venlafaxine  Efexor 37.5 mg tablet  Amitriptyline  Elavil 10 mg tablet  Tryptizol 10 mg tablet  Lentizol 25 mg m/r capsule  Amitriptyline hydrochloride 10 mg + Perphenazine 2 mg tablet  Triptafen tablet  Amitriptyline 12.5 mg + Chlordiazepoxide 5 mg capsule  Limbitrol 10 mg capsule  Limbitrol 5 mg capsule  Phenelzine  MAOI / Phenelzine  Nardil 15 mg tablet  Moclobemide  Manerix 150 mg tablet  Imipramine  Tofranil 10 mg tablet  Trimipramine  Surmontil 10 mg tablet  Dothiepin  Dosulepin  Prothiaden 25 mg capsule  Thaden 25 mg capsule  Clomipramine |
|  | | |
| Supplementary Table 4 (2/2)  *List of Mood Stabilisers, Antidepressants, and Antipsychotics (Psychotropic Medication)* | | |
| Data Field | Codes | Description (substance / trade name) |
| 20003 | 1140867690  1140867726  1140882310  1141146062  1140879556  1140867806  1140867812 | Anafranil 10 mg capsule  Lofepramine  Gamanil 70 mg tablet  Lomont 70 mg / 5 ml s/f suspension  Mianserin  Bolvidon 10 mg tablet  Norval 10 mg tablet |
|  | 1140879658  1140910358  1140863416  1140867168  1140867184  1140867092  1140867398  1140882098  1140867456  1140867156  1140856004  1140909800  1140867150  1140867152  1140867952  1140882100  1140867342  1140867406  1140867414  1140867084  1140867086  1140868120  1140867244  1140879750  1140867312  1141152848  1141152860  1140867444  1141177762  1140928916  1141167976  1141195974  1141202024  1141153490  1141184742  1140867420  1140882320 | Chlorpromazine  CPZ / Chlorpromazine  Largactil 10 mg tablet  Haloperidol  Haldol 5 mg tablet  Serenace 500 micrograms capsule  Fluphenazine decanoate  Fluphenazine  Modecate 12.5 mg/0.5 ml oily injection  Moditen 1 mg tablet  Moditen enanthate 25 mg/ml injection  Flupentixol  Flupenthixol  Depixol 3 mg tablet  Fluanxol 500 micrograms tablet  Zuclopenthixol  Clopixol 2 mg tablet  Loxapine  Loxapac 10 mg capsule  Droperidol  Droleptan 10 mg tablet  Trifluoperazine  Stelazine 1 mg tablet  Thioridazine  Melleril 10 mg tablet  Quetiapine  Seroquel 25 mg tablet  Risperidone  Risperdal 0.5 mg tablet  Olanzapine  Zyprexa 2.5 mg tablet  Aripiprazole  Abilify 5 mg tablet  Amisulpride  Solian 100 mg/ml s/f oral solution  Clozapine  Clozaril 25 mg tablet |

Supplementary Table 5

*Independent Variables Included in Linear Models 1-3*

|  | Linear Model 1 | Linear Model 2 | Linear Model 3 |
| --- | --- | --- | --- |
| Sleep-related variables | Insomnia symptoms, sleep duration, excessive daytime sleepiness, chronotype | **+**  None | **+**  Sleep medication use, sleep apnoea |
| Covariates | None | **+**  Socioeconomic status, level of education, ICV, BMI, sex, age | **+**  Depressive symptoms, psychotropic medication use |

*Note.* All models were compared by means of partial *F*-tests (all results listed in Supplementary Table 6).

Supplementary Table 6

*Results of all Partial F-tests, Comparing Nested Models Regarding their Statistical Explanatory Power*

| Number of IDP | Comparison LM1 vs. LM2  Pr (> F) | Comparison LM2 vs. LM3  Pr (> F) |
| --- | --- | --- |
| 1  2  3  4  5  6  7  8  9  10  11  12  13  14  15  16  17  18  19  20  21  22  23  24  25  26  27  28  29  30  31  32  33  34  35  36  37  38  39  40  41  42  43  44  45  46  47  48  49  50  51  52  53  54  55  56  57  58  59  60  61  62  63  64  65  66  67  68  69  70  71  72  73  74  75  76  77  78  79  80  81  82  83  84  85  86  87  88  89  90  91  92  93  94  95  96  97  98  99  100  101  102  103  104  105  106  107  108  109  110  111  112  113  114  115  116  117  118  119  120  121  122  123  124  125  126  127  128  129  130  131  132  133  134  135  136  137  138  139 | < 0.001  < 0.001  < 0.001  < 0.001  < 0.001  < 0.001  < 0.001  < 0.001  < 0.001  < 0.001  < 0.001  < 0.001  < 0.001  < 0.001  < 0.001  < 0.001  < 0.001  < 0.001  < 0.001  < 0.001  < 0.001  < 0.001  < 0.001  < 0.001  < 0.001  < 0.001  < 0.001  < 0.001  < 0.001  < 0.001  < 0.001  < 0.001  < 0.001  < 0.001  < 0.001  < 0.001  < 0.001  < 0.001  < 0.001  < 0.001  < 0.001  < 0.001  < 0.001  < 0.001  < 0.001  < 0.001  < 0.001  < 0.001  < 0.001  < 0.001  < 0.001  < 0.001  < 0.001  < 0.001  < 0.001  < 0.001  < 0.001  < 0.001  < 0.001  < 0.001  < 0.001  < 0.001  < 0.001  < 0.001  < 0.001  < 0.001  < 0.001  < 0.001  < 0.001  < 0.001  < 0.001  < 0.001  < 0.001  < 0.001  < 0.001  < 0.001  < 0.001  < 0.001  < 0.001  < 0.001  < 0.001  < 0.001  < 0.001  < 0.001  < 0.001  < 0.001  < 0.001  < 0.001  < 0.001  < 0.001  < 0.001  < 0.001  < 0.001  < 0.001  < 0.001  < 0.001  < 0.001  < 0.001  < 0.001  < 0.001  < 0.001  < 0.001  < 0.001  < 0.001  < 0.001  < 0.001  < 0.001  < 0.001  < 0.001  < 0.001  < 0.001  < 0.001  < 0.001  < 0.001  < 0.001  < 0.001  < 0.001  < 0.001  < 0.001  < 0.001  < 0.001  < 0.001  < 0.001  < 0.001  < 0.001  < 0.001  < 0.001  < 0.001  < 0.001  < 0.001  < 0.001  < 0.001  < 0.001  < 0.001  < 0.001  < 0.001  < 0.001  < 0.001  < 0.001 | 0.652  0.187  0.930  0.787  0.750  0.288  0.373  0.891  0.559  0.612  0.798  0.125  0.298  0.261  0.648  0.958  0.601  0.924  0.370  0.327  0.717  0.234  0.512  0.847  0.897  0.674  0.000  0.503  0.395  0.296  0.034  0.417  0.373  0.533  0.088  0.090  0.011  0.010  0.147  0.137  0.907  0.179  0.646  0.480  0.887  0.731  0.426  0.146  0.165  0.361  0.967  0.595  0.215  0.675  0.028  0.124  0.531  0.133  0.226  0.074  0.582  0.814  0.191  0.650  0.343  0.974  0.215  0.583  0.330  0.019  0.152  0.461  0.382  0.382  0.182  0.746  0.177  0.558  0.248  0.336  0.915  0.010  0.087  0.498  0.192  0.062  0.675  0.917  0.602  0.447  0.543  0.182  0.023  0.781  0.955  0.507  0.010  0.007  0.000  0.000  0.000  0.001  0.033  0.145  0.772  0.316  0.065  0.010  0.719  0.499  0.486  0.258  0.055  0.541  0.440  0.336  0.478  0.474  0.229  0.887  0.089  0.016  0.038  0.064  0.002  0.135  0.006  0.005  0.432  0.000  0.089  0.863  0.003  0.474  0.552  0.126  0.592  0.085  0.468 |


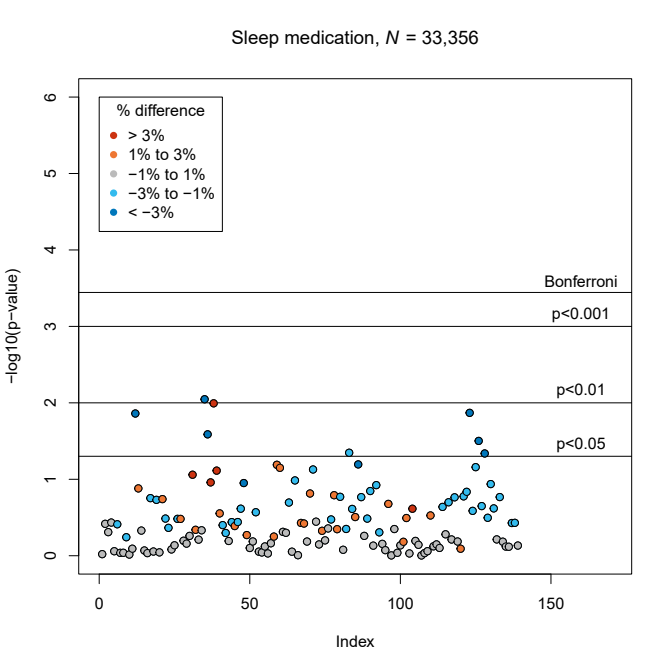


*Supplementary Fig. 1.* **Results of LM3 (1/9; adjusted linear model)**: Associations between sleep medication and GMV of all 139 IDPs as indexed in Supplementary Table 3. The vertical axis indicates the *p*-value, colours indicate the direction of difference (plus percentage difference). Significant associations after Bonferroni correction: None.


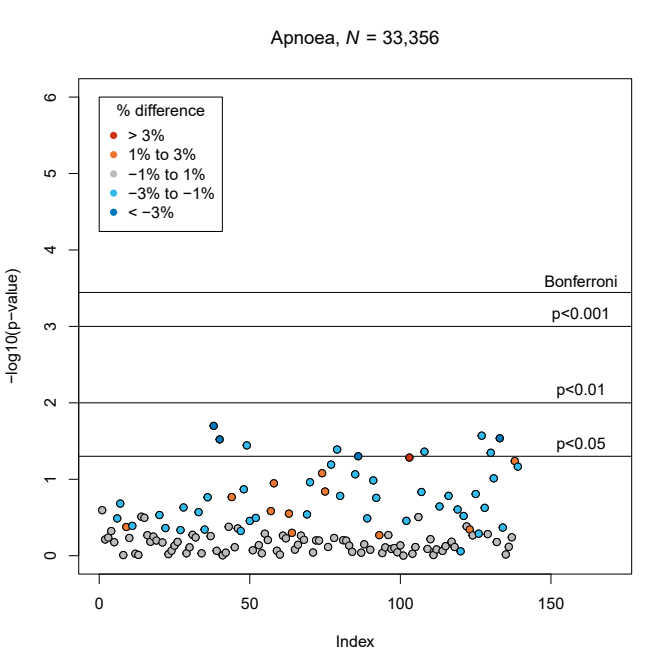


*Supplementary Fig. 2.* **Results of LM3 (2/9; adjusted linear model)**: Associations between sleep apnoea and GMV of all 139 IDPs as indexed in Supplementary Table 3. The vertical axis indicates the *p*-value, colours indicate the direction of difference (plus percentage difference). Significant associations after Bonferroni correction: None.


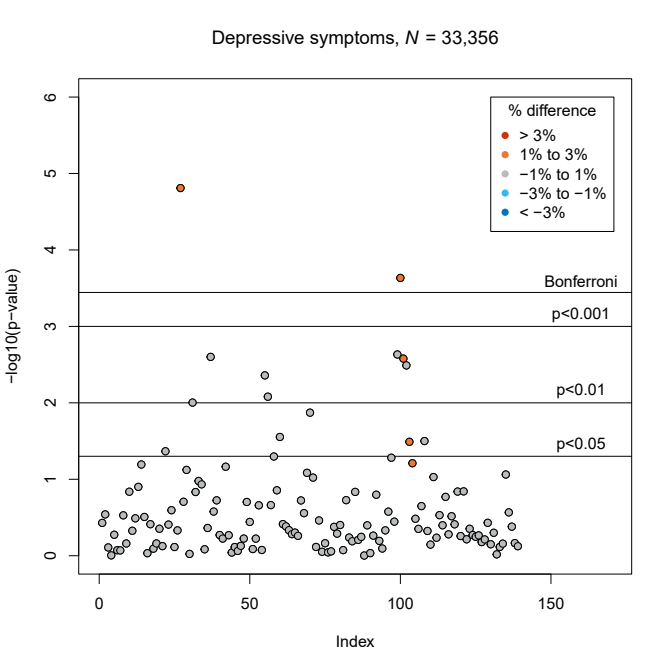


*Supplementary Fig. 3.* **Results of LM3 (3/9; adjusted linear model)**: Associations between depressive symptoms and GMV of all 139 IDPs as indexed in Supplementary Table 3. The vertical axis indicates the *p*-value, colours indicate the direction of difference (plus percentage difference). Significant associations after Bonferroni correction: IDPs 27 and 100 (see Supplementary Table 3).


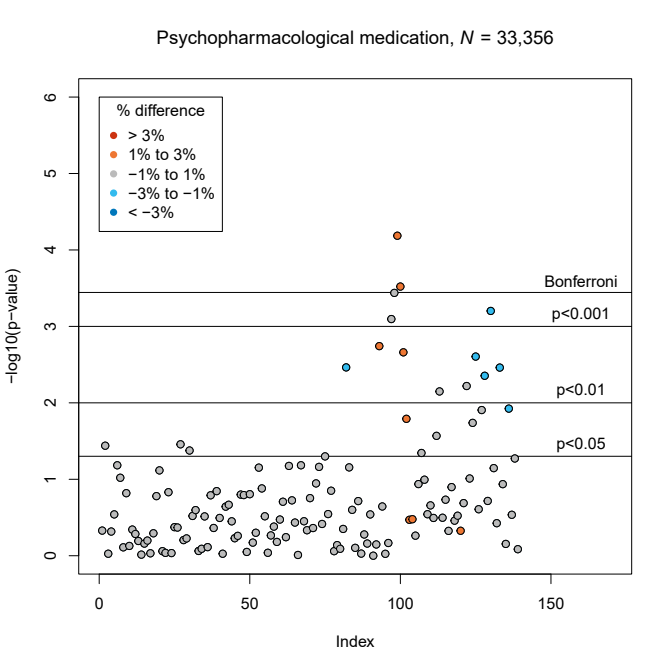


*Supplementary Fig. 4.* **Results of LM3 (4/9; adjusted linear model)**: Associations between psychopharmacological medication and GMV of all 139 IDPs as indexed in Supplementary Table 3. The vertical axis indicates the *p*-value, colours indicate the direction of difference (plus percentage difference). Significant associations after Bonferroni correction: IDPs 99 and 100 (see Supplementary Table 3).


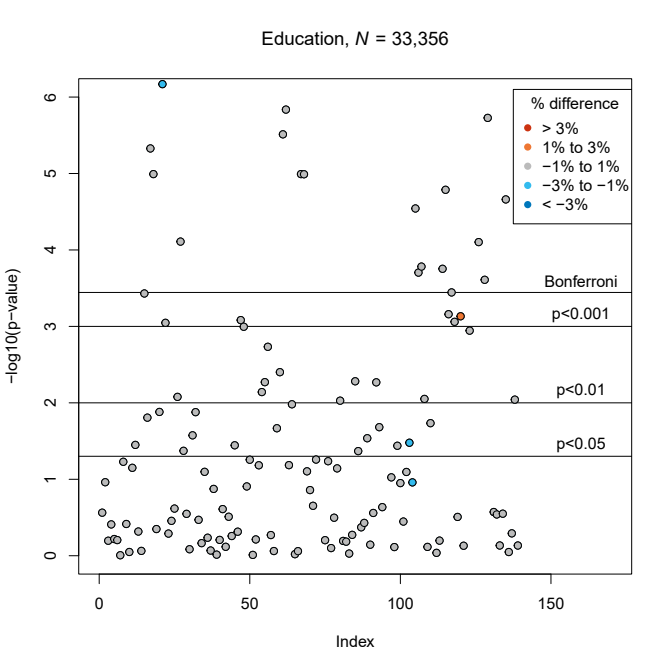


*Supplementary Fig. 5.* **Results of LM3 (5/9; adjusted linear model)**: Associations between educational qualifications and GMV of all 139 IDPs as indexed in Supplementary Table 3. The vertical axis indicates the *p*-value, colours indicate the direction of difference (plus percentage difference). Significant associations after Bonferroni correction: IDPs 17, 18, 21, 27, 61, 62, 67, 68, 73, 74, 95, 96, 105-107, 111, 114, 115, 117, 122, 124-130, and 135 (see Supplementary Table 3).


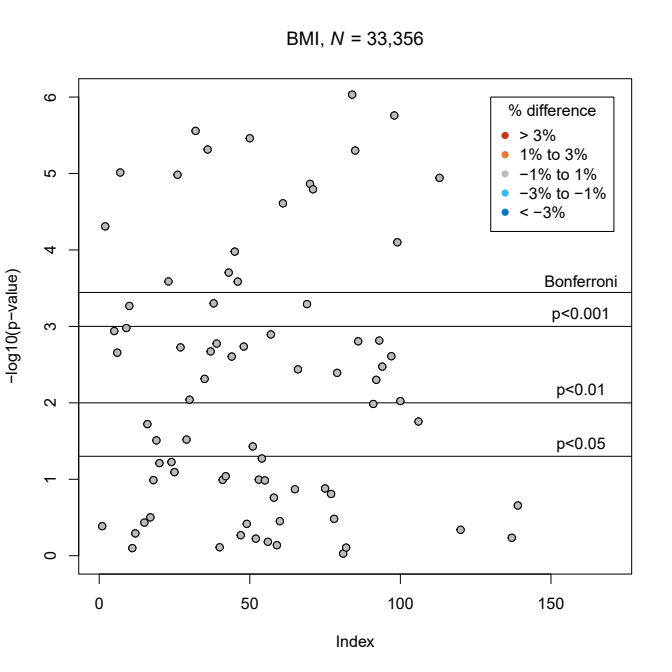


*Supplementary Fig. 6.* **Results of LM3 (6/9; adjusted linear model)**: Associations between BMI and GMV of all 139 IDPs as indexed in Supplementary Table 3. The vertical axis indicates the *p*-value, colours indicate the direction of difference (plus percentage difference). Significant associations after Bonferroni correction: IDPs 2-4, 7, 8, 13, 14, 21-23, 26, 28, 31-34, 36, 43, 45, 46, 50, 61-64, 67, 68, 70-74, 76, 80, 83-85, 87-90, 95, 96, 98, 99, 101-105, 107-119, 121-136, and 138 (see Supplementary Table 3).


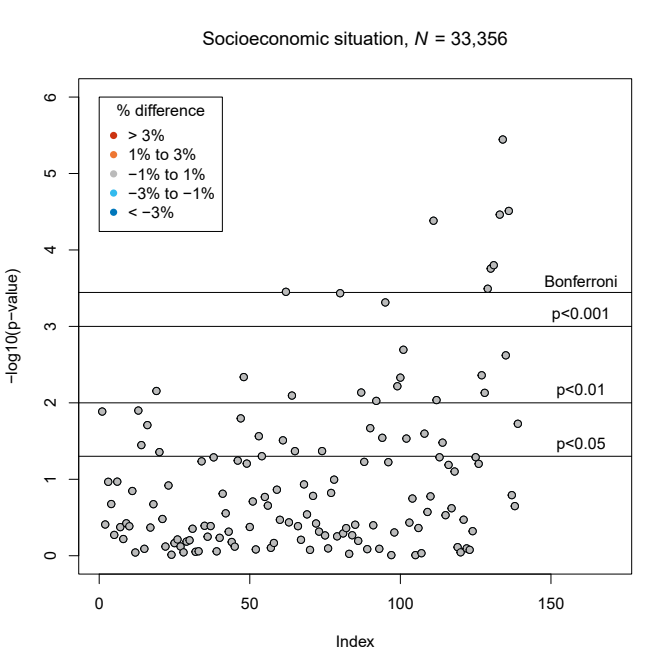


*Supplementary Fig. 7.* **Results of LM3 (7/9; adjusted linear model)**: Associations between socioeconomic situation and GMV of all 139 IDPs as indexed in Supplementary Table 3. The vertical axis indicates the *p*-value, colours indicate the direction of difference (plus percentage difference). Significant associations after Bonferroni correction: IDPs 62, 111, 129-134, and 136 (see Supplementary Table 3).


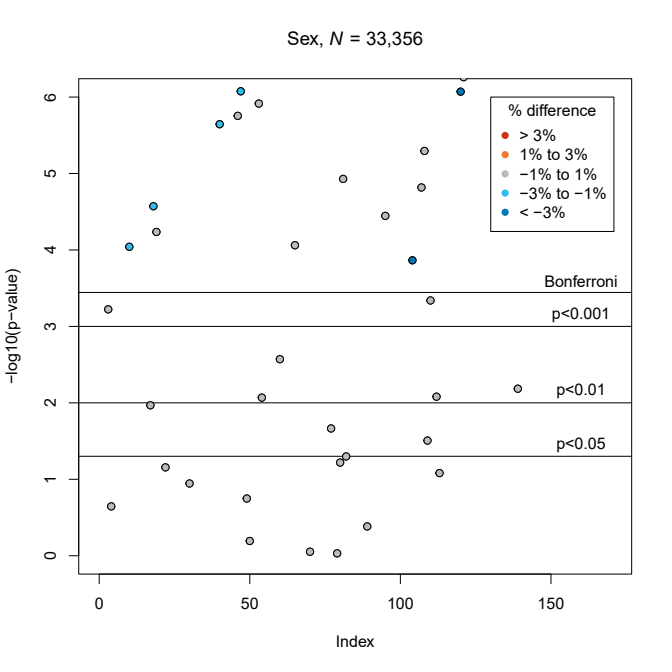


*Supplementary Fig. 8.* **Results of LM3 (8/9; adjusted linear model)**: Associations between sex and GMV of all 139 IDPs as indexed in Supplementary Table 3. The vertical axis indicates the *p*-value, colours indicate the direction of difference (plus percentage difference). Extremely high -log10(*p*)-Values (beyond 6) cannot be displayed, indicating high significance. Significant associations after Bonferroni correction: IDPs 1, 2, 5-16, 18-21, 23-29, 31-48, 51-53, 55-59, 61-69, 71-76, 78, 81, 83-88, 90-108, 111, and 114-138 (see Supplementary Table 3).


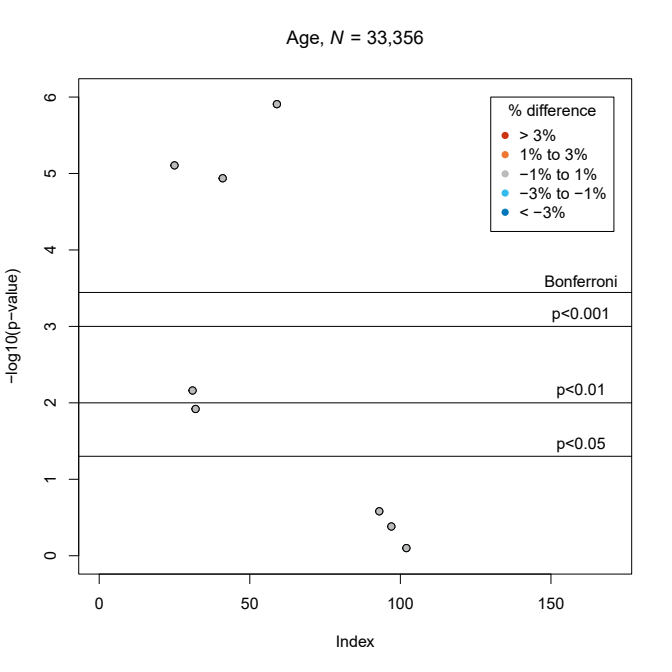


*Supplementary Fig. 9.* **Results of LM3 (9/9; adjusted linear model)**: Associations between age and GMV of all 139 IDPs as indexed in Supplementary Table 3. The vertical axis indicates the *p*-value, colours indicate the direction of difference (plus percentage difference). Extremely high -log10(*p*)-Values (beyond 6) cannot be displayed, indicating high significance. Significant associations after Bonferroni correction: IDPs 1-30, 33-92, 94-96, 98-101, and 103-139 (see Supplementary Table 3).


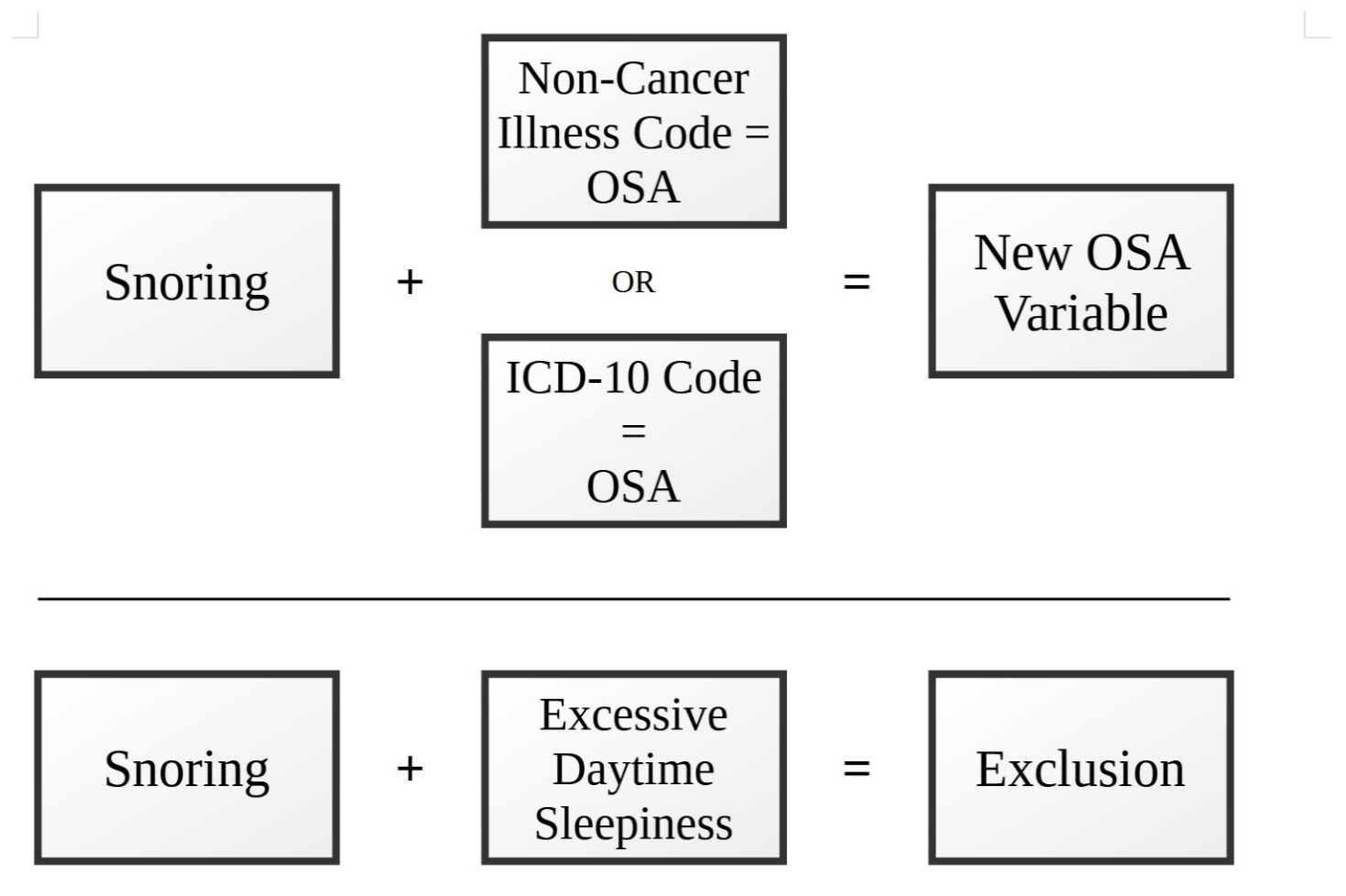


*Supplementary Fig. 10.* **Illustration of the new defined sleep apnoea variable:** Participants who reported snoring and *either* reported sleep apnoea as a non-cancer illness *or* suffered from sleep apnoea according to their ICD-10 main diagnoses were categorized as positive. Participants who did not meet *both* criteria (snoring + illness / ICD-10 code) were not categorised as positive. Participants who were not categorised as positive but reported snoring and excessive daytime sleepiness were excluded from the control group. Participants who did not meet *both* criteria (snoring + sleepiness) were not excluded.


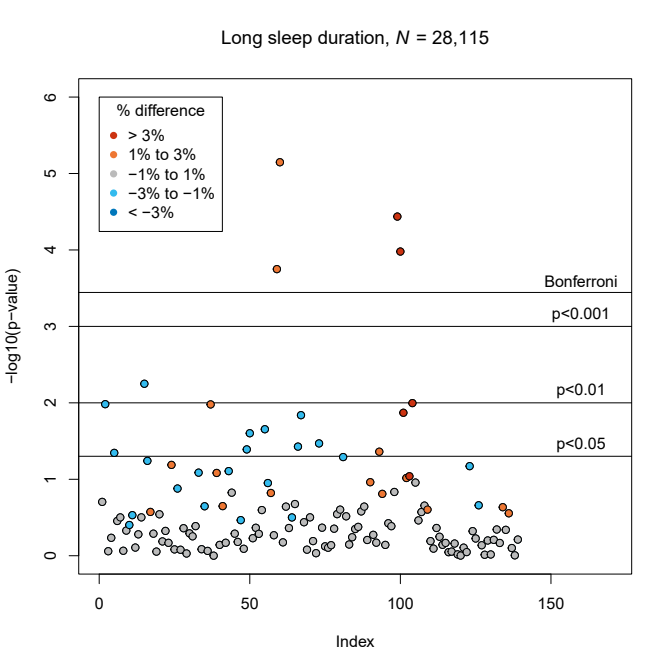


*Supplementary Fig. 11.* **Analyses using the new defined sleep apnoea variable in LM3 (1/2; adjusted linear model)**: Associations between long sleep duration and GMV of all 139 IDPs as indexed in Supplementary Table 3. The vertical axis indicates the *p*-value, colours indicate the direction of difference (plus percentage difference). Significant associations after Bonferroni correction: IDPs 59, 60, 99, and 100 (see Supplementary Table 3).


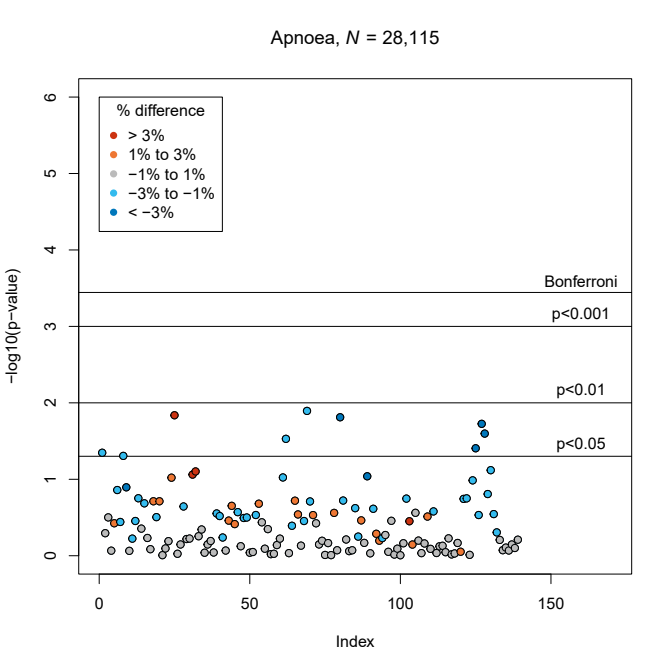


*Supplementary Fig. 12.* **Analyses using the new defined sleep apnoea variable in LM3 (2/2; adjusted linear model)**: Associations between sleep apnoea and GMV of all 139 IDPs as indexed in Supplementary Table 3. The vertical axis indicates the *p*-value, colours indicate the direction of difference (plus percentage difference). Significant associations after Bonferroni correction: None.


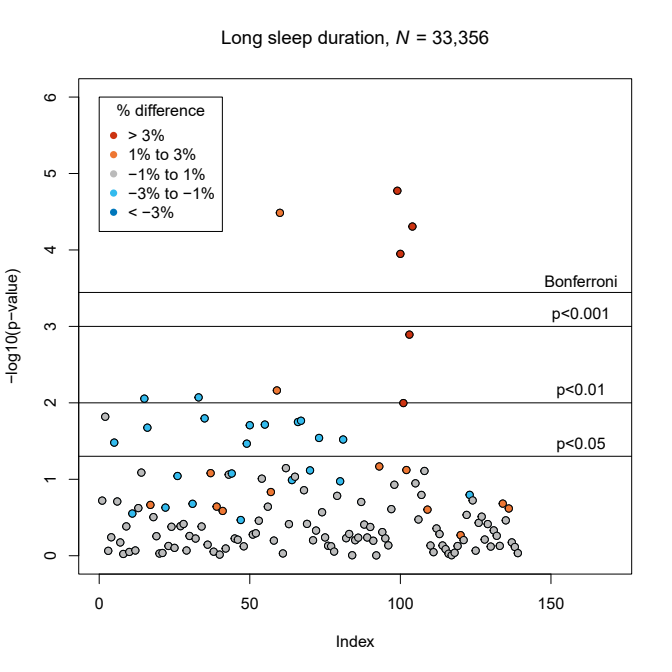


*Supplementary Fig. 13.* **Results of sLM1 (1/2; no further sleep-related variable included besides sleep duration)**: Associations between long sleep duration and GMV of all 139 IDPs as indexed in Supplementary Table 3. The vertical axis indicates the *p*-value, colours indicate the direction of difference (plus percentage difference). Significant associations after Bonferroni correction: IDPs 60, 99, 100, and 104 (see Supplementary Table 3).


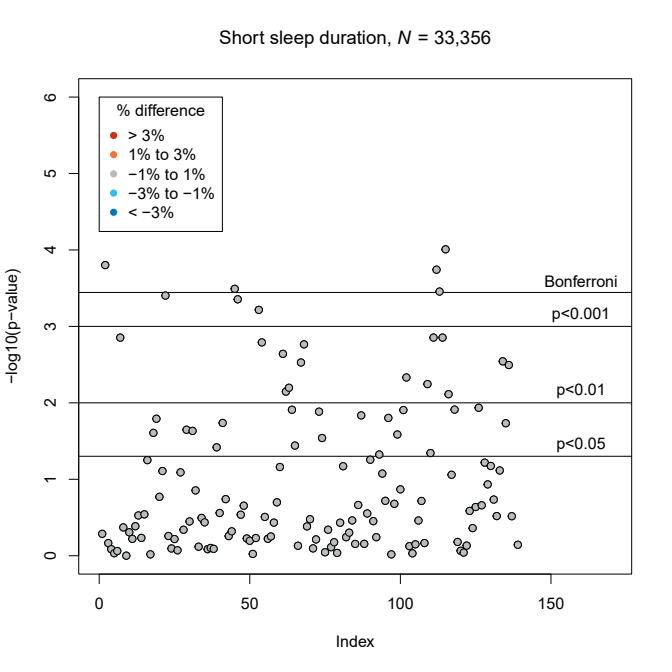


*Supplementary Fig. 14.* **Results of sLM1 (2/2; no further sleep-related variable included besides sleep duration)**: Associations between short sleep duration and GMV of all 139 IDPs as indexed in Supplementary Table 3. The vertical axis indicates the *p*-value, colours indicate the direction of difference (plus percentage difference). Significant associations after Bonferroni correction: IDPs 2, 45, 112, 113, 115, and 138 (see Supplementary Table 3).


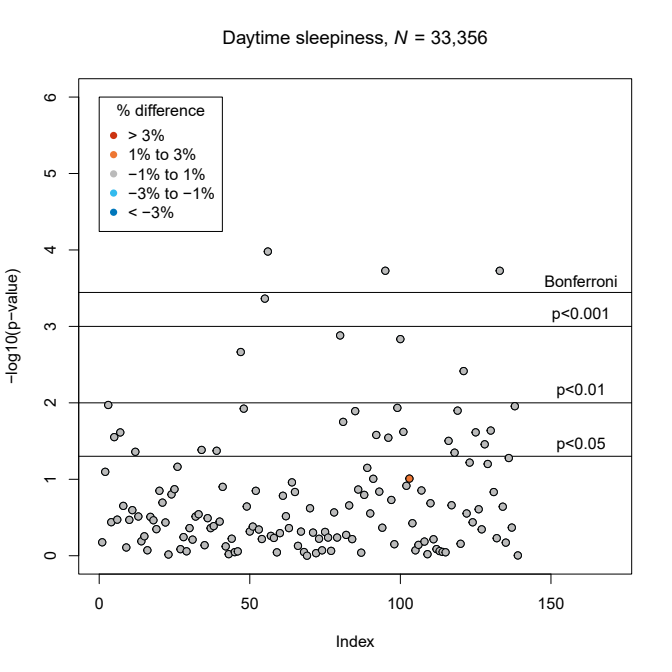


*Supplementary Fig. 15.* **Results of sLM2 (no further sleep-related variable included besides daytime sleepiness)**: Associations between daytime sleepiness and GMV of all 139 IDPs as indexed in Supplementary Table 3. The vertical axis indicates the *p*-value, colours indicate the direction of difference (plus percentage difference). Significant associations after Bonferroni correction: IDPs 56, 95, and 133 (see Supplementary Table 3).


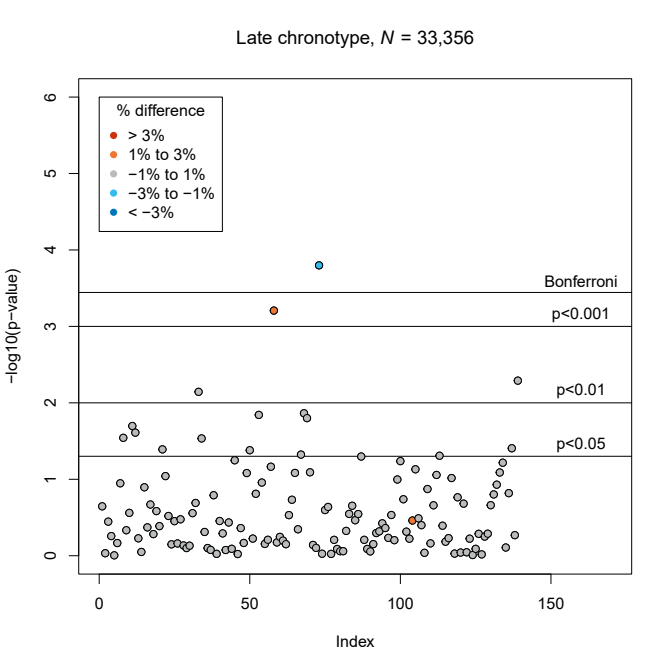


*Supplementary Fig. 16.* **Results of sLM3 (1/2; no further sleep-related variable included besides chronotype)**: Associations between late chronotype and GMV of all 139 IDPs as indexed in Supplementary Table 3. The vertical axis indicates the *p*-value, colours indicate the direction of difference (plus percentage difference). Significant associations after Bonferroni correction: IDP 73 (see Supplementary Table 3).


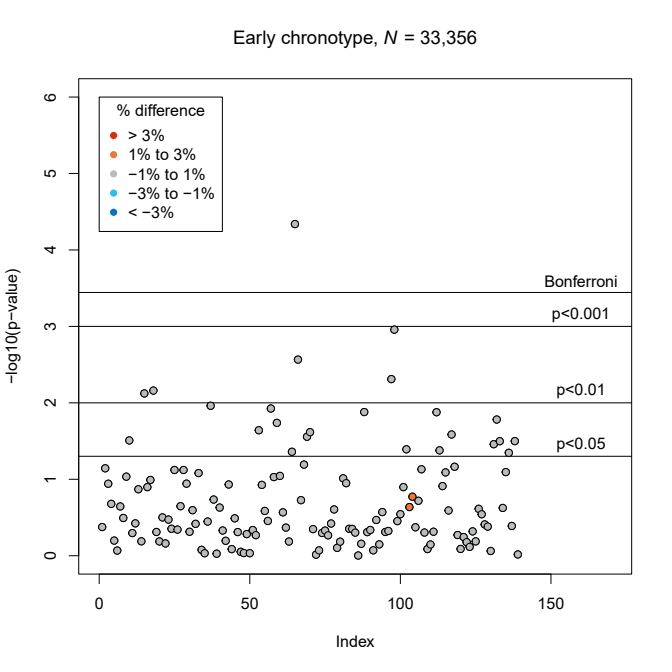


*Supplementary Fig. 17.* **Results of sLM3 (2/2; no further sleep-related variable included besides chronotype)**: Associations between early chronotype and GMV of all 139 IDPs as indexed in Supplementary Table 3. The vertical axis indicates the *p*-value, colours indicate the direction of difference (plus percentage difference). Significant associations after Bonferroni correction: IDP 65 (see Supplementary Table 3).


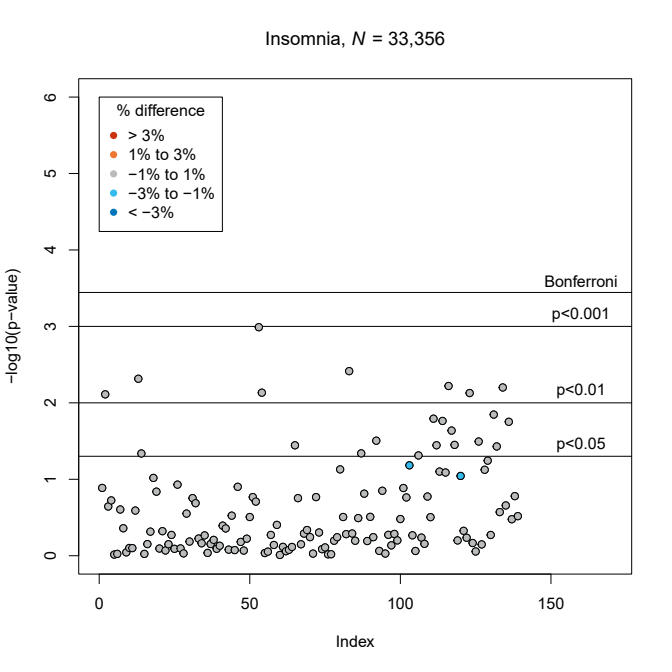


*Supplementary Fig. 18.* **Results of sLM4 (no further sleep-related variable included besides insomnia symptoms)**: Associations between insomnia and GMV of all 139 IDPs as indexed in Supplementary Table 3. The vertical axis indicates the *p*-value, colours indicate the direction of difference (plus percentage difference). Significant associations after Bonferroni correction: None.


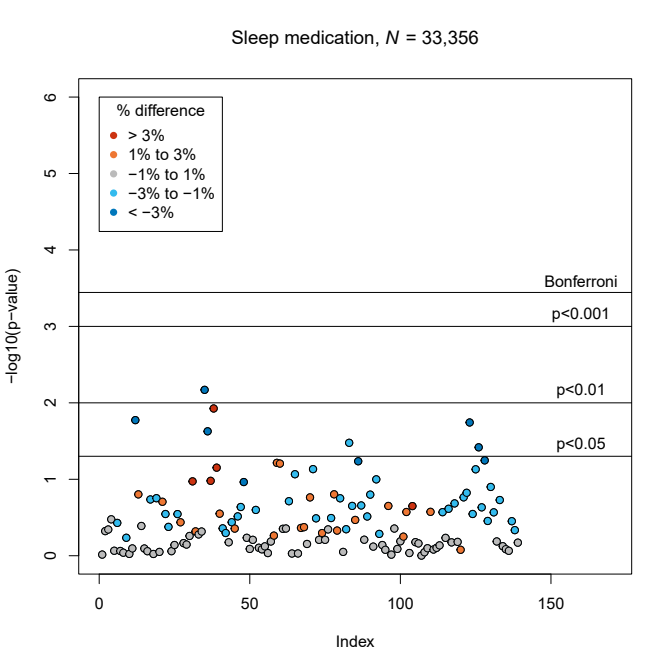


*Supplementary Fig. 19.* **Results of sLM5 (no further sleep-related variable included besides sleep medication)**: Associations between sleep medication and GMV of all 139 IDPs as indexed in Supplementary Table 3. The vertical axis indicates the *p*-value, colours indicate the direction of difference (plus percentage difference). Significant associations after Bonferroni correction: None.


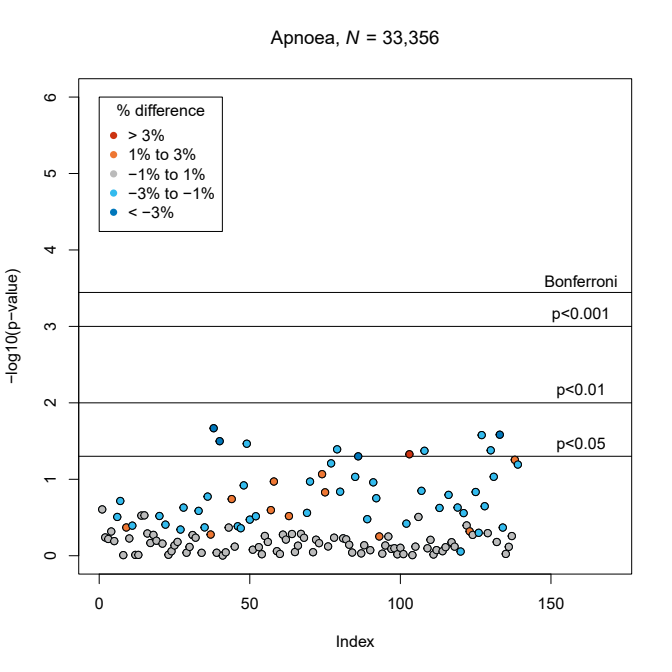


*Supplementary Fig. 20.* **Results of sLM6 (no further sleep-related variable included besides sleep apnoea)**: Associations between sleep apnoea and GMV of all 139 IDPs as indexed in Supplementary Table 3. The vertical axis indicates the *p*-value, colours indicate the direction of difference (plus percentage difference). Significant associations after Bonferroni correction: None.


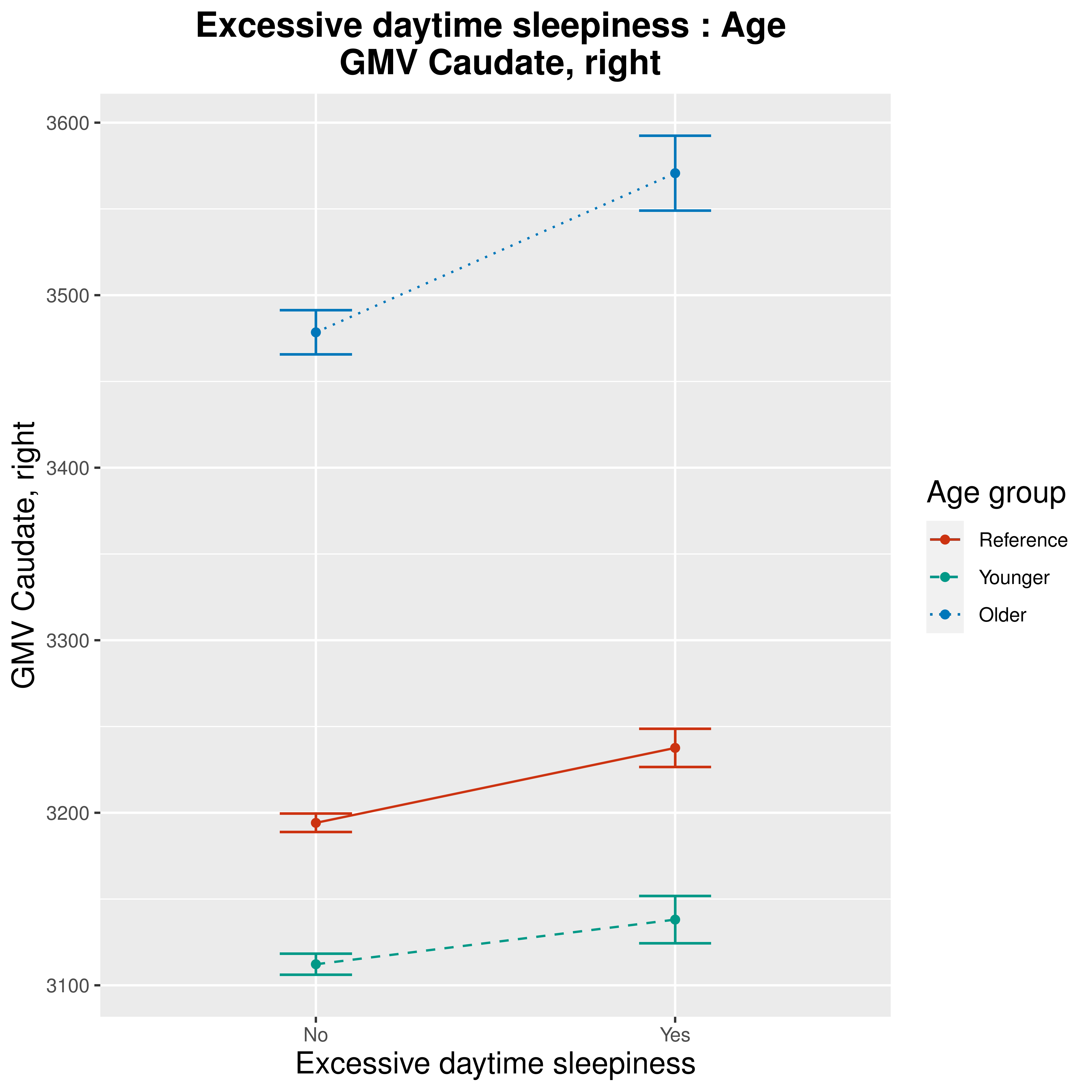


*Supplementary Fig. 21.* **Results of sLM7 (1/4; interaction effects between sex/age and all sleep-related variables included)**: Group-wise mean GMV, depicting interaction effects between excessive daytime sleepiness and age regarding their association with GMV of the right caudate. For illustration purposes, the continuous variable age has been factorised into three categories (“younger”, “reference”, “older”), with a uniform division of the range between minimal and maximal age. Bars indicate group-wise standard error. Units of measurement are mm^3^.


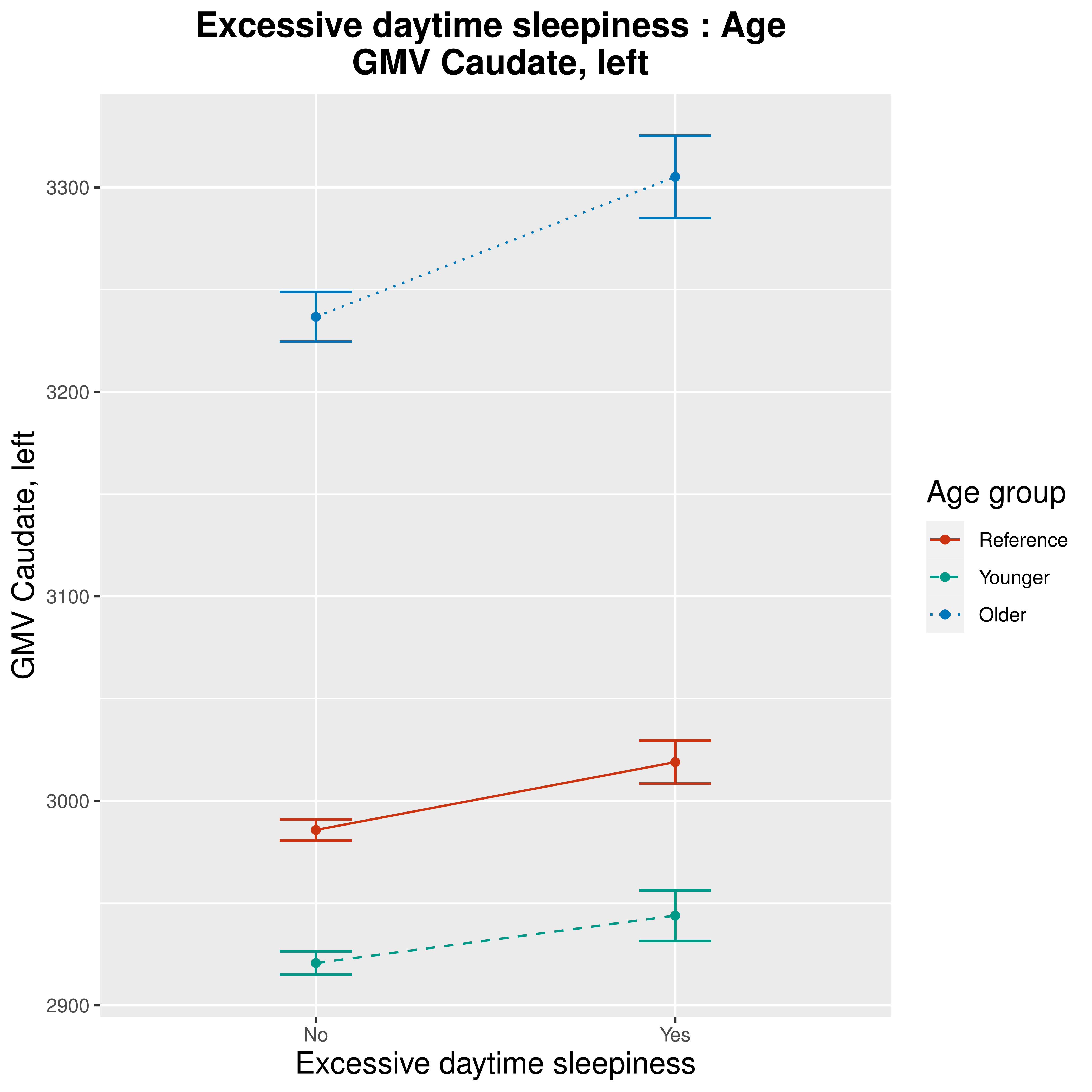


*Supplementary Fig. 22.* **Results of sLM7 (2/4; interaction effects between sex/age and all sleep-related variables included)**: Group-wise mean GMV, depicting interaction effects between excessive daytime sleepiness and age regarding their association with GMV of the left caudate. For illustration purposes, the continuous variable age has been factorised into three categories (“younger”, “reference”, “older”), with a uniform division of the range between minimal and maximal age. Bars indicate group-wise standard error. Units of measurement are mm^3^.


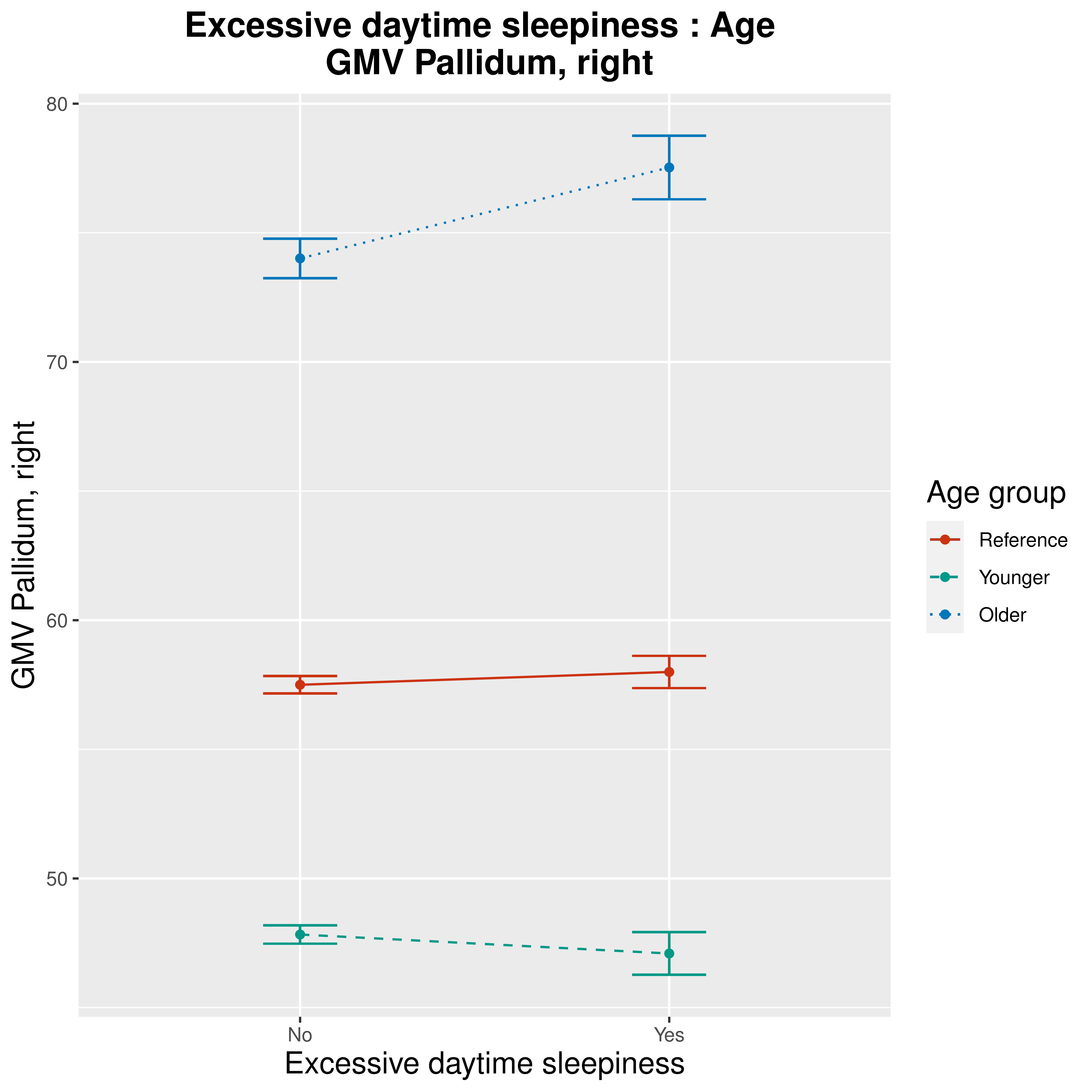


*Supplementary Fig. 23.* **Results of sLM7 (3/4; interaction effects between sex/age and all sleep-related variables included)**: Group-wise mean GMV, depicting interaction effects between excessive daytime sleepiness and age regarding their association with GMV of the right pallidum. For illustration purposes, the continuous variable age has been factorised into three categories (“younger”, “reference”, “older”), with a uniform division of the range between minimal and maximal age. Bars indicate group-wise standard error. Units of measurement are mm^3^.


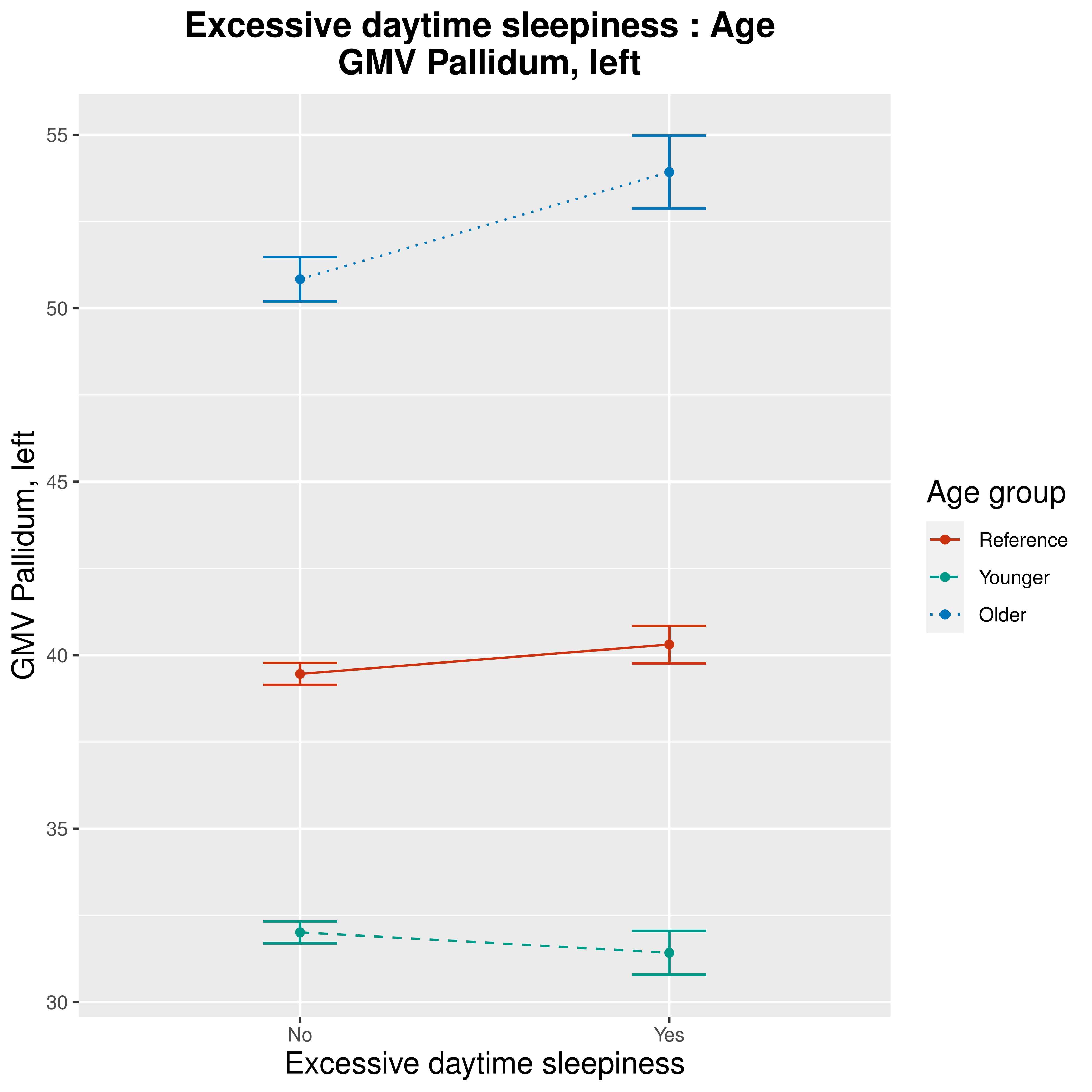


*Supplementary Fig. 24.* **Results of sLM7 (4/4; interaction effects between sex/age and all sleep-related variables included)**: Group-wise mean GMV, depicting interaction effects between excessive daytime sleepiness and age regarding their association with GMV of the left pallidum. For illustration purposes, the continuous variable age has been factorised into three categories (“younger”, “reference”, “older”), with a uniform division of the range between minimal and maximal age. Bars indicate group-wise standard error. Units of measurement are mm^3^.

# **Further findings**

The association between short sleep duration and smaller GMV of the right middle temporal gyrus (anterior division) might be interpreted with regard to findings that sleep disturbances can predict cognitive decline and even dementia (for a systematic review and meta-analysis, see Shi *et al.*^1^). Thinning of the middle temporal gyrus has been described in Alzheimer disease and semantic dementia^2,3^ and is presumably associated with the dissolution of semantic memory, a phenomenon that is suggested to be linked to poor sleep, particularly in the elderly.^4^ The association between short sleep duration and smaller GMV of the left lateral occipital cortex (inferior division) might be interpreted with regard to findings that sleep deprivation is associated with impaired emotion recognition.^5^ As the lateral occipital cortex plays a fundamental role in face recognition,^6,7^ the current results suggest that emotion recognition might be impaired due to deficits in visual object perception rather than due to deficits in interpreting affective social cues. The association between short sleep duration and larger GMV of the cerebellar vermis lobule X might be interpreted with regard to a recent study by Raikes *et al.*^8^: They found that increased cerebellar GMV is related to better psychomotor task performance and poorer sleep quality in individuals with a history of mild traumatic injury. According to the authors, this finding might indicate a compensatory mechanism for trauma-induced psychomotor deficits at the expense of sleep quality. Possibly, this exact mechanism is reflected in the current results, given the assumption that a subsample of the short sleep duration group have an (undetected) history of mild traumatic injury.

The association between excessive daytime sleepiness and smaller GMV of the right paracingulate gyrus might be interpreted with regard to a multimodal meta-analysis on structural brain alterations in patients with obstructive sleep apnoea.^9^ In their study, sleep apnoea, a possible origin of excessive daytime sleepiness, was associated with reduced bilateral GMV of the paracingulate gyri. The associations between excessive daytime sleepiness and smaller GMV of the left occipital pole and the right cerebellar lobule VIIIb are, to our knowledge, new findings that can hardly be integrated into previous research. Interpretations regarding these associations might be possible in the light of future research on excessive daytime sleepiness and related subjects (e.g., sleep apnoea).

The association between early chronotype and reduced GMV of the left frontal orbital cortex might be interpreted with regard to a recent study by Rosenberg *et al.*^10^: Their results indicate reduced GMVs for a rather unspecific pattern of several brain areas in early chronotypes. Although the left frontal orbital cortex was not one of these brain areas, the current outcome might support the hypothesis of generally smaller GMVs in early chronotypes. Rosenberg *et al.*^10^ discuss the idea of specific neural substrates for different chronotypes and suggest that early chronotypes might compensate for smaller neural synaptic responsiveness with higher local connectivity in white matter.^11,12^ An analysis of white matter integrity in the current UK Biobank sample might help to further examine this theoretical approach. The association between late chronotype and smaller GMV of the left temporal fusiform cortex (anterior division), to our knowledge, has not been described in previous research and might be of interest for future studies on chronotype-specific neural substrates.

**Supplementary references**

1. Shi L, Chen SJ, Ma MY, *et al.* Sleep disturbances increase the risk of dementia: a systematic review and meta-analysis. *Sleep Medicine Reviews.* 2018;40:4-16.
2. Chan D, Fox NC, Scahill RI, *et al.* Patterns of temporal lobe atrophy in semantic dementia and Alzheimer's disease. *Annals of Neurology.* 2001;49(4):433-442.
3. Hodges JR, Patterson K. Semantic dementia: a unique clinicopathological syndrome. *The Lancet Neurology.* 2007;6(11):1004-1014.
4. Kang SH, Yoon IY, Lee SD, *et al.* Subjective memory complaints in an elderly population with poor sleep quality. *Aging & Mental Health.* 2017;21(5):532-536.
5. Van Der Helm E, Gujar N, Walker MP. Sleep deprivation impairs the accurate recognition of human emotions. *Sleep.* 2010;33(3):335-342.
6. Nagy K, Greenlee MW, Kovács G. The lateral occipital cortex in the face perception network: an effective connectivity study. *Frontiers in Psychology.* 2012;3:141.
7. Carlson TA, Rauschenberger R, Verstraten FA. No representation without awareness in the lateral occipital cortex. *Psychological Science.* 2007;18(4):298-302.
8. Raikes AC, Satterfield BC, Dailey NS, Bajaj S, Killgore WD. Subjectively poor sleep quality is associated with increased cerebellar grey matter volume following mild traumatic brain injury. *Sleep.* 2018;41:A382.
9. Huang X, Tang S, Lyu X, Yang C, Chen X. Structural and functional brain alterations in obstructive sleep apnea: a multimodal meta-analysis. *Sleep Medicine.* 2019;54:195-204.
10. Rosenberg J, Jacobs HI, Maximov II, Reske M, Shah NJ. Chronotype differences in cortical thickness: grey matter reflects when you go to bed. *Brain Structure and Function.* 2018;223(7):3411-3421.
11. Rosenberg J, Maximov II, Reske M, Grinberg F, Shah NJ. “Early to bed, early to rise”: diffusion tensor imaging identifies chronotype-specificity. *Neuroimage.* 2014;84:428-434.
12. Daselaar SM, Iyengar V, Davis SW, Eklund K, Hayes SM, Cabeza RE. Less wiring, more firing: low-performing older adults compensate for impaired white matter with greater neural activity. *Cerebral Cortex.* 2015;25(4):983-990.
