# Supplementary material for: Associations between sleep health and grey matter volume in the UK Biobank cohort (n = 33 356)
Source: Brain Commun. 2023 Jul 12;5(4):fcad200. doi: 10.1093/braincomms/fcad200 (PMC10365832; doi:10.1093/braincomms/fcad200)
Supplement: fcad200_Supplementary_Data [file fcad200_supplementary_data.zip › Supplementary_material_all_summaries.pdf]

Supplementary material: all summaries

IDPs, Betas, and p-Values of

- **LM3** (original)
- **LM3** (alternative operationalisation of sleep apnoea)
- **sLM1-6**
- **sLM7**

Pages 3-47

LM3_AGE_Summary
LM3_BMI_Summary
LM3_DEPRESSIVE_SYMPTOMS_Summary
LM3_EARLY_CHRONOTYPE_Summary
LM3_EDUCATION_Summary
LM3_EXCESSIVE_DAYTIME_SLEEPINESS_Summary
LM3_INSOMNIA_SYMPTOMS_Summary
LM3_LATE_CHRONOTYPE_Summary
LM3_LONG_SLEEP_DURATION_Summary
LM3_PSYCH_MEDICATION_Summary
LM3_SEX_Summary
LM3_SHORT_SLEEP_DURATION_Summary
LM3_SLEEP_APNOEA_Summary
LM3_SLEEP_MEDICATION_Summary
LM3_SOCIOECONOMIC_STATUS_Summary

Pages 48-92

NewOSA_LM3_AGE_Summary
NewOSA_LM3_BMI_Summary
NewOSA_LM3_DEPRESSIVE_SYMPTOMS_Summary
NewOSA_LM3_EARLY_CHRONOTYPE_Summary
NewOSA_LM3_EDUCATION_Summary
NewOSA_LM3_EXCESSIVE_DAYTIME_SLEEPINESS_Summary
NewOSA_LM3_INSOMNIA_SYMPTOMS_Summary
NewOSA_LM3_LATE_CHRONOTYPE_Summary
NewOSA_LM3_LONG_SLEEP_DURATION_Summary
NewOSA_LM3_PSYCH_MEDICATION_Summary
NewOSA_LM3_SEX_Summary
NewOSA_LM3_SHORT_SLEEP_DURATION_Summary
NewOSA_LM3_SLEEP_APNOEA_Summary
NewOSA_LM3_SLEEP_MEDICATION_Summary
NewOSA_LM3_SOCIOECONOMIC_STATUS_Summary

Pages 93-116

SeparateModels_sLM1_LONG_SLEEP_DURATION_Summary
SeparateModels_sLM1_SHORT_SLEEP_DURATION_Summary
SeparateModels_sLM2_EXCESSIVE_DAYTIME_SLEEPINESS_Summary
SeparateModels_sLM3_EARLY_CHRONOTYPE_Summary
SeparateModels_sLM3_LATE_CHRONOTYPE_Summary
SeparateModels_sLM4_INSOMNIA_SYMPTOMS_Summary
SeparateModels_sLM5_SLEEP_MEDICATION_Summary
SeparateModels_sLM6_SLEEP_APNOEA_Summary

Pages 117-164

Interactions_sLM7_EARLY_CHRONOTYPE_AGE_Summary
Interactions_sLM7_EARLY_CHRONOTYPE_SEX_Summary
Interactions_sLM7_EXCESSIVE_DAYTIME_SLEEPINESS_AGE_Summary
Interactions_sLM7_EXCESSIVE_DAYTIME_SLEEPINESS_SEX_Summary
Interactions_sLM7_INSOMNIA_SYMPTOMS_AGE_Summary
Interactions_sLM7_INSOMNIA_SYMPTOMS_SEX_Summary
Interactions_sLM7_LATE_CHRONOTYPE_AGE_Summary
Interactions_sLM7_LATE_CHRONOTYPE_SEX_Summary
Interactions_sLM7_LONG_SLEEP_DURATION_AGE_Summary
Interactions_sLM7_LONG_SLEEP_DURATION_SEX_Summary
Interactions_sLM7_SHORT_SLEEP_DURATION_AGE_Summary
Interactions_sLM7_SHORT_SLEEP_DURATION_SEX_Summary
Interactions_sLM7_SLEEP_APNOEA_AGE_Summary
Interactions_sLM7_SLEEP_APNOEA_SEX_Summary
Interactions_sLM7_SLEEP_MEDICATION_AGE_Summary
Interactions_sLM7_SLEEP_MEDICATION_SEX_Summary

IDP	Beta	p-Value	LM3_AGE_Summary
1	-60.0632276	0	
2	-57.4132562	1.16532593459759e-312	
3	-3.98344065	8.72E-27	
4	-2.55777983	6.98E-12	
5	-18.9618265	1.31E-62	
6	-13.5736594	1.40E-38	
7	-13.137096	3.83E-32	
8	-11.3699232	9.22E-27	
9	-10.4088486	1.23E-137	
10	-8.52954899	1.65E-124	
11	-11.3635919	1.34E-202	
12	-8.98515103	1.15E-127	
13	-47.776185	0	
14	-48.5748977	0	
15	-24.678951	8.74E-221	
16	-23.0716507	9.56E-200	
17	-4.42808518	6.19E-106	
18	-4.25086345	1.93E-94	
19	-8.75821586	3.19E-159	
20	-11.0533598	4.36E-218	
21	-4.63421496	1.03E-71	
22	-2.90033274	3.57E-42	
23	-13.5692721	8.18E-136	
24	-15.3206697	7.04E-175	
25	-2.38962252	7.83E-06	
26	-4.92850663	1.81E-15	
27	-3.99148145	1.29E-74	
28	-2.60759509	2.35E-39	
29	-5.01182974	4.10E-22	
30	-7.0965664	1.20E-45	
31	1.115040695	0.006894187	
32	-1.24156813	0.012026252	
33	-41.441176	0	
34	-40.4772155	0	
35	-14.6261225	2.46E-111	
36	-11.8769002	6.98E-78	
37	-2.82177662	5.78E-10	
38	-2.94356374	1.21E-11	
39	-5.59386231	2.75E-21	
40	-17.5427538	5.27E-131	
41	-2.42551558	1.15E-05	
42	-12.855575	3.16E-63	
43	-40.8048942	1.15E-192	
44	-46.7371092	1.08E-236	
45	-10.6611409	8.73E-42	
46	-13.8959308	3.41E-63	
47	-3.69341717	1.11E-18	
48	-4.57995422	1.24E-30	
49	-7.08892816	9.03E-220	
50	-7.03825751	6.25E-207	
51	-7.41398642	1.09E-79	
52	-9.28153228	1.43E-123	
53	-3.17245473	1.47E-42	
54	1.499177282	9.94E-13	
55	-23.0588819	0	
56	-26.8693584	0	
57	15.12462992	1.45E-102	

58	20.43533451	1.46E-150
59	1.971919808	1.24E-06
60	4.831672282	6.46E-30
61	-19.5910824	3.76E-140
62	-25.8982854	7.01E-220
63	-6.36292476	4.43E-120
64	-7.40224004	1.90E-128
65	-21.3422682	0
66	-18.9644802	0
67	-6.80122541	1.43E-134
68	-5.85484737	1.20E-88
69	-3.96873457	1.31E-155
70	-2.92901693	1.73E-123
71	-5.79838101	2.57E-30
72	-7.5928492	5.66E-48
73	-5.26001692	1.41E-214
74	-3.93182101	5.94E-146
75	-11.1113127	4.25E-225
76	-8.22950518	1.29E-171
77	-2.9982706	2.28E-22
78	-6.95896671	1.01E-74
79	-12.8753716	1.27E-227
80	-14.4583335	1.87E-289
81	-6.96046614	0
82	-5.88838177	1.75E-270
83	-15.4392726	0
84	-14.7974918	0
85	-4.78189137	1.07E-58
86	-4.47566134	5.14E-60
87	-5.82358477	0
88	-6.91362875	0
89	-8.27919155	0
90	-6.43561855	0
91	-5.12923721	1.19E-62
92	-4.99338389	2.89E-135
93	0.088365288	0.262275157
94	-0.88078922	1.31E-16
95	-29.7617885	5.39E-239
96	-21.9869956	9.24E-143
97	0.161788592	0.414512168
98	4.83904889	4.01E-121
99	24.69217933	0
100	28.41665804	0
101	2.958737343	7.20E-22
102	-0.08631337	0.794501953
103	1.115428627	1.68970450877706e-321
104	1.502298659	0
105	-7.351459	2.77E-182
106	-7.3378909	3.69E-170
107	-6.54126938	0
108	-7.99881471	0
109	-4.33186845	0
110	-3.44535153	0
111	-27.684893	0
112	-4.58995493	1.89E-117
113	-5.24060254	2.11E-131
114	-6.8625998	1.16E-152
115	-6.10219917	5.45E-127

116	-30.0255397	0
117	-4.9879853	1.14E-184
118	-26.7002213	0
119	-44.6617078	0
120	-0.01615196	5.31E-43
121	-51.5534867	0
122	-21.559402	4.12E-155
123	-0.59431512	1.15E-25
124	-24.4875524	2.31E-210
125	-13.4254836	1.42E-195
126	-0.50085196	3.01E-135
127	-12.6374934	1.83E-155
128	-17.1480522	3.03E-299
129	-3.69831325	1.44E-209
130	-16.2753541	1.08E-239
131	-8.65024321	2.47E-135
132	-1.28896611	1.66E-99
133	-10.2871202	3.30E-163
134	-3.59240552	9.76E-39
135	-0.51972985	6.60E-16
136	-4.32608825	2.99E-46
137	-2.0339666	9.93E-306
138	-0.77586205	2.82E-137
139	-2.34880493	0

IDP	Beta	p-Value	LM3_BMI_Summary
1	-1.85990005	0.410523775	
2	-10.2380706	4.91E-05	
3	-8.49225193	3.75E-42	
4	-8.67842969	1.24E-43	
5	6.181469236	0.001150632	
6	5.360482679	0.002207934	
7	8.25571817	9.70E-06	
8	13.61377924	2.10E-14	
9	2.281195806	0.001051109	
10	2.078081474	0.000540126	
11	0.161334969	0.795832197	
12	-0.41144548	0.510041591	
13	-15.7768029	8.08E-19	
14	-18.9190442	1.46E-26	
15	1.165531467	0.368410137	
16	-2.99155634	0.018982753	
17	-0.3404685	0.314784412	
18	-0.56289906	0.102654679	
19	-1.17274999	0.031004025	
20	-1.09254896	0.061398631	
21	-2.59181021	2.22E-09	
22	-1.89082459	1.20E-07	
23	-3.33911152	0.000258372	
24	-1.70821025	0.05950318	
25	-1.56679913	0.080680712	
26	4.582980307	1.04E-05	
27	-1.13646405	0.001880546	
28	-2.89220932	3.89E-18	
29	1.884179936	0.030190116	
30	-2.18729408	0.009094577	
31	4.288506284	5.97E-10	
32	3.889108477	2.77E-06	
33	-11.5288833	6.22E-13	
34	-12.077523	1.40E-14	
35	-3.0718293	0.004853323	
36	-4.86455945	4.85E-06	
37	-2.34703846	0.002126887	
38	-2.53573312	0.000499295	
39	-3.11188575	0.001678258	
40	0.34371093	0.77516767	
41	-1.51795059	0.10183717	
42	2.165832383	0.091278902	
43	-8.55192046	0.000197615	
44	7.165318767	0.002481124	
45	-5.11677612	0.000105171	
46	5.065856345	0.00025937	
47	-0.43130212	0.538971857	
48	-2.0794551	0.00183428	
49	-0.32565731	0.382746621	
50	-1.77353383	3.46E-06	
51	1.367691715	0.037202328	
52	-0.34608607	0.597813552	
53	0.63757275	0.101043705	
54	0.681040595	0.05345428	
55	1.413042932	0.103432974	
56	0.390718421	0.657300855	
57	3.789449598	0.001275124	

58	1.774868373	0.173947013
59	0.237464475	0.727784474
60	-0.66237055	0.352827445
61	5.478015977	2.45E-05
62	7.138342873	1.63E-07
63	2.969285461	7.82E-11
64	3.866701093	4.87E-14
65	-1.20002054	0.134759268
66	-2.17365332	0.003643803
67	-3.25119992	1.63E-12
68	-3.33750033	1.05E-11
69	0.866296754	0.000510895
70	0.90079714	1.37E-05
71	-3.66490025	1.61E-05
72	-6.9905167	1.32E-15
73	-3.1068656	1.65E-28
74	-3.323139	1.14E-38
75	0.87057211	0.131802121
76	-3.26990657	2.95E-11
77	-0.73402812	0.155518041
78	0.6214878	0.3291677
79	-1.91303134	0.004051043
80	-3.51363988	1.05E-07
81	0.022622588	0.937643379
82	-0.07671818	0.783035225
83	-3.55581418	5.68E-10
84	-2.94313781	9.29E-07
85	-2.26327646	5.01E-06
86	-1.45067203	0.001565853
87	-2.46434716	3.31E-27
88	-2.36651242	1.12E-24
89	-2.39185212	8.76E-17
90	-2.47251128	2.09E-31
91	-1.31836554	0.010345355
92	-0.94591818	0.004999729
93	0.419177074	0.0015306
94	0.523761074	0.003357709
95	11.7001069	6.49E-15
96	18.64982159	4.19E-38
97	-1.00776599	0.002456098
98	1.652503871	1.74E-06
99	-3.18347494	7.94E-05
100	-2.1779459	0.009479707
101	-7.46175786	3.28E-47
102	-11.8758	1.59E-100
103	-0.33520937	3.95E-12
104	-0.46241259	3.02E-17
105	-2.7023698	2.23E-10
106	-1.0457521	0.017541895
107	-5.36233546	1.11E-109
108	-6.15435273	2.40E-121
109	-2.3969814	2.20E-84
110	-2.73286749	3.22E-109
111	6.249510772	8.83E-12
112	-2.44974961	1.92E-13
113	-1.57547342	1.14E-05
114	-5.83243517	7.77E-41
115	-5.85469039	5.39E-43

116	-19.7267347	2.43E-68
117	-2.1818196	2.99E-14
118	-16.9056117	1.52E-52
119	-27.3235495	1.82E-54
120	0.001462977	0.457442682
121	-26.0766122	1.88E-43
122	-30.6259753	4.75E-112
123	-0.76143771	1.25E-15
124	-28.5457875	2.62E-103
125	-17.8739901	1.85E-124
126	-0.31191659	2.91E-20
127	-17.8236494	9.62E-111
128	-24.0033668	3.73E-210
129	-3.09804751	3.19E-54
130	-27.699431	2.20E-246
131	-17.99802	5.85E-206
132	-2.01908693	4.98E-87
133	-21.7205583	1.51E-255
134	-12.0124593	2.96E-147
135	-2.31408798	2.54E-101
136	-14.6735419	1.79E-181
137	-0.04968819	0.582418547
138	-0.51711124	2.67E-23
139	-0.11140608	0.220295995

IDP	Beta	p-Value	LM3_DEPRESSIVE_SYMPTOMS_Summary
1	-23.4990322	0.371758586	
2	-31.1855978	0.288062111	
3	-2.0345001	0.779197127	
4	-0.09982527	0.98906419	
5	13.84686915	0.53158853	
6	3.921831721	0.847452442	
7	-4.03641324	0.852589069	
8	21.64667836	0.296212162	
9	-3.21308928	0.691766896	
10	-10.1787118	0.14541492	
11	-5.22482873	0.471650607	
12	-7.16817974	0.324189383	
13	-31.730324	0.125681288	
14	-38.203056	0.064072014	
15	-15.2817718	0.311043433	
16	1.375950484	0.926151486	
17	3.40965081	0.387169421	
18	0.97521024	0.808084592	
19	2.511512332	0.691499414	
20	5.208769945	0.443618646	
21	1.612351509	0.749192592	
22	8.413613459	0.04301477	
23	9.120725463	0.391225562	
24	12.03517982	0.254072188	
25	-3.06118029	0.769396191	
26	8.816708918	0.466198295	
27	18.39418636	1.55E-05	
28	5.001551108	0.196906257	
29	17.99114632	0.075415898	
30	0.652018015	0.946743231	
31	-20.7869983	0.00992369	
32	-14.0138196	0.146749856	
33	-30.205414	0.105230261	
34	-28.6667269	0.116455005	
35	-2.83166959	0.823499918	
36	-9.70082019	0.43353825	
37	-26.8853844	0.002504884	
38	9.465470762	0.264282984	
39	-15.1580709	0.188595016	
40	8.671978195	0.535896975	
41	-5.72446008	0.596119533	
42	-27.2013515	0.068479539	
43	16.42165943	0.539209275	
44	-3.18235215	0.908096714	
45	-4.48949168	0.770025142	
46	-2.73005532	0.865704768	
47	2.670271782	0.743866137	
48	4.117256638	0.596126496	
49	-5.58931942	0.198145464	
50	-4.05923213	0.361391413	
51	-1.75692596	0.818154166	
52	-4.03106558	0.597618238	
53	-5.56462186	0.218914309	
54	0.820934558	0.841499312	
55	-28.7914535	0.004370891	
56	-27.0592527	0.008312157	
57	16.88585459	0.217502281	

58	29.74072163	0.050345
59	-11.7392697	0.139381449
60	-18.2403104	0.027964351
61	-13.1154434	0.385496885
62	-12.997312	0.412609731
63	-3.90309821	0.462567493
64	-3.82497871	0.521799094
65	-6.32604332	0.498237418
66	-5.21082584	0.549333854
67	-7.03374854	0.189184439
68	-6.20601104	0.277324755
69	-5.04214892	0.082297832
70	-5.95978477	0.013438726
71	16.51044435	0.095027331
72	3.005924912	0.767705242
73	3.076445328	0.345702986
74	-0.41929279	0.88775989
75	-2.71312976	0.686616095
76	-0.6914349	0.903837324
77	0.932467676	0.876828569
78	5.993128995	0.418915384
79	-5.05589873	0.514051576
80	6.5216803	0.396472904
81	-0.65899972	0.84479801
82	-4.27449818	0.187532263
83	3.707483316	0.578631649
84	-3.19899165	0.646843155
85	-8.38539459	0.146258908
86	2.646033972	0.620248355
87	-1.51925059	0.566900149
88	-0.02036859	0.993945765
89	2.813515167	0.400251755
90	-0.23407824	0.924390959
91	3.615878285	0.545763707
92	5.521802251	0.15923669
93	0.721444095	0.63941109
94	0.514074038	0.804678323
95	-12.7453974	0.465600912
96	18.7254344	0.265076813
97	-7.51998756	0.052203368
98	-3.69554195	0.358272353
99	28.59077799	0.002329639
100	35.97761449	0.000232281
101	18.07379288	0.002636545
102	19.0598692	0.003238509
103	1.20326004	0.032330601
104	1.190510183	0.061646131
105	-4.84392767	0.328481795
106	-3.91128144	0.445391269
107	-3.3962618	0.224342656
108	-6.54703884	0.031607779
109	1.022619703	0.474392968
110	0.521878925	0.714689973
111	-17.8751754	0.093484841
112	2.136679491	0.581492322
113	4.379821866	0.294553984
114	4.266435067	0.399807525
115	6.796067609	0.169888865

116	8.337014865	0.524990859
117	3.430225079	0.304569672
118	11.11256941	0.388193767
119	-29.7906046	0.144795186
120	0.013604767	0.552795474
121	-32.0448775	0.143982111
122	-8.05901404	0.609777052
123	0.847596292	0.444128914
124	-9.49858412	0.535877802
125	-5.02106776	0.565290378
126	0.239825905	0.54219679
127	-4.02651715	0.663166921
128	4.541379621	0.6125533
129	2.077149539	0.370987793
130	-3.57120465	0.708093414
131	4.574039403	0.500770826
132	0.059937514	0.959666649
133	2.166049414	0.767929239
134	2.110584658	0.695063957
135	2.152566967	0.08664022
136	6.505263306	0.270998006
137	0.854971269	0.416378026
138	0.246594652	0.683527396
139	0.33612946	0.750723761

IDP	Beta	p-Value	LM3_EARLY_CHRONOTYPE_Summary
1	-16.8776232	0.445978165	
2	-37.8254408	0.125802705	
3	-9.30212165	0.127783504	
4	-7.56406415	0.21725668	
5	-8.390085	0.652472869	
6	-2.75391164	0.872500799	
7	-17.7912171	0.330545049	
8	-16.0601056	0.357189286	
9	-11.522227	0.091217243	
10	-12.3838998	0.035345161	
11	-3.75477913	0.538856509	
12	-5.04548345	0.409697877	
13	-24.292051	0.163671855	
14	-7.0863338	0.683225638	
15	-33.1119527	0.009117003	
16	-17.7269962	0.155997409	
17	-5.38805366	0.104490318	
18	-8.60314224	0.010908751	
19	-4.51561108	0.39664634	
20	-3.17995164	0.578451239	
21	-3.69703532	0.383806707	
22	-0.63093752	0.856927189	
23	8.863304704	0.322238367	
24	-6.78471303	0.444952527	
25	-15.608849	0.075752497	
26	-8.01610106	0.431231179	
27	4.732900137	0.186432265	
28	5.941420895	0.068587647	
29	14.62632508	0.085942521	
30	-5.16858365	0.529303765	
31	-6.76025883	0.319111966	
32	-6.18046553	0.447060187	
33	27.25457559	0.082456949	
34	1.737630345	0.909991114	
35	1.33670754	0.900453358	
36	9.225317263	0.376261217	
37	18.94044416	0.011407245	
38	9.663573828	0.175740561	
39	-0.6662895	0.945261129	
40	-14.8261235	0.208636613	
41	-8.19775283	0.36722344	
42	-6.99276307	0.577919112	
43	-34.6448765	0.123820011	
44	5.974743018	0.796802693	
45	-9.87434119	0.44493295	
46	-6.43045753	0.636037905	
47	-0.21527129	0.975035103	
48	0.058852378	0.992819082	
49	-2.34984794	0.520384496	
50	-0.43198221	0.908131222	
51	-4.60263748	0.474250803	
52	-3.99984815	0.533794925	
53	-7.7979265	0.040682447	
54	-4.72836858	0.171194936	
55	-8.68811504	0.3068694	
56	-7.28291786	0.398716346	
57	29.53904434	0.010382335	

58	22.31895681	0.081016265
59	-15.1318483	0.023606719
60	-11.044356	0.113878508
61	-12.0199557	0.34472138
62	12.3849232	0.353663158
63	-1.30685771	0.77011503
64	11.02665321	0.02824864
65	-30.9662804	8.21E-05
66	-21.8047172	0.002916097
67	-5.20628029	0.248250124
68	-8.03275616	0.094820728
69	-5.24416835	0.031801055
70	-4.48825549	0.026990795
71	6.02067511	0.469515999
72	0.64321925	0.940142124
73	0.92700532	0.735690985
74	1.984416256	0.42743914
75	-4.18062673	0.460168781
76	-2.67497433	0.578696673
77	-4.38109171	0.386966495
78	7.518114827	0.228341777
79	1.814607256	0.780825946
80	-2.32714522	0.719245318
81	-4.27719877	0.131181806
82	-4.24509656	0.119956998
83	-4.16705429	0.458326241
84	4.740678349	0.419888896
85	3.069605811	0.527476744
86	-0.46335256	0.917896677
87	-1.23409232	0.580531985
88	-5.64071297	0.01254773
89	1.678253269	0.551103811
90	1.232768633	0.552650875
91	-1.30097541	0.796228003
92	-3.35319317	0.309841196
93	-0.30470445	0.81413765
94	2.123839161	0.224831244
95	12.22007093	0.405901573
96	-7.48565305	0.596598835
97	-9.25116093	0.004548112
98	-10.8032095	0.001421301
99	-8.6799135	0.27211782
100	-9.90520321	0.228541306
101	-8.68543807	0.085989026
102	-12.2079786	0.02506527
103	0.550978884	0.244274594
104	0.738940896	0.168170926
105	-3.44427771	0.409101331
106	-5.95249333	0.16766497
107	-4.33841903	0.065188394
108	-1.78101399	0.487270394
109	0.068581756	0.954548355
110	0.328173248	0.784773354
111	4.328813939	0.629396178
112	7.336448357	0.024550151
113	6.419882878	0.067957898
114	5.696191533	0.181716554
115	6.247231107	0.13388363

116	10.9837031	0.319763277
117	5.988101347	0.033235569
118	18.29638382	0.091440435
119	10.87624937	0.527077724
120	0.004715154	0.806916135
121	11.05864873	0.549137157
122	-5.82666483	0.661095218
123	-0.34755572	0.709304672
124	-9.60412218	0.457097059
125	-3.63840199	0.62059315
126	0.334979482	0.311828507
127	-8.73516219	0.261633784
128	5.786474183	0.443311277
129	1.437076251	0.462136929
130	0.730345743	0.927515973
131	11.69125573	0.040910607
132	2.30777429	0.020710634
133	12.98168439	0.035641293
134	4.585227313	0.31166917
135	1.701918261	0.107523789
136	9.204660139	0.064258598
137	0.76670646	0.386565583
138	0.950613468	0.061909114
139	0.070691211	0.936733993

IDP	Beta	p-Value	LM3_EDUCATION_Summary
1	21.51131306	0.273380976	
2	-35.0947059	0.109252264	
3	-2.56097	0.636376326	
4	-4.68038559	0.389303128	
5	8.556261475	0.604567846	
6	7.460556779	0.623971758	
7	-0.2879534	0.98583204	
8	29.20600824	0.059027481	
9	5.263512496	0.384289909	
10	-0.6996912	0.893345646	
11	9.790676403	0.070786102	
12	11.41207427	0.035503067	
13	10.86477622	0.482405093	
14	-2.62694517	0.864559664	
15	-40.0908815	0.000370985	
16	-26.7929347	0.015619054	
17	-13.4755326	4.70E-06	
18	-13.2287163	1.02E-05	
19	-3.58782671	0.447614132	
20	-12.585379	0.013153953	
21	-18.7053646	6.78E-07	
22	-10.3069941	0.000898042	
23	-5.19570911	0.512916843	
24	-7.36723007	0.349652504	
25	9.127907334	0.241584293	
26	23.82246642	0.008354483	
27	-12.5540996	7.77E-05	
28	-5.87147073	0.042428695	
29	-8.10506939	0.283272353	
30	-1.63660546	0.822285465	
31	13.3428904	0.02660856	
32	17.85970496	0.013236788	
33	-13.300337	0.339268658	
34	5.537902359	0.684549413	
35	16.59753971	0.079890787	
36	5.097143565	0.581465075	
37	1.214316414	0.854871784	
38	9.492865346	0.13367561	
39	-0.35023223	0.967539109	
40	5.168329185	0.621147818	
41	-9.3660982	0.24538571	
42	3.344941777	0.764083634	
43	20.30914842	0.309034524	
44	12.22370799	0.552510095	
45	24.04749295	0.035937314	
46	8.421043189	0.484669861	
47	20.39945645	0.000827159	
48	19.06627224	0.0010109	
49	-4.98702109	0.124020092	
50	-6.3571096	0.055518236	
51	0.17636715	0.975334395	
52	2.891273125	0.61204449	
53	-6.22146632	0.065573216	
54	-8.23199384	0.007227907	
55	20.99724676	0.005362161	
56	23.83060185	0.001848785	
57	6.320543132	0.536332017	

58	-1.88153132	0.868265183
59	13.63107799	0.021497815
60	17.84637567	0.003970084
61	52.64802853	3.07E-06
62	57.05057786	1.46E-06
63	7.309772864	0.06532518
64	11.41425232	0.010447966
65	-0.39852662	0.954420488
66	-1.04939293	0.871672598
67	-17.6510355	1.02E-05
68	-18.8185518	1.02E-05
69	-3.80890251	0.078698067
70	-2.66697449	0.138368768
71	-9.00970454	0.222303987
72	-14.5656202	0.055195711
73	-13.2485943	5.37E-08
74	-11.6891923	1.36E-07
75	-2.44603271	0.626072217
76	-8.09890562	0.058002004
77	1.172901835	0.793965733
78	-5.52384898	0.318266259
79	10.40585465	0.072000424
80	14.91858259	0.009368145
81	-1.17410492	0.640339436
82	-1.08336871	0.654535902
83	-0.39711414	0.93648273
84	3.246211699	0.533411658
85	12.03339804	0.005223893
86	8.075774363	0.042784236
87	-1.58057774	0.42483694
88	1.788651413	0.372061621
89	5.450452286	0.029042735
90	-0.66679207	0.71723619
91	4.869223771	0.275801096
92	-8.14833427	0.00539344
93	2.656776003	0.020819262
94	1.85707098	0.231410221
95	69.77425327	8.80E-08
96	75.38727255	1.87E-09
97	4.836665763	0.094370265
98	0.877517723	0.770115132
99	14.66484877	0.036429369
100	11.59032147	0.11212117
101	-4.13070136	0.357176499
102	-8.4534276	0.080234989
103	-0.89346781	0.033254788
104	-0.76043166	0.109794155
105	-15.4834938	2.87E-05
106	-14.2415253	0.000197708
107	-7.86110779	0.000165083
108	-5.94943196	0.008885642
109	-0.31667696	0.766651282
110	-2.51141015	0.018446108
111	41.19490653	2.25E-07
112	0.301760952	0.916943718
113	1.482836656	0.634503268
114	-14.1883911	0.000176423
115	-15.9318921	1.63E-05

116	-33.2153395	0.000692879
117	-8.90019566	0.000359369
118	-32.0060674	0.000871346
119	-15.4599965	0.3107136
120	0.057753795	0.000737394
121	-5.4311444	0.74008091
122	-69.1862578	4.40E-09
123	-2.69093898	0.001136651
124	-60.2888284	1.42E-07
125	-38.3079895	4.22E-09
126	-1.159665	7.89E-05
127	-43.0218306	4.60E-10
128	-24.5475509	0.000245671
129	-8.26291151	1.87E-06
130	-36.0784187	4.06E-07
131	-5.61776818	0.267976428
132	-0.93926945	0.288398942
133	-1.85860789	0.734464737
134	-4.32518046	0.281878856
135	-3.9818383	2.18E-05
136	-0.58659936	0.894216451
137	-0.51766502	0.509764773
138	1.178191107	0.009079312
139	-0.26715765	0.735174541

IDP	Beta	p-Value	LM3_EXCESSIVE_DAYTIME_SLEEPINESS_Summary
1	13.70012869	0.559848716	
2	-36.1722423	0.167663587	
3	-16.6509883	0.010195378	
4	-6.15810845	0.343773018	
5	-42.9744164	0.029716271	
6	-17.0556134	0.3488984	
7	-39.3713316	0.042423588	
8	-21.1840978	0.2523627	
9	-1.86165448	0.797022567	
10	-5.56114428	0.373109652	
11	-7.09649698	0.27366488	
12	-13.4459143	0.038395482	
13	-14.5849436	0.430604672	
14	-5.59633917	0.76133605	
15	-6.11927236	0.649679757	
16	-0.81580747	0.950934653	
17	-3.45737781	0.326186635	
18	-2.51065295	0.483813296	
19	4.158820181	0.461884128	
20	8.777933336	0.148300158	
21	-5.06524296	0.260772033	
22	-2.67954836	0.47052778	
23	-0.46719488	0.960778989	
24	-13.7567644	0.144373654	
25	14.48108534	0.120469498	
26	20.79008875	0.054370582	
27	1.177348218	0.756743904	
28	2.07949644	0.548011653	
29	-1.08447002	0.904482644	
30	-6.43640076	0.460324723	
31	5.217629388	0.468628792	
32	-7.58470152	0.379184156	
33	18.92974717	0.255623476	
34	32.74987821	0.044635566	
35	4.913352355	0.664767239	
36	10.68590355	0.334076394	
37	5.590121357	0.481570502	
38	6.449641431	0.394380789	
39	19.89273403	0.053370624	
40	11.39557943	0.362394362	
41	13.30031981	0.167967564	
42	3.880316103	0.771048905	
43	3.422489478	0.886064815	
44	-13.535034	0.582490521	
45	0.922401324	0.946379884	
46	1.404092659	0.922417128	
47	-21.8415556	0.00276919	
48	-17.1324087	0.013542697	
49	-4.41540066	0.255011243	
50	-2.87357125	0.469395852	
51	-4.95548607	0.467765432	
52	-9.62636199	0.158145505	
53	-1.60319703	0.691659748	
54	2.968501481	0.418132647	
55	-31.1663018	0.000551621	
56	-35.087547	0.00012735	
57	-8.27439417	0.498642158	

58	-7.46174375	0.582463675
59	0.000791087	0.999911011
60	5.495772229	0.458405388
61	21.11271129	0.117779092
62	16.48573548	0.244588661
63	-3.22374075	0.496892191
64	-7.98878185	0.134129468
65	-9.85729715	0.237351556
66	-1.33483592	0.86364086
67	4.295521767	0.369274024
68	1.63508359	0.748604503
69	0.378063364	0.884018752
70	2.784097519	0.196004844
71	5.648666525	0.522469264
72	0.030226997	0.997346402
73	-0.9866851	0.734889062
74	0.783735806	0.767693365
75	4.330389963	0.47089715
76	-2.52802838	0.620883714
77	1.143784099	0.831427444
78	-7.39278038	0.264242058
79	-3.79932261	0.582966335
80	-22.284554	0.001178757
81	-6.56828716	0.02891661
82	-1.72988516	0.550369439
83	8.433207069	0.157215493
84	3.592164443	0.564587693
85	12.97955292	0.011803257
86	7.092268484	0.137000911
87	-0.32242231	0.891765044
88	-3.5433847	0.139407915
89	5.323690214	0.074727042
90	2.009677585	0.361598719
91	8.876474381	0.096810718
92	8.143965428	0.020096487
93	-1.94207795	0.157897741
94	-1.52234248	0.412226257
95	-57.433587	0.000232145
96	-30.4332533	0.042555949
97	4.662700189	0.177693892
98	1.815305257	0.613363933
99	19.78019711	0.018344499
100	26.52415176	0.002375622
101	11.06809934	0.039200644
102	7.96741872	0.168173795
103	0.832904317	0.097138577
104	0.484627147	0.394306489
105	0.94655414	0.830696572
106	1.222314007	0.789446327
107	-3.75792535	0.132228853
108	-1.19315569	0.660949179
109	-0.18918041	0.882202556
110	-1.78924141	0.160520093
111	2.298276235	0.809187481
112	-1.8862796	0.585852722
113	-1.60482984	0.667163087
114	-1.93100257	0.669603877
115	-0.73380041	0.86820188

116	-28.4730129	0.015068122
117	-4.35144914	0.144759618
118	-26.1018321	0.023246835
119	-46.273554	0.011213131
120	-0.00654893	0.749019994
121	-57.5874028	0.003283025
122	-15.2990389	0.27797269
123	-2.05990213	0.037315002
124	-13.3663994	0.329358085
125	-17.9845241	0.021111543
126	-0.49334969	0.160360343
127	-6.45359718	0.434428423
128	-18.5521085	0.020537227
129	-4.21623417	0.042028737
130	-20.3679402	0.016804569
131	-10.11114	0.095627528
132	0.367736167	0.72828032
133	-25.592661	9.48E-05
134	-7.2159338	0.133462465
135	-0.70875632	0.527587256
136	-11.7881116	0.025523678
137	0.74775529	0.426098908
138	-1.57491674	0.003556797
139	0.083573712	0.929524969

IDP	Beta	p-Value	LM3_INSOMNIA_SYMPTOMS_Summary
1	-31.550766	0.150180744	
2	-42.0392766	0.085735465	
3	8.940899692	0.13931801	
4	8.89778646	0.142687971	
5	1.605755982	0.930635407	
6	2.690960061	0.874161103	
7	-4.17596437	0.817577419	
8	-9.31650076	0.589584129	
9	-0.72159856	0.914921982	
10	-0.22046828	0.969816765	
11	-0.58741053	0.922648162	
12	9.478174857	0.117790072	
13	-45.7843837	0.008021608	
14	-32.9439988	0.055376715	
15	4.483503644	0.721377942	
16	10.97184134	0.375182734	
17	-1.92838408	0.557316912	
18	-3.61801973	0.279587805	
19	-11.4549969	0.029889044	
20	-3.93928282	0.486941916	
21	-0.92886634	0.825099749	
22	4.181326115	0.227558313	
23	5.029563037	0.570507556	
24	5.920196782	0.500845015	
25	-1.45605077	0.867123243	
26	-17.8242496	0.077141316	
27	2.39175867	0.500107058	
28	0.718166551	0.824061293	
29	14.54714436	0.084544587	
30	5.930658125	0.465998077	
31	-5.93126517	0.377336164	
32	-7.19563144	0.371310201	
33	-8.70335937	0.575417006	
34	0.332367767	0.98257638	
35	-4.03900064	0.702661336	
36	-1.76845865	0.863985241	
37	1.982268484	0.789138198	
38	3.244216782	0.646172786	
39	-4.2000549	0.662032127	
40	-0.24290239	0.983401582	
41	1.835581152	0.838423821	
42	-14.4986651	0.243954247	
43	-2.12662932	0.923991139	
44	29.57590751	0.197998768	
45	9.504336976	0.457732953	
46	-8.23616425	0.540426249	
47	-0.05620957	0.993415518	
48	4.380800053	0.498656411	
49	-2.49106976	0.49134982	
50	3.362002475	0.364390404	
51	-8.65355653	0.17422206	
52	-8.89088674	0.162463694	
53	-9.03413878	0.016629369	
54	-6.4921259	0.057764958	
55	4.304236746	0.609165325	
56	1.347249504	0.874717778	
57	9.506968633	0.40480039	

58	-1.54797311	0.9027227
59	-3.39659643	0.607846431
60	3.289687678	0.634349352
61	5.960297475	0.63607068
62	6.551630165	0.620220257
63	4.471597779	0.312575235
64	1.9863643	0.689785311
65	-11.6779244	0.133589994
66	-9.61563273	0.18493392
67	4.924128453	0.27006713
68	7.08652901	0.136646283
69	-1.34004222	0.579526366
70	1.476844791	0.462337012
71	-1.03915564	0.899670647
72	-10.7583783	0.204637142
73	-0.06856064	0.979883785
74	0.762520545	0.758102291
75	-1.55375476	0.781605887
76	1.252864255	0.79280687
77	0.164856652	0.973771567
78	3.785739872	0.54011491
79	3.415924521	0.596809665
80	14.4523034	0.024161904
81	-1.35234486	0.629797774
82	2.344413097	0.385775542
83	-15.7233766	0.004713797
84	-2.41635459	0.677973198
85	-3.53445297	0.462467211
86	-6.1150161	0.169457215
87	3.231633871	0.143888926
88	3.335561701	0.135981076
89	-2.37627705	0.393969482
90	1.097189406	0.593515518
91	-4.58400879	0.358129025
92	-8.00393077	0.014360116
93	1.080191966	0.399954289
94	3.550198065	0.040451836
95	6.474683773	0.656500521
96	1.129091039	0.935735417
97	1.124045173	0.727684905
98	-1.05815176	0.752293523
99	-1.11829742	0.886370168
100	4.222200637	0.604189555
101	4.142259417	0.408230806
102	3.168258555	0.557039972
103	-0.94481891	0.043748284
104	-0.35072585	0.508860829
105	0.121953996	0.976450412
106	7.479261285	0.079966325
107	0.634929065	0.785193122
108	0.703507204	0.78168469
109	0.834831325	0.483487462
110	0.64402367	0.588309823
111	14.31544479	0.107017325
112	3.846194673	0.233844778
113	2.988661898	0.3907796
114	6.884671939	0.103061804
115	3.084954288	0.454712323

116	24.6582148	0.024083336
117	5.463199138	0.049770594
118	17.41668385	0.1046554
119	8.581942722	0.614244883
120	-0.03402044	0.074923078
121	16.09614684	0.378526452
122	-7.23046853	0.582703901
123	2.400512311	0.00931414
124	4.051547888	0.751372452
125	-2.34556654	0.747229477
126	0.534227649	0.103310405
127	-4.84154106	0.529758966
128	11.1941474	0.134188784
129	3.180722499	0.100236671
130	2.408526667	0.761899059
131	12.96785508	0.022008168
132	1.854567779	0.060453308
133	5.389504334	0.378338995
134	9.51457913	0.033987076
135	0.704676988	0.500932192
136	8.768681358	0.075028798
137	1.108008788	0.206318951
138	0.095265583	0.850121809
139	-0.84228349	0.339494887

IDP	Beta	p-Value	LM3_LATE_CHRONOTYPE_Summary
1	-39.241642	0.244060363	
2	1.650578309	0.964971741	
3	-8.39467057	0.366270113	
4	-5.70242372	0.540871153	
5	2.116760578	0.940462006	
6	-9.42221251	0.718133564	
7	46.90528609	0.091725539	
8	58.80799757	0.026670344	
9	7.728216994	0.456429308	
10	9.921922433	0.267691118	
11	22.05214293	0.017663783	
12	21.31181182	0.02207027	
13	15.56003361	0.557537459	
14	-1.76717916	0.946663209	
15	-27.9918942	0.147279243	
16	-13.6762383	0.471826736	
17	-6.29599796	0.212362058	
18	-2.9122032	0.571056017	
19	-9.26541719	0.252901053	
20	-7.40106079	0.395243913	
21	-13.0056677	0.04400936	
22	-8.45245246	0.112349382	
23	-13.7708656	0.31200178	
24	-5.1598235	0.702543054	
25	12.40053807	0.353668694	
26	-5.73202369	0.711389789	
27	-5.03456403	0.355533103	
28	-1.52667033	0.758364866	
29	-2.88546858	0.823763917	
30	4.520632448	0.717576376	
31	11.78896012	0.253399646	
32	16.20025442	0.190148479	
33	-62.7741859	0.008554381	
34	-51.6944813	0.027045314	
35	-9.89877502	0.542556125	
36	4.517851613	0.775755962	
37	-2.97653792	0.793796619	
38	-15.2030554	0.161416346	
39	-0.29011338	0.984320394	
40	-17.1006053	0.340410567	
41	-10.1352742	0.46364643	
42	3.533012071	0.853377684	
43	-29.8969082	0.382641108	
44	-7.12861557	0.839948837	
45	38.8943869	0.047931944	
46	3.205850432	0.876742975	
47	-7.33557989	0.483292255	
48	-3.36126873	0.73542813	
49	-9.34561792	0.092869675	
50	-11.3750709	0.045770522	
51	-4.87601204	0.618234248	
52	-13.5411657	0.166125084	
53	-13.8228339	0.017076255	
54	-8.21608733	0.118036382	
55	6.082396872	0.638164153	
56	-5.73602008	0.662150692	
57	31.55584068	0.071885166	

58	66.5775637	0.000623107
59	3.989341863	0.694834372
60	5.426899407	0.609556644
61	10.26601864	0.595754577
62	-6.20339521	0.760057064
63	7.603868086	0.263678536
64	10.77825754	0.158619761
65	-19.627837	0.100770014
66	-7.84197622	0.481585087
67	-13.0303065	0.057477464
68	-17.5134846	0.016655293
69	-8.82930936	0.017492658
70	-5.28118871	0.087118622
71	-4.54584427	0.719597524
72	-2.96384492	0.820065321
73	-15.4086711	0.000225821
74	0.425014565	0.911034207
75	-9.87698942	0.25134924
76	-8.64787909	0.237951321
77	-0.28770789	0.970206979
78	4.75536686	0.61643576
79	-1.94700651	0.844407216
80	-0.74051657	0.940059036
81	-0.2729906	0.949500414
82	-2.85576961	0.491659841
83	-8.79321366	0.303595611
84	-10.7118705	0.230860573
85	-7.20725884	0.329421044
86	-7.37884385	0.28051886
87	6.422194348	0.058698773
88	-1.77011549	0.606546948
89	-1.03233412	0.809515456
90	-0.64124574	0.839097037
91	-3.09777384	0.686055321
92	-3.33942537	0.506130434
93	1.421588849	0.470892292
94	2.409245428	0.365382847
95	-15.8802736	0.477688378
96	-10.5646872	0.62337281
97	-5.38686566	0.277394966
98	-2.59846584	0.613920772
99	18.05215785	0.133237751
100	22.12510928	0.077042392
101	9.315958627	0.226029523
102	4.935802486	0.551515306
103	0.3194497	0.657190212
104	0.700456498	0.390465254
105	-11.2329453	0.076767815
106	-6.52965128	0.319745903
107	-2.94255446	0.410965031
108	-0.24580814	0.949745525
109	-2.88767168	0.114657365
110	-0.79939076	0.6618753
111	-17.7098499	0.194334141
112	-8.87505302	0.073762589
113	-10.9038119	0.041551512
114	-5.72453432	0.377610162
115	-3.2414421	0.609146622

116	-9.37579679	0.576627109
117	-7.23255573	0.090905531
118	-1.61912839	0.92177915
119	-34.877003	0.182442332
120	-0.00339776	0.907827421
121	-33.9686267	0.226413636
122	-2.39075948	0.905867693
123	0.774893335	0.584797935
124	-0.870498	0.964657986
125	-2.4008605	0.82997807
126	-0.33001418	0.512452024
127	0.536517635	0.96384812
128	-6.73722853	0.557359977
129	-1.91046945	0.520459503
130	-14.9309873	0.221488181
131	-12.3776232	0.154761953
132	-2.43767493	0.108205726
133	-16.1618407	0.085516905
134	-13.3905583	0.052102563
135	-0.53843018	0.737831214
136	-11.2947257	0.13553203
137	-2.74215261	0.041776752
138	-0.54365914	0.482731078
139	-3.74984915	0.005644016

IDP	Beta	p-Value	LM3_LONG_SLEEP_DURATION_Summary
1	-118.758785	0.194566941	
2	-248.105833	0.015147692	
3	-3.32278329	0.895309851	
4	14.41563042	0.569486866	
5	-161.262566	0.03629185	
6	-88.8154058	0.210595227	
7	-31.8814171	0.67319465	
8	-6.80336127	0.924833085	
9	-23.213482	0.410440956	
10	-3.63872882	0.881102479	
11	-27.7740739	0.271519021	
12	5.666741767	0.822775542	
13	-87.7995424	0.223332452	
14	-123.539103	0.085293125	
15	-135.36481	0.009919671	
16	-118.054967	0.022297381	
17	17.76807885	0.195317137	
18	-13.902521	0.31969019	
19	-12.2793625	0.577152118	
20	-1.95501384	0.934144063	
21	-1.31712026	0.940173245	
22	-16.2204468	0.262232925	
23	-10.5887096	0.77483762	
24	30.94412654	0.399392066	
25	-11.1229254	0.759513909	
26	-71.4915012	0.089517209	
27	-12.1763821	0.410956184	
28	-11.7760314	0.382595906	
29	-6.27019263	0.858666729	
30	-20.3930676	0.54825133	
31	-37.111106	0.185855522	
32	-18.9015194	0.573800296	
33	-167.542864	0.009816548	
34	-51.9845209	0.413304137	
35	-103.762512	0.018840425	
36	-14.2667661	0.740653093	
37	52.50990616	0.089759832	
38	-4.97265073	0.866162738	
39	45.77952891	0.253832889	
40	1.553647382	0.97457494	
41	42.70567966	0.25586352	
42	-12.8063022	0.805301626	
43	-159.357074	0.086848948	
44	-162.989084	0.089311161	
45	-31.206131	0.559248422	
46	-27.2513513	0.62758954	
47	-24.6528226	0.386009698	
48	-6.21884834	0.818057933	
49	-31.3448425	0.038093761	
50	-35.3238514	0.022464835	
51	-16.0504725	0.546101358	
52	-15.7099871	0.554420228	
53	15.44626384	0.326738068	
54	23.79179158	0.095819405	
55	-80.6231509	0.02181569	
56	-40.7783895	0.253044903	
57	67.64661905	0.155689387	

58	21.34850037	0.686424334
59	73.31403919	0.007985934
60	118.230177	4.25E-05
61	-5.3111319	0.919557886
62	-99.3271832	0.071971653
63	-15.7974044	0.392851654
64	-34.0320745	0.101455956
65	-52.3463775	0.107293223
66	-71.5283497	0.018183266
67	-44.2520408	0.017605696
68	-28.8707931	0.146413863
69	-8.46090109	0.402096262
70	-14.9396139	0.074953609
71	17.83549732	0.604268388
72	-24.8924869	0.48209602
73	-23.8953274	0.035316381
74	-11.6725918	0.258844654
75	-12.7799505	0.584974757
76	-6.26972443	0.752895487
77	-6.10609939	0.770542712
78	-4.34076926	0.866391607
79	-37.2349054	0.167268703
80	-40.8326168	0.127097134
81	-25.0481454	0.032500569
82	-5.63605542	0.61751196
83	-14.2369154	0.53995246
84	1.323532512	0.956558398
85	9.100110263	0.650467952
86	11.00499515	0.553701541
87	11.84865305	0.19936184
88	8.405886562	0.368176101
89	-6.48071641	0.577656112
90	7.1051949	0.407760923
91	9.418421874	0.651120176
92	0.26399713	0.984569533
93	9.884341856	0.065088672
94	5.056804823	0.484512243
95	-27.8368148	0.646976614
96	-19.378303	0.74031196
97	15.60662551	0.24690025
98	21.59854921	0.122848081
99	138.3581453	2.30E-05
100	128.4040779	0.000159747
101	52.47341585	0.012105129
102	38.88195861	0.084337837
103	6.205067191	0.001515489
104	8.878009358	6.21E-05
105	-26.9660887	0.117986486
106	-16.9063102	0.343171238
107	-13.2370472	0.173535997
108	-18.4881225	0.081117449
109	5.875710698	0.237537352
110	1.741803921	0.725870029
111	-3.53879745	0.923975446
112	11.1839999	0.407034498
113	10.07200382	0.488495553
114	-5.02025809	0.775864868
115	-3.41490368	0.842887372

116	0.530690766	0.99072223
117	0.604909549	0.958504551
118	-1.5957004	0.971594956
119	-17.3305995	0.807402039
120	0.049001709	0.538956587
121	-29.5482798	0.698621582
122	60.52308623	0.270674913
123	-5.132638	0.182971541
124	72.28222778	0.175806399
125	7.843037921	0.796321209
126	-1.10688672	0.418862282
127	34.09838686	0.28917016
128	18.74823116	0.547962023
129	-6.43728131	0.425593408
130	13.50115692	0.684168324
131	19.40473883	0.411746742
132	2.646107782	0.521131898
133	11.77651417	0.644766417
134	25.05945533	0.181063687
135	4.243088733	0.331750488
136	25.68159023	0.211730764
137	-1.37502613	0.707197792
138	0.770252414	0.714422154
139	-0.11600052	0.974865661

IDP	Beta	p-Value	LM3_PSYCH_MEDICATION_Summary
1	30.32082581	0.469093338	
2	97.79813205	0.036367996	
3	-0.85043775	0.941313778	
4	-8.14034071	0.482612082	
5	37.44527942	0.287922497	
6	59.75173854	0.065613642	
7	57.65022865	0.095497148	
8	-9.36315158	0.776540352	
9	18.48133439	0.152013973	
10	-3.62290704	0.74478231	
11	8.637873425	0.454749814	
12	7.462743643	0.519084233	
13	-15.358842	0.641476575	
14	1.336890344	0.967530678	
15	-9.47022474	0.693315487	
16	-11.2568466	0.633833712	
17	0.55532823	0.929499646	
18	4.222999379	0.508785769	
19	-13.9539481	0.166072294	
20	-19.171362	0.076524565	
21	-1.29584711	0.871773176	
22	-0.68251507	0.91786844	
23	-24.5274956	0.147513181	
24	-1.62129783	0.92311175	
25	-13.321324	0.422902445	
26	15.27596744	0.427731665	
27	-14.2941927	0.034874876	
28	-3.01990865	0.62452808	
29	8.588830819	0.593906228	
30	31.59265795	0.042046836	
31	13.24653511	0.301970894	
32	17.57281453	0.253017279	
33	4.990925958	0.866464022	
34	-6.92082535	0.811820919	
35	20.68990142	0.305963115	
36	-5.71641995	0.771896042	
37	19.79687994	0.162037934	
38	-10.5709844	0.433539358	
39	26.88916729	0.142906912	
40	-22.1537856	0.320522184	
41	1.258461216	0.941654923	
42	-28.6615514	0.227864123	
43	-52.7398661	0.215450712	
44	40.60321617	0.354853895	
45	13.15913229	0.5903914	
46	-15.3759031	0.549626415	
47	18.34424373	0.158542294	
48	17.34049407	0.160879243	
49	0.936447138	0.892266368	
50	-10.0290241	0.156620403	
51	-5.12537435	0.673511154	
52	8.185650709	0.500760569	
53	-13.0371505	0.070398134	
54	-9.85189758	0.131679512	
55	-16.5021368	0.304794461	
56	1.741689052	0.915017071	
57	-13.2805991	0.542345776	

58	-19.6777729	0.415983667
59	5.569030359	0.659588905
60	12.70429328	0.336249855
61	31.06012159	0.196708197
62	14.27485516	0.571898854
63	15.49992089	0.066869668
64	12.49662691	0.18862986
65	-13.3749835	0.368390253
66	-0.39434287	0.977291917
67	15.70342808	0.065565451
68	8.428636479	0.354021841
69	3.382795941	0.464001682
70	5.18838678	0.176421934
71	12.31100515	0.434226903
72	25.66022035	0.113213806
73	-9.45001037	0.068839261
74	-4.11600803	0.38412105
75	20.9677934	0.050162573
76	9.73854092	0.285111983
77	14.09367366	0.141148829
78	-1.9043625	0.871822817
79	-4.31605739	0.72639377
80	-2.92118883	0.811426742
81	-4.08246582	0.446182269
82	-15.1058552	0.003438751
83	-19.2649391	0.069861826
84	-12.7574278	0.251085454
85	2.463549794	0.788596635
86	11.07163703	0.192791743
87	-0.33045443	0.937636909
88	-2.70254274	0.527106102
89	-2.10449469	0.692663897
90	-4.17065384	0.288148187
91	-0.01046585	0.999123597
92	-2.30236877	0.712352473
93	7.647205749	0.001811887
94	3.9991915	0.226862524
95	1.958059998	0.943862636
96	-10.9750273	0.681569635
97	20.67171074	0.000801484
98	22.8285068	0.00036459
99	59.69940109	6.51E-05
100	56.23432082	0.000301198
101	29.3152326	0.002184139
102	24.78220509	0.016184274
103	0.856099436	0.338769835
104	0.9820306	0.332850792
105	-4.7592408	0.546438202
106	-12.8421696	0.115503809
107	-8.90855095	0.045279528
108	-7.94642936	0.101262449
109	2.426065621	0.286394537
110	2.79014124	0.219556916
111	16.85438628	0.320478847
112	-13.6450557	0.027027016
113	-17.9104874	0.007093004
114	-8.04768263	0.318449904
115	-10.4482542	0.184988132

116	-14.9746845	0.473232813
117	-8.1263977	0.126544823
118	-19.256043	0.347592592
119	-33.7280433	0.299711705
120	0.026265282	0.471613598
121	-44.2479155	0.205025644
122	-69.0375359	0.006025539
123	-2.92073957	0.097634969
124	-57.6432033	0.018282774
125	-42.0610103	0.002481923
126	-0.7259807	0.246466235
127	-36.7905909	0.012427583
128	-40.6321667	0.004425245
129	-4.82185057	0.192044255
130	-51.9214608	0.000627959
131	-19.4954667	0.071452384
132	-1.67255893	0.375359791
133	-34.1681215	0.003457662
134	-13.4820349	0.115741874
135	-0.76848461	0.700786987
136	-23.6627728	0.011899281
137	-1.76699691	0.291369224
138	-1.85998586	0.053426468
139	0.380147121	0.821439067

IDP	Beta	p-Value	LM3_SEX_Summary
1	-281.117758	5.43E-31	
2	-313.636482	5.47E-31	
3	-22.9689664	0.000598258	
4	8.127680875	0.226184128	
5	-333.266529	1.05E-59	
6	-216.076709	1.61E-30	
7	-390.659619	3.01E-84	
8	-352.487194	1.30E-75	
9	-64.4325549	6.86E-18	
10	-25.2321672	9.09E-05	
11	-71.2635384	1.98E-26	
12	-79.5189966	2.19E-32	
13	-177.576436	1.57E-20	
14	-159.174485	6.12E-17	
15	308.1356158	5.91E-108	
16	191.0301935	3.86E-44	
17	9.271524193	0.010771014	
18	-15.5472911	2.68E-05	
19	-23.4642136	5.82E-05	
20	-63.7975654	2.75E-24	
21	50.40370971	2.50E-27	
22	6.950193719	0.069854485	
23	-143.199567	4.00E-48	
24	-226.832106	2.99E-119	
25	-131.85859	1.42E-42	
26	56.57468524	3.98E-07	
27	60.85883043	4.69E-54	
28	56.15340879	1.97E-55	
29	54.79140197	4.34E-09	
30	14.24685602	0.113457917	
31	54.16444799	3.24E-13	
32	161.4294636	4.26E-73	
33	-559.580927	8.39E-229	
34	-619.820393	8.30E-291	
35	-169.381397	2.63E-47	
36	-128.561957	2.44E-29	
37	-111.09632	1.06E-41	
38	-157.968737	3.09E-90	
39	-63.2878133	2.65E-09	
40	-61.0841438	2.27E-06	
41	-113.286837	6.34E-30	
42	-93.0483475	1.41E-11	
43	-966.111447	0	
44	-466.388638	8.02E-75	
45	-127.617604	2.12E-19	
46	71.15139695	1.76E-06	
47	-37.1251911	8.41E-07	
48	-43.2589599	1.57E-09	
49	5.388172531	0.178508322	
50	1.910705369	0.641269118	
51	-55.9840766	2.00E-15	
52	-46.2066492	5.40E-11	
53	-20.2571247	1.22E-06	
54	9.956019075	0.008537703	
55	-106.187489	4.73E-30	
56	-145.42076	3.27E-53	
57	-177.991546	5.29E-45	

58	-140.228689	1.53E-23
59	54.42150629	1.10E-13
60	22.97069884	0.002686312
61	-267.123358	1.86E-81
62	-326.789774	9.52E-110
63	-60.3452629	8.65E-35
64	-50.6074941	4.09E-20
65	33.8071315	8.68E-05
66	44.6125264	2.73E-08
67	123.4975259	1.06E-136
68	154.4348573	1.43E-186
69	15.81245497	3.46E-09
70	0.318031329	0.886241606
71	226.4554491	6.57E-135
72	199.6944772	7.83E-100
73	47.9408604	5.78E-57
74	71.14338216	3.04E-147
75	56.46107913	9.05E-20
76	47.10547295	4.62E-19
77	-12.7413017	0.021638094
78	56.23601946	2.01E-16
79	-0.61815404	0.931049921
80	-13.3144696	0.060469051
81	-13.6032028	1.18E-05
82	-5.855449	0.050245396
83	-72.6414044	4.45E-32
84	-129.187786	4.97E-89
85	53.20965388	1.67E-23
86	43.72234118	7.05E-19
87	-61.1881911	8.40E-137
88	-59.1242841	4.72E-125
89	2.519970501	0.413888595
90	-20.6550239	1.12E-19
91	131.603734	1.24E-124
92	45.98149158	6.14E-37
93	-9.36925116	4.21E-11
94	-17.384107	1.26E-19
95	-66.578078	3.58E-05
96	246.5931184	7.86E-57
97	18.04877077	4.36E-07
98	43.68755314	5.87E-32
99	-136.426121	9.81E-56
100	-120.309426	1.49E-40
101	52.50703734	2.83E-21
102	55.74636851	1.03E-20
103	-3.50566723	1.38E-11
104	-2.2406785	0.000136598
105	29.72479204	7.97E-11
106	27.00037819	1.12E-08
107	11.14950729	1.52E-05
108	-12.8148201	5.07E-06
109	-2.84077572	0.031158493
110	4.613370701	0.000457809
111	-70.6220417	6.77E-13
112	9.434364222	0.008306993
113	6.679052107	0.083011192
114	35.3804488	3.76E-14
115	35.3529297	9.95E-15

116	-133.723176	2.26E-28
117	-35.9062195	2.48E-31
118	-106.506633	3.14E-19
119	-237.545399	2.25E-36
120	-0.10407382	8.50E-07
121	-101.298251	5.49E-07
122	-79.6988696	4.43E-08
123	-9.06771733	7.11E-19
124	-90.6988184	1.47E-10
125	-54.1934002	1.72E-11
126	-8.01527597	2.25E-107
127	-44.0346041	2.41E-07
128	-115.52147	3.12E-44
129	-35.8456863	1.14E-62
130	-170.536689	2.67E-83
131	-56.7994849	1.29E-19
132	-17.4998216	1.70E-57
133	-124.731968	1.84E-75
134	-144.505255	5.98E-184
135	-21.2226604	1.32E-74
136	-161.837796	2.46E-191
137	8.241053356	2.05E-17
138	-19.5156613	2.00E-263
139	2.65363535	0.006532264

IDP	Beta	p-Value	LM3_SHORT_SLEEP_DURATION_Summary
1	-5.12813726	0.830292143	
2	-80.8256507	0.002466468	
3	-3.67766575	0.577346282	
4	-3.31024742	0.617239428	
5	4.558086443	0.820864326	
6	-1.91685011	0.91765694	
7	-55.9955593	0.0045933	
8	-9.86639754	0.600607003	
9	1.051178496	0.886591629	
10	-3.1115295	0.624571173	
11	-2.51949451	0.702714269	
12	-6.57217853	0.320262964	
13	-4.09895141	0.827800601	
14	0.904817345	0.961537733	
15	-12.5139507	0.361686292	
16	-26.5940976	0.048864172	
17	1.060622003	0.767395882	
18	-6.13238985	0.093062463	
19	16.91235191	0.003302823	
20	9.102761166	0.140991315	
21	-7.06289582	0.123589006	
22	-13.719253	0.000285698	
23	-7.25166152	0.453511639	
24	1.912935711	0.84200337	
25	-4.22381354	0.656474595	
26	6.740114939	0.540193193	
27	-7.57667936	0.050284784	
28	-3.20955995	0.362540232	
29	-25.2951619	0.00598617	
30	-9.00216022	0.310552619	
31	-14.5045709	0.047886295	
32	-9.86376291	0.261401981	
33	-4.51120232	0.790203686	
34	13.70470001	0.409243156	
35	-8.3892581	0.467469476	
36	2.470829858	0.82639006	
37	-0.37389415	0.963129968	
38	-3.88167846	0.614698026	
39	20.53943369	0.050129204	
40	13.72199223	0.281462949	
41	21.735385	0.02692336	
42	21.98929797	0.105359304	
43	-11.5991698	0.633447831	
44	-24.8784089	0.321043958	
45	-50.9466468	0.000264849	
46	-46.5050299	0.001537958	
47	-5.91071851	0.426465425	
48	-8.14589597	0.248931706	
49	3.234385784	0.412888861	
50	1.147598625	0.776615784	
51	2.749118261	0.692411488	
52	7.35042966	0.289921022	
53	-10.3092945	0.012266327	
54	-9.27895965	0.012945581	
55	-7.81092465	0.395190252	
56	-2.55636759	0.783957514	
57	-11.5251328	0.354700714	

58	-13.2608619	0.33730268
59	-7.30488008	0.311847566
60	-14.1417267	0.060981626
61	-42.2951256	0.002090902
62	-40.7935764	0.00469288
63	-13.6450559	0.004746051
64	-13.9754299	0.010070141
65	-10.7989482	0.203642649
66	1.706483689	0.829290574
67	-15.2669066	0.001727562
68	-17.2784419	0.000882705
69	-1.33312798	0.613457732
70	-2.35887983	0.281995759
71	2.181504453	0.808352666
72	-1.20117048	0.896735639
73	-6.96179331	0.018963935
74	-6.16693196	0.022461187
75	-0.24633259	0.967871576
76	-3.7934684	0.466102788
77	-1.12638749	0.83689601
78	-4.20680452	0.532709215
79	-0.14371082	0.983728241
80	-8.37535225	0.231162176
81	-4.33183761	0.157095566
82	-1.82132202	0.536923848
83	0.69554791	0.908786947
84	-5.34955933	0.399544343
85	1.995130542	0.703866185
86	7.486886289	0.123176859
87	4.878748264	0.04318704
88	0.549279118	0.82198075
89	3.5289135	0.246005401
90	3.705137175	0.098586403
91	5.79571758	0.28699993
92	4.16505349	0.243007331
93	-2.83936309	0.042611969
94	-4.20094451	0.026281959
95	-18.9397587	0.23317168
96	-34.0097027	0.026039469
97	-0.15625021	0.964619509
98	-3.60475294	0.324468
99	17.85613737	0.036535795
100	10.40664678	0.241646694
101	11.71737116	0.032049333
102	15.28724831	0.009416294
103	0.330936918	0.517445783
104	0.054335348	0.925276303
105	1.768985299	0.694764847
106	2.42000251	0.60363792
107	3.559609851	0.161423121
108	1.137150217	0.681437722
109	3.233567432	0.012879327
110	2.379636557	0.066830411
111	25.42879037	0.008700975
112	11.33495878	0.001304291
113	11.99048238	0.001604462
114	12.11367278	0.008576211
115	15.73791455	0.000474578

116	25.09015664	0.035424966
117	3.378329024	0.266210842
118	24.05194022	0.040018404
119	8.10321997	0.662743303
120	0.013322163	0.52274368
121	1.383472664	0.944700561
122	8.629948174	0.547859637
123	0.595151856	0.554636191
124	11.18187994	0.422941699
125	11.6118173	0.143701266
126	0.74655461	0.036964914
127	12.69998318	0.130913575
128	12.78300477	0.117018699
129	2.510093061	0.234544574
130	16.36327651	0.059246445
131	4.389993793	0.477376513
132	0.390783853	0.716939525
133	11.00510092	0.099251431
134	11.62691109	0.017579875
135	2.333042498	0.041157665
136	12.96998563	0.015816678
137	-1.31490583	0.169326318
138	2.730512793	6.96E-07
139	-0.05194531	0.95694835

IDP	Beta	p-Value	LM3_SLEEP_APNOEA_Summary
1	-175.957236	0.253733243	
2	-87.0255154	0.612905805	
3	23.59180697	0.579027114	
4	30.42690452	0.475881552	
5	-56.0406297	0.665692681	
6	-117.552234	0.325120904	
7	-160.357678	0.207757518	
8	2.955797234	0.980580214	
9	38.23874904	0.420717863	
10	22.34093161	0.585520973	
11	-35.2627699	0.407090495	
12	3.131311254	0.941411781	
13	-3.757929	0.975308759	
14	-123.063861	0.308708948	
15	-88.1186518	0.318833924	
16	-53.4553985	0.538882145	
17	10.28364731	0.656253237	
18	-13.6503273	0.561776259	
19	-17.7826242	0.631598222	
20	-41.8692154	0.293310699	
21	12.56384278	0.670739601	
22	-19.0240511	0.434895122	
23	3.677843004	0.952950998	
24	10.87448822	0.860401345	
25	20.36284973	0.73928975	
26	-31.2596699	0.659293439	
27	-18.3481124	0.461890376	
28	-27.0418464	0.233799381	
29	-5.18242409	0.930353415	
30	-16.2357224	0.776528011	
31	-29.530744	0.531876218	
32	31.67727874	0.575635106	
33	-120.807764	0.268846461	
34	9.394780506	0.930037502	
35	-55.6277394	0.454612184	
36	-99.2042222	0.171715135	
37	30.84157935	0.553993166	
38	-115.578243	0.020014131	
39	-11.741972	0.862019767	
40	-178.053921	0.030088296	
41	-0.97888883	0.98766033	
42	9.71474445	0.911585401	
43	126.8732516	0.418201576	
44	221.2117419	0.170881729	
45	-25.9113547	0.77339452	
46	-73.1756548	0.439190785	
47	-34.2581875	0.474390067	
48	-68.0194208	0.135138883	
49	-53.3694283	0.036006642	
50	-24.3428662	0.350269375	
51	-8.52797316	0.848955903	
52	-44.4579677	0.320503424	
53	9.229404933	0.727853525	
54	2.140414544	0.929099561	
55	38.57762722	0.514575615	
56	29.52349264	0.623133982	
57	90.26750069	0.26058541	

58	141.1918191	0.112848034
59	-8.19158127	0.860281679
60	2.062777725	0.966166621
61	-53.4625064	0.546055512
62	49.83768184	0.591870669
63	33.53862962	0.28137127
64	23.50607592	0.501716078
65	11.60353844	0.832108181
66	18.34348366	0.719070551
67	-18.9784136	0.54545704
68	-16.6838042	0.618209454
69	-18.0570469	0.28828756
70	-22.6259406	0.109257785
71	-6.70112861	0.907942311
72	-28.7156763	0.630132607
73	9.11511817	0.633516284
74	30.14398708	0.083353201
75	57.49272446	0.144577915
76	-9.87497896	0.768409288
77	-65.2423071	0.064232039
78	23.68576247	0.585643464
79	-92.8672382	0.040817145
80	-62.5617682	0.165103954
81	-9.59524655	0.626671805
82	-9.03428665	0.634536308
83	-13.1610408	0.736532074
84	5.710700486	0.888997539
85	-58.0531262	0.086072362
86	-61.3392407	0.049979116
87	-1.70203573	0.91282413
88	5.876038491	0.708731486
89	-19.3064261	0.324608076
90	-2.99686326	0.835736113
91	-57.1136111	0.103444209
92	-31.178625	0.1749763
93	5.549697284	0.538521557
94	1.178375239	0.922935497
95	-28.9348131	0.777419356
96	60.90247856	0.536199405
97	-5.29981396	0.815369104
98	6.161290378	0.793802417
99	-6.88464763	0.900426643
100	-19.645426	0.731560508
101	-0.13534942	0.996933422
102	-35.4945633	0.349423618
103	6.401775303	0.051984059
104	-0.27004745	0.942328106
105	-8.53301063	0.768940283
106	-30.354444	0.312174455
107	-23.7737872	0.146649494
108	-36.0343206	0.043508801
109	1.963777647	0.814651573
110	-4.29732678	0.607464262
111	1.611458831	0.979412979
112	-4.97249895	0.82672062
113	-29.5972302	0.226749795
114	-5.00640076	0.866107207
115	-9.2211547	0.750622578

116	-106.779308	0.164720046
117	8.71334034	0.656278692
118	-22.2498095	0.768115599
119	-138.148766	0.248516314
120	-0.02097576	0.875893146
121	-132.746486	0.30163945
122	-75.7180313	0.413162984
123	4.872157491	0.452856635
124	-54.8587731	0.54176569
125	-72.6211269	0.155839903
126	-1.50339886	0.514387386
127	-119.904144	0.026878315
128	-62.2274179	0.23633322
129	-8.71649844	0.52174077
130	-112.057365	0.044978128
131	-66.0324016	0.097183907
132	3.038598755	0.661734561
133	-93.8849047	0.029065736
134	-25.0114237	0.427934703
135	0.346456302	0.962463625
136	-10.36922	0.764612403
137	-3.46455739	0.57410129
138	6.728083965	0.057692234
139	-11.3067662	0.068210751

IDP	Beta	p-Value	LM3_SLEEP_MEDICATION_Summary
1	10.90215857	0.953614456	
2	182.6225607	0.382499232	
3	35.57809878	0.491311933	
4	46.70669608	0.368010664	
5	-25.123682	0.873408427	
6	-125.460042	0.387676444	
7	-16.1949407	0.916651815	
8	15.72989434	0.915143626	
9	-32.5755229	0.572605681	
10	2.214125257	0.964540553	
11	-12.4809372	0.809275196	
12	-127.560579	0.013792302	
13	222.5399347	0.131652273	
14	-106.47781	0.468780332	
15	-19.7812441	0.853956571	
16	-10.0576001	0.924232688	
17	-37.9692126	0.176455547	
18	4.286320769	0.880873521	
19	-59.6635224	0.185749662	
20	-5.85579172	0.903772211	
21	47.98400633	0.181695579	
22	-29.0624776	0.326492355	
23	-59.5886058	0.431681976	
24	-16.3895523	0.827411862	
25	-25.6080929	0.730653843	
26	-84.0914189	0.329274554	
27	29.54551158	0.329800415	
28	13.03781861	0.636791772	
29	28.38722657	0.693733117	
30	41.52238008	0.550428321	
31	98.22845528	0.087188589	
32	51.04496187	0.45810955	
33	-66.6837513	0.615636521	
34	95.06273463	0.464928545	
35	-236.331126	0.008976374	
36	-196.676815	0.025831833	
37	101.3172279	0.1098012	
38	155.2903195	0.01014839	
39	145.2113685	0.077065431	
40	107.9419889	0.279435124	
41	-65.0486993	0.397899854	
42	-71.0842484	0.50393038	
43	88.74075618	0.641386822	
44	-178.594259	0.363141532	
45	90.28006448	0.409251459	
46	-104.746645	0.362388428	
47	-68.0576666	0.242421162	
48	-87.9506549	0.1120274	
49	19.12393073	0.536533816	
50	8.341431576	0.792335331	
51	-24.6399023	0.650815767	
52	-60.0369379	0.269812708	
53	-4.7150862	0.883738749	
54	-3.45274153	0.906017494	
55	-22.3177646	0.756458236	
56	6.149375188	0.932902621	
57	39.28495564	0.687141763	

58	62.83173519	0.561655389
59	104.5475025	0.06463232
60	106.8152268	0.070817647
61	-74.8316249	0.487029605
62	-76.0254588	0.501124122
63	-48.343096	0.201521289
64	6.14596233	0.885120006
65	-108.32777	0.103535607
66	-1.23775698	0.98407152
67	34.03076887	0.372538563
68	35.79185688	0.379143661
69	9.315427177	0.652268257
70	24.51838486	0.153421356
71	-125.685534	0.074432778
72	-66.5590872	0.358570852
73	-8.58734223	0.711773623
74	15.21169916	0.47228456
75	-23.099277	0.629685095
76	31.51075563	0.439596693
77	-41.2860968	0.335404987
78	73.93882934	0.161568403
79	41.76487071	0.449252167
80	-75.2621776	0.169567595
81	4.965694699	0.835961549
82	-17.5879108	0.446534785
83	-95.3563377	0.044953644
84	-57.9201418	0.244260168
85	41.58213379	0.311855115
86	-70.4923674	0.063891847
87	-25.8664102	0.171148849
88	-11.4348885	0.549864469
89	-23.3582588	0.32694097
90	-25.769228	0.142495674
91	14.27144043	0.73784916
92	-43.5370781	0.119247256
93	-7.49699129	0.494334026
94	-5.71409091	0.699605777
95	-24.2682449	0.845374992
96	150.0826221	0.209894109
97	0.337853081	0.990230767
98	21.77053275	0.447447727
99	7.096228136	0.915516042
100	23.83379501	0.732095461
101	18.9858524	0.657432473
102	45.75168048	0.321152223
103	0.334591931	0.933417245
104	5.301280776	0.242737883
105	16.51696837	0.639982141
106	13.10950861	0.719564722
107	-0.21344086	0.991447511
108	-2.03765877	0.92518488
109	1.634826503	0.872462816
110	10.59791665	0.297383375
111	-23.8541756	0.753365007
112	-10.2225086	0.711247643
113	7.89974738	0.790711721
114	-43.3440181	0.229864865
115	-22.4500444	0.524470701

116	-119.781833	0.199840478
117	-12.0463073	0.612773867
118	-125.273625	0.172105407
119	-65.7075217	0.651650069
120	0.039326	0.809670972
121	-215.744901	0.167321334
122	-163.567474	0.145918858
123	-19.4889155	0.013519368
124	-123.411232	0.258884633
125	-112.966638	0.069383945
126	-6.02933943	0.031487756
127	-80.0342591	0.224280474
128	-127.508231	0.045943742
129	-16.4881991	0.318840432
130	-106.946606	0.115505959
131	-56.8246667	0.24035033
132	-4.28607387	0.611709971
133	-71.6185036	0.170826338
134	-17.1325804	0.655119378
135	-2.75890204	0.757879051
136	-12.6887235	0.763114638
137	-6.67382814	0.373184271
138	-3.85260742	0.371316334
139	-2.54105156	0.736026068

IDP	Beta	p-Value	LM3_SOCIOECONOMIC_STATUS_Summary
1	-45.0634417	0.013002162	
2	-17.3904255	0.390287981	
3	8.044745822	0.107926059	
4	6.285405609	0.210768517	
5	9.48664523	0.534263282	
6	22.63380846	0.107418332	
7	12.01386672	0.422556237	
8	-7.39988713	0.604578135	
9	4.94019634	0.376731642	
10	3.961211475	0.41128324	
11	-7.34460895	0.1423084	
12	0.583648844	0.907330796	
13	-35.6431025	0.012616696	
14	-29.8809058	0.035711086	
15	-2.47492578	0.811948175	
16	-23.9021869	0.019555662	
17	2.155392399	0.427944543	
18	3.457102367	0.211798448	
19	11.77162963	0.006997393	
20	9.439180086	0.044099631	
21	-3.38338763	0.330633724	
22	0.882516418	0.758231737	
23	11.39049291	0.120490495	
24	-0.26691398	0.970740337	
25	2.928415484	0.684239212	
26	-4.21904309	0.613104523	
27	0.911692604	0.756067276	
28	-0.3255951	0.903043198	
29	3.133781862	0.653364459	
30	-3.25988266	0.628182636	
31	4.258479901	0.443655227	
32	-0.93395026	0.888469144	
33	-2.03008151	0.874542596	
34	23.84962838	0.058239413	
35	7.276562325	0.40588986	
36	-4.93070693	0.563782777	
37	-5.06194452	0.409177353	
38	11.3779519	0.051673852	
39	-1.23485154	0.876570706	
40	-5.30082692	0.58320695	
41	10.60552802	0.154492039	
42	-11.1520808	0.278745573	
43	-12.884589	0.484799572	
44	-8.33755046	0.660966939	
45	-3.22951674	0.760401048	
46	-21.1919636	0.056961081	
47	-13.5804419	0.015970639	
48	-15.181867	0.004601268	
49	-5.58290098	0.062333042	
50	-2.46934073	0.420733032	
51	-6.82561339	0.195225213	
52	-1.14988005	0.827165721	
53	-6.88881527	0.027314427	
54	-5.55110353	0.04989914	
55	-9.55084872	0.170354712	
56	-8.6543471	0.220934102	
57	-2.5309027	0.78867186	

58	-4.30593137	0.681152876
59	-8.14746309	0.136855127
60	5.473429847	0.338882458
61	-22.4902455	0.030937073
62	-39.0898556	0.000353034
63	-3.31152671	0.366085267
64	-10.9153869	0.008032408
65	-13.0452717	0.042845271
66	-4.94058624	0.410372356
67	1.836657459	0.619063114
68	6.181629157	0.116618014
69	-2.12654326	0.28793639
70	-0.3378024	0.838990875
71	-9.46891899	0.165008024
72	-6.18123856	0.378427402
73	1.573912151	0.484199664
74	4.150839517	0.042756745
75	-2.82476739	0.542442147
76	-0.98866875	0.802193296
77	5.960713079	0.150802652
78	-8.38329014	0.10109285
79	-3.13158565	0.557802895
80	-18.8943319	0.000367973
81	-1.52674752	0.510750513
82	1.744591596	0.435382441
83	-0.31766278	0.944985085
84	2.953409452	0.539631526
85	-3.39892198	0.393110312
86	1.726029038	0.639281706
87	4.906406359	0.007328538
88	3.492827462	0.059184281
89	0.524070267	0.820259383
90	3.911876238	0.021458043
91	3.47531256	0.399795268
92	7.023032176	0.009428441
93	0.252453298	0.812078522
94	-3.13691167	0.028656427
95	-42.0244215	0.000485848
96	-21.8161557	0.059723504
97	0.060994079	0.981781399
98	1.888360176	0.496050429
99	17.77071884	0.00606504
100	19.06186832	0.00467962
101	12.79479536	0.002021242
102	9.727518952	0.029331838
103	0.348349199	0.368889249
104	0.590968389	0.178537514
105	-0.07719641	0.981983032
106	-2.76580032	0.433906276
107	-0.17706404	0.926807793
108	-4.69921902	0.02527932
109	-1.09278358	0.267642183
110	-1.35974466	0.167224859
111	-30.1289153	4.15E-05
112	-6.96268274	0.009199411
113	-5.61063453	0.051523324
114	-7.44353233	0.033163943
115	-3.57649961	0.294887159

116	-16.6982581	0.06485854
117	-2.71153481	0.239244731
118	-15.5927138	0.079131647
119	-4.07248741	0.772531056
120	-0.00193845	0.902389545
121	-14.4956147	0.337833823
122	-2.72823826	0.802156124
123	-0.15644166	0.837715266
124	-7.51997793	0.477264364
125	-11.7303748	0.051421691
126	-0.50492357	0.062776953
127	-18.1760119	0.004359819
128	-16.562746	0.00740145
129	-5.76010293	0.000321638
130	-24.6823274	0.000175292
131	-17.6991294	0.000158413
132	-4.20147963	2.75E-07
133	-20.9668235	3.45E-05
134	-17.2079523	3.59E-06
135	-2.63113393	0.002390503
136	-16.9839483	3.09E-05
137	-1.01639453	0.161201595
138	-0.50684837	0.224356444
139	-1.7145676	0.018783652

IDP	Beta	p-Value	NewOSA_LM3_AGE_Summary
1	-58.9488583	0	
2	-57.0368301	5.95E-263	
3	-3.83764638	1.87E-21	
4	-2.45410409	1.33E-09	
5	-19.5604215	8.00E-57	
6	-13.5439429	1.13E-32	
7	-12.7820415	1.77E-26	
8	-10.0516794	3.66E-18	
9	-10.4575836	5.52E-117	
10	-8.78560296	2.29E-111	
11	-11.2723419	1.60E-168	
12	-9.07658	2.01E-109	
13	-47.7967212	0	
14	-48.5688457	0	
15	-24.5756642	4.93E-184	
16	-22.8551486	9.67E-165	
17	-4.3170972	7.88E-85	
18	-4.21309774	7.91E-79	
19	-8.8423698	1.21E-136	
20	-10.8998536	7.21E-180	
21	-4.69346087	5.15E-62	
22	-2.87547718	3.50E-35	
23	-13.2586457	1.08E-109	
24	-15.132339	3.67E-144	
25	-2.58985436	9.33E-06	
26	-5.17652241	1.86E-14	
27	-4.01379121	3.05E-64	
28	-2.65960347	8.46E-35	
29	-5.38399303	1.07E-21	
30	-7.43466451	2.83E-42	
31	0.872820531	0.052175116	
32	-1.74677088	0.001179143	
33	-40.772728	0	
34	-40.0187442	0	
35	-14.411827	1.55E-91	
36	-11.300172	1.97E-59	
37	-2.88139287	7.38E-09	
38	-2.73374526	8.52E-09	
39	-5.60159968	3.29E-18	
40	-17.1816355	1.10E-105	
41	-2.70084809	7.29E-06	
42	-12.9964196	2.19E-54	
43	-39.0851896	1.85E-150	
44	-46.4433117	1.06E-198	
45	-10.894709	6.60E-37	
46	-14.4042809	1.99E-57	
47	-3.67427023	7.62E-16	
48	-4.45086364	1.01E-24	
49	-7.16287143	6.20E-189	
50	-7.05105399	2.57E-174	
51	-7.41775043	1.09E-67	
52	-9.26004711	4.16E-104	
53	-2.92583146	3.05E-31	
54	1.711261962	6.08E-14	
55	-22.4893765	0	
56	-26.2618974	0	
57	15.40008453	3.31E-90	

58	20.27856325	6.27E-125
59	2.025910952	4.78E-06
60	4.951141472	1.08E-26
61	-20.1220248	3.47E-125
62	-26.1598881	1.95E-189
63	-6.64412122	4.23E-110
64	-7.65655655	2.69E-116
65	-21.3863198	0
66	-19.060165	0
67	-6.63582058	2.19E-108
68	-5.86261741	7.93E-75
69	-3.94178052	4.93E-130
70	-2.88734327	9.55E-102
71	-5.90883211	1.04E-26
72	-7.67328633	1.28E-41
73	-5.16324973	1.37E-174
74	-3.99184424	1.46E-126
75	-11.2723498	2.31E-195
76	-8.49557528	1.05E-154
77	-3.10106017	2.52E-20
78	-6.97492065	1.16E-63
79	-12.8207261	7.13E-191
80	-14.3425403	4.36E-240
81	-6.85008063	9.02E-285
82	-5.82963576	2.42E-223
83	-15.1223985	0
84	-14.5527773	2.05E-298
85	-5.07138503	1.98E-55
86	-4.57772853	6.31E-53
87	-5.79278733	0
88	-6.85783299	0
89	-8.1481913	0
90	-6.5215725	0
91	-5.42036533	7.71E-59
92	-5.13548632	3.85E-120
93	0.077790818	0.366328296
94	-0.89470105	1.19E-14
95	-28.9688132	3.64E-192
96	-21.5645219	5.88E-116
97	0.112422521	0.603459014
98	4.743868145	7.91E-98
99	24.05424632	0
100	27.63305913	0
101	3.075900413	3.60E-20
102	-0.01668235	0.962960009
103	1.077510314	6.43E-254
104	1.462098635	0
105	-7.25467313	1.97E-150
106	-7.2902385	4.53E-142
107	-6.51266011	0
108	-8.00362265	0
109	-4.28755292	0
110	-3.41660222	0
111	-27.563355	0
112	-4.43253215	7.97E-93
113	-5.12651419	6.62E-107
114	-6.88376519	2.92E-130
115	-6.06566365	2.79E-106

116	-30.0355731	0
117	-4.80453455	1.20E-144
118	-26.5846869	2.33E-293
119	-44.9444599	0
120	-0.01572655	1.09E-34
121	-52.6068311	0
122	-20.5802256	1.72E-119
123	-0.54809022	8.19E-19
124	-24.080881	5.54E-171
125	-12.8168808	4.66E-151
126	-0.48433682	2.64E-107
127	-12.1445495	7.73E-122
128	-16.3983066	3.58E-233
129	-3.55786411	1.54E-164
130	-15.5836085	1.38E-186
131	-8.17306403	1.64E-102
132	-1.23708397	7.40E-78
133	-9.95433477	1.19E-128
134	-3.36889854	5.01E-29
135	-0.49112987	2.47E-12
136	-4.18575644	1.19E-36
137	-2.02503338	6.57E-256
138	-0.74981317	6.94E-108
139	-2.35443742	0

IDP	Beta	p-Value	NewOSA_LM3_BMI_Summary
1	0.152447288	0.951483891	
2	-10.615996	0.000151818	
3	-8.42057446	7.60E-34	
4	-8.42432523	1.23E-33	
5	5.809000026	0.005988297	
6	6.851665204	0.000457053	
7	7.336565135	0.000376152	
8	15.25881325	1.73E-14	
9	2.695009043	0.000539257	
10	2.170755656	0.001216427	
11	0.570824324	0.412148413	
12	-0.55201488	0.430008004	
13	-13.0397754	5.28E-11	
14	-17.7906374	2.56E-19	
15	1.479991314	0.307545406	
16	-2.80886795	0.049150649	
17	-0.25446041	0.502215836	
18	-0.66464044	0.083764545	
19	-0.95218014	0.117333361	
20	-0.70272342	0.280311009	
21	-2.38533249	8.62E-07	
22	-1.74403871	1.25E-05	
23	-3.06575458	0.002664259	
24	-1.88659481	0.062370755	
25	-1.48765448	0.138807006	
26	4.360212753	0.000175421	
27	-0.89086126	0.028601353	
28	-2.77697274	7.69E-14	
29	2.368924627	0.0143021	
30	-2.41843603	0.009861049	
31	3.936845337	3.58E-07	
32	3.530080185	0.000138532	
33	-11.4861088	1.46E-10	
34	-11.0825972	2.39E-10	
35	-2.29513733	0.059386656	
36	-4.23689534	0.000383091	
37	-2.46592198	0.004011875	
38	-2.34312809	0.004111156	
39	-2.44222554	0.027341165	
40	0.404358681	0.76418778	
41	-1.55714486	0.132731044	
42	2.243709509	0.118321157	
43	-8.68259103	0.000688587	
44	5.326126199	0.04327229	
45	-4.72907501	0.001342344	
46	5.459033076	0.000421023	
47	-0.60743227	0.438305829	
48	-2.34548006	0.001648337	
49	-0.24866311	0.551038424	
50	-1.78720088	2.96E-05	
51	1.964783613	0.007278979	
52	0.276864477	0.705299776	
53	0.775659291	0.072851905	
54	0.689500631	0.078574127	
55	1.64412537	0.088297153	
56	-0.04370612	0.964478389	
57	4.370500397	0.000855153	

58	2.25253268	0.122868572
59	0.762590212	0.316773442
60	0.225736542	0.77661585
61	6.487237162	7.40E-06
62	7.931167615	1.86E-07
63	2.751433932	7.07E-08
64	4.091936487	8.55E-13
65	-0.50867103	0.569202579
66	-1.51474656	0.06960376
67	-3.34009008	8.18E-11
68	-3.20174154	5.67E-09
69	0.877577916	0.001597881
70	0.850426695	0.000232001
71	-4.03496432	2.13E-05
72	-7.13025016	2.70E-13
73	-2.89056475	2.81E-20
74	-3.10809992	1.50E-27
75	1.400197477	0.029995893
76	-2.96010875	6.69E-08
77	-0.59914271	0.299126948
78	1.195777598	0.092391477
79	-1.45655895	0.049840672
80	-2.98585214	5.27E-05
81	0.106231169	0.742231787
82	0.067669301	0.828020305
83	-3.68659011	8.74E-09
84	-3.14627	2.65E-06
85	-2.48451843	7.63E-06
86	-1.29235582	0.011798212
87	-2.38158577	1.26E-20
88	-2.48309632	6.40E-22
89	-2.55808129	1.29E-15
90	-2.42196336	1.94E-24
91	-1.34022739	0.019801842
92	-0.67177075	0.074946347
93	0.281029601	0.057811771
94	0.437959004	0.028028382
95	12.28524216	2.08E-13
96	18.84390772	1.86E-31
97	-1.34548788	0.00030215
98	1.408065784	0.000277162
99	-3.8747667	1.27E-05
100	-2.75909294	0.002768263
101	-7.42512635	4.51E-38
102	-11.6879153	2.72E-79
103	-0.31185728	7.28E-09
104	-0.4355154	7.26E-13
105	-2.64749313	2.49E-08
106	-1.05830271	0.031258592
107	-5.14020915	1.25E-81
108	-5.95166932	3.24E-92
109	-2.43563426	7.14E-70
110	-2.77842762	5.91E-91
111	5.355181288	2.53E-07
112	-2.4619742	3.58E-11
113	-1.5102814	0.000158774
114	-5.79260062	8.62E-33
115	-5.68501817	5.35E-33

116	-19.2915576	3.70E-53
117	-2.29112002	9.50E-13
118	-16.0239428	2.03E-38
119	-26.9928615	5.03E-43
120	0.001825031	0.40661492
121	-25.8509353	1.62E-34
122	-29.8664097	7.85E-86
123	-0.78245644	1.95E-13
124	-28.0661301	3.96E-80
125	-17.2003424	3.87E-93
126	-0.30662082	4.36E-16
127	-16.9359459	5.36E-81
128	-23.7377895	1.27E-166
129	-3.08011307	1.77E-43
130	-26.793364	2.13E-186
131	-18.2207506	6.88E-170
132	-1.98231151	7.38E-68
133	-21.8755804	2.45E-207
134	-12.3541269	1.27E-124
135	-2.36601486	3.43E-85
136	-14.9546928	1.24E-150
137	-0.10500945	0.297943141
138	-0.52644313	1.59E-19
139	-0.16947939	0.095518581

IDP	Beta	p-Value	NewOSA_LM3_DEPRESSIVE_SYMPTOMS_Summary
1	-10.8757574	0.708462713	
2	-37.4712229	0.24934166	
3	-4.34960249	0.588973849	
4	-2.6249271	0.745273737	
5	3.891663434	0.873963737	
6	-3.74556481	0.868893801	
7	-10.5165959	0.660523374	
8	27.92940425	0.226345547	
9	-5.75255089	0.524538872	
10	-8.37674698	0.28216016	
11	-7.22177897	0.371423402	
12	-9.20694515	0.256847692	
13	-40.4523548	0.079345206	
14	-61.2566372	0.007650112	
15	-14.7228062	0.381884853	
16	-5.42858667	0.743259632	
17	7.098022274	0.106882427	
18	-1.21202542	0.785885653	
19	1.667046624	0.813279504	
20	2.431427284	0.747607807	
21	1.445617202	0.79721565	
22	6.92865402	0.13480031	
23	3.059701237	0.796182729	
24	3.296606867	0.779063851	
25	-4.5822282	0.694485265	
26	10.45653492	0.438210938	
27	19.08711844	5.35E-05	
28	3.676978739	0.393605069	
29	23.03882775	0.040142779	
30	-6.32520032	0.560927659	
31	-25.9866004	0.003793186	
32	-19.936633	0.063714134	
33	-36.1221672	0.082400548	
34	-47.2267476	0.020026379	
35	2.607590157	0.853601123	
36	-4.08580805	0.767946314	
37	-29.9713199	0.002591183	
38	1.561496407	0.869145085	
39	-20.499943	0.110573841	
40	1.109515135	0.943470974	
41	-10.2352608	0.39460208	
42	-30.9952261	0.063079252	
43	11.17246016	0.706720222	
44	15.57870513	0.610566435	
45	-2.81855727	0.869212395	
46	2.66552817	0.882065338	
47	4.638780206	0.610135176	
48	0.761377835	0.929864139	
49	-6.21661281	0.199146846	
50	-4.1708187	0.401084091	
51	-12.4232343	0.143768608	
52	-11.4828518	0.176653451	
53	-5.22266273	0.298131895	
54	-0.3284862	0.942448654	
55	-26.1040347	0.019744259	
56	-24.8145876	0.029401523	
57	11.98207707	0.430965226	

58	25.38781507	0.134140062
59	-12.4663344	0.158602986
60	-22.4381286	0.015124122
61	-11.2263432	0.503965833
62	-8.42255321	0.633378094
63	-1.10467164	0.852096414
64	-5.74017074	0.38721782
65	-7.66955197	0.459700305
66	-7.94623682	0.412218781
67	-10.1888042	0.08764844
68	-9.94491746	0.118911942
69	-6.48147451	0.04461772
70	-7.53823254	0.004935968
71	13.84323446	0.208968243
72	3.74267565	0.740919776
73	2.652715141	0.465447389
74	0.313863644	0.924546473
75	-2.43720677	0.74486737
76	-2.21220081	0.72807347
77	-3.02210409	0.651870293
78	10.03959613	0.223529457
79	-1.63418685	0.849647275
80	6.584304674	0.442399427
81	-2.81632752	0.452569551
82	-3.88461255	0.282692764
83	7.711743553	0.299696717
84	-2.38767502	0.758784945
85	-9.37533033	0.145664746
86	2.6403403	0.657616573
87	0.940367708	0.751277727
88	-0.71577619	0.81098884
89	4.889163985	0.18777814
90	-0.45146574	0.86975321
91	3.541153392	0.595850608
92	5.536594638	0.206103272
93	0.863675019	0.615480264
94	-0.13489993	0.953513535
95	-11.1404177	0.566022793
96	21.82814425	0.24374001
97	-7.65229428	0.076641425
98	-3.59437206	0.423944136
99	30.05424907	0.003535966
100	36.20089666	0.000719366
101	19.33966277	0.003749885
102	19.42340242	0.006779062
103	0.837411353	0.180761674
104	1.035116426	0.141672397
105	-5.31818901	0.334619185
106	-7.75924694	0.173723645
107	-4.83090783	0.120098639
108	-10.2661956	0.00238595
109	1.401249904	0.37985785
110	0.61020116	0.700953252
111	-24.8606736	0.039206985
112	0.528722983	0.902480978
113	6.084095237	0.189908675
114	1.872241041	0.739521862
115	6.15192447	0.264047812

116	5.27628382	0.717092395
117	3.51485762	0.345325507
118	10.16278967	0.478200866
119	-34.8679334	0.125350223
120	0.023267242	0.362084583
121	-36.3020377	0.137753041
122	6.559407023	0.709333568
123	1.912286743	0.121474605
124	6.46286126	0.70600626
125	1.232014299	0.899108564
126	0.410514538	0.34825426
127	5.078448973	0.621335732
128	8.564818393	0.389292335
129	2.611957257	0.31166969
130	3.93659386	0.710387966
131	5.402412053	0.474897689
132	0.95657456	0.468129096
133	2.078596797	0.799771201
134	3.364742498	0.5755583
135	2.31296595	0.098384813
136	7.828113643	0.235636783
137	1.517337075	0.195115584
138	0.08783227	0.896571662
139	0.459440493	0.697057136

IDP	Beta	p-Value	NewOSA_LM3_EARLY_CHRONOTYPE_Summary
1	-37.4076184	0.117515059	
2	-51.2810216	0.055022978	
3	-9.56447072	0.148165346	
4	-9.23411233	0.164239008	
5	-18.8690894	0.349236772	
6	-5.62885597	0.762721	
7	-16.7936615	0.393338164	
8	-14.1280141	0.456362663	
9	-17.3994161	0.019154066	
10	-16.5880373	0.009543998	
11	-6.32980369	0.340344731	
12	-6.67508881	0.317054534	
13	-31.3332787	0.09812494	
14	-14.6844319	0.436445441	
15	-32.5994778	0.018453169	
16	-27.1251585	0.04638719	
17	-5.18842599	0.151442914	
18	-9.80360448	0.007491251	
19	-9.12585684	0.115563284	
20	-5.87037904	0.344356459	
21	-1.78262385	0.699763968	
22	-0.03920983	0.991781776	
23	6.98414254	0.473023417	
24	-7.68791536	0.425880357	
25	-16.0579134	0.093893093	
26	-7.66668899	0.489076619	
27	6.472507323	0.095424322	
28	8.911504775	0.011859952	
29	20.59706575	0.025544956	
30	-0.93101658	0.917036863	
31	-9.56265223	0.194768812	
32	-2.3916885	0.786597399	
33	29.50554298	0.084209757	
34	-2.67488473	0.872613001	
35	2.079685696	0.857845557	
36	11.61524209	0.307302184	
37	20.17230688	0.013594895	
38	15.54500474	0.045930137	
39	0.571735205	0.956805176	
40	-13.5636273	0.291421196	
41	-9.35198461	0.34379429	
42	-7.92005107	0.563234019	
43	-44.5178915	0.068047655	
44	12.83692784	0.609536138	
45	-12.5767233	0.371202737	
46	-11.1157725	0.451477051	
47	0.104386621	0.988858107	
48	-4.27123119	0.547876753	
49	-1.97913121	0.618823597	
50	-0.55531513	0.891766362	
51	-6.76647929	0.332490423	
52	-6.55817769	0.347629127	
53	-12.239694	0.003002263	
54	-7.64486107	0.040874297	
55	-10.3444208	0.260853428	
56	-8.59578899	0.358459919	
57	23.62919229	0.058735075	

58	19.11562245	0.169830223
59	-18.9870341	0.008969458
60	-10.7606243	0.1561812
61	-14.2625122	0.301464883
62	12.07620261	0.405212076
63	-0.93913552	0.847025337
64	13.38123141	0.014160745
65	-33.3252476	9.25E-05
66	-24.316227	0.002259912
67	-5.81954176	0.235104127
68	-8.14849713	0.119934051
69	-5.61935312	0.03408299
70	-4.93513876	0.025089349
71	-3.1606238	0.7269876
72	-3.57243	0.700898275
73	1.069344782	0.720259748
74	2.219275335	0.415049789
75	-3.70398045	0.54723103
76	-4.19981535	0.421758738
77	-5.95136771	0.279546113
78	5.405461413	0.425083528
79	4.115060775	0.561253079
80	-3.3748258	0.631801566
81	-5.50549785	0.073923457
82	-5.31021705	0.073886814
83	-4.39776544	0.471641705
84	5.41337891	0.396802777
85	5.976406461	0.258949035
86	0.404732115	0.934100467
87	-2.20328002	0.366082572
88	-6.67888982	0.006612215
89	2.4632361	0.419270365
90	0.084333369	0.970262365
91	-0.73813746	0.892963501
92	-3.65869374	0.309202927
93	-0.98386551	0.486196151
94	0.815487826	0.667993146
95	7.995184256	0.616157901
96	-10.6909552	0.487131774
97	-8.41244314	0.017842044
98	-10.7895599	0.003488152
99	-6.97107164	0.410228483
100	-11.2732521	0.199855651
101	-9.96786428	0.069019106
102	-14.2934731	0.015308736
103	0.577224946	0.261501802
104	0.890972477	0.12367032
105	-3.97062117	0.380619577
106	-7.40948109	0.113875877
107	-5.24544021	0.039966584
108	-2.87979347	0.299696706
109	-0.74971541	0.567427346
110	-0.21096545	0.871623885
111	5.235698083	0.597114372
112	7.302080063	0.039447395
113	5.9413995	0.119235018
114	5.957729911	0.197855475
115	7.141415711	0.11457509

116	13.94951276	0.243641145
117	5.637620883	0.065448418
118	20.46052061	0.082256604
119	7.617629263	0.683603798
120	0.000736435	0.971992442
121	8.241268352	0.681733091
122	-7.46129572	0.605820214
123	-0.45333957	0.654995971
124	-12.6813769	0.367657286
125	-3.35039001	0.674747575
126	0.466288058	0.194734744
127	-8.07337481	0.339209813
128	2.958246889	0.717422994
129	1.486002926	0.48358241
130	-1.30304103	0.881081592
131	7.948452657	0.20072613
132	2.253929835	0.037473105
133	10.20588419	0.129591384
134	0.870581808	0.8600495
135	0.573615617	0.617871182
136	6.447829534	0.234473722
137	1.14005956	0.236104278
138	0.715218721	0.19763614
139	0.469066432	0.628574052

IDP	Beta	p-Value	NewOSA_LM3_EDUCATION_Summary
1	13.01586115	0.539767181	
2	-26.6098319	0.262335095	
3	-1.14906253	0.844935705	
4	-2.47190538	0.675071899	
5	-3.67907272	0.837200202	
6	1.233230785	0.940638869	
7	3.973030509	0.820155701	
8	19.15638479	0.255538727	
9	4.775857282	0.469124801	
10	-1.25056722	0.825871223	
11	8.704195514	0.139923272	
12	12.4156778	0.03616761	
13	10.12910833	0.547193704	
14	-5.12054862	0.759980752	
15	-43.6578839	0.000381576	
16	-32.3172875	0.007549317	
17	-12.2137507	0.000144032	
18	-13.4025395	3.86E-05	
19	-2.56267348	0.618827382	
20	-13.9046911	0.011689221	
21	-19.59452	1.83E-06	
22	-10.511137	0.001881581	
23	-3.62505764	0.674994417	
24	-8.51760521	0.320625661	
25	6.052633267	0.477159681	
26	24.1283378	0.014249642	
27	-14.8323472	1.70E-05	
28	-4.9912927	0.112577708	
29	-9.07014516	0.268251844	
30	-3.94606642	0.619155539	
31	12.24595032	0.061583432	
32	16.13181212	0.039809723	
33	-9.66777596	0.524135542	
34	-1.56461646	0.915910604	
35	24.79871134	0.016198536	
36	10.0243309	0.321241575	
37	2.768404419	0.702980165	
38	6.075415298	0.379797769	
39	0.706929026	0.93989986	
40	0.882861145	0.938376476	
41	-8.23605331	0.347928602	
42	-3.02452822	0.803735368	
43	10.56339545	0.625937806	
44	24.56981523	0.271108594	
45	30.66777264	0.014099396	
46	16.81701931	0.199689383	
47	23.24889262	0.000463335	
48	20.5713914	0.001121519	
49	-4.53219349	0.199623769	
50	-5.15545476	0.154985633	
51	-1.54822942	0.802867249	
52	3.821973852	0.537758953	
53	-4.87397231	0.183375434	
54	-6.50355487	0.050186001	
55	22.63568852	0.005610396	
56	24.88446851	0.002765445	
57	0.244909593	0.982403001	

58	-11.2864887	0.361511305
59	10.34230603	0.109032118
60	15.8590382	0.018635661
61	57.64664639	2.59E-06
62	53.12238497	3.76E-05
63	10.04203855	0.020221656
64	12.38691508	0.010571853
65	2.512454131	0.739992739
66	-0.04290451	0.995159644
67	-19.7284247	5.88E-06
68	-20.0454192	1.66E-05
69	-5.3045084	0.024322539
70	-3.83881984	0.049805358
71	-10.952387	0.173189902
72	-14.8195217	0.072845076
73	-13.9812786	1.36E-07
74	-10.6367089	1.10E-05
75	-1.45904295	0.789523121
76	-4.88284413	0.293016608
77	-1.83984083	0.706657032
78	-7.93767546	0.18729452
79	10.48725904	0.095541313
80	16.7081804	0.007569591
81	-1.52654892	0.576933314
82	-0.7750595	0.768990509
83	3.07547067	0.570911692
84	8.039123381	0.156590083
85	13.19388783	0.005023248
86	6.015417899	0.166501406
87	-0.35899486	0.868317824
88	2.182159733	0.31779908
89	6.92240481	0.010611112
90	-0.68024499	0.734965494
91	4.955905156	0.309127232
92	-8.05282841	0.011747146
93	3.338772213	0.007807404
94	2.008734047	0.234288554
95	70.22322796	7.20E-07
96	85.47325237	4.05E-10
97	5.783437085	0.066730374
98	1.03319647	0.752812729
99	10.8650498	0.148476605
100	8.660247254	0.267553009
101	-4.54858697	0.350233903
102	-8.28146291	0.113692787
103	-0.89399142	0.050257135
104	-0.78008513	0.129136346
105	-15.5214282	0.000114332
106	-14.7457169	0.000397264
107	-6.84794145	0.002537367
108	-5.4351072	0.027561328
109	0.016944648	0.988390809
110	-2.30484395	0.046866595
111	41.30861482	2.68E-06
112	-1.52843248	0.627442718
113	1.837552626	0.587485453
114	-17.8692593	1.38E-05
115	-17.1046668	2.10E-05

116	-41.5230628	9.36E-05
117	-9.92690448	0.000260688
118	-41.8295533	6.36E-05
119	-17.916377	0.280534263
120	0.055461277	0.002915484
121	-9.16517836	0.60763716
122	-80.3118434	4.07E-10
123	-2.87857562	0.00140362
124	-71.2366766	1.23E-08
125	-43.8725252	6.24E-10
126	-1.31415479	3.89E-05
127	-49.7758439	3.33E-11
128	-29.6560471	4.43E-05
129	-9.056343	1.54E-06
130	-43.2311575	2.32E-08
131	-8.12618374	0.140845673
132	-1.33329301	0.165867821
133	-3.35611656	0.574700036
134	-4.82498813	0.271297783
135	-4.50841469	1.02E-05
136	-0.41623868	0.93114327
137	-0.59035079	0.489757578
138	1.110964791	0.024268574
139	0.244707972	0.776327524

IDP	Beta	p-Value	NewOSA_LM3_EXCESSIVE_DAYTIME_SLEEPINESS_Summar
1	-33.245399	0.270750861	
2	-83.0998702	0.013838129	
3	-18.7441726	0.02486545	
4	-5.74844746	0.49301323	
5	-39.7870613	0.118151575	
6	-7.73872376	0.742454044	
7	-57.7307057	0.020183001	
8	-28.379295	0.236216007	
9	-3.18321632	0.734383735	
10	-11.9034889	0.140874132	
11	-9.36147251	0.264256572	
12	-10.0628839	0.232437429	
13	13.81314658	0.563753089	
14	4.531404835	0.849216728	
15	-1.02320639	0.95330579	
16	4.115637088	0.810897309	
17	0.35001083	0.938933339	
18	3.230965571	0.485322073	
19	3.861221208	0.598099467	
20	4.915262377	0.530784594	
21	-2.75936835	0.636509795	
22	-1.57097624	0.743883866	
23	-19.9866495	0.104021341	
24	-20.5412716	0.092127641	
25	9.352192185	0.43986881	
26	8.474615183	0.544926608	
27	10.81680916	0.027378581	
28	4.083512967	0.361307131	
29	-9.61173482	0.409362815	
30	-2.66062685	0.813688516	
31	-10.5326487	0.258216468	
32	-5.16984218	0.643151288	
33	31.25423037	0.14759654	
34	21.4996195	0.307602724	
35	3.723585668	0.799578679	
36	7.023320866	0.625048987	
37	-3.95079498	0.701974443	
38	-5.16649812	0.599434354	
39	15.67234474	0.239834171	
40	5.503720201	0.734674776	
41	10.89927147	0.382401731	
42	4.495001557	0.795076362	
43	-36.6887942	0.233834065	
44	-12.460883	0.694693334	
45	16.2006218	0.361809119	
46	-7.2311	0.698179912	
47	-26.2483614	0.005439718	
48	-18.8741748	0.035532243	
49	-1.42913772	0.776089735	
50	-2.15399757	0.676061067	
51	1.413378172	0.872677568	
52	-0.78075349	0.929463649	
53	-3.91160929	0.452739047	
54	1.476330092	0.754562338	
55	-34.3239311	0.003142947	
56	-51.4431148	1.36E-05	
57	-9.73140494	0.537699813	

58	-5.61454302	0.749569385
59	-3.02800846	0.741436798
60	-3.32152577	0.728943588
61	36.58496213	0.035878713
62	6.235128991	0.733684162
63	-0.06551271	0.991499501
64	-7.89802057	0.251653114
65	-10.0262075	0.351707903
66	-1.74078684	0.862581598
67	1.744708663	0.778090855
68	6.323522106	0.339380872
69	-2.75784789	0.410296212
70	0.135460663	0.961175753
71	14.23539079	0.21316943
72	9.715618471	0.40823574
73	3.614655485	0.337898576
74	4.812794599	0.161725533
75	1.89045737	0.807842151
76	-4.08862297	0.535800474
77	-5.71119886	0.41134488
78	-15.0604585	0.078521316
79	0.523156291	0.953370605
80	-23.6283458	0.007909337
81	-8.64933979	0.02623847
82	-4.7616313	0.204506417
83	11.19306671	0.1469565
84	7.125483306	0.377243045
85	14.38294773	0.03149567
86	9.104236872	0.14088801
87	-0.27554248	0.928694878
88	-2.63288297	0.396657156
89	3.507780572	0.36251714
90	1.754034264	0.539323426
91	13.34002941	0.054216139
92	8.326826157	0.066923738
93	-2.85681959	0.109428121
94	-4.08492611	0.088972044
95	-75.863443	0.000166352
96	-48.4677958	0.012636317
97	2.049181011	0.647781121
98	0.270021061	0.953845169
99	20.83613073	0.051348309
100	34.5639109	0.001861539
101	14.80519321	0.032511878
102	14.36503599	0.053673614
103	1.40641702	0.030323746
104	0.85190951	0.243856973
105	-5.2758376	0.356386108
106	-6.19301638	0.295481878
107	-0.44101334	0.891246437
108	-0.57279736	0.870276311
109	-0.02893979	0.986057374
110	-0.91512624	0.578933848
111	-0.43886512	0.972020041
112	-4.02077496	0.369286768
113	-5.66135583	0.239873728
114	0.129855044	0.982272517
115	-3.17799408	0.578257603

116	-28.8542504	0.056228933
117	-4.92907544	0.202259887
118	-20.3753786	0.17068076
119	-46.9927982	0.046553808
120	-0.00477106	0.857093854
121	-56.0882777	0.027138676
122	-34.7303435	0.057222232
123	-3.03113264	0.018024064
124	-22.5453104	0.204820596
125	-19.3233098	0.055362954
126	-0.54747442	0.228084293
127	-9.65520744	0.365530521
128	-15.9765162	0.121784426
129	-5.8601814	0.028736948
130	-22.3436704	0.042280762
131	-3.40343867	0.664480609
132	0.023209401	0.986467103
133	-20.983425	0.013623287
134	-0.98327634	0.874732494
135	-0.21649374	0.881506345
136	-4.21780937	0.538085963
137	0.57161191	0.638148648
138	-1.77265122	0.011479071
139	0.900597915	0.462170466

IDP	Beta	p-Value	NewOSA_LM3_INSOMNIA_SYMPTOMS_Summary
1	-35.459642	0.137377917	
2	-53.3076011	0.045825738	
3	4.723682184	0.474567777	
4	5.767495132	0.384376691	
5	-5.26993586	0.793505235	
6	-0.75400111	0.967700912	
7	-1.86557272	0.924361795	
8	-8.81347944	0.641760445	
9	0.642887372	0.930937712	
10	-1.65322993	0.795898845	
11	-3.39176381	0.608963414	
12	7.564567282	0.256266295	
13	-29.4068151	0.120123835	
14	-18.2526275	0.33279058	
15	-4.08302843	0.767599636	
16	3.845268951	0.777386497	
17	-4.94634422	0.170928137	
18	-3.54952621	0.332305788	
19	-7.49865706	0.195425999	
20	-2.6215968	0.672433121	
21	-2.10961884	0.647709845	
22	4.026203103	0.289615572	
23	5.712463322	0.556771453	
24	6.443291617	0.504017061	
25	-0.76386061	0.936404815	
26	-17.562342	0.112598364	
27	1.558589981	0.687658932	
28	-1.78892657	0.613008841	
29	6.028884304	0.512809306	
30	1.161317879	0.896490604	
31	-6.11574868	0.406384421	
32	-9.97051956	0.258466396	
33	-11.3264883	0.506879539	
34	-8.76000776	0.599060494	
35	-0.71179478	0.951055706	
36	-8.14437758	0.47354198	
37	1.637987542	0.84097669	
38	2.553988404	0.742639092	
39	-5.54575563	0.598869085	
40	5.60475095	0.662477098	
41	-1.50398549	0.878841543	
42	-12.3289625	0.367618063	
43	-9.93357258	0.683513557	
44	24.86610036	0.321901576	
45	15.63939849	0.265553065	
46	-8.81552252	0.549916415	
47	5.090723047	0.495317101	
48	7.913915882	0.264922549	
49	-4.04088562	0.309123544	
50	0.997886721	0.80659792	
51	-11.180935	0.108862714	
52	-9.41729365	0.176913846	
53	-10.1498162	0.013742027	
54	-8.6786298	0.02011633	
55	-0.49098374	0.957386157	
56	0.4493631	0.961663376	
57	17.6432964	0.157621578	

58	-2.56416351	0.853721452
59	-6.21703641	0.391571261
60	1.504605003	0.842633077
61	3.455296082	0.802089002
62	9.305576531	0.520754052
63	3.589875114	0.460308824
64	2.248401636	0.679804458
65	-12.7092498	0.135445789
66	-5.25261064	0.508917861
67	1.716712023	0.725823771
68	6.393526741	0.221835736
69	-2.47064428	0.350880844
70	0.305554203	0.889554829
71	0.207474237	0.981692562
72	-7.38186956	0.426797735
73	-2.50100848	0.401695344
74	-0.43460472	0.873030708
75	-7.61864921	0.215125704
76	-2.67724979	0.608116354
77	0.784449427	0.886519203
78	1.240217401	0.854613033
79	6.276374349	0.374958885
80	15.1110563	0.031697179
81	-1.58272788	0.606967847
82	0.999561843	0.736222202
83	-19.7009402	0.001245261
84	-3.96591059	0.53423797
85	-1.64586091	0.75559138
86	-7.90952143	0.105686157
87	2.909073222	0.232147312
88	2.603111274	0.289212032
89	-2.83771516	0.351525849
90	1.510302319	0.503845876
91	-2.89959285	0.59665027
92	-7.3854335	0.039859772
93	2.035278947	0.149210959
94	4.340711043	0.022270432
95	16.77441632	0.292310616
96	6.633024715	0.665986536
97	1.117047801	0.752792933
98	-0.36893961	0.920331823
99	3.603252564	0.66997456
100	7.450193297	0.396284318
101	6.063897145	0.268057908
102	5.064394515	0.389613054
103	-0.63791819	0.214069663
104	0.006741977	0.990693301
105	0.254787214	0.955078863
106	8.1371971	0.082133619
107	1.915261356	0.452673038
108	1.900056473	0.493273589
109	1.268299432	0.332748983
110	0.642890603	0.621973443
111	16.93476027	0.086954648
112	4.690122959	0.185342511
113	2.261294256	0.552699568
114	7.225317124	0.117914091
115	3.178075084	0.481987502

116	21.16197487	0.076570557
117	4.626401077	0.130115772
118	17.11706799	0.145504378
119	-4.15236931	0.823977438
120	-0.03774983	0.071560135
121	1.350335133	0.946359909
122	-0.80956716	0.955292236
123	2.233525109	0.02751642
124	16.93796102	0.228302652
125	-1.35319259	0.865245799
126	0.442094733	0.218341283
127	-1.53301338	0.855813808
128	10.18197329	0.212334336
129	2.821293933	0.1829611
130	0.267271291	0.975490619
131	12.60722952	0.04216365
132	1.406295313	0.1936774
133	5.14778491	0.443985865
134	7.87292783	0.110403774
135	0.839333395	0.464859376
136	7.131453935	0.187973271
137	1.052634537	0.273390278
138	0.113092239	0.838378439
139	-0.71190474	0.462291902

IDP	Beta	p-Value	NewOSA_LM3_LATE_CHRONOTYPE_Summary
1	-22.7915227	0.537477081	
2	21.78791744	0.598127834	
3	-4.72355394	0.644260739	
4	-4.58754141	0.655017923	
5	-8.54348111	0.784057627	
6	-18.5171415	0.520772421	
7	34.48469132	0.257101098	
8	47.57541238	0.104865973	
9	6.724483996	0.558292287	
10	12.96936378	0.190088659	
11	23.87975407	0.020036664	
12	18.32483957	0.07575171	
13	11.19283468	0.702441611	
14	1.021775844	0.972070747	
15	-29.4149756	0.169201752	
16	-10.4328329	0.620353069	
17	-6.24623478	0.26418168	
18	-0.93281719	0.869315669	
19	-12.9562413	0.148585176	
20	-7.89503222	0.410935374	
21	-11.4867972	0.108131021	
22	-5.07193477	0.388988196	
23	-9.99086403	0.50688986	
24	-2.07093152	0.889700076	
25	9.807553219	0.508261832	
26	6.670463989	0.697158651	
27	-0.96850133	0.871837186	
28	-2.50737859	0.647106003	
29	12.39163595	0.385032186	
30	5.931324419	0.667868014	
31	16.0775883	0.158694191	
32	20.76848091	0.128513142	
33	-58.6509691	0.026469095	
34	-61.3078222	0.017502512	
35	-11.0902346	0.536855356	
36	-20.0637213	0.254208875	
37	0.312371287	0.980286933	
38	-20.3452041	0.091201117	
39	2.60459052	0.87324653	
40	-15.4844612	0.436136635	
41	-11.6997225	0.443818205	
42	6.870528444	0.745774232	
43	-33.442408	0.375464744	
44	23.24624589	0.549842744	
45	35.06195978	0.106999947	
46	-1.8329459	0.936016875	
47	-9.0390996	0.434304709	
48	-5.57409882	0.612106355	
49	-11.1407792	0.070185763	
50	-13.4950093	0.032524881	
51	-6.57952385	0.542338391	
52	-16.8073494	0.119650002	
53	-9.51214902	0.135909169	
54	-3.65878814	0.526885378	
55	0.668918642	0.962504915	
56	-10.5360227	0.46675965	
57	52.50076968	0.006622079	

58	77.27171987	0.000333868
59	4.699693437	0.675772766
60	10.06465311	0.391137028
61	8.603033733	0.686955179
62	-11.5582087	0.606494604
63	7.533284171	0.317054788
64	9.809996684	0.244886577
65	-12.0473071	0.360771836
66	-5.33305804	0.664961679
67	-11.2851354	0.136581279
68	-14.6422712	0.070811239
69	-8.25549465	0.044127142
70	-6.28766616	0.064998464
71	-10.3801778	0.458469267
72	-7.14396767	0.619444736
73	-13.7469356	0.002917055
74	1.662996477	0.69292736
75	-6.46915038	0.496688203
76	-6.6495121	0.41084437
77	4.011578271	0.637444076
78	7.871318078	0.452664448
79	1.439813284	0.895432923
80	6.724063627	0.537032823
81	0.064163946	0.989255408
82	-2.13993989	0.641425733
83	-8.6398573	0.360544561
84	-8.67598928	0.379914368
85	-5.74505895	0.482902842
86	-4.16207455	0.582470624
87	6.494166702	0.084988887
88	-2.6241931	0.490221791
89	-0.58905742	0.90061509
90	-0.17607584	0.959863952
91	-1.81536613	0.830576104
92	-3.24815224	0.559412781
93	1.589275767	0.467040801
94	3.04427934	0.300564506
95	-4.12802389	0.867094749
96	-9.01293046	0.704862327
97	-6.93886484	0.206451176
98	-1.96937708	0.730277515
99	19.3742348	0.138939469
100	27.16074266	0.045829519
101	6.874445871	0.417473139
102	0.914655807	0.92007636
103	0.482613047	0.54385047
104	1.110845186	0.214579623
105	-11.4307869	0.102696001
106	-6.21070804	0.391527396
107	-2.74888424	0.486418198
108	0.514377036	0.904665179
109	-3.83029288	0.058903752
110	-1.97445228	0.328147341
111	-28.0613163	0.067016418
112	-10.5981611	0.053277818
113	-12.0238727	0.041493322
114	-8.58703147	0.230136993
115	-6.26008045	0.371133759

116	-19.0748036	0.302620946
117	-8.89807265	0.060116951
118	-7.59550034	0.676601112
119	-28.8570588	0.318178691
120	-0.01484461	0.647253148
121	-22.5236706	0.468651379
122	-15.4524957	0.489558565
123	0.889610542	0.570760201
124	-23.5341993	0.279720628
125	-12.989045	0.292853774
126	-0.37999952	0.494445336
127	-12.0849387	0.354974148
128	-11.7292553	0.353529038
129	-2.94129391	0.369965484
130	-23.6229751	0.079518994
131	-15.6629844	0.103064765
132	-3.32962404	0.04689619
133	-16.9223413	0.104175888
134	-15.8780829	0.037613942
135	-1.17463915	0.508925566
136	-12.1449645	0.14764288
137	-3.46878997	0.01977005
138	-0.39319931	0.646992741
139	-3.6705563	0.014391743

IDP	Beta	p-Value	NewOSA_LM3_LONG_SLEEP_DURATION_Summary
1	-133.223114	0.197629864	
2	-296.29043	0.01040931	
3	4.575498767	0.872980408	
4	15.77635795	0.582858115	
5	-174.745984	0.045136235	
6	-75.2447724	0.350975782	
7	-85.4930632	0.315260905	
8	-14.3352196	0.861343174	
9	-23.2785413	0.46886522	
10	-23.4954122	0.396166875	
11	-30.1036651	0.294647745	
12	7.993696239	0.781851006	
13	-52.1266713	0.524818442	
14	-82.0769002	0.314781106	
15	-165.746456	0.005627034	
16	-111.981711	0.057380906	
17	17.35956419	0.267350437	
18	-10.3393685	0.514509481	
19	-3.69373526	0.882969044	
20	28.60390534	0.286960508	
21	9.072016471	0.65014456	
22	-11.8022093	0.473675697	
23	17.48083956	0.678088136	
24	77.13379415	0.064855044	
25	9.107649374	0.826192224	
26	-72.2119312	0.132114169	
27	-3.49293269	0.835255268	
28	-11.9201312	0.436628888	
29	-3.26987468	0.934700334	
30	-25.4828368	0.509949992	
31	-18.7253367	0.55735302	
32	-31.4952696	0.40998121	
33	-128.652383	0.081852473	
34	-16.2584337	0.821799893	
35	-60.9016135	0.225435809	
36	-8.36982937	0.864998651	
37	90.52899956	0.010483166	
38	-0.07216574	0.998291266	
39	79.27395868	0.08264209	
40	-19.8121131	0.721732478	
41	51.93067007	0.224405845	
42	-24.7076027	0.676861225	
43	-185.965911	0.078141852	
44	-156.710558	0.149609543	
45	-39.679489	0.514392247	
46	-28.1655357	0.659270886	
47	-30.7096456	0.342393423	
48	-7.35149324	0.811075095	
49	-35.2367243	0.040654369	
50	-39.5878198	0.024981758	
51	-16.2806753	0.589966086	
52	-23.7989281	0.430890653	
53	11.55635369	0.517264381	
54	18.50463858	0.252668828	
55	-91.0800178	0.022147235	
56	-64.3742725	0.11198309	
57	77.66281838	0.151070184	

58	36.95904951	0.539614862
59	117.8458517	0.000178152
60	147.4542055	7.12E-06
61	25.57110677	0.668543871
62	-75.7022642	0.2278767
63	-16.4860795	0.433835863
64	-23.6843784	0.315614307
65	-46.1633032	0.210678327
66	-71.7243137	0.037362143
67	-51.8475468	0.014503472
68	-20.5520594	0.36470243
69	-2.42177384	0.832833362
70	-9.58503574	0.314665514
71	18.13435301	0.643400281
72	3.734003081	0.926074647
73	-27.4027336	0.033943116
74	-9.30396745	0.429719253
75	8.346267352	0.753937551
76	-6.14802253	0.785782181
77	8.2697197	0.728398116
78	22.4980343	0.442944156
79	-32.7137076	0.28579519
80	-35.0855819	0.249602455
81	-25.9918449	0.051197367
82	-13.1848425	0.305074814
83	-9.71781311	0.713176747
84	15.576049	0.573122773
85	17.39761172	0.447571522
86	17.10013252	0.419448261
87	11.80182774	0.263196903
88	12.83618002	0.227698724
89	-6.49817441	0.622417806
90	15.68085151	0.109175337
91	14.80270691	0.532882573
92	6.529376825	0.674918047
93	12.33773842	0.043583065
94	11.69528914	0.155161339
95	-24.5737	0.721776743
96	-58.9174666	0.376153585
97	12.6456383	0.410516618
98	23.20278272	0.1465483
99	151.2301708	3.66E-05
100	147.615278	0.000104898
101	58.60716416	0.013486362
102	42.42840259	0.096189795
103	3.756488127	0.09126905
104	6.443565067	0.010080336
105	-31.2832311	0.110404404
106	-19.1478689	0.345044756
107	-12.2330666	0.268240166
108	-14.7055523	0.220992646
109	6.537242049	0.249178497
110	2.609570874	0.644113975
111	10.47705609	0.806892545
112	12.01054989	0.433691494
113	9.503228322	0.564664937
114	-7.16660483	0.720357855
115	-8.07129748	0.680215959

116	6.637683398	0.89797675
117	1.941674512	0.883420188
118	20.12509835	0.692821913
119	-4.02296296	0.960328352
120	0.000595776	0.994762538
121	-23.8500176	0.78386896
122	8.181503346	0.89595399
123	-8.03149424	0.067334796
124	43.34043787	0.476747529
125	-18.3721696	0.594859797
126	-1.91305609	0.218888813
127	12.64733889	0.72933296
128	1.277071995	0.97119732
129	-4.39893524	0.631746266
130	1.761590727	0.962720802
131	13.339371	0.619714737
132	3.512252105	0.453678537
133	11.98322486	0.680851672
134	25.54765063	0.23180867
135	3.687959926	0.458544371
136	25.43039633	0.278505829
137	-1.09259861	0.793002174
138	0.036364028	0.987921963
139	2.113813318	0.614408027

IDP	Beta	p-Value	NewOSA_LM3_PSYCH_MEDICATION_Summary
1	-15.5553998	0.736995378	
2	59.70811207	0.249059301	
3	-1.93313975	0.880135963	
4	-5.67439436	0.659208106	
5	61.15535837	0.117521383	
6	66.58882297	0.065377976	
7	84.52057397	0.026664962	
8	7.089200355	0.847087548	
9	28.59122949	0.047028762	
10	5.056059883	0.683545209	
11	11.03259064	0.391203106	
12	3.778765685	0.770109552	
13	-16.8431696	0.64642015	
14	-12.0576453	0.741634719	
15	-39.1745096	0.144010413	
16	-24.287665	0.357466482	
17	-2.63544055	0.706965413	
18	3.287573358	0.643572988	
19	-19.0641164	0.089863043	
20	-23.902058	0.046991112	
21	-4.65270098	0.603544288	
22	-2.65755138	0.718704347	
23	-21.7309821	0.249348336	
24	-11.7518593	0.530007994	
25	-10.5572845	0.569862154	
26	5.096065746	0.812466884	
27	-20.0567831	0.007680661	
28	-7.41183907	0.280217666	
29	5.694032109	0.750091515	
30	29.24009997	0.091434374	
31	18.3443631	0.199383598	
32	26.78886789	0.117695871	
33	-11.0716086	0.738143758	
34	-36.2662273	0.262034424	
35	2.158636282	0.923582118	
36	-19.3923555	0.379183597	
37	11.43392399	0.470464362	
38	-22.4358409	0.137180795	
39	24.76320737	0.226144307	
40	-34.4135872	0.167264749	
41	0.658641985	0.972558078	
42	-57.8578336	0.029360656	
43	-73.9332975	0.117933859	
44	52.16499701	0.284255454	
45	10.39421756	0.70297627	
46	-13.5939253	0.634722358	
47	19.13946555	0.186491765	
48	21.17287893	0.124309926	
49	1.034438365	0.893271672	
50	-13.4279544	0.089593896	
51	-4.74883714	0.725652286	
52	14.61495129	0.280200339	
53	-18.4295901	0.02114271	
54	-12.641674	0.081057339	
55	-18.3766209	0.302747576	
56	2.303987886	0.898944741	
57	-15.601955	0.519615222	

58	-10.1871074	0.705837885
59	-1.80271635	0.898134289
60	0.63293531	0.965674022
61	9.647185787	0.718393067
62	-1.49481636	0.957606227
63	16.70499024	0.076659054
64	15.49225915	0.142807885
65	-34.0207719	0.039461729
66	-10.5627802	0.493680343
67	12.59870452	0.184780524
68	1.221264279	0.904284708
69	1.864762376	0.7167313
70	1.929215935	0.651407392
71	13.96105787	0.426217946
72	19.99501615	0.267345517
73	-9.82836033	0.089482333
74	-10.2156362	0.052913273
75	23.32875102	0.050477672
76	9.091936427	0.369551626
77	18.34674316	0.085454094
78	-4.12833731	0.753290895
79	-10.8475872	0.429430202
80	-6.11596899	0.654118062
81	-2.92641863	0.624052574
82	-12.4520139	0.030592212
83	-24.1505453	0.041411266
84	-19.0671691	0.123601817
85	-1.31361992	0.898132002
86	6.839225801	0.470970821
87	-3.60916954	0.44493476
88	-4.20071509	0.378139626
89	-2.1790564	0.712391983
90	-7.14117532	0.103386979
91	-0.83294772	0.937557781
92	-6.24142494	0.370772667
93	6.716109287	0.014188144
94	3.616480608	0.326417112
95	1.379002474	0.96441763
96	-12.299686	0.679997808
97	18.20481753	0.008172441
98	21.21170755	0.003047617
99	76.26996149	3.36E-06
100	72.56859911	2.07E-05
101	33.95244944	0.001397
102	26.10038612	0.022332337
103	0.698496765	0.483276348
104	0.864739535	0.440740559
105	-10.1808673	0.246108072
106	-18.5420551	0.041224625
107	-12.2121807	0.01361288
108	-8.37175712	0.119831282
109	2.066818187	0.416010966
110	2.705487415	0.284976278
111	24.69317006	0.19839455
112	-16.5317492	0.016145108
113	-23.7428259	0.001318008
114	-11.7334933	0.190720739
115	-16.0260456	0.067701739

116	-28.7771742	0.214614625
117	-11.2425683	0.058038418
118	-29.0036141	0.203748254
119	-49.0623431	0.175651183
120	-0.00046795	0.990816242
121	-68.803316	0.077325417
122	-88.3233676	0.001624397
123	-3.12422725	0.112116637
124	-71.83441	0.008470252
125	-48.9552935	0.001559717
126	-0.676262	0.331899497
127	-44.5017848	0.006569891
128	-47.8937414	0.00250472
129	-5.04687575	0.219614402
130	-64.3514716	0.000138244
131	-26.2352913	0.029342687
132	-1.48089015	0.480616365
133	-41.2846118	0.001560461
134	-18.0459482	0.059356062
135	-1.21364824	0.586044028
136	-30.0715951	0.004227446
137	-3.42071805	0.066640386
138	-1.8667179	0.082770256
139	-0.37637432	0.841277041

IDP	Beta	p-Value	NewOSA_LM3_SEX_Summary
1	-287.042746	1.07E-27	
2	-320.265072	1.42E-27	
3	-22.3146573	0.002166088	
4	9.697667542	0.184235324	
5	-323.439155	5.23E-48	
6	-220.675891	6.07E-27	
7	-391.713819	8.58E-73	
8	-361.939727	4.78E-67	
9	-65.1448887	1.61E-15	
10	-25.0273269	0.000378741	
11	-73.3447183	1.08E-23	
12	-82.6036159	2.54E-29	
13	-179.110773	8.78E-18	
14	-160.029872	1.31E-14	
15	306.705723	1.09E-89	
16	189.8174101	1.09E-36	
17	10.09148881	0.011213533	
18	-16.0878296	6.65E-05	
19	-22.1496355	0.000517429	
20	-65.0133416	1.88E-21	
21	52.12574187	1.30E-24	
22	3.825021096	0.361059072	
23	-144.485195	2.29E-41	
24	-222.565239	9.50E-97	
25	-131.406145	1.51E-35	
26	54.28467042	8.52E-06	
27	60.67467709	1.16E-45	
28	56.08205179	8.15E-47	
29	54.20164519	9.28E-08	
30	16.46934444	0.093950337	
31	54.04720642	2.76E-11	
32	158.6607288	1.23E-59	
33	-565.230838	1.53E-195	
34	-615.238016	1.30E-241	
35	-170.718506	1.28E-40	
36	-133.897857	1.17E-26	
37	-114.639849	4.01E-37	
38	-155.279484	5.32E-73	
39	-56.3361981	1.23E-06	
40	-63.2456543	7.79E-06	
41	-111.530859	1.15E-24	
42	-104.303888	4.62E-12	
43	-986.088973	1.13E-288	
44	-474.21645	1.35E-65	
45	-127.670614	1.63E-16	
46	75.6093343	3.25E-06	
47	-32.8486033	6.50E-05	
48	-44.5679946	1.21E-08	
49	6.610922543	0.130895098	
50	2.260430538	0.614639298	
51	-56.525732	1.90E-13	
52	-47.0908369	8.89E-10	
53	-19.6802895	1.45E-05	
54	9.449241695	0.021597939	
55	-100.781687	2.56E-23	
56	-144.430259	1.54E-44	
57	-181.529049	1.15E-39	

58	-137.107776	3.76E-19
59	51.15893078	1.57E-10
60	16.27499529	0.05123842
61	-263.775797	3.07E-67
62	-326.215182	3.59E-92
63	-56.8462603	2.86E-26
64	-48.8644686	4.00E-16
65	38.3234447	4.38E-05
66	46.70227276	9.80E-08
67	119.600791	4.47E-108
68	152.4106482	3.53E-152
69	15.12748978	2.17E-07
70	-0.0756746	0.975091482
71	230.9650019	7.13E-118
72	202.506709	1.36E-86
73	48.20012428	1.46E-48
74	71.74485056	1.65E-125
75	54.86951608	5.48E-16
76	44.99658058	5.30E-15
77	-10.363042	0.086985699
78	55.58133479	9.23E-14
79	-1.82149514	0.815171189
80	-11.779057	0.128446602
81	-14.473335	1.96E-05
82	-5.99822581	0.066486875
83	-69.7267623	3.61E-25
84	-131.82775	5.10E-78
85	58.33503169	1.42E-23
86	49.74122717	2.70E-20
87	-60.9895037	1.81E-113
88	-60.9716196	1.74E-111
89	3.997170716	0.233512659
90	-20.4379166	2.26E-16
91	138.4313763	2.23E-115
92	47.31189422	7.51E-33
93	-8.2816249	9.99E-08
94	-16.2303564	8.82E-15
95	-71.9879828	4.09E-05
96	247.7431127	2.45E-48
97	16.51722713	2.36E-05
98	43.46196106	1.19E-26
99	-126.899264	3.80E-42
100	-111.008723	2.06E-30
101	57.30773711	2.20E-21
102	58.88718329	1.14E-19
103	-2.98545624	1.31E-07
104	-1.98015309	0.001871105
105	27.46923975	3.55E-08
106	24.42967511	2.16E-06
107	10.14423381	0.000305493
108	-14.1741814	3.50E-06
109	-2.50836468	0.082022332
110	4.579986657	0.001429629
111	-78.2027544	7.34E-13
112	7.419795694	0.057141234
113	4.562945233	0.276757383
114	34.64977354	1.01E-11
115	32.88999901	4.02E-11

116	-127.577888	3.50E-22
117	-37.3335856	1.62E-28
118	-102.722168	2.26E-15
119	-226.480148	3.73E-28
120	-0.0999395	1.49E-05
121	-82.2745825	0.000198411
122	-83.5297682	1.52E-07
123	-8.91099173	1.47E-15
124	-89.3524106	8.02E-09
125	-54.2739657	6.53E-10
126	-8.09395859	2.30E-92
127	-45.9103459	7.85E-07
128	-115.17487	1.89E-37
129	-35.9843187	1.98E-53
130	-172.883668	2.34E-72
131	-56.6592869	1.18E-16
132	-18.1029792	6.59E-52
133	-125.433665	5.29E-64
134	-145.632147	2.33E-156
135	-21.229141	6.63E-63
136	-163.149073	1.64E-162
137	8.478652954	1.20E-15
138	-19.6143679	2.75E-222
139	2.460841814	0.021071123

IDP	Beta	p-Value	NewOSA_LM3_SHORT_SLEEP_DURATION_Summary
1	0.13750567	0.995800442	
2	-82.5606758	0.004719603	
3	-5.02610358	0.486981382	
4	-1.33301167	0.854265086	
5	13.64332525	0.535831824	
6	-10.3512008	0.611548543	
7	-68.6406798	0.001417182	
8	-14.5195484	0.483782763	
9	1.748539288	0.829494147	
10	-4.12798867	0.555154491	
11	-0.99026346	0.891464359	
12	-10.6731961	0.143357488	
13	-14.6614209	0.478960197	
14	-5.45622451	0.791390492	
15	-0.2140531	0.988707054	
16	-4.75655428	0.749334929	
17	1.920604018	0.627157356	
18	-5.23958198	0.191061926	
19	16.8078834	0.008021453	
20	9.268267709	0.172054662	
21	-6.86482988	0.174319221	
22	-14.6440829	0.000433885	
23	-5.93512885	0.576977906	
24	-0.29412175	0.977768892	
25	-4.98658917	0.634162221	
26	7.315915573	0.545943462	
27	-3.33845133	0.431429747	
28	-4.03479255	0.297319116	
29	-21.3134069	0.034537788	
30	-8.91208048	0.361708549	
31	-16.8315541	0.036836062	
32	-2.93590934	0.761125608	
33	-11.4759702	0.538970777	
34	12.23902189	0.502160456	
35	-11.2325951	0.376184438	
36	3.833414357	0.757923557	
37	-4.89937687	0.583486856	
38	-7.87529821	0.354948818	
39	19.91939696	0.084323307	
40	9.154888624	0.514798847	
41	26.74719729	0.013262092	
42	7.936365266	0.596213327	
43	-7.90029313	0.767063671	
44	-20.407577	0.457650045	
45	-53.5734874	0.000493876	
46	-46.9732494	0.00360986	
47	-10.1104225	0.215999624	
48	-9.56222403	0.218451444	
49	3.195916294	0.462399048	
50	-0.48577372	0.913296093	
51	5.078708431	0.505810917	
52	6.945674179	0.362879983	
53	-14.2742219	0.001547108	
54	-10.7631944	0.008454019	
55	-4.02584165	0.688948006	
56	1.441634078	0.887962114	
57	-10.0008738	0.464280008	

58	-6.149842	0.686217562
59	-9.99377254	0.208299477
60	-17.6919471	0.032955121
61	-40.7163785	0.006971425
62	-42.4110043	0.00749965
63	-13.85908	0.009213231
64	-16.0431914	0.007136675
65	-18.2943365	0.049603412
66	-0.11669672	0.989303011
67	-10.979814	0.040453098
68	-14.3364086	0.012328107
69	-0.13140516	0.963843641
70	-1.04677535	0.663826211
71	-0.0537547	0.99566612
72	-5.16911173	0.611172376
73	-4.46432895	0.171445311
74	-5.42005188	0.068637802
75	-1.33883494	0.842247631
76	-3.52402272	0.537475681
77	-2.12215174	0.7243005
78	-5.67013589	0.444058227
79	-0.29943096	0.969152745
80	-15.0568824	0.050511888
81	-4.03815435	0.23050606
82	-2.06303864	0.525300879
83	-0.80076523	0.904567859
84	-6.88668291	0.324105354
85	-2.97083544	0.60772563
86	7.702545593	0.150023255
87	5.535076015	0.037804141
88	0.671694098	0.802699031
89	0.82699654	0.804093508
90	3.039953788	0.218989501
91	-0.73346079	0.902658138
92	2.384217265	0.544397013
93	-2.58922067	0.093668522
94	-4.57837376	0.027622624
95	-31.9041122	0.067277515
96	-37.337957	0.02642805
97	-1.8779397	0.628560799
98	-6.65851	0.099132025
99	17.78948949	0.054572968
100	11.22021826	0.243146781
101	8.281593443	0.166996886
102	12.09057065	0.060598123
103	0.591634022	0.292449584
104	0.255203957	0.68666076
105	0.978294012	0.843355891
106	3.828927688	0.454843894
107	2.954028026	0.289966447
108	-0.0590169	0.984488755
109	2.670491706	0.06243298
110	2.30702973	0.105995906
111	19.63098316	0.069864786
112	9.97413593	0.010075211
113	12.33341755	0.003093805
114	9.361175447	0.064202096
115	14.39315135	0.003625773

116	23.09440065	0.077447071
117	6.037310945	0.071136787
118	18.31563628	0.154745716
119	20.71052289	0.310792128
120	0.024813061	0.279208962
121	8.148481315	0.710701986
122	3.6111117	0.81928495
123	0.553022112	0.618051482
124	6.64780934	0.665745828
125	11.48372358	0.188271263
126	0.896641496	0.022559118
127	11.18725873	0.225727337
128	10.19105917	0.254099329
129	2.892167913	0.212318862
130	15.48791467	0.103844949
131	3.324696329	0.62444125
132	0.572508223	0.62878483
133	7.046324906	0.338429509
134	11.09033922	0.039930293
135	2.072913467	0.099133678
136	11.54737841	0.051456428
137	-1.76681083	0.09304281
138	2.688294183	9.47E-06
139	-0.43997333	0.678105288

IDP	Beta	p-Value	NewOSA_LM3_SLEEP_APNOEA_Summary
1	-363.675942	0.044959148	
2	134.5213581	0.507214555	
3	50.42798388	0.315109738	
4	-8.95580826	0.858919412	
5	135.3971585	0.376152742	
6	-209.789131	0.138192727	
7	-136.17571	0.361788574	
8	-282.876705	0.049420268	
9	-85.9471026	0.127345626	
10	-8.36155489	0.863315608	
11	-26.6106677	0.597392344	
12	-47.1990108	0.351259625	
13	-194.059742	0.17709837	
14	-110.11525	0.441956575	
15	-132.664623	0.206396777	
16	56.33678327	0.585689732	
17	-6.19502609	0.821455238	
18	36.14217729	0.193933561	
19	-44.4408866	0.312621495	
20	61.20573238	0.193947064	
21	0.731063606	0.983374876	
22	7.200840139	0.803177371	
23	34.03240964	0.645009992	
24	122.2538268	0.095251593	
25	177.7890588	0.014536567	
26	-6.03310686	0.94281938	
27	-10.8567091	0.712480702	
28	-32.4805822	0.226872337	
29	36.24327622	0.604632806	
30	35.92817136	0.596357329	
31	95.85725283	0.086807154	
32	117.7888048	0.07896314	
33	-76.377845	0.555887422	
34	94.85559332	0.453757511	
35	9.595185382	0.913292804	
36	-31.8320657	0.712396295	
37	28.8498777	0.641896154	
38	-6.91139689	0.906913039	
39	-86.5523402	0.279988536	
40	-100.570287	0.302684735	
41	-41.7577895	0.577553732	
42	18.96608444	0.855279853	
43	174.1290636	0.347010158	
44	232.7142243	0.222494	
45	92.62381112	0.385546988	
46	-123.832418	0.269073592	
47	-17.9743195	0.751376387	
48	-53.5506851	0.320850296	
49	-30.2598805	0.316220777	
50	3.385334919	0.912968166	
51	-7.07174662	0.893837246	
52	-55.698998	0.293261997	
53	39.35562946	0.208649101	
54	25.67386142	0.36555742	
55	-16.8548894	0.8092546	
56	-53.8454426	0.448492229	
57	-5.57163086	0.953170659	

58	7.528007502	0.94321387
59	19.36342339	0.725468087
60	30.40206622	0.597580469
61	-175.104981	0.094627663
62	-239.600705	0.029567347
63	-3.49252418	0.924690339
64	-34.4275398	0.405612047
65	84.66429134	0.190603425
66	64.16620819	0.288311475
67	12.43586382	0.73815304
68	-37.0609551	0.351388839
69	-50.1264105	0.012753183
70	-21.674425	0.19488501
71	72.11531948	0.293901743
72	62.43078772	0.376462055
73	-8.38605937	0.711359594
74	9.73631243	0.637546341
75	-1.35109717	0.976921223
76	16.20458017	0.682965868
77	-0.94517793	0.981947159
78	56.12117651	0.275218319
79	10.3187132	0.847780111
80	-129.458425	0.015441892
81	-30.6625154	0.189716003
82	11.40139732	0.613112925
83	-7.58013713	0.870146565
84	9.257443501	0.848583666
85	-47.3468916	0.238653827
86	-21.5488292	0.561876015
87	17.47555591	0.344883455
88	7.718023352	0.679222154
89	-39.0764402	0.091373417
90	1.510765954	0.92988293
91	-48.6059114	0.243041402
92	17.72582837	0.516247719
93	5.079542592	0.635708633
94	-7.81727206	0.588009995
95	-74.9604332	0.535736128
96	16.35877669	0.888584627
97	-25.2262718	0.349278063
98	1.253211062	0.964340754
99	15.53389828	0.80895008
100	-1.20389817	0.985608103
101	-16.7732718	0.686834741
102	-60.1222823	0.178944784
103	3.618058116	0.35375737
104	1.607950426	0.714296762
105	37.558442	0.274521018
106	16.93515214	0.63398318
107	-1.83619278	0.924518099
108	-8.36979811	0.691259727
109	10.15871802	0.307286667
110	2.343578079	0.813024831
111	-84.1165309	0.263203835
112	-2.65548119	0.92138828
113	-9.10481199	0.753078
114	-11.6050338	0.741028464
115	-4.35326866	0.899145763

116	-48.5683194	0.592737134
117	-1.13897848	0.960887942
118	-7.40420546	0.933963995
119	-58.313023	0.681027861
120	0.02263996	0.886910355
121	-204.281354	0.180454216
122	-147.900082	0.177729901
123	0.252492743	0.973841535
124	-174.04831	0.103299806
125	-124.898832	0.039291
126	-2.86748986	0.293408091
127	-150.612569	0.018818808
128	-138.826088	0.025244065
129	-22.8431411	0.155929819
130	-117.284197	0.076048694
131	-50.3919897	0.285160976
132	-5.60865246	0.495129115
133	-25.2667315	0.621003874
134	-7.243134	0.846743538
135	-2.40418654	0.78293575
136	7.598772784	0.853531794
137	-2.68211969	0.713425762
138	-1.11048244	0.792118565
139	3.680579396	0.616995359

IDP	Beta	p-Value	NewOSA_LM3_SLEEP_MEDICATION_Summary
1	71.10966817	0.725126166	
2	177.2008526	0.433350042	
3	50.80980966	0.364007219	
4	48.84786762	0.384579043	
5	-126.969262	0.456688679	
6	-150.46298	0.340271086	
7	-45.5216271	0.784537625	
8	60.21503781	0.707558151	
9	-82.2810992	0.190513932	
10	-63.3489264	0.242111111	
11	-34.9096173	0.534340487	
12	-138.629784	0.014076312	
13	268.7587103	0.093649177	
14	-84.2090444	0.597953036	
15	-13.0068939	0.911533929	
16	0.834027151	0.994225514	
17	-29.2718606	0.33891183	
18	23.53282862	0.448104511	
19	-68.0987549	0.16524511	
20	-2.86469086	0.956514893	
21	48.72157543	0.212958468	
22	-36.5880699	0.256062375	
23	-65.0020447	0.430009649	
24	-11.7014798	0.886120892	
25	12.78946382	0.874719518	
26	-69.4996572	0.458672985	
27	33.28670822	0.310897678	
28	3.619615424	0.903865951	
29	-4.34464271	0.955610439	
30	43.64941636	0.56387621	
31	61.06257795	0.327892413	
32	16.22455869	0.828191094	
33	-34.1303212	0.813404826	
34	132.2220311	0.348985877	
35	-294.624366	0.002715181	
36	-179.162867	0.062778406	
37	122.6041569	0.076325106	
38	136.3083316	0.038620085	
39	177.333493	0.047133993	
40	26.00773783	0.811069034	
41	-62.3928857	0.455459506	
42	-112.308792	0.332748293	
43	-81.3173221	0.693681201	
44	-220.225652	0.300495188	
45	87.5436971	0.462016528	
46	-139.030108	0.265777841	
47	-65.4081133	0.301145384	
48	-97.1033782	0.106446677	
49	22.40184339	0.50576408	
50	26.8255873	0.43732374	
51	-37.1479684	0.529545025	
52	-57.4719817	0.330760865	
53	-3.1824046	0.927348328	
54	-12.8210613	0.685297266	
55	3.059475329	0.968653996	
56	51.04739802	0.519299876	
57	59.9821622	0.570713801	

58	83.33311967	0.479462278
59	101.8471027	0.097630046
60	116.5536112	0.069530656
61	-24.4545896	0.834168585
62	-61.1928233	0.618205605
63	-46.8303105	0.255651472
64	21.17829293	0.646365443
65	-69.5727268	0.334763131
66	-2.56566491	0.969625674
67	47.97598453	0.247420555
68	41.21815446	0.352620404
69	2.051324885	0.927163909
70	14.18511536	0.44674574
71	-115.803643	0.130640332
72	-43.5649314	0.579916571
73	-6.39711607	0.800150583
74	8.796225439	0.70265773
75	-21.1146232	0.68514025
76	18.23819597	0.680151288
77	-89.2836568	0.055248013
78	51.97976467	0.364745638
79	1.339831068	0.982166455
80	-101.03086	0.090053585
81	6.587583119	0.800509684
82	-30.0278591	0.23235761
83	-85.6459569	0.097628732
84	-21.3737088	0.692592139
85	40.12904539	0.370411071
86	-60.0311522	0.147276063
87	-22.8729376	0.267534709
88	-9.19193327	0.658712033
89	-21.7827353	0.398665345
90	-10.4897888	0.583736019
91	18.13893555	0.696000428
92	-38.5325009	0.205681842
93	-3.07044762	0.797328536
94	3.421790413	0.83158941
95	-137.167816	0.309491392
96	107.9286572	0.407136761
97	1.429220609	0.962067211
98	16.77940768	0.59137766
99	38.55912253	0.590403785
100	75.43748532	0.310722648
101	36.25014579	0.434561482
102	73.16706083	0.142404589
103	1.602709301	0.712567316
104	8.094465637	0.098377563
105	24.8700054	0.516387623
106	29.33785068	0.459457811
107	-0.61681308	0.9772288
108	-7.76883669	0.74094318
109	3.209022252	0.772401108
110	12.57812404	0.254918124
111	-31.4621854	0.707422727
112	-13.893301	0.643326535
113	-3.55790242	0.912211524
114	-28.25703	0.470479208
115	-5.31978958	0.889526735

116	-144.846554	0.152546057
117	-15.4511306	0.550754142
118	-156.518961	0.116218257
119	-220.156185	0.16397788
120	-0.00172012	0.992268249
121	-330.928801	0.051671267
122	-196.733649	0.10787017
123	-22.9895336	0.007418549
124	-184.776144	0.120884265
125	-138.265789	0.040722576
126	-6.43756758	0.034395881
127	-98.4457287	0.168478173
128	-157.522196	0.022781227
129	-22.3244304	0.213633012
130	-125.825386	0.087830422
131	-84.0885343	0.109710921
132	-7.53486379	0.411123643
133	-76.569995	0.179023673
134	-44.0499801	0.29180771
135	-8.71748722	0.370314596
136	-36.0573705	0.432071221
137	-6.26999051	0.441314435
138	-4.48312901	0.339955004
139	-1.45952884	0.858831394

IDP	Beta	p-Value	NewOSA_LM3_SOCIOECONOMIC_STATUS_Summary
1	-40.712818	0.038170699	
2	-15.2655361	0.487022135	
3	7.777193887	0.152479562	
4	7.287800573	0.181596699	
5	11.78658073	0.476757885	
6	27.88089235	0.068807631	
7	9.905103405	0.540116531	
8	-3.02786831	0.845976756	
9	8.513900974	0.163053873	
10	0.641124541	0.902970074	
11	-6.58139084	0.227672072	
12	-1.33488165	0.807633571	
13	-37.5644516	0.01582543	
14	-28.4363007	0.066687345	
15	2.926372877	0.796858823	
16	-23.0845179	0.039135331	
17	2.674842756	0.368169182	
18	4.433372881	0.141117357	
19	10.55138688	0.026826191	
20	9.007539598	0.077471583	
21	-2.39650458	0.528122664	
22	0.371313757	0.90551626	
23	6.158901243	0.441292852	
24	-1.2112471	0.878666147	
25	0.672052093	0.932008988	
26	-5.28366463	0.561814575	
27	2.314471936	0.4680956	
28	-0.29252652	0.919933331	
29	-1.48924678	0.844232075	
30	-2.12907136	0.771913942	
31	3.8795053	0.522102444	
32	-3.69237747	0.61102627	
33	-1.14626078	0.934938079	
34	24.42672225	0.07479793	
35	11.14828897	0.242650104	
36	-6.9321701	0.458432958	
37	-6.72664423	0.316624649	
38	6.300871959	0.3248496	
39	-2.85134809	0.74238052	
40	-4.19997424	0.69097331	
41	9.642768297	0.234904781	
42	-2.6538822	0.813665162	
43	-13.0893652	0.513838055	
44	-9.67966831	0.639326489	
45	-0.77411266	0.946599524	
46	-18.0679848	0.1364132	
47	-11.1373754	0.069828729	
48	-14.3464173	0.014045132	
49	-6.0722296	0.063248742	
50	-1.73024395	0.605919044	
51	-7.20018956	0.209528902	
52	-0.26231463	0.963538634	
53	-9.31387947	0.005997848	
54	-6.59520419	0.031824525	
55	-6.38402334	0.398447977	
56	-5.62824043	0.464366529	
57	-3.21524809	0.754291574	

58	-3.22727702	0.777922989
59	-9.41753663	0.114724659
60	5.968957559	0.33847403
61	-22.7543625	0.044858347
62	-30.3532538	0.010906823
63	-2.6810231	0.502752842
64	-10.5632899	0.018446038
65	-15.3853321	0.028056022
66	-6.86764475	0.293903133
67	3.879991236	0.335406151
68	7.191414003	0.094913584
69	-2.57277653	0.237741938
70	-1.16799581	0.518833878
71	-9.93835293	0.181582502
72	-5.08236758	0.506072679
73	2.205429408	0.3687896
74	4.465850372	0.045962726
75	-5.86134718	0.246433774
76	-1.34341677	0.754499468
77	5.260232497	0.244814238
78	-8.18758172	0.141518698
79	-0.83379758	0.886093436
80	-17.4934489	0.002508243
81	-1.30672585	0.605738727
82	1.984544316	0.416311669
83	2.049241381	0.683160101
84	2.744622217	0.601126118
85	-3.83351237	0.378236293
86	-1.66526086	0.67887773
87	4.293642555	0.032092057
88	4.520510841	0.025298665
89	1.003684554	0.688803013
90	3.544684075	0.056563508
91	2.982443992	0.508244287
92	6.421939384	0.029860623
93	0.840821905	0.46894819
94	-2.04911573	0.189710273
95	-30.6661432	0.019300666
96	-15.8948396	0.208698187
97	0.105044394	0.971285187
98	-0.43999742	0.884737323
99	14.3756455	0.038789399
100	14.15631884	0.050124456
101	14.15391402	0.001679996
102	10.52979192	0.029712113
103	0.197275904	0.640514081
104	0.455970063	0.337673543
105	-1.02899349	0.782171716
106	-3.47900624	0.366343946
107	0.545880412	0.794760842
108	-3.20887202	0.159666039
109	-1.1483295	0.286499184
110	-1.16361961	0.278097833
111	-32.5884697	6.26E-05
112	-7.36768231	0.011451919
113	-7.26801447	0.020387947
114	-8.17578476	0.031534562
115	-5.27484847	0.156103864

116	-17.9943023	0.067227643
117	-2.66215072	0.289792399
118	-17.2965287	0.073840346
119	-3.42067005	0.823770147
120	-0.00273335	0.874005279
121	-13.0464976	0.429523181
122	1.37694126	0.907741556
123	-0.57825721	0.48795684
124	-5.17656511	0.654520708
125	-10.2227796	0.119216365
126	-0.58960232	0.046024569
127	-13.7269607	0.048000759
128	-17.7983426	0.008062047
129	-5.95168526	0.000640194
130	-22.5837948	0.001606107
131	-20.3502537	6.73E-05
132	-4.61447794	2.19E-07
133	-22.5616528	4.56E-05
134	-19.0265985	2.76E-06
135	-3.31426127	0.000452986
136	-19.5241622	1.19E-05
137	-0.98734381	0.21181242
138	-0.75093161	0.099768351
139	-1.45146562	0.068541761

IDP	Beta	p-Value	SeparateModels_sLM1_LONG_SLEEP_DURATION_Summary
1	-119.875626	0.190176809	
2	-247.882961	0.015192061	
3	-4.36245083	0.862783438	
4	14.24690766	0.57382167	
5	-164.033538	0.033113153	
6	-91.8492329	0.195180235	
7	-32.1957002	0.670027412	
8	-4.65084982	0.948550685	
9	-23.0616395	0.413268558	
10	-3.29256277	0.892290645	
11	-27.23864	0.280645804	
12	4.663847616	0.853704461	
13	-85.0289625	0.238095196	
14	-124.914482	0.081717681	
15	-137.429072	0.008815524	
16	-119.053809	0.021119351	
17	16.95190147	0.216411821	
18	-14.0991842	0.312669467	
19	-12.9876662	0.555204587	
20	-1.92425307	0.935144001	
21	-1.74868058	0.92058978	
22	-17.1893334	0.234561164	
23	-12.0105572	0.745454216	
24	29.71678667	0.418099297	
25	-9.71536239	0.789077017	
26	-71.2229883	0.090577289	
27	-12.1822825	0.410489628	
28	-11.7217008	0.384555564	
29	-6.47188302	0.854099373	
30	-20.2069613	0.551685543	
31	-35.2057942	0.209251994	
32	-17.8114286	0.595906483	
33	-170.712005	0.00848802	
34	-51.7854852	0.414886636	
35	-106.404852	0.015969882	
36	-15.6290502	0.716783721	
37	53.61387362	0.083080305	
38	-4.25879166	0.885194304	
39	48.37233091	0.227700355	
40	2.02783993	0.966803552	
41	42.3577711	0.259501946	
42	-12.8618995	0.80436674	
43	-159.252158	0.086887078	
44	-165.681692	0.083986037	
45	-28.3748867	0.595253569	
46	-28.0881044	0.616865337	
47	-27.0665048	0.34102153	
48	-8.48486124	0.753522012	
49	-31.9960216	0.034186028	
50	-36.0951224	0.019626961	
51	-16.7313384	0.528984046	
52	-17.5530595	0.508735854	
53	14.76718654	0.348275044	
54	23.61623928	0.098134606	
55	-82.248954	0.019247711	
56	-42.9650723	0.228318595	
57	69.11441042	0.14673941	

58	25.19980694	0.633569329
59	74.65430624	0.006884891
60	119.9165267	3.27E-05
61	-4.40347193	0.933235409
62	-99.4427193	0.071488775
63	-16.0207077	0.385955503
64	-33.9162521	0.102465008
65	-54.6394832	0.092677782
66	-71.7300167	0.017810316
67	-44.4091768	0.017154499
68	-29.3976957	0.139017381
69	-8.80165988	0.383202951
70	-14.8554341	0.076492925
71	16.65943038	0.628136432
72	-25.655145	0.468531963
73	-24.8123851	0.028795609
74	-11.3979814	0.269965362
75	-13.0660753	0.576398927
76	-6.57820644	0.741024729
77	-6.60234378	0.752358685
78	-3.83432236	0.881798051
79	-37.4423842	0.164700122
80	-43.223451	0.106207646
81	-25.3823171	0.030177756
82	-6.07919829	0.589937273
83	-14.9620566	0.519359774
84	0.427538207	0.985953569
85	9.764775312	0.626679246
86	10.26149706	0.580650835
87	11.87547472	0.198137163
88	8.009408143	0.390993222
89	-6.48484207	0.577224609
90	6.91037637	0.420489754
91	9.828691905	0.636827397
92	0.172192616	0.989931643
93	9.774828163	0.067973722
94	4.986133432	0.490425762
95	-32.4895261	0.592853088
96	-20.0470769	0.731562554
97	15.6388271	0.245719654
98	21.88076017	0.117875968
99	140.5685951	1.68E-05
100	131.2995835	0.000112504
101	53.79827745	0.010067071
102	40.00134609	0.075647162
103	6.297370695	0.001280977
104	8.992841608	4.94E-05
105	-27.3294962	0.112921513
106	-17.1667437	0.335551741
107	-13.6530364	0.160213228
108	-18.6815858	0.07783234
109	5.727326506	0.249336623
110	1.678754317	0.735278163
111	-4.73055975	0.898441612
112	10.41390643	0.439907078
113	9.341888062	0.520381888
114	-5.97764851	0.734480796
115	-3.94245647	0.818913687

116	-3.416793	0.940299194
117	-0.21649884	0.985137428
118	-4.80338358	0.914605759
119	-23.0128096	0.746054554
120	0.049116339	0.537788197
121	-37.3849388	0.62409685
122	57.82046527	0.292399002
123	-5.41569793	0.159853
124	70.11856477	0.18885579
125	5.417414292	0.858439962
126	-1.22377294	0.371246211
127	32.75925723	0.308305375
128	15.74175721	0.613794479
129	-7.0115266	0.385271033
130	10.16537486	0.759305067
131	17.2273689	0.466038418
132	2.473031621	0.548596133
133	8.352756806	0.743633646
134	23.57880607	0.208044607
135	4.129060375	0.344651722
136	24.10107125	0.241037728
137	-1.56382694	0.669094583
138	0.62261934	0.767317194
139	-0.34994036	0.924247942

IDP	Beta	p-Value	SeparateModels_sLM1_SHORT_SLEEP_DURATION_Summary
1	-14.9186326	0.516768487	
2	-97.0221717	0.000157821	
3	-2.58923694	0.683334415	
4	-1.43604826	0.821638863	
5	1.766526221	0.927293885	
6	-2.96452556	0.867949293	
7	-60.6980007	0.001401801	
8	-14.4198617	0.426286111	
9	0.017939101	0.997980727	
10	-4.18233966	0.494001499	
11	-3.31293915	0.601807198	
12	-5.22668007	0.411226435	
13	-18.8944147	0.297188164	
14	-9.88150895	0.583955197	
15	-14.0317851	0.287578645	
16	-24.7949957	0.056173021	
17	-0.1752508	0.959470676	
18	-7.88799757	0.024696735	
19	13.31870757	0.016137727	
20	8.167734035	0.169596194	
21	-7.77494826	0.077977998	
22	-12.8860427	0.000395246	
23	-5.53580392	0.551836506	
24	2.30351453	0.802901139	
25	-4.72513021	0.60489886	
26	2.016234714	0.848905332	
27	-6.49076328	0.081203359	
28	-2.51889693	0.457456034	
29	-20.1991702	0.022477984	
30	-7.87812604	0.356101378	
31	-15.9985425	0.023272439	
32	-12.4718888	0.139791973	
33	-4.942011	0.761882117	
34	15.87074739	0.320401902	
35	-10.0429605	0.365773941	
36	2.446446311	0.821354336	
37	2.058694799	0.791288655	
38	-1.75753844	0.812696051	
39	20.90464581	0.038171863	
40	13.34976813	0.275941356	
41	22.28614723	0.018321542	
42	17.43330814	0.181855879	
43	-13.8774824	0.552995614	
44	-17.0429123	0.479655745	
45	-48.3121589	0.000322553	
46	-49.6107804	0.000442111	
47	-7.55480526	0.290592423	
48	-8.30618752	0.221584103	
49	2.024099316	0.594179078	
50	1.834576433	0.637191095	
51	-0.44235726	0.947227304	
52	3.631708153	0.586664212	
53	-13.5730517	0.000608297	
54	-11.3200008	0.001619171	
55	-8.95425962	0.310885485	
56	-4.69426067	0.600689074	
57	-6.98553469	0.559725084	

58	-11.9434411	0.368949192
59	-8.89921316	0.20017663
60	-13.1962391	0.069077441
61	-40.3230724	0.002285785
62	-37.3356696	0.00712742
63	-12.6831201	0.0063487
64	-13.0719524	0.012322928
65	-17.1155445	0.036221192
66	-2.52502508	0.740111238
67	-13.9229532	0.002965283
68	-15.6663928	0.001718391
69	-2.07691023	0.413218917
70	-2.04100298	0.333138299
71	2.176176876	0.801354021
72	-4.52003783	0.611558585
73	-7.08525301	0.013045456
74	-5.67910895	0.028841746
75	-0.73247932	0.90088977
76	-3.72268804	0.457033167
77	-1.51730369	0.773077215
78	-2.79793287	0.666136035
79	0.692107122	0.918651928
80	-6.03420069	0.369805707
81	-5.38380756	0.067484786
82	-1.60167966	0.572323527
83	-3.94670993	0.499125454
84	-5.76365845	0.345276531
85	1.939781915	0.700804253
86	5.766649611	0.217010935
87	5.667670349	0.014597518
88	0.907065946	0.699262943
89	3.161031212	0.279909092
90	4.130867169	0.05550814
91	4.861381742	0.353084967
92	1.938212124	0.572184929
93	-2.66973808	0.047445105
94	-3.13947404	0.084227116
95	-19.9666671	0.191310713
96	-35.4825075	0.01576188
97	-0.17237913	0.959420633
98	-4.41581671	0.209497193
99	18.29296718	0.025935103
100	12.76128872	0.135507542
101	13.14046616	0.012431922
102	16.02321054	0.004659551
103	0.15677951	0.749858116
104	0.048686299	0.930365425
105	1.615491348	0.709428163
106	4.224209036	0.346041405
107	3.18930551	0.192055489
108	1.091046637	0.682142251
109	3.45769744	0.005685114
110	2.499027595	0.045346336
111	29.77944354	0.001399716
112	12.69587308	0.000181172
113	13.0681925	0.000350027
114	14.160316	0.001399565
115	16.86982154	9.81E-05

116	30.57134127	0.007708886
117	4.998247525	0.087234882
118	28.21504181	0.012258631
119	7.852613232	0.660349827
120	0.003507978	0.861081145
121	2.182997913	0.909403694
122	4.633881163	0.737214627
123	1.093173262	0.259225563
124	10.47471277	0.435061976
125	9.156371843	0.230621359
126	0.868829272	0.011595253
127	9.940425238	0.218958576
128	14.72419406	0.060510293
129	3.186636111	0.116622032
130	15.28704621	0.066919626
131	7.893266759	0.184143304
132	1.068039787	0.302922228
133	11.37032466	0.076676392
134	14.04655001	0.002861441
135	2.588769944	0.018473726
136	15.24000411	0.003199839
137	-0.94561832	0.304101756
138	2.718907896	2.79E-07
139	-0.33278744	0.71916598

IDP	Beta	p-Value	SeparateModels_sLM2_EXCESSIVE_DAYTIME_SLEEPINESS_
1	10.06255293	0.667429868	
2	-45.774556	0.079885946	
3	-16.4940304	0.010666828	
4	-5.88208286	0.364232822	
5	-43.2682594	0.028087569	
6	-17.4134163	0.33725148	
7	-43.5362995	0.024374517	
8	-22.4850788	0.222862356	
9	-2.0159235	0.779901224	
10	-5.94203925	0.339666804	
11	-7.3791001	0.253450748	
12	-13.0552834	0.043708031	
13	-18.8484448	0.306861539	
14	-8.40438697	0.647217103	
15	-7.88398953	0.557165297	
16	-2.52578001	0.848424179	
17	-3.56235909	0.310108994	
18	-3.38598469	0.343463733	
19	4.263303864	0.44925796	
20	8.900130822	0.141393447	
21	-5.72824292	0.201972572	
22	-3.33773581	0.367213679	
23	-0.42936192	0.963831295	
24	-13.2727307	0.15761751	
25	13.89575827	0.134905979	
26	19.61201893	0.068626684	
27	0.856355991	0.821162418	
28	1.955379534	0.57087203	
29	-1.41027982	0.875590968	
30	-6.78206306	0.435037409	
31	3.603996316	0.61552624	
32	-8.77786509	0.307193294	
33	17.67641037	0.286950591	
34	33.16919817	0.041296801	
35	3.927618902	0.728204058	
36	10.90524179	0.322649895	
37	6.172684791	0.43560485	
38	6.218541592	0.410058109	
39	20.83115459	0.042383817	
40	11.47236175	0.357587958	
41	14.72540754	0.125634847	
42	4.152566825	0.754683407	
43	1.3842848	0.953631356	
44	-12.9099669	0.59881793	
45	-1.76158341	0.897482618	
46	-2.22224963	0.877110684	
47	-22.3110223	0.002163082	
48	-17.3897845	0.011916314	
49	-4.67137068	0.226992614	
50	-2.78399773	0.481935382	
51	-5.53480362	0.415788306	
52	-9.99361228	0.141549298	
53	-3.01757883	0.454006105	
54	1.892999808	0.604506505	
55	-31.64397	0.000433344	
56	-35.4024027	0.000104876	
57	-7.28404275	0.5501266	

58	-7.38260813	0.585281138
59	-0.85803345	0.903414154
60	4.908795598	0.506516741
61	18.71091811	0.164307729
62	14.51042271	0.304185945
63	-3.69112566	0.435152935
64	-8.50303224	0.109694874
65	-12.0712006	0.14667545
66	-2.5404339	0.742990636
67	3.342239265	0.483450003
68	0.67468405	0.894470526
69	-0.00475694	0.998530731
70	2.525390722	0.239349336
71	5.962393888	0.498192386
72	-0.84082553	0.926041515
73	-1.52350576	0.599958572
74	0.498885507	0.850367975
75	4.148822375	0.488237786
76	-2.83251044	0.578178898
77	0.916465833	0.864116584
78	-7.25974795	0.271322804
79	-3.8348475	0.57818816
80	-22.0005403	0.001313303
81	-7.10460848	0.017752167
82	-1.79337972	0.534462534
83	7.292923104	0.219759092
84	3.203821939	0.606210991
85	12.78314867	0.012841032
86	7.085487621	0.136104088
87	0.261092956	0.911980533
88	-3.35793785	0.159947079
89	5.37760271	0.070881509
90	2.372652235	0.279837791
91	8.801451806	0.098531036
92	7.757637203	0.026318606
93	-1.99715429	0.145117146
94	-1.46007183	0.430097573
95	-58.0885016	0.00018715
96	-32.7396139	0.028599747
97	4.555811582	0.186396249
98	1.345550654	0.707122139
99	21.09606689	0.011631397
100	27.68294195	0.001466023
101	12.07839759	0.023982517
102	8.919951558	0.121691479
103	0.828208403	0.097983005
104	0.502364826	0.375741543
105	0.83328797	0.85020926
106	1.626612915	0.721445826
107	-3.6715594	0.140050867
108	-1.21862848	0.653107194
109	0.071883244	0.954954193
110	-1.60769795	0.205818456
111	4.853214499	0.608966711
112	-0.79144207	0.818632642
113	-0.61463442	0.868780894
114	-0.61322547	0.891874526
115	0.54759164	0.901139379

116	-25.122836	0.031434378
117	-3.65958923	0.218570417
118	-22.9996989	0.04484691
119	-45.3478784	0.012645347
120	-0.00792098	0.697819168
121	-56.440488	0.003840363
122	-15.1902043	0.279790982
123	-1.85109828	0.060494159
124	-12.3633219	0.365336895
125	-17.5058079	0.024318897
126	-0.40589235	0.246593212
127	-6.19371035	0.45168254
128	-16.8443476	0.034860151
129	-3.84167553	0.063066177
130	-19.296609	0.023052775
131	-8.77438953	0.146873236
132	0.569120507	0.589616397
133	-24.4160411	0.000187262
134	-5.77558007	0.228282229
135	-0.47086329	0.67372256
136	-10.1939403	0.052697633
137	0.741611821	0.428396818
138	-1.36867568	0.01106654
139	-0.01029053	0.991283029

IDP	Beta	p-Value	SeparateModels_sLM3_EARLY_CHRONOTYPE_Summary
1	-17.7653309	0.421444861	
2	-44.4003327	0.07182327	
3	-9.62570391	0.11431055	
4	-7.67552962	0.209588359	
5	-8.85994566	0.633700819	
6	-3.1058799	0.856069931	
7	-22.0688521	0.226538998	
8	-17.2784847	0.320844195	
9	-11.4560547	0.092383957	
10	-12.6636963	0.031036696	
11	-4.06133008	0.505321092	
12	-5.39170981	0.377365183	
13	-26.0075516	0.135126729	
14	-7.90756551	0.648177037	
15	-33.8634934	0.007532808	
16	-19.0468903	0.126655904	
17	-5.41485513	0.10204932	
18	-9.10946791	0.006912379	
19	-3.67833211	0.489041961	
20	-2.59208127	0.649912627	
21	-4.25407747	0.315267586	
22	-1.38855735	0.690965989	
23	8.585037671	0.336614342	
24	-6.78625888	0.443872328	
25	-15.5848208	0.075574406	
26	-7.59145319	0.455119336	
27	4.336308337	0.225110309	
28	5.784727695	0.075584429	
29	13.43182201	0.11405692	
30	-5.71221816	0.48596933	
31	-7.72942525	0.253690921	
32	-7.08302963	0.382549427	
33	27.15656489	0.082934847	
34	3.158388341	0.836856866	
35	0.97254839	0.927340636	
36	9.580271237	0.357178132	
37	19.01485102	0.010920494	
38	9.460723619	0.184106171	
39	0.722888097	0.940500417	
40	-13.9920771	0.234417342	
41	-6.58755287	0.467798105	
42	-5.90470018	0.637746422	
43	-35.2137639	0.116987075	
44	5.278277957	0.819688957	
45	-12.7186624	0.324193217	
46	-9.35039802	0.490507898	
47	-0.96027118	0.888759295	
48	-0.64380918	0.921414099	
49	-2.33870501	0.521523471	
50	-0.34605914	0.926193371	
51	-4.72650545	0.461477357	
52	-3.95069122	0.537987706	
53	-8.65540962	0.022836171	
54	-5.3858	0.11837044	
55	-9.56935406	0.259485048	
56	-8.03656823	0.350789683	
57	28.93950176	0.01186516	

58	21.40918727	0.093491309
59	-15.744422	0.018283978
60	-11.8108059	0.090306738
61	-13.9850988	0.270653451
62	10.55014755	0.428542645
63	-2.01390328	0.651821611
64	10.11812502	0.043682323
65	-31.9842175	4.59E-05
66	-21.915795	0.002719036
67	-5.9302187	0.187597167
68	-8.88084683	0.064238056
69	-5.36361008	0.027769416
70	-4.56480709	0.024198894
71	6.293647671	0.44864613
72	0.341861983	0.968096747
73	0.513755447	0.851297297
74	1.668813382	0.503662041
75	-4.08922791	0.469082398
76	-2.9417216	0.540545203
77	-4.44389682	0.379184567
78	7.194139743	0.24801273
79	1.738490944	0.789370891
80	-2.88923531	0.654784754
81	-4.69222521	0.097067767
82	-4.32436085	0.112419909
83	-4.29079629	0.444191912
84	4.470006799	0.445942245
85	3.280506014	0.498629687
86	-0.03816964	0.993211137
87	-0.86357359	0.698401043
88	-5.58806563	0.013201859
89	1.934669366	0.491063735
90	1.527636898	0.460905853
91	-0.9509511	0.849975903
92	-3.13997321	0.340670115
93	-0.48038916	0.710345964
94	1.932117007	0.268513122
95	10.15807967	0.488787189
96	-10.0963044	0.474403694
97	-9.15511349	0.004893308
98	-11.0253875	0.001103156
99	-7.33309452	0.352657286
100	-8.75999902	0.286048985
101	-7.71050439	0.126719203
102	-11.1389573	0.040536646
103	0.565848289	0.230899635
104	0.736100331	0.168997357
105	-3.32283429	0.424822643
106	-5.62875734	0.191061416
107	-4.19491654	0.07399319
108	-1.73692854	0.497220181
109	0.275495551	0.818537916
110	0.441120023	0.712976594
111	6.245460412	0.485404177
112	8.064887374	0.013267982
113	7.144018932	0.04183404
114	6.56874976	0.122804197
115	7.251282989	0.081286098

116	12.51607713	0.256005003
117	6.250345242	0.025943436
118	19.70458734	0.068557083
119	10.62605605	0.535783028
120	0.004508847	0.814820152
121	10.46895463	0.569865645
122	-5.76394844	0.663853906
123	-0.27687531	0.766054695
124	-9.0948376	0.480377102
125	-3.3376898	0.649102161
126	0.385647908	0.24335578
127	-8.27432633	0.286653863
128	6.48239247	0.389519888
129	1.588141225	0.415485543
130	1.347635496	0.866435479
131	12.04724999	0.034783107
132	2.386143816	0.016537705
133	13.24570902	0.03174425
134	5.349032981	0.236990254
135	1.844246689	0.080522044
136	9.951284707	0.045033484
137	0.731171085	0.407960997
138	1.09220775	0.031674303
139	0.042205035	0.962122173

IDP	Beta	p-Value	SeparateModels_sLM3_LATE_CHRONOTYPE_Summary
1	-40.7841835	0.225787974	
2	-3.40531582	0.927793732	
3	-8.5350258	0.358103837	
4	-5.52558812	0.55329416	
5	0.420875739	0.988146396	
6	-10.6802313	0.682278741	
7	44.08739593	0.112832259	
8	58.02650639	0.028666863	
9	7.594945974	0.463987571	
10	9.766087092	0.275013992	
11	21.59481165	0.020093239	
12	20.92789725	0.024526775	
13	14.21050576	0.592065839	
14	-3.56227824	0.892681576	
15	-29.4375599	0.127327003	
16	-15.1392498	0.425552944	
17	-6.27338048	0.213780974	
18	-3.29535763	0.521309791	
19	-9.10965366	0.260792133	
20	-7.17261505	0.409764643	
21	-13.2151948	0.040616015	
22	-8.99871908	0.090860735	
23	-14.0487889	0.302070286	
24	-5.02679276	0.709715084	
25	12.4136758	0.352904351	
26	-6.15263585	0.691143867	
27	-5.26263431	0.333928034	
28	-1.68258892	0.734441321	
29	-3.38957584	0.793529847	
30	4.160478072	0.739087972	
31	11.20762604	0.277363935	
32	15.69657639	0.20406903	
33	-64.1573835	0.007178572	
34	-50.9631154	0.029210828	
35	-11.2461122	0.488882987	
36	4.154510266	0.793272516	
37	-2.32222428	0.838336197	
38	-15.1886782	0.161654409	
39	1.018447425	0.944975321	
40	-16.7146679	0.351201641	
41	-9.10146751	0.510289207	
42	3.811770566	0.841875726	
43	-30.8543391	0.367343062	
44	-8.43348861	0.811077895	
45	37.4985225	0.056433106	
46	1.298448459	0.949892457	
47	-8.17041211	0.434746748	
48	-4.08888769	0.680911602	
49	-9.64086857	0.082877005	
50	-11.5933221	0.041675061	
51	-5.1743255	0.59672111	
52	-13.9049503	0.154845517	
53	-14.1808563	0.014382697	
54	-8.38954259	0.110360698	
55	4.96936978	0.700737647	
56	-6.53047924	0.618747717	
57	31.92288475	0.068503941	

58	66.56109099	0.000621088
59	4.332107252	0.669981836
60	6.083337807	0.56692053
61	9.244828756	0.632701892
62	-7.6482999	0.706406883
63	7.132773295	0.294222701
64	10.12509919	0.185230215
65	-20.7541669	0.082568257
66	-8.39863906	0.450814508
67	-13.5867337	0.047543342
68	-18.0272193	0.013688058
69	-8.95930709	0.015845627
70	-5.38424889	0.081000902
71	-4.49477125	0.722480378
72	-3.48857598	0.788798736
73	-15.7713327	0.00015899
74	0.280455991	0.941199776
75	-9.84591403	0.252595183
76	-8.78796875	0.230178751
77	-0.52539497	0.945595914
78	4.708970103	0.619701084
79	-2.31227059	0.815616876
80	-1.58110412	0.872414505
81	-0.69344122	0.872140048
82	-2.97514888	0.473495547
83	-9.15832184	0.283790595
84	-10.9128707	0.221982522
85	-6.99986099	0.34333075
86	-7.30790937	0.284988091
87	6.644435193	0.050388491
88	-1.70597396	0.619478531
89	-1.00126649	0.815058425
90	-0.48409419	0.878116005
91	-2.89986457	0.705010409
92	-3.34874617	0.504801087
93	1.391587461	0.480126474
94	2.351183979	0.376840457
95	-17.4803999	0.434313713
96	-11.7762801	0.583959285
97	-5.20944564	0.293275205
98	-2.5078758	0.626158396
99	19.73528238	0.100653192
100	23.72618733	0.057908069
101	10.2550846	0.182498974
102	5.794974104	0.48431062
103	0.378955396	0.598470022
104	0.767917942	0.346317998
105	-11.3312121	0.074070432
106	-6.46596576	0.324258325
107	-3.02098926	0.398397985
108	-0.41206371	0.915817834
109	-2.74092167	0.134112524
110	-0.73072823	0.689196633
111	-16.7675608	0.218968475
112	-8.47122373	0.087763825
113	-10.5197061	0.049196119
114	-5.40120466	0.404971517
115	-2.82669833	0.655581079

116	-9.07771638	0.588708404
117	-7.11070645	0.096333382
118	-1.29087667	0.937579077
119	-35.7037972	0.172099875
120	-0.00335454	0.908948033
121	-35.3258372	0.208223127
122	-2.50237655	0.901446926
123	0.747509113	0.598039181
124	-0.51761283	0.978970117
125	-2.646625	0.812810413
126	-0.32646093	0.516866452
127	0.585241727	0.960550938
128	-6.66511464	0.561456708
129	-1.9345865	0.51503749
130	-15.0316468	0.21823432
131	-12.2827826	0.157794739
132	-2.37343093	0.117641404
133	-16.371379	0.081479428
134	-12.9278707	0.060686958
135	-0.44761149	0.780706061
136	-10.8387898	0.151903856
137	-2.77477943	0.039303608
138	-0.47665221	0.5382859
139	-3.79042303	0.005123981

IDP	Beta	p-Value	SeparateModels_sLM4_INSOMNIA_SYMPTOMS_Summary
1	-31.9894347	0.129725226	
2	-62.7485477	0.007743102	
3	7.036977184	0.226921856	
4	7.678062076	0.18893734	
5	0.765525998	0.965625014	
6	1.10577044	0.946111024	
7	-20.1219593	0.248470229	
8	-12.920478	0.437196174	
9	-0.78846901	0.903504766	
10	-1.47972415	0.791976819	
11	-1.51640035	0.794623584	
12	6.621418533	0.256531334	
13	-46.84988	0.004841123	
14	-33.0413739	0.045965202	
15	0.822393888	0.945845197	
16	4.519228188	0.70444468	
17	-2.21370387	0.484165973	
18	-5.36398675	0.095974173	
19	-7.40109422	0.145107712	
20	-1.3471531	0.804980671	
21	-2.87587223	0.477361364	
22	0.614363225	0.853935351	
23	3.20385449	0.707417763	
24	5.261331563	0.534375401	
25	-1.99055793	0.812230692	
26	-15.2009297	0.117475486	
27	0.851705875	0.803068567	
28	0.266791191	0.931645063	
29	8.756136642	0.280930789	
30	3.529608446	0.652263852	
31	-8.74794752	0.176330151	
32	-9.81266028	0.205446028	
33	-7.92994136	0.596198893	
34	5.907864072	0.686852845	
35	-6.21240494	0.542054155	
36	-1.01966644	0.918299542	
37	2.72076253	0.703073116	
38	3.368588419	0.62061004	
39	2.209873471	0.811233896	
40	3.716336511	0.740976157	
41	7.232235243	0.40408501	
42	-9.26306299	0.439431792	
43	-4.5454863	0.832272304	
44	22.97568761	0.299007982	
45	-2.43064406	0.84367578	
46	-19.8551409	0.125393076	
47	-2.88678103	0.659844919	
48	1.132679915	0.855842343	
49	-1.8346738	0.598668877	
50	3.611718577	0.311584282	
51	-8.39451215	0.170997043	
52	-7.92811291	0.195809499	
53	-11.9300592	0.001024265	
54	-8.83034041	0.00736167	
55	0.789014162	0.922476601	
56	-1.20086699	0.883974299	
57	6.828026182	0.534375748	

58	-4.32219664	0.72306259
59	-5.42202468	0.395002371
60	-0.19117766	0.977106916
61	-3.60958857	0.766017
62	-2.04159065	0.872601018
63	0.875396765	0.837340476
64	-1.41265999	0.768173571
65	-15.7285191	0.035932189
66	-9.44181406	0.176445419
67	1.609706556	0.708131167
68	2.996777946	0.513401649
69	-1.71699928	0.460961764
70	1.097011708	0.570760254
71	-0.61808739	0.937920084
72	-11.1797156	0.170983105
73	-1.78024062	0.496646836
74	-0.54018048	0.820755021
75	-1.51502332	0.778904172
76	0.240386273	0.958254674
77	-0.26730977	0.955845347
78	2.812265416	0.636454634
79	3.502288821	0.57324608
80	11.02116993	0.074215367
81	-2.73481103	0.311432676
82	1.666325271	0.522016839
83	-15.484916	0.003848043
84	-3.67305155	0.512122605
85	-2.18631093	0.636921763
86	-4.24647894	0.321776218
87	4.253212207	0.045776833
88	3.069680889	0.154179029
89	-1.2475591	0.642083529
90	2.009000792	0.310110501
91	-2.71530367	0.571861348
92	-6.78027195	0.031267164
93	0.210084773	0.865009035
94	2.45381965	0.141319522
95	-1.11570773	0.936570222
96	-8.40417292	0.533120001
97	1.065282203	0.731822018
98	-2.08417293	0.51857929
99	3.629028121	0.630215869
100	7.627495707	0.330939807
101	7.3013219	0.130127455
102	7.084516737	0.172726141
103	-0.83064639	0.065643406
104	-0.31280159	0.540691098
105	0.668726219	0.866497479
106	8.105931711	0.048747463
107	1.244316974	0.579097278
108	0.945525611	0.698894923
109	1.581120539	0.168147336
110	1.156401254	0.312785402
111	20.58046192	0.016115342
112	6.528643823	0.035887313
113	5.886411836	0.079250182
114	9.684771798	0.017247827
115	6.9218753	0.081546812

116	28.91368613	0.006020034
117	6.093313454	0.023065365
118	21.73436583	0.035485094
119	7.881372267	0.630732561
120	-0.03114028	0.090423969
121	12.65117114	0.472294895
122	-6.98406881	0.581504042
123	2.379197319	0.007443949
124	5.049177866	0.681749074
125	-1.06211701	0.879537979
126	0.676926496	0.032075799
127	-2.74790651	0.711104806
128	12.81019571	0.075093948
129	3.546188776	0.057020183
130	4.748372636	0.535079821
131	13.36618765	0.014236006
132	1.981909312	0.037196339
133	6.520884492	0.268491013
134	11.80674161	0.006294594
135	1.237005136	0.2198416
136	11.25103229	0.017698241
137	0.817012705	0.333170555
138	0.672594357	0.166110536
139	-0.87326843	0.30375806

IDP	Beta	p-Value	SeparateModels_sLM5_SLEEP_MEDICATION_Summary
1	-7.95217263	0.966122665	
2	148.8093302	0.476393954	
3	38.66763244	0.454041413	
4	50.06294865	0.334096615	
5	-27.6409864	0.860713637	
6	-129.488933	0.372109123	
7	-25.9525295	0.866707654	
8	16.20811065	0.912488341	
9	-31.7284903	0.582238071	
10	3.289478536	0.947287313	
11	-12.9120893	0.802632873	
12	-123.699735	0.016835865	
13	208.4571042	0.157502519	
14	-121.456885	0.408120765	
15	-26.5501987	0.804695151	
16	-16.9831901	0.872296169	
17	-37.3113777	0.183622096	
18	1.839559938	0.948672123	
19	-60.8349937	0.176862819	
20	-6.60817462	0.891369854	
21	46.31062617	0.196964599	
22	-31.6688614	0.284572999	
23	-61.1167894	0.419466409	
24	-12.1038284	0.87194971	
25	-26.2493192	0.723915095	
26	-92.0390525	0.285154568	
27	27.43018335	0.365120211	
28	11.24353278	0.68355242	
29	26.38594777	0.714082356	
30	41.36246294	0.551536382	
31	92.62438665	0.106445097	
32	48.49079344	0.480467	
33	-84.0694864	0.526447933	
34	91.26151251	0.482550318	
35	-244.709833	0.006762559	
36	-199.585017	0.023568517	
37	102.6773276	0.104781011	
38	151.8730972	0.011850627	
39	148.4250423	0.070469958	
40	107.2955612	0.281848225	
41	-59.5618253	0.43844246	
42	-70.9289221	0.504409239	
43	81.91795858	0.666911491	
44	-177.929007	0.364445533	
45	84.14404454	0.441438114	
46	-117.685145	0.305708518	
47	-69.7581391	0.230414014	
48	-88.6945956	0.108683864	
49	16.98024468	0.582813631	
50	7.416333093	0.814751739	
51	-26.9980849	0.619557343	
52	-62.3029305	0.251676053	
53	-8.73018046	0.786433337	
54	-6.48051964	0.824490109	
55	-22.4872552	0.754473706	
56	7.058735748	0.922943263	
57	44.06541385	0.65115268	

58	65.33923494	0.545796813
59	105.913027	0.060987073
60	110.1295911	0.06231083
61	-82.2661007	0.444395365
62	-87.0679638	0.440619522
63	-49.1615796	0.193573902
64	3.458326991	0.935147505
65	-114.220716	0.085844807
66	-5.16043749	0.933605622
67	29.89443331	0.433052001
68	32.5753833	0.423084642
69	7.91155034	0.701669785
70	23.40714482	0.172546942
71	-125.890131	0.073645509
72	-71.3901671	0.324233851
73	-11.5295577	0.619589095
74	14.09551717	0.504978561
75	-23.7677862	0.619438187
76	30.59760247	0.452478181
77	-42.4623894	0.321307482
78	74.6227037	0.157282288
79	39.96177474	0.468616236
80	-73.8594813	0.1773058
81	3.268815498	0.891484599
82	-17.4267908	0.450232636
83	-101.11347	0.033325091
84	-60.5456205	0.223042335
85	39.16741544	0.340331314
86	-72.0208281	0.05810243
87	-23.1589717	0.220049456
88	-9.54633899	0.617300713
89	-24.3327391	0.306671544
90	-24.7382839	0.158734109
91	13.02159653	0.759833055
92	-45.8939482	0.100224343
93	-7.07989564	0.518264468
94	-5.21800202	0.724317563
95	-25.7873475	0.835689699
96	145.6171373	0.223332407
97	1.118996116	0.967620959
98	22.06125562	0.440995535
99	15.67303445	0.814635337
100	31.81949516	0.647428666
101	24.75499161	0.562814699
102	51.05728406	0.267838057
103	0.395869141	0.921198008
104	5.511416541	0.224184225
105	15.29989224	0.664503277
106	14.54160753	0.690145227
107	0.118008789	0.99526693
108	-2.68322675	0.901490429
109	2.601138411	0.798214298
110	11.28536785	0.266660038
111	-16.3182266	0.829662657
112	-7.37385247	0.789300909
113	10.16309151	0.732592769
114	-39.8369246	0.269368419
115	-19.2898978	0.584149076

116	-108.967499	0.243130258
117	-10.1715731	0.668857836
118	-115.714892	0.206813628
119	-64.1614169	0.659017515
120	0.033744636	0.836097669
121	-213.062725	0.172264569
122	-161.662909	0.150235592
123	-18.638933	0.018069499
124	-116.887369	0.284403926
125	-111.02593	0.074024085
126	-5.8058887	0.038169972
127	-78.4981104	0.232845814
128	-121.680133	0.056594621
129	-15.413307	0.350953397
130	-103.871822	0.126007282
131	-53.2653883	0.270693003
132	-3.8230664	0.650403611
133	-69.0016841	0.186721715
134	-12.1595591	0.751048862
135	-2.02861136	0.820510963
136	-7.32341031	0.861800387
137	-6.94727001	0.353450717
138	-3.16463535	0.462513888
139	-3.16368808	0.674399951

IDP	Beta	p-Value	SeparateModels_sLM6_SLEEP_APNOEA_Summary
1	-178.15484	0.247740371	
2	-96.059744	0.576569066	
3	22.06922494	0.603707962	
4	29.9959156	0.482049187	
5	-59.9714405	0.643753463	
6	-121.021216	0.310914938	
7	-165.987391	0.192222111	
8	2.70992035	0.982192004	
9	37.7261296	0.426857737	
10	21.81836121	0.594247876	
11	-35.4792519	0.404130316	
12	1.299571976	0.975665597	
13	-3.72344219	0.975532856	
14	-125.671382	0.298470066	
15	-92.2851254	0.296446336	
16	-57.0935988	0.511548013	
17	9.485300473	0.681350642	
18	-14.6399746	0.533717782	
19	-17.5176611	0.636654891	
20	-41.0958613	0.302198461	
21	11.60570783	0.694483713	
22	-20.8589102	0.391891871	
23	2.219478808	0.971589128	
24	9.806780631	0.873953585	
25	20.78690383	0.734010095	
26	-31.2099843	0.659750423	
27	-18.646086	0.454558535	
28	-26.9823984	0.234716182	
29	-6.44351869	0.913452658	
30	-16.9347164	0.767116216	
31	-29.236565	0.535919795	
32	31.26538012	0.58052926	
33	-123.328266	0.258980634	
34	11.25335513	0.916230657	
35	-59.2315049	0.425868663	
36	-99.9537553	0.168406238	
37	32.87899404	0.528065544	
38	-114.240652	0.021477143	
39	-7.31121907	0.913809064	
40	-176.264751	0.031740548	
41	0.502013484	0.993670452	
42	10.60169906	0.903525706	
43	124.1661946	0.428093867	
44	215.8623342	0.181361729	
45	-27.4557864	0.760282763	
46	-77.6462028	0.411694168	
47	-37.2381995	0.436754509	
48	-70.7332311	0.120181171	
49	-53.8736289	0.034257365	
50	-25.0098566	0.33715064	
51	-9.3655952	0.834281784	
52	-45.9418944	0.304520253	
53	7.675313829	0.772304302	
54	1.332922917	0.955811387	
55	35.26413673	0.551330528	
56	26.41784666	0.660118087	
57	91.69067164	0.253076037	

58	143.5474969	0.106930487
59	-7.45939374	0.872653134
60	3.188490828	0.947730252
61	-55.6587269	0.529641018
62	46.81273237	0.614506827
63	32.07503012	0.302832706
64	22.52283561	0.519743027
65	7.216380203	0.895120042
66	17.01554606	0.738614978
67	-20.292531	0.517985371
68	-18.4248324	0.582046296
69	-18.55678	0.275071245
70	-22.7571514	0.107180459
71	-7.32188539	0.899432481
72	-29.7800304	0.617408611
73	7.767539903	0.684541593
74	29.9079844	0.085728757
75	56.97328905	0.148125463
76	-10.4225078	0.755899661
77	-65.8052586	0.061889261
78	23.98274587	0.580858153
79	-92.9615836	0.040557254
80	-65.6189447	0.145398808
81	-10.5725122	0.591951513
82	-9.68046835	0.610415019
83	-14.1171214	0.718158907
84	4.710821437	0.908315625
85	-56.8008567	0.093002412
86	-61.2926308	0.050114936
87	-1.32541566	0.932054517
88	5.43819763	0.729509859
89	-18.9872641	0.332563118
90	-2.79623319	0.846560289
91	-56.0938603	0.109662212
92	-31.0307787	0.176987646
93	5.262787869	0.559662164
94	0.924557405	0.939488426
95	-34.7715418	0.734059408
96	57.25569551	0.560826058
97	-5.3174695	0.814737413
98	6.01052804	0.798719417
99	-2.90459688	0.957907028
100	-15.4763578	0.786999787
101	2.113451234	0.952145458
102	-33.2826669	0.380258272
103	6.54198204	0.047041538
104	-0.0838485	0.982078883
105	-8.8590135	0.760324435
106	-30.4186459	0.311050297
107	-24.0723203	0.141574369
108	-36.2186565	0.042391683
109	2.130727397	0.799188525
110	-4.1520784	0.619585476
111	2.837806098	0.963750896
112	-4.45851257	0.844400384
113	-28.9522223	0.237045771
114	-4.76820231	0.872414322
115	-8.35304413	0.773416844

116	-108.000023	0.159939744
117	8.458607893	0.665690684
118	-23.033594	0.76018214
119	-142.410121	0.234136962
120	-0.02003387	0.881392284
121	-139.694014	0.27698472
122	-77.6389012	0.401295501
123	4.590283634	0.47941211
124	-55.9581974	0.533594439
125	-74.2607915	0.146643926
126	-1.55048134	0.501280151
127	-120.239288	0.026420323
128	-63.7338543	0.225139694
129	-9.03848935	0.506416812
130	-113.75809	0.041796295
131	-66.9226359	0.092744174
132	3.043118216	0.661231432
133	-95.7021276	0.026110324
134	-25.0099834	0.427966751
135	0.48915042	0.947013815
136	-10.3451573	0.765152099
137	-3.65772434	0.55287309
138	6.785337301	0.055664276
139	-11.4756219	0.064142219

IDP	Beta	p-Value	Interactions_sLM7_EARLY_CHRONOTYPE_AGE_Summary
1	-4.86788912	0.096548932	
2	-1.16488347	0.721525156	
3	-1.91498439	0.01779662	
4	-2.3528977	0.003717717	
5	-4.30498721	0.080729219	
6	-5.18146275	0.022473156	
7	-4.44677994	0.066009883	
8	-4.25838068	0.06493165	
9	0.15438604	0.864189649	
10	0.249063071	0.74902639	
11	0.103468181	0.89815361	
12	-1.01689411	0.209149908	
13	-0.93754592	0.684536171	
14	0.726966174	0.751710713	
15	-0.22448606	0.893706419	
16	-2.94391332	0.074961839	
17	-0.55873009	0.203284464	
18	-0.49333032	0.269816676	
19	1.843396317	0.008918935	
20	-0.24596034	0.745304657	
21	0.43026314	0.443585673	
22	-1.12236747	0.015341753	
23	2.080684772	0.079019786	
24	-0.32398748	0.782754181	
25	-1.64469378	0.157288351	
26	0.849726212	0.528301599	
27	0.190514977	0.687747514	
28	-0.48721899	0.258995048	
29	-0.34939444	0.756517505	
30	-1.003804	0.355754749	
31	0.330586296	0.712673932	
32	0.26290697	0.80689445	
33	-2.94308653	0.156365253	
34	0.109525338	0.957048047	
35	-0.85645127	0.54466533	
36	-1.11388458	0.419398786	
37	0.119609318	0.903884415	
38	-1.52373183	0.106573089	
39	-1.17506187	0.360129418	
40	-0.46366202	0.766316392	
41	-1.9211915	0.110275204	
42	0.220157671	0.894664693	
43	0.417758404	0.888429789	
44	-0.34384339	0.910812508	
45	-1.4100329	0.409657493	
46	-2.65725605	0.139385	
47	1.259757111	0.166343775	
48	0.773522587	0.371348564	
49	-0.40540683	0.401909662	
50	-0.71286968	0.150070506	
51	-0.31230326	0.713636369	
52	-0.46531919	0.584298189	
53	-0.33175072	0.510355366	
54	-0.24522517	0.591605936	
55	0.087504922	0.93799029	
56	-0.70346964	0.537748065	
57	-0.22938388	0.880437018	

58	0.630298233	0.709585955
59	0.016614494	0.985013859
60	-0.74586121	0.419668699
61	0.325606535	0.846606498
62	-0.04345613	0.980375935
63	0.20044836	0.734757068
64	0.364683082	0.583410789
65	-0.74169085	0.475723202
66	-0.2020014	0.834887098
67	0.858047926	0.150256082
68	0.216233493	0.73387707
69	-0.27088722	0.401840718
70	-0.27415718	0.307146665
71	0.06574307	0.952400464
72	1.095403631	0.333751545
73	0.173514334	0.63290944
74	0.415500167	0.209108308
75	-0.27685942	0.711616943
76	-0.38527521	0.545530699
77	-0.31069395	0.642882405
78	-0.03224853	0.968851003
79	-0.39337188	0.648468019
80	-0.2767016	0.746676092
81	-0.17745634	0.635929674
82	-0.65680932	0.068938266
83	-0.93394666	0.209054796
84	-1.39464602	0.072888209
85	0.334797073	0.602464523
86	0.006739893	0.990958073
87	-0.25190409	0.393956157
88	-0.16664589	0.577207902
89	-0.23670444	0.525164597
90	-0.16220912	0.554783404
91	0.421140236	0.527557341
92	0.001689249	0.996914852
93	-0.15906005	0.35362648
94	-0.20745058	0.370191377
95	0.004179454	0.998285628
96	3.482065783	0.062751622
97	-0.35377327	0.412141537
98	-0.29047741	0.516761419
99	-1.1886229	0.255452284
100	-0.86683302	0.425499124
101	-1.07102898	0.109432978
102	-1.40951529	0.050501478
103	0.068748282	0.272013845
104	0.077888443	0.272053504
105	-0.33575124	0.542988119
106	-0.5899126	0.301248888
107	-0.29476799	0.343623425
108	-0.55351945	0.102666831
109	0.003512494	0.982391213
110	0.011380536	0.9429166
111	1.315160859	0.267740772
112	0.419540321	0.33110596
113	0.454645292	0.328501086
114	0.240222956	0.670336099
115	0.482033858	0.38196118

116	-1.61708467	0.268176632
117	-0.34099447	0.35938322
118	-1.01673668	0.478328537
119	-1.05698829	0.642252354
120	-0.0056817	0.025976932
121	-1.321548	0.588432245
122	1.447499908	0.410302674
123	0.023623367	0.848079053
124	0.928287518	0.58688854
125	-0.27437077	0.777768178
126	-0.00191024	0.965223981
127	-0.35246586	0.732034249
128	-0.9109457	0.361443275
129	-0.0843942	0.744077052
130	-0.65725531	0.535920052
131	-0.01009884	0.989345096
132	-0.03362745	0.798858106
133	0.531413307	0.515469537
134	-0.44762536	0.455236327
135	-0.07807189	0.576775417
136	-0.47927221	0.466350453
137	0.000256667	0.998251904
138	0.058185564	0.387655607
139	-0.06697601	0.569749628

IDP	Beta	p-Value	Interactions_sLM7_EARLY_CHRONOTYPE_SEX_Summary
1	-4.66192556	0.91664819	
2	52.6201396	0.289729131	
3	-2.11587861	0.86328775	
4	-5.09067695	0.679765277	
5	-52.4043256	0.162110538	
6	-57.9916927	0.093010457	
7	-10.6663054	0.771836394	
8	-7.18675484	0.83769639	
9	20.08878795	0.143328709	
10	24.5382533	0.038209143	
11	10.5859122	0.389177193	
12	-3.43154892	0.780480316	
13	-49.3651455	0.159519606	
14	-33.521566	0.337387837	
15	-8.53685555	0.738281374	
16	16.08975506	0.522178991	
17	4.539705	0.496660678	
18	-6.71316922	0.323431541	
19	1.058658271	0.921324521	
20	-2.64830499	0.818095871	
21	12.48082267	0.143905956	
22	0.890318724	0.89936869	
23	17.97292672	0.318432697	
24	-14.7874311	0.407920447	
25	29.54344098	0.094818194	
26	-26.5476003	0.195138715	
27	0.269546318	0.970171859	
28	2.561941082	0.696311391	
29	2.407700394	0.888264493	
30	-0.96376535	0.953505827	
31	30.76579553	0.024220298	
32	-5.91307978	0.717716372	
33	-19.7102063	0.532484397	
34	18.88791524	0.541358401	
35	37.19012588	0.083680023	
36	46.79836207	0.025698132	
37	19.27335855	0.200716172	
38	16.51814956	0.249974008	
39	-29.0703166	0.136562444	
40	33.0095435	0.164129855	
41	-15.5709777	0.394699008	
42	18.04333139	0.475497441	
43	3.474110802	0.938847624	
44	-88.5596182	0.05781812	
45	-11.6905762	0.65305829	
46	1.219063624	0.964433154	
47	-12.5964283	0.362796471	
48	-7.7820846	0.554246901	
49	-3.50640329	0.633550284	
50	4.850520827	0.519581703	
51	-5.27133586	0.683775353	
52	-19.4360374	0.132909131	
53	6.368016677	0.406023644	
54	1.893109725	0.785347234	
55	4.745817897	0.781434178	
56	17.47781493	0.314049477	
57	25.15192667	0.278124831	

58	30.75718787	0.232084844
59	12.54084163	0.351174851
60	22.22314493	0.113852447
61	0.26590975	0.991711191
62	1.036267269	0.969232269
63	-1.02898518	0.908945835
64	-5.46295692	0.589048633
65	0.58381698	0.970551938
66	1.569193698	0.91520531
67	-13.7374459	0.129879201
68	-0.41514323	0.965764565
69	-12.9251709	0.008532287
70	-4.35933779	0.285607587
71	-24.8282582	0.138239428
72	-22.0836065	0.200054736
73	-3.39692406	0.538628191
74	5.134135683	0.3074563
75	-3.14778302	0.782245951
76	-1.28800772	0.894284923
77	-8.24663886	0.418348797
78	4.161553355	0.740367325
79	-39.1243711	0.002868908
80	-15.8515983	0.223654839
81	2.585616933	0.650135368
82	12.64659133	0.021286564
83	3.516606443	0.755776779
84	11.05880594	0.349678896
85	10.10510282	0.30124038
86	17.19374968	0.057299908
87	3.485699754	0.437939309
88	-1.34948571	0.766577495
89	3.109537184	0.583077147
90	4.163523934	0.318834479
91	-0.6049946	0.952412175
92	9.032067416	0.173993146
93	-0.50300295	0.847044232
94	0.169055806	0.961699484
95	20.17674186	0.495178792
96	-25.0152169	0.379323423
97	3.698964176	0.572833695
98	-5.21495987	0.444027224
99	-9.65974572	0.54336409
100	-0.74471801	0.964090057
101	6.510342571	0.522266463
102	7.480138263	0.494924627
103	0.598019394	0.529794432
104	0.989534975	0.358834187
105	-10.4757356	0.212013818
106	-1.08834052	0.900195011
107	0.412529328	0.930548535
108	3.690755642	0.474233384
109	1.129892315	0.640593595
110	3.530186112	0.144137153
111	26.71310127	0.138800605
112	-10.0610783	0.125371305
113	-9.18508281	0.194247627
114	-2.25175357	0.793021117
115	-7.8810665	0.347235731

116	-30.0826282	0.175571183
117	-4.95700468	0.380953607
118	-19.028828	0.382903703
119	19.61256748	0.570834064
120	0.067563297	0.08166731
121	27.36330602	0.461275067
122	-29.9799467	0.262128733
123	-0.93540393	0.617914288
124	-33.9491484	0.191319131
125	-20.4630977	0.166317217
126	-0.4534965	0.49610608
127	-23.2066424	0.13820406
128	-13.4525864	0.375487109
129	-1.83325518	0.640983154
130	-8.98620925	0.577856933
131	-13.4009671	0.243903358
132	1.070947134	0.593576119
133	-4.81503157	0.698393195
134	-2.89246239	0.751022449
135	0.259000401	0.90309697
136	5.078598433	0.611751633
137	0.822062821	0.644487285
138	0.427718479	0.676249725
139	0.998620557	0.577312441

IDP	Beta	p-Value	Interactions_sLM7_EXCESSIVE_DAYTIME_SLEEPINESS_AGE
1	-5.6124653	0.066560437	
2	0.732057844	0.830175217	
3	-0.6915755	0.412489281	
4	-0.99717695	0.239041282	
5	-3.72117229	0.148310085	
6	-4.47536861	0.059083546	
7	2.630227027	0.297777862	
8	-3.5864565	0.136621214	
9	0.118058495	0.900332134	
10	1.602310467	0.048756003	
11	-0.9106967	0.280714468	
12	1.178814554	0.16330134	
13	-1.96979277	0.413730081	
14	-3.40491485	0.155925424	
15	-0.53320954	0.761209943	
16	-0.31441658	0.855496145	
17	-0.27499388	0.548782532	
18	-0.56009107	0.230290706	
19	-0.76198969	0.300603845	
20	-0.5212368	0.509796062	
21	0.021889724	0.970227443	
22	-0.05890601	0.903030717	
23	-1.18107624	0.339741161	
24	-0.45732625	0.709385378	
25	-0.25195168	0.83565649	
26	-1.45138376	0.302373147	
27	-0.05211215	0.916163048	
28	0.211353242	0.639166642	
29	1.020675654	0.38578232	
30	0.945848673	0.404724002	
31	1.210395624	0.196674671	
32	1.360171426	0.225939486	
33	-1.71686717	0.428509613	
34	-3.81668935	0.072322411	
35	-1.55118879	0.293457882	
36	-1.59397886	0.268547241	
37	0.284520276	0.783278661	
38	-1.1402243	0.247541091	
39	0.239558957	0.858218237	
40	-1.47039208	0.366813767	
41	1.641488082	0.191374955	
42	0.700394006	0.686703658	
43	-10.3552658	0.000869886	
44	-1.88122848	0.557324689	
45	0.321033176	0.857350001	
46	-0.81362332	0.664752659	
47	0.618116243	0.515518478	
48	0.355201263	0.694267299	
49	-0.02936689	0.953636729	
50	0.270871922	0.600506606	
51	-0.6623226	0.456139324	
52	-1.25891152	0.156384746	
53	-1.11318282	0.034430613	
54	-0.77402075	0.104914663	
55	-2.31782375	0.048485505	
56	-2.60877181	0.028662297	
57	2.454679115	0.123258858	

58	2.964990546	0.093457112
59	-0.13908744	0.880317319
60	1.028670843	0.286551462
61	3.677408499	0.036440423
62	-2.16424383	0.240796849
63	1.680219984	0.00654381
64	0.739317783	0.287073618
65	0.250605563	0.817512564
66	-0.33426921	0.741194244
67	-1.67467996	0.007177981
68	-1.54623868	0.019926024
69	-0.61091387	0.070245685
70	-0.4160617	0.137811957
71	0.176398191	0.878112659
72	-2.12670466	0.072354531
73	-0.95125346	0.012168633
74	-0.61086977	0.077033146
75	0.283172009	0.717309399
76	0.125076309	0.850952567
77	-0.2619955	0.708112839
78	-0.04928477	0.954430463
79	-0.72898187	0.418538048
80	0.167976986	0.851056153
81	-0.1094148	0.779869616
82	0.329758353	0.38190149
83	-0.47133589	0.543827363
84	-0.50395619	0.534873544
85	0.566180013	0.398991408
86	0.996733512	0.108553483
87	0.218087857	0.479760926
88	-0.06866165	0.825921928
89	0.013254909	0.972821109
90	0.501159322	0.080601381
91	0.698168282	0.315947803
92	0.616819554	0.176402604
93	0.478782178	0.00750913
94	0.293833058	0.224232758
95	-1.3328302	0.511757009
96	-0.36918443	0.85014617
97	-0.08815038	0.844863592
98	0.051023476	0.913164097
99	4.45455158	4.50E-05
100	5.718214146	4.83E-07
101	-0.23654019	0.734969148
102	-0.40789784	0.587863254
103	0.234253173	0.000338945
104	0.312824519	2.41E-05
105	-2.0489733	0.000379027
106	-1.99610943	0.000810554
107	-0.18914269	0.560651888
108	-0.47814606	0.177029422
109	-0.32625011	0.049656383
110	-0.21221603	0.201069122
111	-1.35795835	0.273188723
112	-1.02514446	0.022973119
113	-0.93912054	0.053281651
114	-0.85953954	0.144719007
115	-1.33522164	0.020404157

116	-3.36561436	0.027340637
117	-1.4084252	0.000289485
118	-3.05752852	0.041202721
119	-0.82223888	0.729318737
120	-0.00369269	0.16584588
121	-0.23277914	0.927282611
122	-6.90432684	0.000169824
123	-0.54975033	1.97E-05
124	-4.23907986	0.017512114
125	-4.76947526	2.64E-06
126	-0.15753691	0.000576161
127	-3.95937382	0.000230676
128	-5.65059985	5.98E-08
129	-0.9614696	0.000369532
130	-4.98830611	6.87E-06
131	-3.46012483	1.18E-05
132	-0.36410025	0.008246575
133	-2.85023927	0.0008386
134	-2.29752353	0.000242991
135	-0.31464428	0.031266687
136	-2.22059541	0.001231673
137	-0.13263507	0.278326584
138	-0.20924512	0.002934323
139	-0.0191924	0.876058576

IDP	Beta	p-Value	Interactions_sLM7_EXCESSIVE_DAYTIME_SLEEPINESS_SEX
1	-23.7147132	0.612048592	
2	39.01027784	0.454641473	
3	-14.8058919	0.251049678	
4	-14.2248711	0.271864429	
5	59.3739727	0.131327944	
6	51.51242926	0.155210092	
7	-7.15563241	0.852981697	
8	2.911274266	0.936995168	
9	17.93477358	0.21324954	
10	-3.06247386	0.805356412	
11	-1.70229385	0.895053449	
12	-14.1674989	0.27304148	
13	-36.7915579	0.317925879	
14	-70.9367611	0.053128279	
15	40.98612453	0.126475295	
16	38.61069216	0.143467212	
17	-5.38093849	0.442761591	
18	10.59457239	0.137679089	
19	-6.00863228	0.593340426	
20	-4.37231867	0.717556555	
21	-7.41665143	0.408073654	
22	5.85942188	0.427881938	
23	-11.9890206	0.52609111	
24	-12.7036185	0.498243446	
25	27.3429637	0.140794972	
26	23.3296008	0.278122076	
27	-3.18909819	0.673430855	
28	-10.8105446	0.116673005	
29	10.46092648	0.560891104	
30	-13.1630501	0.448098479	
31	18.54055353	0.195737376	
32	7.338424155	0.669090604	
33	-45.5595129	0.169293107	
34	-71.3730774	0.027913944	
35	1.713503331	0.939481947	
36	19.49306635	0.37605969	
37	24.50163662	0.121255317	
38	-45.7854865	0.002385677	
39	-9.56216797	0.640864018	
40	-35.3130971	0.156225038	
41	4.893361591	0.798874391	
42	34.42087899	0.194721156	
43	-26.293941	0.580172963	
44	-43.9665832	0.369601325	
45	8.5413699	0.754383014	
46	-14.8775115	0.60417426	
47	-18.6324729	0.199722495	
48	-5.67002383	0.681451371	
49	-2.90576322	0.706654684	
50	-3.57570481	0.651093819	
51	10.46099966	0.44128795	
52	16.79916084	0.215968999	
53	6.565958648	0.414417049	
54	10.96850139	0.132782597	
55	-40.5546459	0.023915364	
56	-32.6254347	0.073417991	
57	-10.0671854	0.679219829	

58	-3.51124822	0.896598445
59	21.11511769	0.134823222
60	12.4576234	0.398480045
61	-4.7469049	0.859770191
62	20.99968824	0.456515635
63	-11.9852089	0.204447267
64	16.01356721	0.131431919
65	12.66574159	0.445503786
66	24.54659372	0.112598028
67	17.02862857	0.073700528
68	20.56699538	0.04280883
69	3.879369092	0.45200641
70	-0.13180416	0.975464474
71	1.115826451	0.949396082
72	-9.02554431	0.617852774
73	2.221348584	0.701688314
74	3.906920592	0.459403033
75	7.483830064	0.531324326
76	1.035238394	0.918958887
77	10.32626183	0.334366302
78	0.317241733	0.980801768
79	-5.65024338	0.681660519
80	8.666287665	0.526236965
81	5.016243724	0.401883546
82	-8.75158944	0.12898074
83	-10.8826754	0.359183553
84	5.24611753	0.672564789
85	-18.7625659	0.067477883
86	-11.2474111	0.236151987
87	-6.23074732	0.18655855
88	5.149094309	0.280587593
89	0.413280914	0.944594927
90	2.097210437	0.63240996
91	-10.0199748	0.346421883
92	3.376744074	0.628256308
93	1.266252838	0.643670469
94	3.410194193	0.356123838
95	-65.0127047	0.036293751
96	-30.4481752	0.30802424
97	3.277610988	0.63409125
98	4.826043876	0.499823895
99	-36.8259944	0.027312704
100	-28.1086312	0.105501411
101	-30.1523308	0.004759372
102	-33.9841334	0.003140387
103	-1.87074747	0.06115689
104	-1.52979201	0.176586354
105	-2.56664819	0.770824048
106	-2.7400281	0.763577398
107	-3.16554199	0.524065517
108	-4.59166467	0.396381132
109	-7.0791731	0.005330638
110	-6.59297627	0.009365941
111	23.38602601	0.217012958
112	6.078462662	0.377736313
113	6.628237005	0.372189214
114	9.55634695	0.288781532
115	1.506579849	0.864086231

116	-2.3499888	0.919709431
117	-1.33940143	0.821574286
118	-10.4688156	0.647455876
119	-42.9682709	0.236818054
120	-0.00366493	0.928309808
121	-16.1819791	0.678103874
122	50.24939183	0.073381235
123	-0.96667258	0.623385433
124	86.54707182	0.001508105
125	11.48017647	0.459458201
126	-0.55657206	0.426177791
127	19.61976081	0.232481675
128	-7.60214614	0.633295711
129	-4.52866033	0.272482366
130	-14.7876865	0.382985462
131	-27.109673	0.024731349
132	-1.69666112	0.420590612
133	-31.0575528	0.017273333
134	-19.9867918	0.036750984
135	-6.41514317	0.004071793
136	-17.3429073	0.098706307
137	1.813016317	0.332323862
138	-1.90263842	0.076808554
139	2.881330523	0.125569133

IDP	Beta	p-Value	Interactions_sLM7_INSOMNIA_SYMPTOMS_AGE_Summary
1	6.092386077	0.034559151	
2	7.163809055	0.025925529	
3	1.520931739	0.055789719	
4	1.488111156	0.062231249	
5	2.31064806	0.340799451	
6	1.189717418	0.594354198	
7	-0.0741153	0.975160248	
8	-0.37640172	0.86832162	
9	0.413602722	0.641469255	
10	0.63807071	0.404917255	
11	0.093973152	0.905964382	
12	0.497099512	0.532704439	
13	-0.47795049	0.833300327	
14	-2.28807162	0.311581044	
15	1.537600144	0.352370632	
16	-0.58983107	0.716935315	
17	0.10525458	0.807578233	
18	0.294901158	0.502654063	
19	0.062626434	0.928058108	
20	0.702009126	0.346121885	
21	0.678550287	0.21951486	
22	0.111525255	0.806615558	
23	0.221528279	0.849282547	
24	0.872057794	0.450739649	
25	0.458375917	0.688760468	
26	-0.7796147	0.556575252	
27	-0.61064424	0.190512221	
28	0.325869899	0.442969578	
29	-0.95116638	0.39103727	
30	0.507901953	0.634909025	
31	-0.5419626	0.539539558	
32	1.360760641	0.198577333	
33	-0.18466357	0.92798856	
34	-1.4914424	0.456104776	
35	-1.05017877	0.450355945	
36	0.702542364	0.60478956	
37	-1.60993343	0.09860698	
38	-0.4411269	0.634950327	
39	-1.3280061	0.29327251	
40	-1.33139537	0.385829852	
41	0.226052251	0.848566687	
42	2.236426055	0.171702356	
43	4.648189204	0.112695735	
44	7.097380004	0.018801462	
45	1.861398038	0.268708528	
46	-0.16178437	0.927134973	
47	-0.08628944	0.923251264	
48	0.55550783	0.51415078	
49	0.883956502	0.063281301	
50	-0.05613996	0.908299918	
51	-0.03717342	0.964594822	
52	0.328589637	0.69460969	
53	0.908738413	0.066901156	
54	1.168558844	0.009380481	
55	1.629203014	0.141057672	
56	2.2712119	0.043209745	
57	0.295051625	0.844134271	

58	0.062899091	0.969874527
59	-0.43250189	0.619275104
60	-1.22652109	0.177489541
61	1.394114853	0.399967586
62	1.11629934	0.520815291
63	-1.10383428	0.057974386
64	-0.09050925	0.889994417
65	3.055535572	0.002830865
66	1.227602275	0.198021469
67	0.466826415	0.426396062
68	-0.29527867	0.637093371
69	0.7537015	0.017777531
70	0.29079394	0.271012796
71	-0.39172429	0.717777523
72	-0.96581982	0.386469302
73	0.73191258	0.040628305
74	0.128880284	0.692174938
75	1.11114948	0.131633165
76	1.099562146	0.079596698
77	1.03942754	0.114955948
78	1.044589897	0.19867225
79	-0.06963635	0.934638399
80	1.206102402	0.152489287
81	0.214113814	0.561621776
82	0.423877688	0.232942486
83	0.287620654	0.694230942
84	0.189684506	0.804218552
85	-0.49174172	0.436917626
86	-0.06690206	0.908990611
87	0.13732118	0.636761677
88	-0.23975962	0.41505427
89	-0.16226957	0.658022155
90	-0.35461962	0.189497137
91	-0.44597453	0.4966171
92	-0.50915001	0.236297309
93	-0.15186113	0.368155485
94	-0.34074108	0.134732938
95	-1.75568118	0.359038943
96	2.52623193	0.170067622
97	0.220576991	0.603321646
98	-0.00554336	0.989968359
99	1.026594625	0.318236172
100	1.800871364	0.092492488
101	0.484179939	0.462110768
102	0.41111783	0.56213551
103	-0.06131472	0.319480039
104	-0.07170984	0.304142873
105	0.231324648	0.67018793
106	-0.06376163	0.909598314
107	-0.25028417	0.413853243
108	-0.30133821	0.366580938
109	-0.08679544	0.579430803
110	0.064354013	0.68073532
111	-1.8169041	0.119739053
112	0.194174981	0.64759794
113	0.415655938	0.363982125
114	0.270885988	0.625695808
115	0.131550892	0.808420465

116	1.116888001	0.43706765
117	-0.08264332	0.821411421
118	0.388011663	0.783354374
119	3.4141231	0.127310818
120	0.001380724	0.582416018
121	3.963144784	0.099157105
122	-2.60152855	0.132651111
123	-0.15221204	0.209730215
124	-2.36237653	0.159983544
125	0.013632845	0.98863028
126	0.034601554	0.422251626
127	-0.77706986	0.442996861
128	-0.2310679	0.814021581
129	0.14269897	0.574843503
130	-0.00592973	0.995472059
131	-0.3279264	0.659460968
132	-0.16425915	0.205916345
133	-0.76313287	0.342600592
134	-0.64174678	0.276654097
135	-0.08376861	0.542843845
136	-0.63844702	0.324103671
137	-0.28628336	0.013021992
138	0.12533858	0.058627285
139	-0.26083893	0.024482141

IDP	Beta	p-Value	Interactions_sLM7_INSOMNIA_SYMPTOMS_SEX_Summary
1	-37.9341766	0.392855367	
2	-141.286495	0.004343384	
3	-22.7108465	0.06368558	
4	-12.3704369	0.314206276	
5	0.21934641	0.995315385	
6	12.11186492	0.724834115	
7	-8.22178693	0.822548645	
8	56.42396384	0.106614141	
9	-1.88059418	0.890661328	
10	-17.3732226	0.140921141	
11	-1.96956779	0.872287493	
12	13.18481505	0.282646303	
13	-13.6283455	0.696789482	
14	9.476228176	0.785538171	
15	9.377807862	0.71266599	
16	-13.6424298	0.586119133	
17	-5.66252971	0.394921133	
18	-7.11941182	0.29339554	
19	-7.37502828	0.489975353	
20	8.214099694	0.474141901	
21	-12.6210723	0.138131311	
22	-3.37991552	0.630027645	
23	-19.4053697	0.279778416	
24	-7.32277867	0.680934669	
25	14.27678986	0.417942653	
26	12.59079011	0.537562798	
27	-4.04725968	0.573203249	
28	3.227001546	0.621817506	
29	12.20642393	0.474798993	
30	8.131061061	0.621619831	
31	4.849421676	0.721518262	
32	10.60967434	0.515156351	
33	-29.7514134	0.344463268	
34	22.57079225	0.463984802	
35	-41.5284542	0.052626027	
36	-39.5190775	0.058743233	
37	16.55882695	0.2700274	
38	11.11642878	0.437268967	
39	27.22305429	0.161872348	
40	6.681610042	0.777504524	
41	4.600776791	0.800786222	
42	-11.0596656	0.660770739	
43	7.415145089	0.869496692	
44	7.807337284	0.866732104	
45	-5.02417245	0.846304917	
46	-30.9742611	0.25563034	
47	-10.9652355	0.426692521	
48	-11.2100879	0.392671159	
49	-0.43868816	0.952278928	
50	-0.19418917	0.979362089	
51	-32.6350508	0.011404013	
52	-18.6571962	0.14779626	
53	5.035187519	0.509760893	
54	-2.81734265	0.684238417	
55	-39.538522	0.020385283	
56	-29.1195205	0.092382332	
57	11.9556054	0.604976932	

58	-4.94505874	0.847133318
59	6.079648558	0.650192408
60	6.047721501	0.665936961
61	-12.8704941	0.613897333
62	16.90340565	0.52785844
63	6.249220303	0.485858162
64	7.283267101	0.469901814
65	10.39631002	0.50951786
66	-1.32682011	0.92802352
67	5.959139605	0.509752762
68	-10.0426121	0.297519163
69	2.064338261	0.673374245
70	0.741428951	0.855408064
71	-2.84055223	0.864877467
72	-19.7108682	0.25114869
73	-2.03836095	0.711225252
74	3.658282981	0.465605881
75	7.546164623	0.506165029
76	-2.50739062	0.795205088
77	-8.03633443	0.428768423
78	9.568501516	0.444595136
79	-11.4665667	0.380600053
80	-8.313554	0.521945451
81	-10.7661957	0.058102282
82	-5.26255432	0.336286445
83	14.65580529	0.193397526
84	18.33327407	0.119806805
85	18.24933547	0.061042021
86	-3.43011378	0.70355015
87	0.47050992	0.916331765
88	3.339568648	0.461066911
89	-0.11264808	0.984082065
90	7.974121179	0.055417511
91	10.54668265	0.296562529
92	7.460962881	0.2598339
93	-1.40906079	0.587708351
94	1.044908534	0.765849828
95	-54.4526573	0.064751283
96	-42.3660676	0.135197987
97	-2.01885481	0.75747678
98	-0.64987858	0.923754937
99	-3.77774002	0.811514981
100	-7.67683099	0.64145995
101	-18.9890318	0.061133985
102	-16.4749242	0.131497479
103	-1.01179886	0.286138664
104	-0.51033516	0.63491354
105	5.44688675	0.51497462
106	0.097014595	0.991050278
107	-7.59749699	0.107293426
108	-4.71667893	0.358829918
109	-3.24901011	0.177987185
110	-2.52687572	0.294189971
111	8.767859713	0.625904518
112	5.2830744	0.419384473
113	6.110844645	0.386191115
114	-0.58104863	0.94583718
115	9.823651357	0.239760154

116	-18.8458903	0.394533691
117	-1.14028309	0.839743939
118	-20.2507442	0.351487266
119	-64.0260396	0.063369103
120	-0.05273359	0.172721157
121	-75.0325587	0.042666187
122	-28.7729255	0.280212018
123	-2.97849627	0.111024239
124	-46.6823771	0.071419789
125	-13.3614459	0.364502341
126	-0.56006975	0.399003476
127	-18.3413136	0.239737159
128	-12.3295648	0.415078889
129	-7.1714977	0.067205353
130	-16.9902939	0.291086352
131	-4.05499244	0.723495308
132	-1.39828064	0.484480925
133	-7.92432581	0.522268319
134	-1.9928155	0.826386433
135	0.498916809	0.813962615
136	-1.79842982	0.856880753
137	0.400597406	0.821500056
138	-1.24801613	0.221505969
139	-1.1789832	0.509136755

IDP	Beta	p-Value	Interactions_sLM7_LATE_CHRONOTYPE_AGE_Summary
1	-3.65399768	0.400306763	
2	-4.92834892	0.309293405	
3	-1.65218379	0.168019527	
4	-1.69992935	0.157564298	
5	-4.30393448	0.239087583	
6	-2.95987977	0.379367417	
7	3.738388727	0.297384888	
8	-0.03659902	0.991466023	
9	-1.3818828	0.301958391	
10	-1.93098615	0.094458627	
11	-2.12441458	0.07641866	
12	-1.98035074	0.099135934	
13	0.879795641	0.797133684	
14	2.172767887	0.523752521	
15	-3.54973661	0.154291157	
16	-3.77529336	0.12363509	
17	-0.60327648	0.354341935	
18	-0.725326	0.273998822	
19	1.429617344	0.171467025	
20	1.158940723	0.302078595	
21	-2.02119491	0.01524352	
22	-1.60275145	0.01959078	
23	0.668757143	0.703472353	
24	-1.48847591	0.393044008	
25	-0.68140763	0.692793904	
26	1.4779486	0.459591135	
27	-0.36770037	0.600964619	
28	-1.02719675	0.108598567	
29	-2.45962387	0.141116394	
30	-4.34632869	0.007020612	
31	2.378135942	0.074073064	
32	2.050859171	0.198582012	
33	3.54680899	0.249437163	
34	-0.31001306	0.918132829	
35	1.234257478	0.55612165	
36	-1.05559514	0.605897502	
37	-0.3310749	0.821695892	
38	0.883365153	0.528161918	
39	-0.26856458	0.887853361	
40	0.659200373	0.775729228	
41	-0.04755511	0.978736606	
42	-3.4241691	0.164998096	
43	3.394838651	0.442090454	
44	7.734188193	0.089364603	
45	-1.00278666	0.692582016	
46	1.081237605	0.685097211	
47	0.644897484	0.632847288	
48	0.035251197	0.978086186	
49	-0.64157251	0.371114464	
50	-0.93183638	0.204616995	
51	-0.1109877	0.929929742	
52	-0.36533752	0.772101033	
53	0.493948905	0.508711829	
54	0.255532205	0.706216484	
55	-0.0159734	0.992360384	
56	-0.48674536	0.77374345	
57	-0.11310154	0.960118416	

58	1.339462642	0.593611586
59	0.536263691	0.682706557
60	-0.39687149	0.772182258
61	2.172483499	0.384161968
62	1.405196927	0.591763307
63	-0.39974402	0.648707604
64	-0.53033127	0.590773656
65	0.02368783	0.987746704
66	0.328797243	0.81906117
67	0.362637991	0.681841803
68	1.048863768	0.26619593
69	0.611409933	0.202033041
70	0.744538481	0.06149806
71	1.849728903	0.257476886
72	2.850924331	0.089859892
73	-0.12952309	0.810024226
74	-0.38983802	0.426867279
75	-0.07443942	0.946567289
76	-0.53439722	0.571864992
77	0.950291407	0.338971103
78	0.119261076	0.922433692
79	-1.51568621	0.236275339
80	0.674043955	0.595729018
81	-1.5366251	0.005714672
82	-1.48444054	0.005579776
83	-0.36459619	0.740914592
84	-0.11662132	0.919453029
85	-0.73398074	0.44135485
86	0.802356035	0.363025739
87	0.300593054	0.492800094
88	-0.14790683	0.738679255
89	-0.55701275	0.313385047
90	0.632935443	0.120234645
91	-0.87580737	0.375726977
92	0.074126328	0.908919549
93	0.015156688	0.952477938
94	0.147661319	0.667148727
95	-0.13403666	0.962943112
96	-0.14565425	0.958141081
97	1.244089369	0.051831205
98	1.30820642	0.048989913
99	-0.54208467	0.726572298
100	-0.13118978	0.93518774
101	1.011546621	0.308031532
102	0.827341658	0.438929933
103	0.002946131	0.974680673
104	-0.13374677	0.203501431
105	0.602421778	0.461793936
106	2.091044358	0.013490429
107	0.01452581	0.974897851
108	-0.66983849	0.182973289
109	0.345098073	0.14372923
110	0.303917176	0.197304456
111	3.815247284	0.030182008
112	0.919998157	0.150732009
113	1.735333598	0.011917667
114	0.768848468	0.358300884
115	1.849618193	0.023707614

116	1.64565867	0.447395428
117	-0.17208676	0.755141184
118	2.233781245	0.293608197
119	4.994039955	0.138906233
120	0.003664327	0.332929887
121	5.180736498	0.152653161
122	-1.0436312	0.688969019
123	0.094525482	0.605273275
124	1.510748645	0.55102941
125	-1.88077229	0.19209807
126	0.060876284	0.348858458
127	-0.41558164	0.785455136
128	-0.25544137	0.863007848
129	-0.14867944	0.698180672
130	-0.28639808	0.855691801
131	0.913578413	0.41533777
132	0.002102904	0.991427339
133	0.651941941	0.590616323
134	1.087587605	0.221233972
135	-0.02206716	0.915296158
136	0.185156593	0.849511784
137	0.023991036	0.890179831
138	0.056305109	0.572999437
139	-0.13895547	0.426535186

IDP	Beta	p-Value	Interactions_sLM7_LATE_CHRONOTYPE_SEX_Summary
1	-122.922822	0.068157672	
2	-79.4727288	0.290552609	
3	15.37629829	0.408177618	
4	10.07700128	0.589131577	
5	-12.5138978	0.825353112	
6	-21.592342	0.679311311	
7	-54.7246123	0.325425571	
8	-36.7311618	0.488933792	
9	10.79163905	0.603294138	
10	-12.3646128	0.489985339	
11	17.09540698	0.357999699	
12	-4.7248808	0.799772927	
13	-72.5087939	0.172027352	
14	-16.3845294	0.756606909	
15	2.377123008	0.950962479	
16	-15.6847876	0.680062222	
17	3.600658912	0.721564683	
18	12.37910644	0.228768829	
19	-18.5516435	0.252630515	
20	8.315815579	0.633105358	
21	18.50664966	0.152046539	
22	0.496050738	0.962854559	
23	-67.2319517	0.013634431	
24	36.14274261	0.181238085	
25	2.133582175	0.936440679	
26	-19.486195	0.529639146	
27	5.82268243	0.593403394	
28	-17.1393168	0.084368603	
29	-41.1968911	0.112059987	
30	-48.5898455	0.052023718	
31	-22.8707544	0.268128917	
32	-1.84907117	0.940434871	
33	-40.7895754	0.393183665	
34	34.01259063	0.467242498	
35	31.648775	0.330562514	
36	-9.37078638	0.767795923	
37	-7.17327943	0.752931095	
38	-9.59488235	0.658705893	
39	-38.6569487	0.19069386	
40	-8.3642899	0.81573735	
41	-18.0893005	0.513387553	
42	4.725634686	0.901688018	
43	-1.44977179	0.983116783	
44	-60.7013608	0.39006153	
45	-84.4498561	0.031850083	
46	-91.7766764	0.026495677	
47	-1.28683577	0.950999268	
48	-6.37945265	0.748622852	
49	-22.3759691	0.044342256	
50	-24.6438201	0.030571441	
51	28.66217987	0.14322552	
52	3.946206351	0.840170768	
53	-14.3308685	0.216464591	
54	-24.4943254	0.019849305	
55	37.36311821	0.148798541	
56	38.70667312	0.140557984	
57	17.05116135	0.626976642	

58	29.18758184	0.45350906
59	-15.3753291	0.449929879
60	7.208484878	0.734608794
61	-19.8361917	0.608481412
62	5.268102784	0.89687513
63	-9.28528711	0.49514114
64	-11.123825	0.467174251
65	-62.2586072	0.009268015
66	-28.3755093	0.203148621
67	16.99965497	0.215398882
68	19.29860537	0.187230992
69	-3.43841941	0.643706298
70	-6.5252607	0.290747494
71	-7.45748132	0.76852128
72	22.47833639	0.388615976
73	8.979123981	0.282678264
74	2.08151229	0.784471603
75	-28.9948523	0.092417809
76	-29.5279352	0.044054372
77	-14.2662847	0.354757931
78	-9.70389462	0.609539829
79	-20.9361024	0.291602318
80	-17.357737	0.378447418
81	0.423042318	0.96087747
82	-6.74363586	0.416963037
83	-13.0209566	0.446521513
84	-11.7025812	0.513014996
85	7.851187411	0.595484873
86	-8.08998716	0.554357547
87	-1.1893033	0.861130221
88	0.666854288	0.922756996
89	-2.67912635	0.754589362
90	-1.80011257	0.775733863
91	-6.21937646	0.685103213
92	-2.98204407	0.766705086
93	-0.16097546	0.967452809
94	-8.69239501	0.102675989
95	-54.6830663	0.221745238
96	-70.0194355	0.103838231
97	8.806640503	0.374873816
98	-9.44547403	0.359486288
99	-28.773617	0.231481761
100	-27.7279581	0.267866159
101	-12.1135724	0.431316442
102	-13.0669442	0.430663828
103	-0.35723468	0.804063277
104	0.608779797	0.709044188
105	8.943324917	0.481262475
106	8.364672631	0.524043272
107	13.88844472	0.052451304
108	8.059777159	0.301633644
109	-0.35596394	0.922551419
110	4.970013822	0.174094728
111	14.10753883	0.605341689
112	12.61592689	0.203982383
113	10.94197191	0.306703496
114	18.08954159	0.163533985
115	10.46745621	0.409256415

116	14.6213919	0.66344154
117	5.575210738	0.514825521
118	11.65668282	0.723858411
119	-14.3691261	0.783702988
120	-0.09677282	0.099274234
121	17.64858171	0.753452408
122	-46.0950744	0.254437706
123	0.264849565	0.925623321
124	-63.4266467	0.106607792
125	-6.03955924	0.787137824
126	0.160124466	0.873786755
127	-22.695477	0.337888821
128	5.730219507	0.802955352
129	2.443844482	0.681149023
130	-3.53602585	0.884910166
131	7.555979458	0.66407535
132	8.72982367	0.004037611
133	20.70380351	0.270771332
134	15.83439916	0.250926181
135	4.856355272	0.131320982
136	25.86030434	0.087573872
137	-1.47406199	0.584443516
138	2.508631432	0.10547731
139	-1.23229719	0.649413014

IDP	Beta	p-Value	Interactions_sLM7_LONG_SLEEP_DURATION_AGE_Summary
1	-7.25320922	0.545875133	
2	-8.25843961	0.537689334	
3	-0.97663625	0.768148934	
4	0.98876603	0.766176019	
5	7.060691945	0.484763757	
6	3.390780441	0.715637682	
7	-4.27045417	0.666747561	
8	6.405020564	0.498320474	
9	-1.67391942	0.6510324	
10	-2.81304111	0.378146202	
11	-0.86788071	0.793431417	
12	-2.76938391	0.404147692	
13	4.923434309	0.602796432	
14	-2.68588016	0.775561468	
15	-0.57732885	0.933204825	
16	-9.20495943	0.174450522	
17	-0.75598089	0.674585679	
18	-2.21693877	0.226473773	
19	2.268800171	0.432405119	
20	0.831626367	0.788787069	
21	0.462585294	0.840770876	
22	0.536626882	0.777395726	
23	1.823209124	0.70736873	
24	-2.89422841	0.547989804	
25	-1.18880079	0.803103836	
26	-1.87491178	0.73432782	
27	0.189990727	0.922123394	
28	0.849335832	0.63127113	
29	-2.35385709	0.61041597	
30	-0.16736483	0.97004238	
31	-4.77240813	0.194741641	
32	-1.39602199	0.751567618	
33	7.541743205	0.375668269	
34	-4.18674722	0.615560163	
35	-7.25359353	0.210807238	
36	-2.70599115	0.632332929	
37	2.374259843	0.558786648	
38	-3.83312516	0.322081541	
39	-0.99538823	0.850034291	
40	-3.55448897	0.578415903	
41	-2.13910283	0.664510475	
42	-5.12993776	0.451757755	
43	3.318648266	0.785757049	
44	21.31211383	0.090386813	
45	-9.75801639	0.164020017	
46	-4.51085766	0.540536567	
47	3.826452843	0.305183592	
48	-0.98932769	0.780343281	
49	-1.55730645	0.432251137	
50	-0.6046721	0.765877148	
51	-1.34746572	0.699355754	
52	-3.93254534	0.259415852	
53	-4.55234473	0.027584166	
54	-3.43948329	0.066454529	
55	2.041350732	0.658016523	
56	-3.59207153	0.442806001	
57	7.803343209	0.212018734	

58	6.893738551	0.320484569
59	5.028689379	0.165556807
60	8.144494762	0.031614836
61	3.889320202	0.573027055
62	4.113086527	0.570139991
63	-2.90342182	0.231328148
64	-3.80217995	0.163147536
65	-7.43264044	0.081301111
66	-2.51670068	0.526476066
67	-0.78155286	0.749262392
68	-1.78799097	0.492929426
69	-0.19789788	0.881252882
70	0.442099182	0.687928471
71	-3.2937357	0.465743221
72	-3.70204058	0.425588153
73	-0.92616156	0.5340558
74	-0.37416389	0.782643196
75	-6.85835095	0.025511745
76	-2.68584761	0.30404795
77	-3.30910412	0.228396614
78	-1.34060614	0.692151066
79	-1.24750015	0.724364979
80	-6.44832378	0.066351403
81	-0.09265799	0.951925297
82	1.7782297	0.229725488
83	0.474151312	0.876387865
84	1.420124052	0.65599202
85	1.303339695	0.620911184
86	-0.4375272	0.857597217
87	-0.60384234	0.618194074
88	-1.54611103	0.207132917
89	-0.17470702	0.908930911
90	-0.765639	0.496543986
91	0.960177265	0.72535913
92	-0.51246693	0.774795095
93	-0.46775215	0.50584821
94	-1.2724115	0.180054526
95	-9.03429118	0.257293804
96	-11.235771	0.143023744
97	2.734870595	0.122017364
98	1.232082499	0.502384843
99	18.27082451	2.02E-05
100	18.39705641	3.71E-05
101	1.097744503	0.689026103
102	0.041308784	0.988845938
103	0.656850383	0.01047718
104	0.826983228	0.004452138
105	-1.94991902	0.388872786
106	1.329352338	0.569904026
107	-2.06153253	0.10621633
108	-1.95691776	0.159325956
109	-1.99607546	0.002220894
110	-1.61084728	0.01343901
111	9.559360678	0.049440371
112	-0.61911243	0.726478624
113	-0.3485706	0.855013664
114	-3.79870421	0.100628067
115	-1.36515887	0.545893198

116	-12.0161029	0.044774231
117	-1.25967477	0.408906476
118	-9.8900675	0.092553023
119	3.446065791	0.711821561
120	0.023102573	0.027230691
121	-1.72454813	0.863259495
122	3.878745458	0.590488341
123	0.009068968	0.985688601
124	3.962281353	0.571617036
125	-1.5629234	0.694967296
126	-0.10826285	0.546720036
127	-2.35972554	0.576061783
128	-5.14715593	0.208493782
129	-1.03944431	0.326740684
130	-0.94353436	0.828413308
131	-3.08278408	0.320082277
132	-0.05460752	0.919605582
133	-0.21330868	0.949232068
134	0.702385384	0.775043435
135	-0.07388281	0.897499888
136	1.726297074	0.522207927
137	0.261392923	0.586293907
138	0.36171614	0.190245222
139	0.165231536	0.732327532

IDP	Beta	p-Value	Interactions_sLM7_LONG_SLEEP_DURATION_SEX_Summary
1	102.2386532	0.584620113	
2	-172.898822	0.407365056	
3	79.719721	0.12231154	
4	60.71394131	0.240984832	
5	63.95121748	0.684492809	
6	-16.5768966	0.908951713	
7	27.40960883	0.859134462	
8	83.87149249	0.569109439	
9	101.8587551	0.077165597	
10	91.38922027	0.065989209	
11	-34.0185647	0.509842733	
12	94.13230384	0.068640921	
13	0.173024684	0.999063033	
14	109.3058576	0.456242104	
15	-58.2524328	0.587109491	
16	163.7283548	0.120883089	
17	21.19862183	0.4496477	
18	47.15630451	0.098538197	
19	-36.036107	0.423297458	
20	-40.8308341	0.398359023	
21	2.299529879	0.948866404	
22	-2.77800257	0.925126189	
23	0.909602941	0.990404865	
24	-36.0967701	0.630419592	
25	-6.44299259	0.9308531	
26	49.34154539	0.56630891	
27	-37.4457551	0.216008732	
28	-7.47703525	0.786157145	
29	117.3473046	0.102907362	
30	44.96770995	0.517033368	
31	31.42195673	0.583542526	
32	1.141307842	0.986740562	
33	-14.9111181	0.91044826	
34	-125.923912	0.332142759	
35	16.32274646	0.856509766	
36	78.82557265	0.37082132	
37	-24.8263711	0.694649912	
38	31.29611748	0.603668225	
39	-41.690722	0.61109544	
40	116.8827257	0.240652382	
41	14.97164102	0.845460565	
42	54.3157651	0.608927659	
43	133.142998	0.483759756	
44	-262.77884	0.180012319	
45	71.29868548	0.51378469	
46	55.45903565	0.628985549	
47	-72.0190576	0.215257153	
48	-11.8454735	0.830230391	
49	12.59290246	0.683429506	
50	15.59135464	0.621995963	
51	3.552421228	0.947873375	
52	-12.8403625	0.81307922	
53	-5.57561409	0.862434717	
54	-9.36063807	0.748403026	
55	-20.085281	0.779731293	
56	-12.7324316	0.861330127	
57	86.38509722	0.374986967	

58	139.0820223	0.198086767
59	-79.8731876	0.157290032
60	-61.0880198	0.300591325
61	-95.548204	0.373963415
62	-93.2734946	0.408308207
63	-6.52874295	0.862785298
64	-32.0038634	0.450993654
65	32.40289344	0.625554594
66	-37.2180713	0.547524191
67	-67.4853087	0.076380474
68	-54.914197	0.176310173
69	-20.4513088	0.321552672
70	-40.3939979	0.018448199
71	184.3822607	0.008745178
72	86.92781465	0.22962044
73	-48.1942206	0.037736002
74	11.81236487	0.57598877
75	56.85656912	0.234429403
76	15.363477	0.705789496
77	7.803827482	0.85526754
78	-36.9443749	0.483529581
79	-63.2698381	0.250803453
80	3.870606053	0.943581212
81	-36.9656919	0.122484304
82	-30.7705502	0.182024952
83	-0.24068378	0.995954632
84	71.99547585	0.14703297
85	27.89039275	0.496775937
86	5.27576215	0.889503357
87	1.385056739	0.941480146
88	21.49826883	0.2600239
89	30.28368348	0.202953354
90	22.16671051	0.206213552
91	39.74638175	0.350412409
92	23.49957613	0.399537288
93	17.61293601	0.107696237
94	16.69597433	0.258670517
95	34.24602434	0.782748825
96	38.69389885	0.746024175
97	-37.9888093	0.167809132
98	-39.846953	0.163643829
99	-95.3978611	0.15287104
100	-137.871986	0.047132043
101	-94.8203723	0.026451962
102	-123.226131	0.007412611
103	-6.33695104	0.112798935
104	-5.77049333	0.202507301
105	-65.3682111	0.063623523
106	-74.1493685	0.041847779
107	-10.3896333	0.601120064
108	-38.6569462	0.074239444
109	-8.74860822	0.389255108
110	-23.4457364	0.020872103
111	-144.760467	0.056064493
112	-5.15053644	0.851762367
113	-1.72768528	0.953624568
114	34.59430382	0.33700121
115	7.29917534	0.835739789

116	92.73287248	0.319987374
117	-4.51813316	0.849153223
118	46.3897575	0.612408168
119	-23.9423166	0.869096738
120	-0.09935578	0.54197779
121	-130.517655	0.402607218
122	85.90907277	0.444070512
123	-1.37102008	0.861763971
124	25.54275114	0.814862776
125	-4.08566931	0.94751983
126	-0.8144051	0.77096024
127	-70.6024037	0.282715354
128	-28.6640443	0.652885472
129	-13.9786703	0.397061956
130	-122.561852	0.07064406
131	-25.9269177	0.591294403
132	-3.73500288	0.657561183
133	-62.0453687	0.234356219
134	-10.371892	0.786404603
135	-1.33518599	0.881169555
136	-34.7614046	0.407979327
137	1.341350638	0.857682879
138	-5.21883444	0.224928206
139	-4.74132425	0.528551184

IDP	Beta	p-Value	Interactions_sLM7_SHORT_SLEEP_DURATION_AGE_Summar
1	7.116587926	0.022062567	
2	5.492273005	0.113306722	
3	1.089675762	0.203823358	
4	1.560361714	0.069826644	
5	2.732023271	0.29629261	
6	4.025670762	0.09473781	
7	3.242434148	0.206535472	
8	5.330158541	0.02948269	
9	0.89875552	0.348102471	
10	0.298927523	0.717484318	
11	0.664164542	0.438815112	
12	0.048995704	0.954528101	
13	2.675984392	0.274517961	
14	2.968810893	0.223410145	
15	2.556261943	0.151655629	
16	1.140897933	0.515497645	
17	0.304927171	0.512931176	
18	0.845162975	0.074850572	
19	-0.06097696	0.935029205	
20	1.27028011	0.113916744	
21	0.883443894	0.138249366	
22	1.36723952	0.005390771	
23	0.148381499	0.906042025	
24	1.987656789	0.110938421	
25	0.272242361	0.825407258	
26	1.643232843	0.25050667	
27	0.454924989	0.365811509	
28	0.424954107	0.353551494	
29	2.588265947	0.030442903	
30	1.786263166	0.121498906	
31	-0.20150814	0.832476426	
32	-0.83442155	0.464758224	
33	-0.15446885	0.944112363	
34	2.262100094	0.294551011	
35	1.597544602	0.286984705	
36	1.453371291	0.320817741	
37	0.843076599	0.422523621	
38	-0.14747454	0.882987626	
39	0.344892512	0.800188958	
40	-2.31363872	0.16228873	
41	-0.61610408	0.629387163	
42	-1.08968941	0.536880829	
43	5.686044518	0.071974133	
44	4.71533871	0.147764564	
45	-2.84748032	0.116660385	
46	-1.56176215	0.4130063	
47	-0.54245811	0.57438656	
48	0.555284179	0.545368448	
49	0.484421912	0.345270197	
50	0.460304079	0.381167626	
51	1.485332057	0.100044071	
52	1.802267614	0.045849021	
53	0.743652351	0.164387351	
54	0.470525257	0.332028979	
55	1.060316185	0.374388321	
56	1.424194741	0.239763447	
57	-0.62342134	0.700077695	

58	-0.73647864	0.681772918
59	0.443095558	0.636898488
60	0.671654592	0.49348016
61	2.490014721	0.163315293
62	4.703016409	0.012129023
63	1.213898103	0.053193006
64	1.120631693	0.112296151
65	0.655864356	0.552320909
66	1.31188192	0.20210634
67	1.251545813	0.048008417
68	1.62877546	0.015821804
69	-0.6239569	0.068823378
70	0.106555855	0.70838818
71	-0.50475546	0.66584199
72	0.433672699	0.718399419
73	0.372995144	0.333288497
74	0.684812192	0.051096411
75	0.489994928	0.537541797
76	0.766737185	0.256982551
77	0.242065922	0.73354551
78	-0.74865694	0.392973517
79	-0.05088847	0.955681158
80	-1.4635266	0.107409884
81	0.406170095	0.307246023
82	0.688860516	0.072251225
83	-0.35707097	0.650863798
84	1.610493078	0.05098334
85	0.393764526	0.563765928
86	0.501271839	0.427065909
87	-0.02214517	0.943701685
88	0.683816679	0.031124156
89	0.213922718	0.58842379
90	0.695641159	0.017003562
91	0.943187524	0.182463198
92	0.750495176	0.105503762
93	0.015968232	0.930076101
94	0.466492918	0.057591108
95	2.526301151	0.221017902
96	1.082528688	0.585629237
97	0.401629674	0.380291774
98	0.512193669	0.281365078
99	-0.86974222	0.432970931
100	-0.87681091	0.447501475
101	-0.02747215	0.969136313
102	0.069301598	0.927801853
103	0.04770032	0.472649293
104	-0.03848156	0.60910829
105	0.638771391	0.27548035
106	0.867251887	0.152120912
107	0.850263157	0.010052462
108	1.089644692	0.002467195
109	0.210435036	0.212766893
110	0.035021247	0.835512992
111	-0.29090542	0.81731147
112	0.275784571	0.547163234
113	0.641167441	0.194111718
114	0.200370141	0.737940446
115	0.879384734	0.132851601

116	2.191480353	0.157361324
117	0.650535025	0.099426208
118	2.27131211	0.135582299
119	-0.03167291	0.989534162
120	-0.00212977	0.431577986
121	-0.97373946	0.707141363
122	1.819641262	0.329397975
123	0.330718876	0.011501789
124	0.061219147	0.973063762
125	0.780507512	0.449324957
126	0.065722576	0.157514074
127	0.194405921	0.858746637
128	2.222752431	0.035877687
129	0.208796699	0.446607668
130	1.624960502	0.149285247
131	2.180395876	0.006591852
132	0.300198204	0.032068245
133	2.624739093	0.002472781
134	0.663899226	0.296671025
135	0.108293377	0.465708586
136	1.480636813	0.03396533
137	0.307950677	0.013252496
138	0.010834847	0.879514509
139	0.165316224	0.186144531

IDP	Beta	p-Value	Interactions_sLM7_SHORT_SLEEP_DURATION_SEX_Summar
1	1.348696829	0.977572238	
2	-42.1947745	0.43053918	
3	-5.34626275	0.686230617	
4	-15.3147041	0.248900454	
5	-21.3686351	0.596589035	
6	2.156194803	0.953756188	
7	81.09807364	0.040653137	
8	59.83112024	0.113330933	
9	-5.82564158	0.693526166	
10	-1.94798774	0.878573662	
11	6.72283123	0.611611219	
12	1.453113524	0.91274419	
13	16.82407215	0.656214996	
14	19.54342434	0.603535505	
15	-4.75554309	0.862789498	
16	-33.5003449	0.215995469	
17	0.537464216	0.94043411	
18	-2.22448426	0.761274765	
19	-1.68586545	0.883893253	
20	19.57033373	0.114548909	
21	-13.1974754	0.151332605	
22	-8.25932202	0.276039545	
23	8.238646163	0.67110014	
24	23.03244316	0.231374924	
25	-18.2985691	0.336691147	
26	-27.6238834	0.210686896	
27	-1.14784772	0.882460167	
28	5.808979961	0.411241021	
29	-4.63872581	0.801551487	
30	-5.75163244	0.746634248	
31	-13.5433401	0.356963325	
32	-6.99448359	0.69132602	
33	-34.2398063	0.314010776	
34	-12.8948575	0.698637573	
35	27.02639039	0.243147524	
36	-12.0392468	0.594124301	
37	-8.08045438	0.618415017	
38	16.44776091	0.287500916	
39	7.625348634	0.71691161	
40	9.176627999	0.719491531	
41	0.155381548	0.9937078	
42	-36.6349047	0.178554239	
43	-105.321378	0.030815074	
44	-9.88493172	0.844127378	
45	-37.359237	0.182269603	
46	-37.6747867	0.200705633	
47	31.30892458	0.035711163	
48	26.46646049	0.061829804	
49	3.857294913	0.626292837	
50	-7.36755062	0.363752553	
51	15.55390816	0.264454846	
52	6.556178749	0.637876039	
53	1.551414002	0.850907392	
54	2.452698115	0.743185418	
55	36.70605194	0.046322253	
56	42.20219733	0.024002733	
57	-11.2628709	0.652036361	

58	15.95336673	0.564934595
59	-17.0509855	0.239202873
60	-11.7253368	0.438578198
61	-26.703216	0.332713082
62	-31.4459569	0.277136832
63	-0.37191098	0.969383475
64	-5.10699975	0.639132081
65	-28.7790116	0.09109848
66	12.41642725	0.434059213
67	8.748056797	0.370493995
68	-7.24737966	0.486601228
69	-5.80812649	0.27242594
70	-6.57923758	0.134564372
71	30.52033873	0.090655834
72	27.99970137	0.131421181
73	9.383845504	0.114762239
74	-7.17974944	0.185115398
75	0.554732049	0.963926756
76	-1.64116226	0.875075575
77	-3.69858808	0.736104345
78	7.592224346	0.574580802
79	1.437493883	0.91897986
80	2.928048154	0.834675936
81	-2.96406137	0.629245019
82	-8.49820599	0.150753287
83	-5.74590501	0.637017312
84	14.4410098	0.256825224
85	0.814557765	0.938325553
86	5.657941059	0.56133512
87	0.777981288	0.872290825
88	-5.94980895	0.224275824
89	-0.9632721	0.874550341
90	5.12177559	0.254862886
91	-3.01507367	0.782432119
92	7.965754605	0.265589953
93	2.211413482	0.431046452
94	-0.92062825	0.808150098
95	49.36691867	0.121245376
96	42.91461141	0.161401675
97	-3.53204225	0.617114065
98	3.350208669	0.647983222
99	29.41363183	0.08575941
100	12.75893311	0.473873239
101	38.99266478	0.000373665
102	32.84751192	0.005391878
103	1.798611458	0.079325785
104	0.856402591	0.460900183
105	-8.29205117	0.359000727
106	-10.4837495	0.261981875
107	5.993455317	0.239715669
108	3.152512531	0.570340476
109	7.157683386	0.006033498
110	5.328702559	0.040655964
111	-17.3684564	0.371506502
112	3.295824827	0.641092678
113	1.77314243	0.816006984
114	6.169158072	0.504466602
115	6.610437839	0.46413398

116	40.20706276	0.092772501
117	-2.0910946	0.731465933
118	51.16930363	0.029365706
119	-6.75973135	0.856055789
120	-0.08252834	0.048303705
121	13.62500666	0.73338628
122	19.30693168	0.502515935
123	-0.18316383	0.927738482
124	24.63550109	0.37862746
125	22.21432969	0.162964769
126	0.713304268	0.320211411
127	32.47052271	0.054103884
128	31.27007983	0.055782982
129	5.921821627	0.16192484
130	41.8048345	0.016225713
131	30.32573187	0.014349912
132	2.218917029	0.304584857
133	38.17116484	0.004343745
134	19.87007943	0.042992405
135	3.957060821	0.084148887
136	22.59932501	0.035982253
137	-2.36360991	0.21799969
138	0.753556715	0.494538064
139	-1.37641002	0.475698962

IDP	Beta	p-Value	Interactions_sLM7_SLEEP_APNOEA_AGE_Summary
1	-43.2475804	0.033707235	
2	-46.3149862	0.041526572	
3	5.874360443	0.295718099	
4	0.820477625	0.884298711	
5	-3.1017572	0.856369878	
6	-18.7186879	0.23564058	
7	-19.9829248	0.234728188	
8	-0.66582311	0.966888937	
9	4.070824197	0.516532361	
10	0.654416878	0.903763701	
11	11.7598645	0.03640655	
12	12.14538699	0.030968053	
13	-30.9669659	0.053591326	
14	-16.0878095	0.313901451	
15	-1.23586468	0.915737539	
16	-4.26500718	0.710583166	
17	-1.81001519	0.553303217	
18	1.624152426	0.601291567	
19	-3.95966638	0.419080831	
20	-7.5904733	0.14933826	
21	-0.74237641	0.849200129	
22	-2.76604021	0.390142281	
23	-7.11027983	0.38795646	
24	-7.47186601	0.36038142	
25	1.87825013	0.816298213	
26	-9.53165066	0.308947267	
27	-1.45089979	0.659751516	
28	0.651289165	0.828183533	
29	6.366104823	0.416463118	
30	5.689514403	0.451526626	
31	-4.79080037	0.442709688	
32	-4.7672103	0.523791934	
33	-19.1464031	0.184739396	
34	-0.98684817	0.944353156	
35	8.923536931	0.363964787	
36	-0.09558678	0.992047921	
37	-6.48231294	0.346543603	
38	3.989455046	0.543354461	
39	10.09599278	0.258092254	
40	-8.30799193	0.443705121	
41	-1.95819829	0.814886186	
42	-9.57835419	0.407356912	
43	14.26900696	0.490680205	
44	25.20003071	0.237687196	
45	3.679825764	0.756945229	
46	11.78471679	0.345745075	
47	-2.84780648	0.652686262	
48	-1.66343634	0.782155157	
49	-3.2241546	0.337641399	
50	-4.18250313	0.224512989	
51	0.737675517	0.900777918	
52	-4.06883312	0.491378148	
53	-3.29243701	0.347379229	
54	-5.3271093	0.093672534	
55	-6.13901961	0.432435618	
56	-4.53639271	0.567615399	
57	6.012443336	0.570660591	

58	9.278243886	0.430399148
59	1.0778801	0.860862592
60	8.717631818	0.174887706
61	-2.59753917	0.824334019
62	14.41337773	0.240612338
63	5.607557742	0.172800138
64	7.906349448	0.087247552
65	-5.47567278	0.448848157
66	-16.9470226	0.011898999
67	-4.12935616	0.319330552
68	-3.31079533	0.454029039
69	-2.24135704	0.318418812
70	-3.28612332	0.078303496
71	-7.19703676	0.347264157
72	-5.91174023	0.453061584
73	2.797112056	0.268090042
74	2.83503554	0.217696665
75	5.966913037	0.251796558
76	2.466985497	0.577719897
77	0.533020923	0.908907781
78	0.80650778	0.888290233
79	-1.62645503	0.786297009
80	6.612532424	0.266855295
81	0.690786937	0.790963573
82	0.324653949	0.897108777
83	-5.10404634	0.323431137
84	-1.96286558	0.716540469
85	1.798562759	0.687340868
86	1.86469997	0.652009347
87	3.182260923	0.121394867
88	3.146452008	0.130044923
89	-5.07495416	0.050062659
90	-1.29373081	0.498066516
91	-3.94545123	0.394615195
92	-0.84463053	0.780948653
93	0.852543634	0.474542323
94	1.142474229	0.477802861
95	8.757073093	0.517281293
96	7.818609294	0.547815332
97	-3.62294217	0.227031723
98	-0.81848917	0.792729243
99	1.351125444	0.852496378
100	-0.81879472	0.913779308
101	-0.83434094	0.857651093
102	-6.37906008	0.202986231
103	0.275838923	0.526131693
104	-0.0015591	0.997476822
105	-5.03047767	0.189891141
106	-5.88229823	0.13816669
107	1.939834683	0.370026871
108	-3.07231592	0.192582589
109	0.323400863	0.770064662
110	0.148809277	0.892872386
111	-6.60652379	0.423262278
112	-0.3957416	0.895092878
113	-1.17364028	0.716743125
114	-2.93752206	0.454025339
115	-0.63867032	0.867670954

116	-2.31433904	0.819694491
117	-1.89889231	0.462872181
118	1.546354041	0.876743345
119	-6.55292753	0.67868991
120	-0.0095597	0.58998076
121	-23.1607731	0.172567429
122	1.092811543	0.928756458
123	-0.70135072	0.413324851
124	-1.64837339	0.889626112
125	1.735680692	0.797331965
126	0.057507085	0.850259014
127	1.140972279	0.873326369
128	4.070168606	0.55753302
129	0.534110241	0.766333268
130	-4.25974149	0.56391728
131	-2.70589406	0.606786452
132	0.633250982	0.490055909
133	4.131426779	0.467081311
134	-0.30980786	0.94074316
135	-0.72790744	0.454193821
136	3.748417472	0.412535027
137	0.025178864	0.975338068
138	-0.50482385	0.281010735
139	0.360205181	0.660148033

IDP	Beta	p-Value	Interactions_sLM7_SLEEP_APNOEA_SEX_Summary
1	-600.270449	0.111860308	
2	-325.446285	0.439780836	
3	9.617114756	0.926428064	
4	10.3516753	0.921111424	
5	702.6940547	0.026987846	
6	382.1848448	0.191519411	
7	-256.387578	0.410868598	
8	-426.642405	0.151367048	
9	3.842169438	0.973654208	
10	109.0378792	0.277195774	
11	-172.539519	0.097738872	
12	37.93493336	0.716233642	
13	89.75492869	0.762831808	
14	31.10986149	0.916340257	
15	-241.016602	0.265717887	
16	-190.534616	0.371228754	
17	33.89275716	0.549329495	
18	107.9006069	0.061138393	
19	64.78146564	0.475810862	
20	-26.1875537	0.788444792	
21	-31.3455466	0.664981808	
22	-20.7875958	0.727566776	
23	-193.182965	0.205791923	
24	-383.645612	0.01130758	
25	-124.755708	0.405238151	
26	-229.325582	0.186708248	
27	-12.7344751	0.834893158	
28	-105.968161	0.056820497	
29	-231.543999	0.110904579	
30	-265.973766	0.057644506	
31	-190.28	0.100069464	
32	-212.903047	0.124609032	
33	-296.388408	0.268093797	
34	-447.938361	0.087465433	
35	87.04731679	0.63287681	
36	-155.562155	0.381624145	
37	-51.4830445	0.686758974	
38	-219.475513	0.071321821	
39	-120.104325	0.468028427	
40	-242.181088	0.228453572	
41	-176.014862	0.256314509	
42	-365.897177	0.087779034	
43	-831.445227	0.030295573	
44	-177.139447	0.654359689	
45	-463.857381	0.035352516	
46	-298.793138	0.197232277	
47	-70.0381801	0.550502618	
48	-73.9091191	0.507524858	
49	160.7198173	0.009936192	
50	42.62304567	0.504345165	
51	0.787090883	0.994274691	
52	103.4662432	0.345247996	
53	-87.6169515	0.177388186	
54	-34.1782823	0.561815227	
55	-104.018181	0.473079879	
56	-150.930634	0.305008623	
57	-153.026507	0.436263356	

58	21.80491647	0.920379424
59	23.17775808	0.838901529
60	-95.8492175	0.421053462
61	-97.5449529	0.652977209
62	13.01507243	0.954420755
63	-83.1901777	0.275310868
64	41.25924174	0.630248015
65	-307.788874	0.021667413
66	-144.441132	0.247543609
67	-93.3510503	0.224625452
68	-32.3083341	0.693504275
69	43.62164944	0.294921467
70	21.68670109	0.530823677
71	103.572523	0.465630813
72	109.1214728	0.455030354
73	-70.1611822	0.134029935
74	-73.3446265	0.085406261
75	-115.694542	0.230702215
76	5.659008465	0.945081618
77	-37.0244452	0.66814947
78	55.9770196	0.598969994
79	-7.94223841	0.943069098
80	-0.00120911	0.999991262
81	25.85535042	0.592559485
82	18.80909435	0.68612827
83	-83.23958	0.38505072
84	34.65466143	0.729512951
85	-50.0537274	0.545738648
86	-105.269345	0.169675894
87	-55.4309203	0.145575523
88	-26.9980404	0.483490313
89	-16.2809274	0.734551168
90	-8.09172369	0.819194234
91	40.4700049	0.637641311
92	-17.3515374	0.757971356
93	-43.5223726	0.048941817
94	-45.2521659	0.129378482
95	-460.18644	0.066438221
96	-87.3326891	0.717257655
97	24.015016	0.66578905
98	13.30367853	0.817797464
99	97.63487554	0.468616184
100	124.5402102	0.3743767
101	106.9277183	0.215008465
102	95.06499413	0.306129735
103	17.5076619	0.029989641
104	9.420885823	0.302680087
105	96.18002974	0.176395863
106	117.7244181	0.109477193
107	14.35974644	0.720389576
108	85.81010951	0.049649953
109	-4.63118165	0.821376382
110	-9.15747139	0.654859645
111	137.9961261	0.366938013
112	81.29180801	0.144005513
113	102.9834009	0.085944003
114	129.0586881	0.07601256
115	148.2607979	0.036955649

116	314.5433404	0.094725087
117	63.05622911	0.188531092
118	333.5697929	0.071133525
119	297.2773783	0.310738014
120	-0.73503772	0.02543006
121	757.3190278	0.016142289
122	398.7176379	0.078482051
123	32.68066723	0.039775625
124	215.1616573	0.32852354
125	303.1014815	0.015569929
126	12.37802261	0.028394496
127	201.1263633	0.129536095
128	290.3307883	0.024034174
129	76.70610443	0.021335449
130	299.7195779	0.028529905
131	174.9617198	0.072657395
132	34.60023163	0.041933417
133	155.4472306	0.139964496
134	146.2522535	0.05838287
135	34.09003879	0.058671616
136	190.3160297	0.024826206
137	6.959506699	0.644868906
138	14.74954613	0.089326477
139	12.51936991	0.409745882

IDP	Beta	p-Value	Interactions_sLM7_SLEEP_MEDICATION_AGE_Summary
1	21.80545687	0.347888063	
2	14.86887523	0.56618604	
3	4.693534501	0.463895472	
4	2.953343299	0.646076647	
5	4.77933541	0.806844856	
6	1.095654148	0.951472573	
7	55.68660304	0.00369759	
8	65.30496287	0.000358325	
9	7.107076208	0.320769899	
10	-1.13923021	0.853600778	
11	-8.06700233	0.208267061	
12	-7.87920241	0.219792009	
13	9.840271462	0.590775721	
14	9.611467915	0.597865859	
15	7.191822825	0.589349663	
16	17.94589738	0.171057923	
17	1.036241739	0.766054124	
18	-4.60326896	0.194155864	
19	6.511354813	0.244073461	
20	3.100163139	0.605652161	
21	-3.45091484	0.438421053	
22	7.035019544	0.05535308	
23	12.87491787	0.170534486	
24	0.782577658	0.933069331	
25	-15.0205115	0.10338279	
26	-13.3159872	0.212726088	
27	1.957707418	0.602514868	
28	1.384131977	0.685945711	
29	-6.13194828	0.492605299	
30	6.920149	0.422091623	
31	2.457431926	0.729944971	
32	-3.88438838	0.648822562	
33	-6.10919177	0.710627688	
34	12.43455968	0.440684195	
35	7.518798979	0.502473978	
36	-1.85195834	0.865570184	
37	-0.71593131	0.927378869	
38	-1.85312799	0.804527385	
39	-5.75080146	0.572244033	
40	12.39311037	0.316498138	
41	-4.38532114	0.645757006	
42	-5.15986905	0.695572007	
43	-25.1378854	0.287108341	
44	-19.2609866	0.428816432	
45	15.90014211	0.241045336	
46	-6.00145247	0.673786097	
47	0.656562248	0.92752349	
48	-0.34079056	0.960390319	
49	4.613285009	0.229066135	
50	3.406540029	0.385784575	
51	1.096597058	0.870922319	
52	1.34678544	0.841729614	
53	5.17082203	0.195735076	
54	1.420027843	0.695235862	
55	-3.22682648	0.717538202	
56	-7.0604137	0.435458624	
57	12.97094526	0.283485615	

58	11.51641279	0.390875931
59	-10.2139398	0.14537177
60	-3.08761933	0.673566763
61	-8.87038989	0.506339474
62	-12.8693138	0.358330111
63	-1.56339397	0.738972114
64	-0.34160599	0.948351049
65	3.567290882	0.66534185
66	-0.34561119	0.964131501
67	-5.10693175	0.280269095
68	-3.7390026	0.458521088
69	0.053557736	0.983324722
70	1.63245423	0.443208211
71	-8.00121323	0.359625406
72	-11.5411274	0.199083944
73	-0.29985065	0.917104099
74	-1.82038587	0.487739697
75	3.901587744	0.511222421
76	0.036871732	0.994179712
77	2.404849268	0.650865907
78	-3.65478666	0.576808804
79	6.601641533	0.334667502
80	-0.32644465	0.961671926
81	6.379764861	0.031871254
82	-0.9294295	0.745518278
83	8.641655079	0.142743626
84	1.901323639	0.757828846
85	2.196369476	0.666557241
86	0.068245094	0.988455292
87	1.031586925	0.659786312
88	-3.00671902	0.204689705
89	1.903967649	0.519261011
90	-1.8629763	0.392362563
91	-0.30691696	0.953704506
92	0.295618427	0.932001781
93	-1.3958791	0.304670528
94	-0.37322876	0.838901064
95	12.1816903	0.429704616
96	-6.91137128	0.64137268
97	-1.72111644	0.614873811
98	-1.12832642	0.750811196
99	-6.73313047	0.416612078
100	-7.54929449	0.381480755
101	-5.51746392	0.298404676
102	-1.39191388	0.807589589
103	0.295224709	0.551970722
104	1.32481497	0.018488784
105	0.786991622	0.857312352
106	-3.33858589	0.460670549
107	-0.57349108	0.816274677
108	0.390637756	0.884518964
109	-1.45814981	0.247944311
110	-1.60582432	0.202652274
111	-12.3629797	0.188941671
112	-4.3832293	0.200406168
113	-5.57402368	0.130883906
114	-3.47810785	0.437047265
115	-4.26992924	0.328778493

116	-10.9206223	0.345717085
117	-1.48596045	0.614512067
118	-4.30027733	0.705334301
119	-19.2670978	0.285614279
120	-0.00684761	0.735068368
121	-25.2633139	0.192111227
122	-14.7129493	0.291284238
123	-0.65331366	0.504095344
124	-12.6411797	0.350807842
125	-8.5945025	0.264944276
126	-0.18534445	0.593736736
127	-2.41392865	0.767445885
128	-2.18881572	0.782153014
129	-1.04762447	0.609343087
130	-2.01340943	0.811021414
131	-3.94149692	0.511030348
132	-0.54845802	0.600220466
133	-7.31029714	0.259271956
134	-2.60044284	0.584370368
135	-0.64740211	0.559501987
136	-2.69993434	0.604841626
137	-1.1452685	0.217677257
138	0.240183089	0.652951285
139	-1.52723411	0.102175511

IDP	Beta	p-Value	Interactions_sLM7_SLEEP_MEDICATION_SEX_Summary
1	4.822414821	0.990498037	
2	-415.315057	0.357976461	
3	-8.84118707	0.936913997	
4	26.12212021	0.815754455	
5	27.05030547	0.93672643	
6	364.0857032	0.246004865	
7	-126.345975	0.705539841	
8	-353.183561	0.268128605	
9	80.45540828	0.519062614	
10	-38.7314347	0.718931167	
11	16.86291708	0.880056393	
12	27.84994832	0.80350342	
13	-341.741389	0.284052367	
14	85.15863936	0.788622503	
15	10.67070919	0.963354919	
16	161.6318238	0.479416642	
17	22.1185917	0.715608864	
18	57.25871851	0.354194316	
19	35.66408171	0.714355165	
20	-135.836539	0.194386418	
21	24.80969546	0.749291679	
22	-33.4263153	0.601471048	
23	115.9375792	0.478961686	
24	-384.733024	0.017862175	
25	-128.518763	0.424042639	
26	-308.444345	0.097759613	
27	41.95960341	0.521960782	
28	-17.481201	0.769545174	
29	46.78939617	0.763903703	
30	-40.5636997	0.787196702	
31	108.7325518	0.380922056	
32	-70.2128255	0.636771008	
33	-256.836106	0.370903953	
34	-134.342403	0.632736843	
35	314.1509453	0.107981769	
36	107.9572513	0.57131707	
37	-215.468712	0.115586843	
38	-14.5842342	0.911031908	
39	-161.150735	0.363955729	
40	289.795334	0.179052843	
41	-65.5157001	0.69361695	
42	129.7843657	0.572332786	
43	-291.310771	0.479153584	
44	-120.562335	0.776324939	
45	-0.85087631	0.997128333	
46	-176.236432	0.478235609	
47	51.61727979	0.681634155	
48	17.38799964	0.884422869	
49	94.12919104	0.159180134	
50	100.3819719	0.142626092	
51	113.657678	0.333990969	
52	140.9270827	0.230669929	
53	114.011927	0.101738592	
54	106.6371761	0.091480189	
55	-55.9549511	0.718954098	
56	-92.1806122	0.559143056	
57	233.3139995	0.268420724	

58	263.9434505	0.259273112
59	47.42079621	0.69815384
60	102.9228114	0.420503083
61	-137.993418	0.553139851
62	-282.678249	0.247094913
63	-106.474711	0.192974143
64	-9.12836442	0.920899947
65	66.25717558	0.644884703
66	-95.7950973	0.474583922
67	96.67098209	0.241008174
68	273.7078966	0.001853541
69	-15.6494895	0.726077569
70	45.62893985	0.21888447
71	-83.992922	0.581174807
72	-128.950257	0.410447837
73	29.7362896	0.553765753
74	104.2369498	0.022648957
75	2.386292463	0.981610691
76	-99.5230552	0.258680969
77	56.57291285	0.541374567
78	-24.9230444	0.82718819
79	1.747609449	0.988310539
80	55.39254139	0.639941266
81	-48.8011921	0.34632759
82	-51.9810669	0.297742225
83	91.54471375	0.373093407
84	-16.9245818	0.874891042
85	-106.078193	0.232559857
86	62.87512928	0.444418827
87	-4.73533539	0.907715378
88	74.41422843	0.071752179
89	54.85708924	0.286774463
90	4.881346057	0.897699897
91	48.96281566	0.595206882
92	11.76886515	0.845494855
93	-19.9288965	0.400517306
94	-30.4572029	0.341243119
95	12.27545385	0.96358844
96	-357.971122	0.166377204
97	-52.3096123	0.380369878
98	26.6590886	0.666879633
99	25.05604792	0.862327833
100	-19.4604726	0.897025678
101	58.58840471	0.526444989
102	0.987017263	0.992095625
103	6.342953353	0.463486728
104	14.04630622	0.151908751
105	-11.8286964	0.876800583
106	60.22656331	0.445187319
107	-22.8017352	0.596159095
108	1.261150554	0.978539823
109	4.739813723	0.829420206
110	-8.97663809	0.682861111
111	-237.056937	0.148439655
112	-31.4129331	0.59860668
113	-27.409876	0.670000822
114	43.8067692	0.574427281
115	-1.58757031	0.983381644

116	-42.8894528	0.831761454
117	-32.0409936	0.533293288
118	-53.8963374	0.785722056
119	-14.9294367	0.962142496
120	-0.33620868	0.340533271
121	20.58475503	0.951383185
122	-391.536992	0.107171996
123	-13.6362121	0.423759659
124	-208.315137	0.377760414
125	-137.430497	0.306492329
126	-5.98302124	0.323246573
127	-217.196761	0.126921138
128	-51.2936829	0.710092608
129	-28.2652369	0.428989364
130	-151.602404	0.301689731
131	-51.2802871	0.623757419
132	11.47877258	0.529198361
133	12.30995573	0.913220542
134	-19.895754	0.810262968
135	9.173718636	0.63522572
136	42.62366158	0.639338796
137	-26.5387631	0.101279854
138	-5.79077448	0.533988286
139	-27.3024708	0.093710342
